# Synaptic Ca2+ channels and neurexins are linked through direct and indirect binding complexes

**DOI:** 10.1038/s41598-026-62077-2

**Published:** 2026-07-15

**Authors:** Nils Hohaus, Carsten Reissner, Markus Missler

**Affiliations:** Institute of Anatomy and Molecular Neurobiology, University of Münster, Münster, Germany

**Keywords:** Biochemistry, Neuroscience

## Abstract

Rapid release of neurotransmitters from presynaptic boutons is essential for brain function and is triggered by Ca^2+^ influx through voltage-gated calcium channels (VGCCs), which consist of an α1 pore-forming subunit, intracellular β subunits, and mostly extracellular α2δ auxiliary subunits. Neurexins (Nrxn) regulate neurotransmission and presynaptic Ca^2+^ influx, but the physical interactions with VGCC subunits remain unknown. Here, we examined these interactions in recombinant VGCC-Nrxn complexes using a nanobody-based co-precipitation system. We found that the α2δ-1 and α2δ-3 variants bind to the α1 pore-forming subunits of Ca_V_2.1- and Ca_V_2.2-type VGCCs with distinct preferences, whereas Nrxn1α and Nrxn1β do not directly interact with α1. Since Nrxn1α binds both α2δ variants but Nrxn1α/α2δ complexes do not include α1, mobile α2δ subunits may dynamically toggle between Nrxn1α and the Ca_V_ core. Additionally, Nrxn1α associates with α1 subunits independently of α2δ through the intracellular scaffold protein Mint2, which enhances Nrxn1α/α2δ complex formation by inhibiting full glycosylation of α2δ. Extracellularly shorter Nrxn1β cannot bind α2δ but can indirectly associate with Ca_V_2 α1 pore-forming subunits via either Mint2 or CASK proteins. Therefore, our findings reveal distinct molecular complexes through which αNrxn and βNrxn variants interact with VGCC subunits to regulate presynaptic Ca^2+^ influx. (196 words)

**Supplementary Information:**

The online version contains supplementary material available at 10.1038/s41598-026-62077-2.

## Introduction

Synaptic transmission is vital for neuronal communication, and many molecular interactions regulate it at the presynaptic bouton, across the synaptic cleft, and in the postsynaptic compartment. αNrxn and βNrxn variants are likely involved in this regulation across all three levels because they are primarily presynaptic transmembrane proteins that form trans-synaptic interactions with various postsynaptic partners^1,2^^,3^. In the presynaptic bouton, Ca^2+^ influx through high-threshold voltage-gated calcium channels (VGCCs) is a crucial step for action potential-driven transmitter release^4^. Synaptic strength and synchronous release depend on the subtype, number, activity, and topography of mainly Ca_V_2.1 (P/Q-type) and Ca_V_2.2 (N-type) VGCCs^5,6^^,7^. VGCCs consist of a large pore-forming Ca_V_ α1 subunit with four homologous transmembrane domains that determine the channel subtype, and two associated auxiliary subunits, a cytosolic Ca_V_ β and a mostly extracellular Ca_V_ α2δ, which regulate channel abundance at the plasma membrane and the biophysical properties of the channel^8^.

The glycosylated, membrane-anchored α2δ proteins are conserved across species (α2δ−1 to α2δ−4 in mammals), serve as drug targets, and mutations in these molecules are linked to neuropsychiatric disorders^9,10^. Atomic structures of Ca_V_2.1 and Ca_V_2.2 complexes^11,12^ and mutagenesis studies at the interface of α1 and α2δ subunits show that the divalent metal ion-dependent adhesion site (MIDAS) loop of all α2δ isoforms binds similarly to the VSDII of all VGCCs^13,14^^,15^. The common binding site might explain why deleting α2δ−1 to −3 in excitatory synapses can be rescued by any of the three α2δ variants^16^. Despite this redundancy, α2δ−1 and α2δ−3 differ in their first cache domain, causing α2δ−1 to bind gabapentinoids but not α2δ−3^17^. We found that Nrxn are involved in regulating VGCC-dependent neurotransmitter release^18^. Although α2δ proteins may play a specific role in this regulation through functional and physical interactions with Nrxn^19^^[,20^, our understanding of the molecular determinants of the interaction between synaptic VGCC subunits and Nrxn remains incomplete.

Nrxns are components of transsynaptic nanocolumns that span the synaptic cleft, aligning presynaptic readily releasable vesicles and VGCCs opposite postsynaptic receptors^21–23^^,24^. They are encoded by three genes in vertebrates (*Nrxn1-3*), each with independent promoters that regulate transcription of αNrxn and βNrxn, and additional variants arise from up to six conserved splice sites^25,26^^,27^. Extracellularly, αNrxn proteins consist mainly of six laminin-Nrxn-sex-hormone-binding (LNS) domains interspersed with epidermal growth factor (EGF)-like repeats. βNrxns differ by expressing a β-specific, 37-residue N-terminal domain before splicing into the last (sixth) LNS domain of the respective gene^1^^,2^. Because LNS6 and subsequent sequences are identical in α- and β-Nrxns, they share features such as a C-terminal PDZ recognition motif necessary for intracellular trafficking^28^^,29^, a heparan sulfate glycan moiety^30^, and physiological ectodomain cleavage^22^. α- and β-Nrxns also share extracellular binding partners such as neuroligins^31,32^^,33^, leucine-rich repeat transmembrane neuronal proteins (LRRTMs)^34,35^^,36^, α-dystroglycan^37,38^, latrophilins^39^, and cerebellins^40^^[,41^. Intracellularly, Nrxns bind to Mint (also known as X11 and Apba^42–44^, CASK^43–45^, and Synaptotagmin^46^.

The functional link between Nrxns and VGCCs was observed in both constitutive and conditional knockout mouse models of αNrxn and βNrxn: presynaptic Ca^2+^ influx and Ca^2+^ channel-dependent evoked release are decreased at synapses in various brain regions of mice lacking either α-Nrxns^18,19,47,48^, β-Nrxns^49^^,50^, or both variants^48^^,51^. However, some studies of neuron subpopulations lacking all Nrxn variants did not find decreased presynaptic Ca^2+^ influx, such as at terminals of parvalbumin-positive interneurons in the medial prefrontal cortex^51^ or the parvalbumin-positive excitatory calyx synapses in the brainstem^52^. A possible reason for this discrepancy is that the connection between Nrxn and VGCCs involves specific combinations of Nrxn variants and Ca_V_ subtypes that vary across brain regions and synapse types. More research is needed to clarify the direct and indirect binding options and preferences among members of these key synaptic protein families.

Here, we focused on functional divergent α2δ−1 and α2δ−3 isoforms, and Nrxn1. Our previous study by Brockhaus et al. demonstrated that expression of Nrxn1α rescued not only Ca^2+^ influx in triple α-Nrxn knockout neurons but also revealed synergistic effects when co-expressed with α2δ−1^19^. We found that Nrxn1α binds to α2δ−1 and α2δ−3, but not directly to the α1 pore-forming subunits of Ca_V_2.1 or Ca_V_2.2. We discovered that α2δ−1 preferentially binds to Ca_V_2.1, whereas α2δs associate equally with Nrxn1α, mainly to the three N-terminal LNS domains. Although the α2δ binding site on Nrxn1α also depends on glycosylation in the stalk region, which is conserved in Nrxn1β, Nrxn1β cannot directly interact with α2δ proteins. We also confirmed that both Nrxn1α and Nrxn1β have an α2δ-independent, indirect mechanism for interacting with α1 subunits through intracellular adaptor proteins. Consistent with an earlier study^53^, the PDZ proteins CASK or Mint1/2 mediate the interaction between Nrxn1β and the α1 core complex. More unexpectedly, Nrxn1α relies only on Mint, affecting its binding to Ca_V_ subunits in two ways. Overall, the variety of direct and indirect binding interactions between Nrxn and Ca_V_ subunits revealed here will be crucial for understanding the complex regulation of synaptic strength and plasticity.

## Results

###  Pore-forming α1 subunits show preferences for α2δs but do not bind αNrxn

**Fig. 1:**
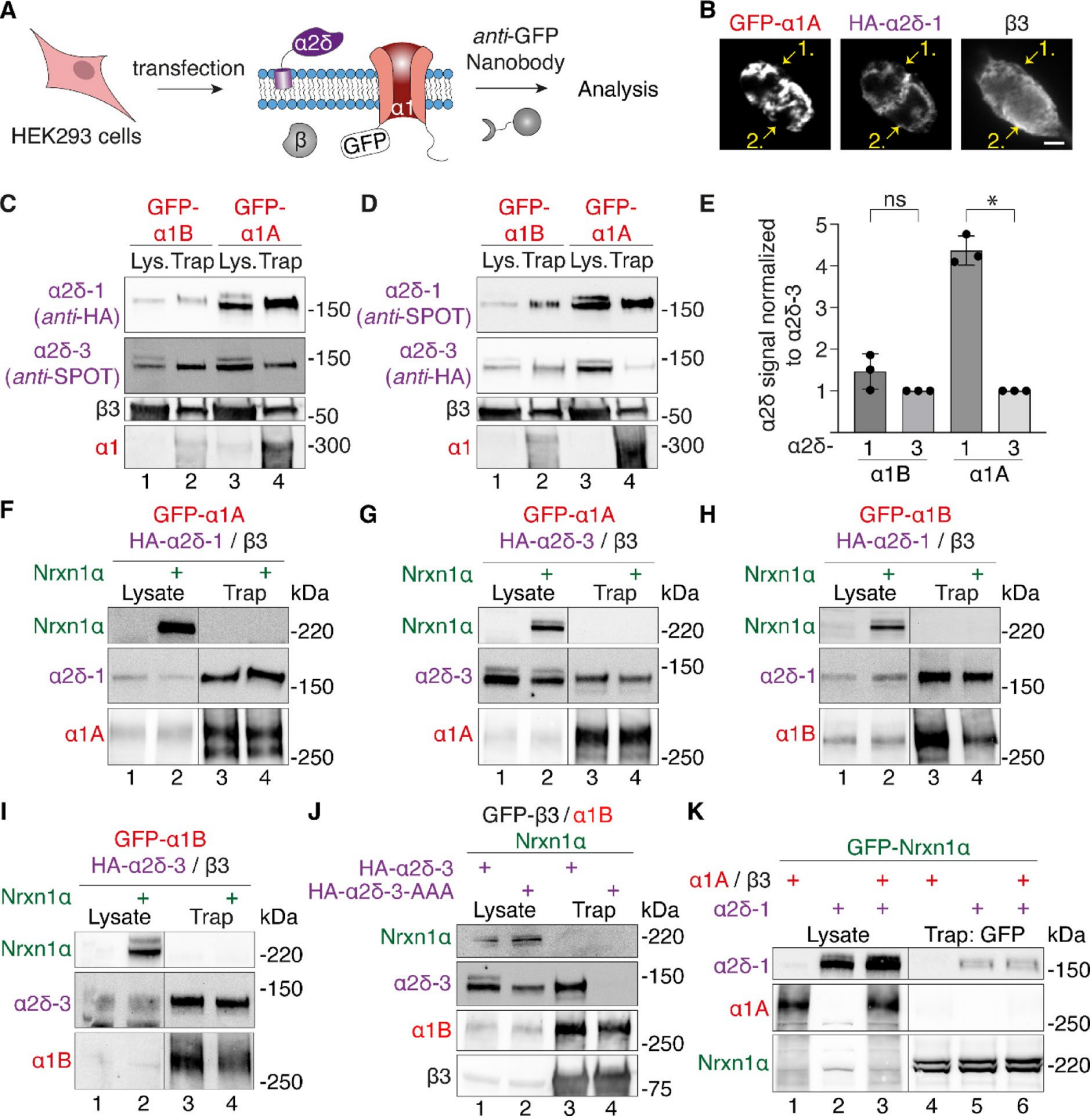
α1 subunits possess preferences towards α2δ but do not bind Nrxn1α. (**A**) HEK293 cells were transfected with VGCC subunits for subsequent analysis of digitonin lysates using an anti-GFP nanobody trap. (**B**) Representative fluorescent images of HEK293 cells expressing GFP-α1A with primary mouse anti-HA antibody labeling of HA-α2δ-1 and β3 labelling with a primary rabbit anti-β3 antibody. Images are maximum intensity Z-projections, which were made from a 13 slice Z-stack (0.7 μm slice-1). Yellow arrows mark the presence of two cells (1. and 2.). Scale bar, 5 μm. (**C**) Co-expression of HA-α2δ-1 and SPOT-α2δ-3 for analysis of competitive binding to either GFP-α1A or GFP-α1B. (**D**) The experiment in C was repeated, but tags on the α2δ subunits were swapped. (**E**) Quantified ratio of α2δ-1 to α2δ-3 binding to α1B or α1A. (**F**) Nrxn1α does not bind to GFP-α1A—antibodies: Nrxn1α (anti-Nrxn1/2/3), α2δ-1 (anti-HA), and the α1A subunit (anti-α1A). (**G**-**I**) The experiment in F was repeated with different combinations of α2δ-1, α2δ-3, and GFP-labeled α1A (anti-α1A) or α1B (anti-α1B). (**J**) GFP-β3 binds to α1B in complex with HA-α2δ-3 but not to HA-α2δ-3-AAA MIDAS mutant or Nrxn1α. (**K**) GFP Nrxn1α binds to α2δ-1 but not to α1A. Original, uncropped blots are presented in the supplementary data file.

To analyze how Nrxn variants physically interact with subunits of Ca_V_2.1 or Ca_V_2.2 channels and influence presynaptic Ca^2+^ influx, we used recent nanobody technology combined with magnetic beads. These camelid antibody fragments target known epitope tags, allowing for specific and reliable pull-down of recombinant proteins. We co-transfected HEK293 cells with GFP-tagged α1A or α1B pore-forming subunits along with β3 and α2δ−1 or α2δ−3 auxiliary subunits to reconstitute typical Ca_V_2.1 or Ca_V_2.2 channels (Fig. 1A and B). To confirm known interactions among these Ca^2+^ channel subunits, we precipitated the pore-forming α1 subunits using *anti-*GFP nanobodies. Building on a previous purification study using *anti-*α1 antibodies in mouse brain^54^, we primarily used digitonin for lysis and verified the presence of auxiliary β and α2δ subunits by immunoblotting (Figs. 1C and D). Our nanobody-based method successfully purified the complete core subunits of these recombinant Ca_V_2.1 or Ca_V_2.2 channels. Previous studies showed all α2δ isoforms can compensate for each other^16^, but α2δ−1 is more functionally related to α1A^16,55^. To test whether these differences are caused by binding, we competitively co-overexpressed α2δ−1 and α2δ−3 together with either α1A or α1B (Figs. 1C and D). Unexpectedly, we noticed a binding preference: α1A subunits enriched α2δ−1 significantly more than α2δ−3 in the trap condition compared to lysates (Fig. 1C, panels 1–2, lane 2 vs. 4). To determine if the epitope tag on α2δ proteins affected this preference, we conducted experiments swapping the SPOT- or HA-tags between α2δ−1 and α2δ−3 (Fig. 1C: HA-α2δ−1, SPOT-α2δ−3; Fig. 1D: SPOT-α2δ−1, HA-α2δ−3), with consistent results. Repeated experiments showed that the ratio of α2δ−1 to α2δ−3 signals after precipitation by α1A was approximately three-times higher than with α1B, normalized to the lysates (Figs. 1E and 4.36 ± 0.35, *p* = 0.0131 vs. 1.46 ± 0.42, *p* = 0.2055). The C-terminus of α2δ−1, including the transmembrane domain, is a few residues longer than that of α2δ−3 and has been shown to interact with NMDA receptors^56^. To explore whether this difference explains the binding preferences, we repeated α2δ−1 to α2δ−3 competition experiments with swapped C-terminal sequences; no increase in binding to α1A was observed when α2δ−3 carried the α2δ−1 C-terminus (Supplementary Fig. S1). These results show that α2δ−1 binds more effectively to the pore-forming α1A subunit than α2δ−3. Notably, the increase in α2δ−1 to α2δ−3 ratio for α1A is not driven by an exceptionally strong binding of α2δ−1, but rather by weak binding of α2δ−3. This is evident in the robust binding of α1A to α2δ−1 (Fig. 1F, lane 3), which is comparable to the binding of α1B to both α2δ−1 (Fig. 1H, lane 3–4) or α2δ−3 (Fig. 1I, lane 3–4). Interestingly, we did not observe an increased association of the β3 subunit with α1Β pore-forming subunits, as seen in earlier co-purification studies from mouse brain^54^. Considering the preferential association of α1Α with α2δ−1, the following experiments were performed expressing only a single α2δ at a time to avoid potentially confounding effects.

Removing the single Nrxn1α variant from hippocampal neurons of conditional knockout mice is sufficient to reduce presynaptic Ca^2+^ influx by nearly 18%^48^. This may account for the decreased excitatory synaptic strength and behavioral abnormalities^57,58^. A proteomic analysis of the nano-environment of Ca_V_2.x channels identified Nrxn1α at low abundance among 200 proteins that met the set stringency filters^54^, but it did not clarify its connection to the Ca_V_2.x core. After GFP-α1A purification using nanobodies, β3 (not shown) and α2δ−1 co-precipitated as expected (Fig. 1F, panels 2–4). In contrast, Nrxn1α did not co-precipitate with α1A (Fig. 1F, panel 1). To rule out the possibility that this was due to an “unsuitable” α2δ variant, we repeated the experiment including α2δ−3, but no Nrxn1α was recovered (Fig. 1G, panel 1). These findings suggest that Nrxn1α, unlike α2δs, which co-precipitated strongly, does not directly bind to the α1A pore-forming subunit. Since removing a single Nrxn1α variant from hippocampal neurons altered Ca_V_2.2-mediated Ca^2+^ influx^48^ and proteomics showed a moderate preference of Nrxn1α for Ca_V_2.2 nano-environments^54^, we also repeated the experiment with GFP-α1B pore-forming subunits. However, when testing the α1B pore-forming subunits for Nrxn1α binding, no co-precipitated Nrxn1α was found, regardless of α2δ−1 (Fig. 1H) or α2δ−3 (Fig. 1I). Thus, the pore-forming subunits of Ca_V_2.1 (α1A) and Ca_V_2.2 (α1B) do not directly bind Nrxn1α. This raised the question of whether the previously observed effects of Nrxn1α on VGCCs are due to binding of Nrxn1 to auxiliary VGCC subunits instead.

Since the β subunit is essential for cell surface delivery of α1 pore-forming subunits^59^^,60^ and is usually co-purified in a tight complex with α1^11,12,14^, we always transfected α1 and β subunits together. Accordingly, GFP-tagged α1 subunits reliably precipitated the β3 subunit (Fig. 1C-D). We found that the signals for precipitated β3 were weaker than those in the lysates and confirmed its tendency to oligomerize (Supplementary Fig. S2A), thereby limiting the amount of β3 monomers available to bind α1 subunits. The β4 isoform, which does not tend to oligomerize, was enriched when trapped by GFP-α1A or -α1B subunits (Supplementary Fig. S2B). Since β subunits can independently bind to synaptic proteins like synaptotagmin^61^ and Rim^62^, we tested whether GFP-tagged β3 can bind Nrxn1α. The β3 subunit precipitated α1B (Fig. 1 J, panel 3, lanes 3–4) and α2δ−3 (Fig. 1 J, panel 2, lane 3), as expected, but not Nrxn1α when expressed together with all VGCC subunits (Fig. 1 J, panel 1, lane 3) or when expressed alone (Supplementary Fig. S2C). These data suggest that the VGCC complex forms through a series of physical interactions, specifically β3→α1B→α2δ−3^14^, but the complex does not directly interact with Nrxn1α. To demonstrate that α2δ−3 binds to α1B in our complex solely via the previously described interaction MIDAS region^13,14,63,64^^,65^, we tested a triple alanine mutation shown to prevent binding to α1. Consistent with earlier reports, α2δ−3-AAA did not bind to a GFP-β3/α1B complex in our nanobody-mediated GFP-trap experiments (Fig. 1 J, panel 2, lane 4).

The results above show that the VGCC core subunits in the complex apparently cannot directly interact with Nrxn, as neither GFP-α1 nor GFP-β subunits used as baits pulled down Nrxn1α. This finding is surprising, given our earlier data that clearly demonstrated an effect of deleting or overexpressing either a single Nrxn1α or all αNrxn on Ca^2+^ influx^19,48,66^. To rule out the possibility that co-purification of Nrxn1α depended on specific conditions, we performed the reverse experiment using GFP-tagged Nrxn1α as bait (Fig. 1 K). Nanobody-mediated pulldown of Nrxn1α strongly enriched the molecule under our purification conditions (Fig. 1 K, panel 3), but it did not pull down the α1A pore-forming subunit (Fig. 1 K, panel 2) or the β3 auxiliary subunit (not shown), supporting earlier data (Fig. 1F–J). However, when we co-transfected the α2δ−1 auxiliary subunit alone (Fig. 1 K, panel 1, lane 5) or with β3 and α1A (Fig. 1 K, panel 1, lane 6), α2δ was pulled down by GFP-tagged Nrxn1α. These findings suggest Nrxn1α can directly bind to an α2δ auxiliary subunit but not to the core α1 and β subunits of Ca_V_2. The physical interaction between Nrxn1α and α2δ−1 might explain their particularly strong effect on presynaptic Ca^2+^ influx observed in rescue experiments with αNrxn-deficient neurons when co-expressed (Fig. 6 C in Brockhaus et al.^19^. Unlike its strong enrichment by GFP-α1A (Fig. 1 F, panel 2), only moderate amounts of α2δ−1 were co-precipitated by GFP-tagged Nrxn1α under the same conditions (Fig. 1 K, panel 1). This raises the question of whether the Nrxn1α/α2δ association is exclusive or can be part of a VGCC core complex.

### Nrxn1α binds to α2δ subunits independent of pore-forming α1

**Fig. 2:**
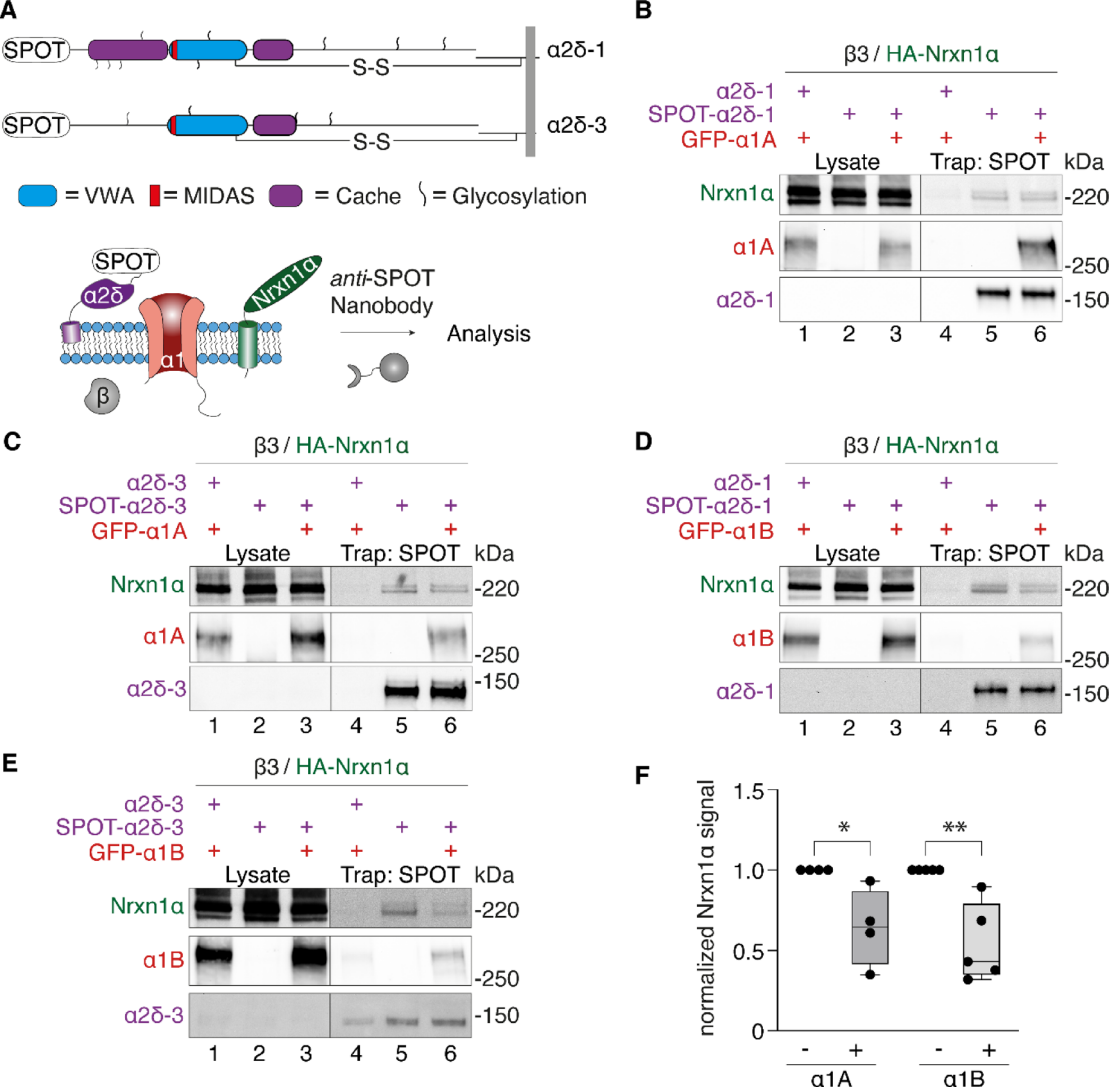
Nrxn1α binds directly to α2δ and competes for α1 subunit binding. (**A**) Domain arrangement of N-terminally SPOT-tagged α2δ-1 and α2δ-3, used as bait proteins (**B**-**E**) to precipitate target proteins from co-transfected HEK293 cell lysates via anti-SPOT nanobodies. B SPOT-α2δ-1 was transfected with α1A, β3 (not shown), and HA-Nrxn1α into HEK293 cells, and digitonin lysates were subjected to SPOT trap to investigate precipitation of VGCC subunits and Nrxn1α. α2δ-1 was probed with anti-α2δ-1 antibody, and HA-Nrxn1α was detected using anti-HA. C–E Similarly to B, SPOT-tagged α2δ-1 or α2δ-3 were used as bait to examine precipitation of HA Nrxn1α, β3 subunit (not shown), and either α1A or α1B from HEK293 cell lysates. All Western blot data (B–E) are shown with one representative image from at least two independent experiments. (**F**) Densitometric quantification of Nrxn1α precipitated by SPOT-α2δs in the presence of an α1 subunit, normalized to precipitation in its absence. Kruskal-Wallis with post-hoc Dunn’s test; **p = 0.0059; *p = 0.0323; α1A, *n* = 4; α1B, *n* = 5. Original, uncropped blots are presented in the supplementary data file.

To test the hypothesis that Nrxn1α can bind to an α2δ subunit but does not form a direct complex with the α1 pore-forming subunits, we designed a different experiment. We checked whether α2δ subunits could co-precipitate both Nrxn1α and other Ca_V_2.x core subunits at the same time. We used 12-amino acid SPOT-epitope tags^67^ attached to α2δ−1 and α2δ−3 subunits along with *anti-*SPOT nanobodies for precipitation (Fig. 2 A), including untagged α2δ−1 and α2δ−3 as negative controls. The controls did not precipitate with *anti-*SPOT nanobodies (Fig. 2B, panel 3, lane 4), confirming the method’s specificity. The α1A subunit of Ca_V_2.1 was reliably co-precipitated with SPOT-α2δ−1 (Fig. 2B, panel 2, lane 6) or SPOT-α2δ−3 (Fig. 2 C, panel 2, lane 6), as was the auxiliary β3 subunit (not shown). The same result was observed for the α1B subunit of Ca_V_2.2 with SPOT-α2δ−1 or SPOT-α2δ−3 (Fig. 2D–E). Importantly, we detected co-precipitation of Nrxn1α with α2δ subunits even without an α1 subunit (Fig. 2, B–E, panel 1, lane 5), confirming Nrxn1α binds to α2δ but not to the pore-forming subunit, consistent with previous data (Fig. 1 K). Conversely, when paired with an α1 pore-forming subunit, α2δ precipitated both the α1 subunit and Nrxn1α (Fig. 2B–E, lane 6). Additionally, densitometric analysis showed that Nrxn1α precipitation by α2δ decreases when co-transfected with an α1 subunit (Fig. 2 F) - about 35% decrease with α1A (0.64 ± 0.24, *p* = 0.0323) and over 40% with α1B (0.54 ± 0.24, *p* = 0.0059). This suggests that α2δ may bind either to Nrxn1α or to α1 pore-forming subunits, but not both at the same time. One possible explanation for this competitive behavior is the use of the same binding epitope. Since α2δ subunits bind their MIDAS loop to α1 pore-forming subunits, we tested whether Nrxn1α uses the same site for binding to α2δ. We confirmed that the MIDAS motif is necessary for α2δ−1 to bind to α1 (Supplementary Fig. S3A, panel 1, lane 4), but it is not needed for Nrxn1 binding to α2δ−1 (Supplementary Fig. S3B, panel 1, lane 7) or α2δ−3 (Supplementary Fig. S3B, panel 1, lane 8). Overall, our results from GFP- (Fig. 1) and SPOT- (Fig. 2) trap experiments suggest that Nrxn1α competes with α1 subunits for binding to α2δ auxiliary subunits but probably connects to α2δ at a different epitope. Therefore, we decided to further explore the molecular factors that govern Nrxn1α binding to α2δ in the absence of α1 subunits.

### α2δ/Nrxn1α complexes are present at the cell surface

Using single-particle tracking techniques, we previously showed that deleting αNrxn slightly affects the axonal surface mobility of α2δ subunits in neurons^19^, indicating that they engage in weak, transient interactions at the cell surface. Additionally, our physical interaction assays presented above revealed that only small amounts of Nrxn1α and α2δ were precipitated in trap experiments compared to lysates, regardless of which molecule was used as bait (Figs. 1 K and 2B–E). To confirm that these Nrxn1α/α2δ complexes are present on the cell surface, we developed a triple-split GFP approach that specifically isolates and fluorescently visualizes them on the cell surface.

**Fig. 3:**
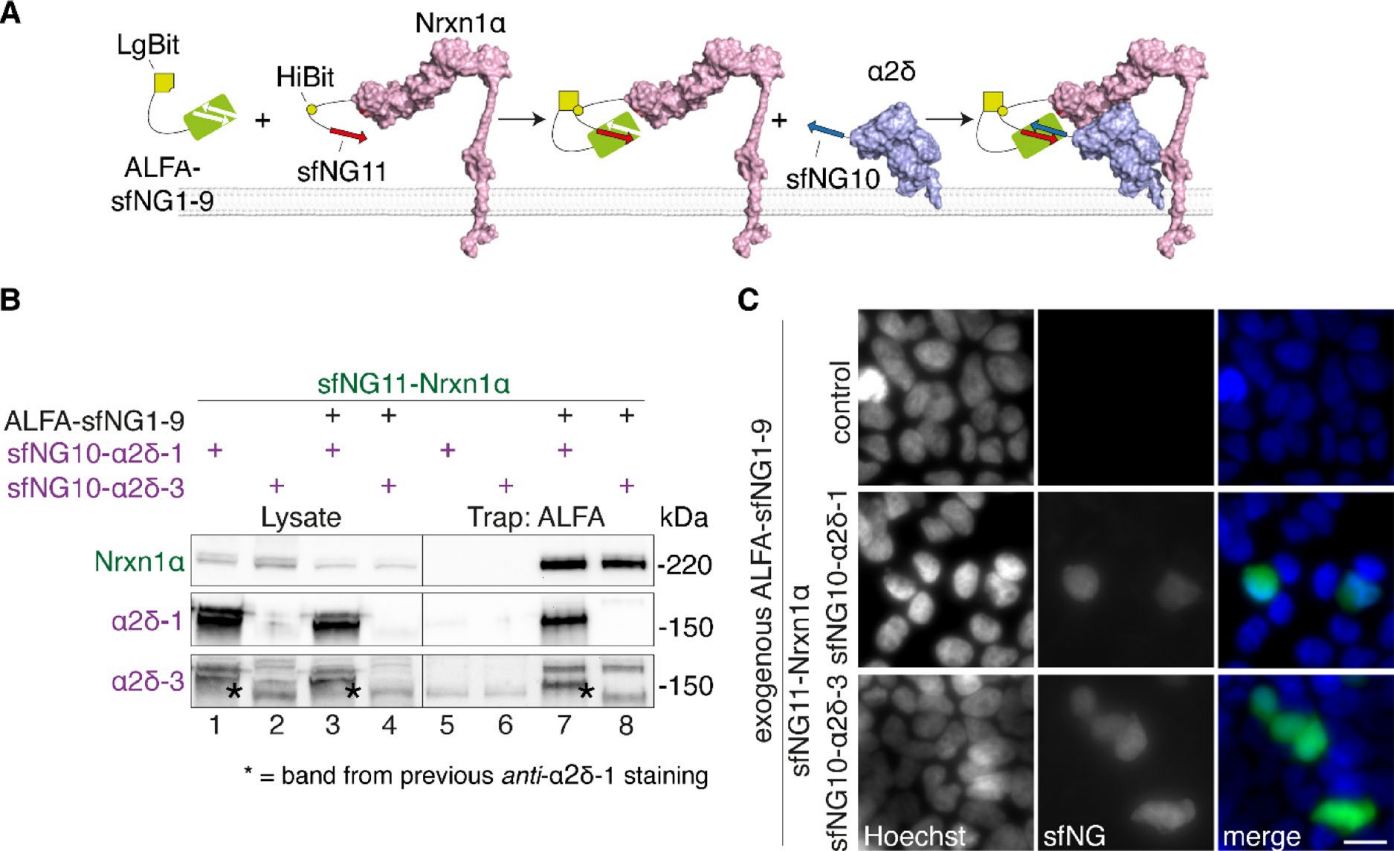
Nrxn1α and α2δ reconstitute in a triple-split sfNG model. (**A**) An extracellular serial reconstitution of high-affinity HiBit and LgBit fragments to a luciferase domain, followed by a triple NeonGreen (NG) split. (**B**) Anti-ALFA trap from HEK293 cell lysates containing sfNG11-Nrxn1α and sfNG10-α2δ-1 or -α2δ-3, in the absence or presence of ALFA-sfNG1-9. (**C**) HEK293 cells expressing sfNG11-Nrxn1α along with sfNG10-α2δ-1 or -α2δ-3 were supplied with 2 μg ml-1 purified sfNG1-9, and reconstituted sfNG fluorescence was imaged after 16 hours. Fluorescent signal of non-transfected cells treated with sfNG1-9 was used to determine non-specific fluorescence, which was subtracted from sample images. Scale bar, 20 μm. Original, uncropped blots are presented in the supplementary data file.

Previous split-GFP techniques used to study the formation of trans-synaptic complexes with Nrxn1α^68^ have two main limitations: (i) attaching large peptide segments to proteins of interest (POIs) can disrupt their proper folding^69,70^, and (ii) the assembly of a two-part GFP is irreversible and can occur prematurely, driven by the GFP segments rather than the POIs, leading to false-positive results^71^. To address these issues, we adapted a recent triple-split GFP method^72^. This approach keeps the two POIs inside the cell unaffected until a third GFP fragment completes the process, thereby fluorescently highlighting the reconstituted complexes. This system is especially effective for co-secreted proteins, as their complex formation becomes visible only at the cell surface, preventing premature visualization in the endoplasmic reticulum, Golgi apparatus, and inside axons traveling together in the same transport vesicle^29^. The superfolder NeonGreen (sfNG) system uses highly affine luciferase fragments, HiBit and LigBit, turning the three-part reconstitution into a sequential two-step reaction (Fig. 3A). First, the N-terminal 12-residue HiBit sequence fused to Nrxn1α binds to the LgBit domain of LgBit-sfNG1-9. This binding allows the β-strand 11 from sfNG, also fused to Nrxn1α, to attach to sfNG1-9, forming sfNG1-9–11. When α2δ binds to Nrxn1α, sfNG10 is positioned close enough to complete the sfNG structure. We added an ALFA-tag^73^ to sfNG1-9 to isolate only reconstituted Nrxn1α/α2δ complexes from HEK293 cell lysates using *anti-*ALFA nanobodies (Fig. 3B). As expected, we observed efficient co-precipitation of α2δ−1 (Fig. 3B, lane 7) or α2δ−3 (Fig. 3B, lane 8) along with Nrxn1α (Fig. 3B, lanes 7–8) in the presence of ALFA-sfNG1-9, but not without it (Fig. 3B, lanes 5–6). These complexes could also be seen on the surface of HEK293 cells after live labeling with purified recombinant sfNG1-9. When we co-transfected cells with sfNG10-α2δ−1 or sfNG10-α2δ−3 along with sfNG11-Nrxn1α, we detected an sfNG signal. This signal was absent in cells not expressing both sfNG10-α2δ and sfNG11-Nrxn1α (Fig. 3 C). This triple-split GFP method requires additional rigorous time-dependent conditions and controls to e.g., to visualize the competitive transient interaction of Nrxn1α and α2δs to α1 subunits, but here, it independently confirmed that Nrxn1α and α2δ reach the cell surface and form a complex, raising questions about the molecular determinants of their interaction.

**Fig. 4:**
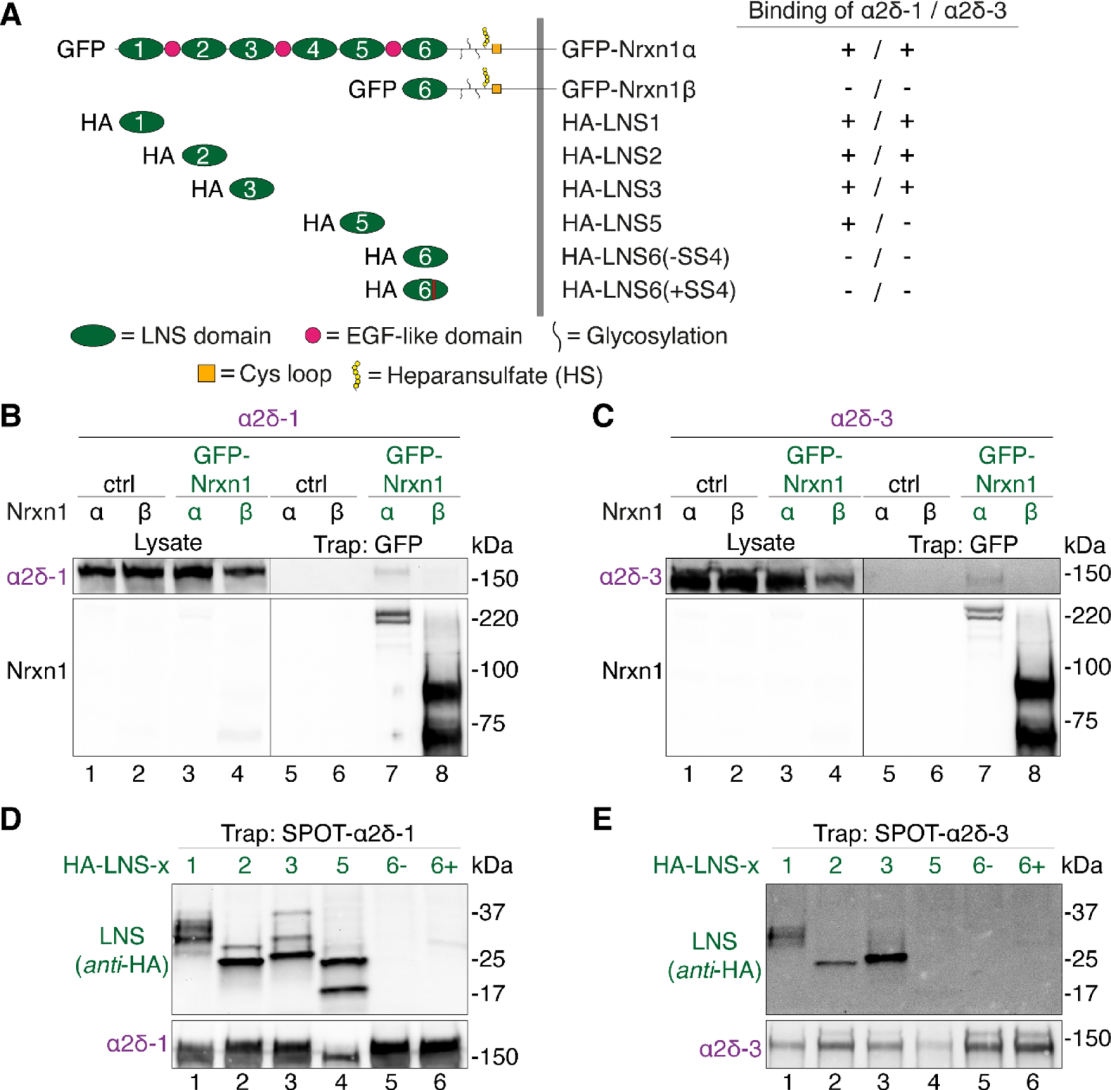
Several LNS domains of Nrxn1α participate in binding α2δ subunits. (**A**) Nrxn1 domains were used to test binding to α2δ-1 and α2δ-3 subunits. Note that the isolated LNS4 domain could not be tested due to technical difficulties. (**B**) GFP-tagged Nrxn1α but not Nrxn1β precipitates α2δ-1, indicating that binding is mediated by the non identical N-terminal domains containing LNS1-5. (**C**) Similar to B but with α2δ-3. (**D**) Full-length SPOT-α2δ-1 precipitates distinct HA-tagged LNS domains of Nrxn1α when co-expressed in HEK293 cells. (**E**) Similar to D but with α2δ-3, which also precipitates LNS1-3 but not LNS5. Original, uncropped blots are presented in the supplementary data file.

### Several extracellular Nrxn1α domains bind redundantly to α2δ

Nrxn variants have limited but well-defined binding sites^1^. Extracellularly, several postsynaptic binding partners interact with calcium-binding sites at the LNS2 domain (Dystroglycan^37^^[,38^ or at the LNS6 domain (Neuroligins^31^^[,33^, LRRTMs^34^^[,35^, Dystroglycan^37^^[,38^. Other secreted proteins bind to the highly O-glycosylated stalk domain (CA10, CA11^74^, FAM19A^75^ or to inserts at the splice site (SS)#4 (Cbln^76^ or SS#5 (C1QL2^77^). The stalk region contains two heparan sulfate conjugation sites: a constitutive one^30^ and a splice-insertion-dependent one at SS#5^78^. Conjugated heparan sulfate binds PTPRs^79^ and LRRTM3-4^80,81^. Intracellularly, cytosolic binding to Nrxns occurs primarily at the C-terminal PDZ-binding motif via CASK, Mint, and Veli^43^.

To identify the Nrxn1 domains that bind α2δ−1 and α2δ−3, we first tested the extracellularly shorter Nrxn1β variant, which has the same structure as Nrxn1α from the LNS6 domain to the C-terminus (Fig. 4A). However, using GFP-Nrxn1β as bait did not precipitate α2δ−1 (Fig. 4B, panel 1, lane 8) or α2δ−3 (Fig. 4C, panel 1, lane 8). This suggests that the binding region depends on sequences from the N-terminus to the LNS5 domain of Nrxn1α, which are absent in Nrxn1β (Fig. 4A). Next, we co-expressed SPOT-α2δ−1 or SPOT-α2δ−3 with six isolated LNS domains fused to HA epitope tags (Fig. 4A, Supplementary Fig. S4). We included both functional LNS6 variants with and without the 30 amino acids insert in SS#4 (−4 or + 4)^1,82,83^ but had to omit LNS4 due to technical difficulties. We expected LNS6 to serve as a negative control and one of the other LNS domains to mediate the interaction. Unexpectedly, the nanobody-mediated SPOT-trap of α2δ−1 effectively precipitated four HA-tagged LNS domains (LNS1, LNS2, LNS3, LNS5), but not either LNS6 splice variant (Fig. 4D). Conversely, SPOT-α2δ−3 precipitated LNS1, LNS2, and LNS3, but not LNS5 or the LNS6 variants (Fig. 4E). The interaction involving several LNS domains is surprising because the LNS domains of Nrxns show very low sequence homology^1^. Since an unusual glycan sequence allows Dystroglycan to bind two different LNS domains (LNS2, LNS6)^37^^[,38^, the finding that up to four LNS domains bind to α2δ suggests glycosylation of either Nrxn or α2δ may play a role in their interaction. Furthermore, the fact that α2δ−3, unlike α2δ−1, did not precipitate the HA-LNS5 domain implies that specific molecular properties, possibly their glycan moieties, of these α2δ subunits explain this difference.

## Binding of Nrxn1α to α2δ is inversely related to glycosylation

**Fig. 5:**
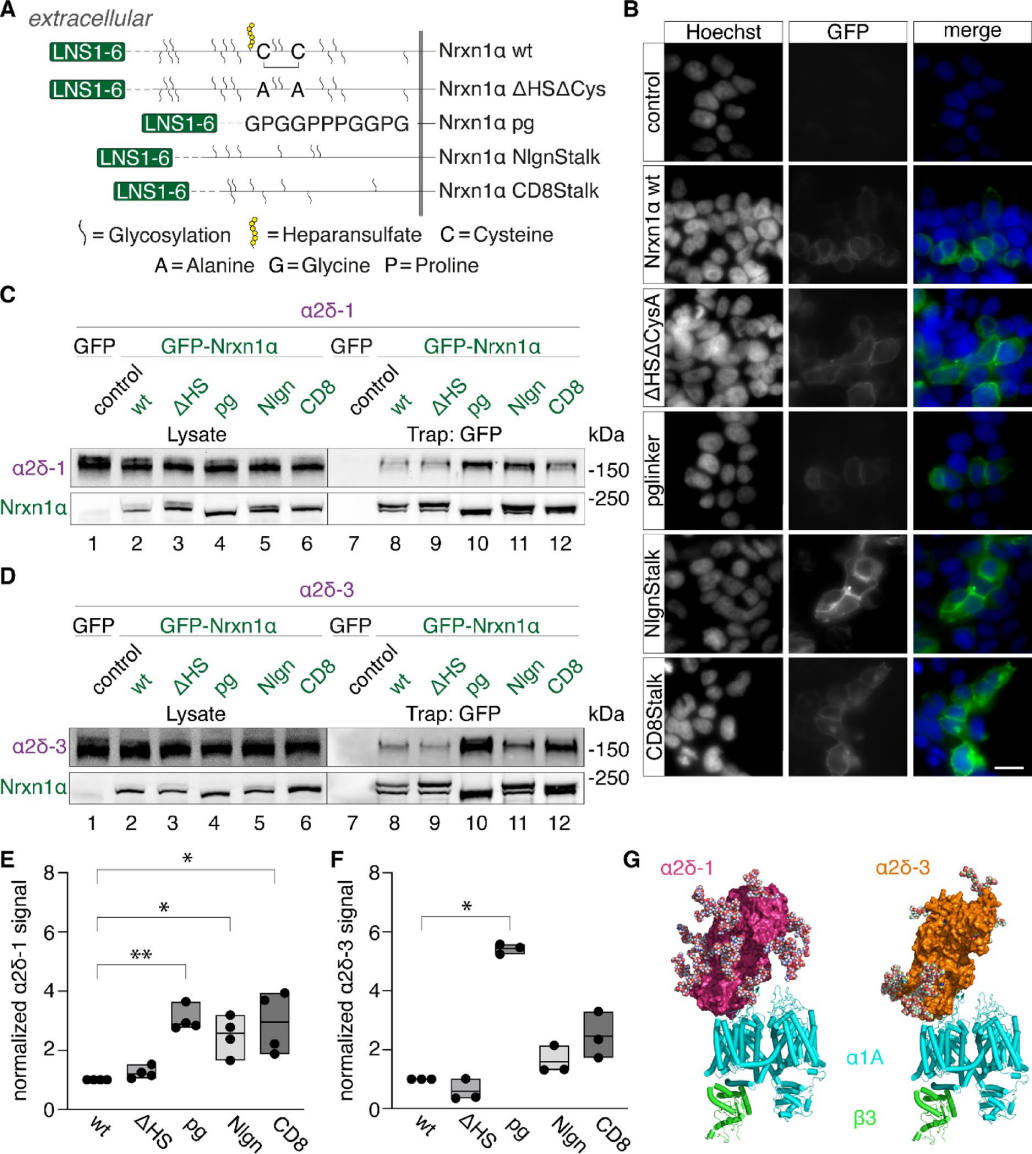
Glycosylation is inversely related to Nrxn1α binding to α2δ. (**A**) Native (wt) and mutant GFP-Nrxn1α constructs were used to precipitate α2δ-1 or α2δ-3 to examine the effect of O-Glycosylation on Nrxn1α‘s stalk domain. (**B**) Representative fluorescent images of HEK293 cells expressing GFP-Nrxn1α stalk variants. Scale bar, 20 μm. (**C**) GFP-trap of GFP-tagged Nrxn1α stalk variants co-expressed with α2δ-1 in HEK293 cells. (**D**) Similar to C but with α2δ-3. All Western blot data are representative images of at least three independent experiments. (**E**) Densitometric quantification of α2δ-1 precipitated by GFP-Nrxn1α variants, normalized to α2δ-1 available in the lysate and shown as %-change compared to precipitation by wildtype GFP-Nrxn1α (wt). Non-parametric Kruskal-Wallis with post-hoc Dunn’s test was used to determine statistical significance; *n* = 4; **p ≤ 0.01; *p ≤ 0.05. (**F**) Similar to E but for α2δ-3 precipitated by GFP-Nrxn1α; n = 3; *p ≤ 0.05. (**G**) Image depicting an N glycosylated α2δ-1/α1A/β3 model (left; based on PDB: 8X90), and a homology model with α2δ-3 (right) created with AlphaFold and Glycam server. Original, uncropped blots are presented in the supplementary data file.

Nrxn1α and α2δ proteins are heavily O- and/or N-glycosylated^38^^[,55^^[,84^. To further investigate whether glycosylation affects their binding, we first designed several mutated Nrxn constructs (Fig. 5A). Nineteen of 33 Thr/Ser sites in the stalk domain of wild-type Nrxn1 are predicted to be O-glycosylated (NetOGlyc4.0, O-GlcNAcPRED-DL), consistent with the density of O-glycosites recently identified by LC-MS/MS in the Nrxn family^85^. We reduced the number of O-glycosites by replacing the stalk region in Nrxn1α with similar regions from Neuroligin1 (Nlgn1, 6 of 19 sites glycosylated) and from CD8 (6 of 7 sites). We also completely blocked O-glycosylation using a stalk region containing a synthetic proline-glycine (pg) linker. Additionally, Nrxn1 can be conjugated to heparan sulfate (HS), increasing its molecular weight by 30 kD^30,78^. We used splice-insertion SS#5-deficient constructs and created a non-HS Nrxn1α construct (ΔHS) through site-directed mutagenesis. Furthermore, we removed the downstream cysteine loop (Fig. 5A), which binds CA10 and is linked to HS conjugation^74^. All constructs were transfected into HEK293 cells (Fig. 5B) and demonstrated adequate expression levels (Fig. 5C–D, lanes 2–6). Following GFP-trap, wild-type and mutant Nrxn1α constructs all precipitated SPOT-α2δ−1 (Fig. 5C) and SPOT-α2δ−3 (Fig. 5D), indicating that O-glycosylation or heparan sulfate conjugation are not necessary for their binding. However, differences were observed in the amount of precipitated α2δ variants under specific conditions.

We used ImageJ to measure, normalize, and compare the levels of α2δ−1 (Fig. 5E) and α2δ−3 (Fig. 5F) precipitated by mutant versus wild-type GFP-Nrxn1α. Removing the large heparan sulfate conjugation and cysteine-loop regions did not significantly change the binding of either α2δ subunit (α2δ−1: 1.24 ± 0.2, *p* > 0.99; α2δ−3: 0.59 ± 0.37, *p* > 0.99). However, replacing the Nrxn1α stalk with a completely non-glycosylated pg-linker increased binding to α2δ−1 threefold (3.04 ± 0.41, *p* < 0.01) and to α2δ−3 by fivefold (5.42 ± 0.17, *p* < 0.05). Similarly, substituting with a less glycosylated stalk from Nlgn1 or CD8 also enhanced precipitation of α2δ−1 (Nlgn: 2.50 ± 0.65, *p* < 0.05; CD8: 2.93 ± 1.03, *p* < 0.05) and α2δ−3 (Nlgn: 1.60 ± 0.48, *p* = 0.93; CD8: 2.46 ± 0.79, *p* = 0.27). These findings suggest that O-glycosylation on the Nrxn1α stalk reduces its binding to α2δ subunits, indicating a regulatory role for this post-translational modification. Because de-glycosylation of the Nrxn1α stalk region improves binding to α2δ−3 more than to α2δ−1 (Fig. 5E-F), we examined additional differences in α2δ. Since no structural data are available for α2δ−3, we modeled a Ca_V_2.1/α2δ−3/β3 complex based on a published Ca_V_2.1/α2δ−1/β3 cryo-EM structure (PDB: 8 × 90^*11*^). We replaced α2δ−1 with an α2δ−3 homology model generated using AlphaFold and then added N-glycosylation moieties to both α2δ variants using the Glycam server, assuming high-mannose, fucosylated, and sialylated complex-type N-glycans^38^. We found that the surface of α2δ−3 is less covered by N-glycosylation than that of α2δ−1 (Fig. 5G), consistent with the lower molecular weight of α2δ−3 (< 150 kDa) compared to α2δ−1 (> 150 kDa, Figs. 2 and 3, & 4). Interestingly, fully O-glycosylated Nrxn1α binds equally well to both α2δ variants, but reducing O-glycosylation at Nrxn1α enhances its interaction with α2δ−3 compared to α2δ−1. Thus, modifying the glycosylation pattern during the passage through the secretory pathway may add another layer of regulatory complexity to the interaction of Nrxn1α and α2δ proteins.

### Nrxn1α binding to α2δ depends on membrane anchoring

**Fig. 6:**
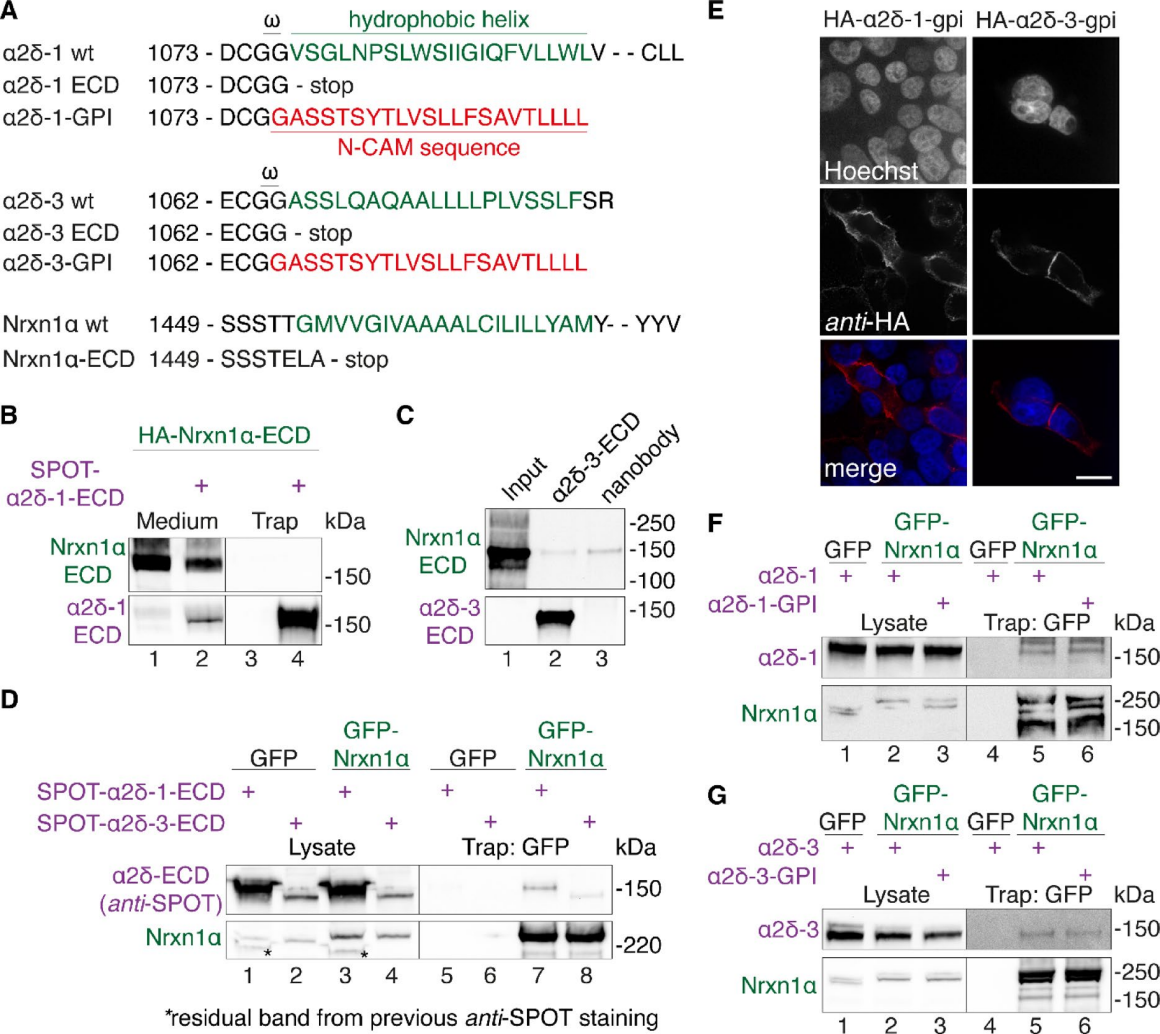
Membrane anchoring is essential for Nrxn to bind to α2δ. (**A**) Ectodomains (ECD) of α2δ-1, α2δ-3, and Nrxn1α were created by replacing their hydrophobic transmembrane domains with stop codons. Additionally, for α2δ-1 and α2δ-3, unequivocal GPI-anchored variants were produced by substituting their protein sequences with an NCAM sequence downstream of the identified ω site. (**B**) ECDs of HA-Nrxn1α and SPOT-α2δ1 were expressed in 293-F cells and secreted into the medium. Using SPOT trap, α2δ-1 was precipitated to assess the binding of Nrxn1α-ECD. (**C**) ECD of SPOT-α2δ-3 was expressed in 293-F cells and bound to SPOT-nanobody to examine the binding of 5 μg of purified HA-Nrxn1α-ECD. (**D**) GFP-Nrxn1α was expressed alongside SPOT-α2δ-1-ECD or SPOT-α2δ-3-ECD in HEK293 cells, and cell lysate was subjected to GFP trap to analyze the binding of α2δ- ECDs to membrane-anchored Nrxn1α. (**E**) Representative images of extracellular anti-HA staining to label surface HA-α2δ-GPI in HEK293 cells to demonstrate the presence of GPI-anchored proteins at the plasma membrane. Scale bar, 20 μm. (**F**) HEK293 cells were transfected with HA-α2δ-1 or its GPI-anchored variant along with GFP Nrxn1α as bait protein for GFP trap. G Similar to F, but with α2δ-3. Original, uncropped blots are presented in the supplementary data file.

We demonstrated that Nrxn1α and α2δ are highly mobile^19^ and bind to each other with low capacity (Fig. 1 K). The binding of α2δ−1 to a heteromeric NMDAR complex was shown to require the C-terminus that anchors α2δ−1 to the membrane^56,86^, whereas other interaction partners outside the VGCC complex, such as Low Density Lipoprotein Receptor-related Protein-1 (LRP1), Thrombospondins (TSPs), or large (big) potassium channel α (BKα), bind via the extracellular sequences of α2δ^87^. To test if Nrxn1α can bind α2δ fully independently of transmembrane domains, we attempted to precipitate a complex from secreted extracellular domains (ECDs) of Nrxn1α and α2δ (Fig. 6A). We detected HA-Nrxn1α-stalk-ECD and SPOT-α2δ−1-ECD in the medium of co-transfected HEK293 cells (Fig. 6B, lanes 1–2). However, pulling down SPOT-α2δ−1-ECD from the medium with *anti-*SPOT nanobodies did not co-precipitate Nrxn1α-ECD (Fig. 6B, lane 4). Similarly, both recombinantly expressed ECDs could be purified from HEK-f cell suspension cultures at concentrations up to 0.5 mg ml^− 1^ but did not combine to form a complex detectable by Western blot (Fig. 6C). As a result, the attempt to produce a large amount of stable Nrxn1α::α2δ complex with ECD failed. Interestingly, GFP-trap from lysate of co-transfected full-length GFP-Nrxn1α (instead of just ECD) with SPOT-α2δ−1-ECD or SPOT-α2δ−3-ECD resulted in complex formation of Nrxn1α tο both α2δ-ECDs (Fig. 6D), although with a low capacity comparable to full-length proteins (Fig. 2B-E, lane 5). To investigate whether the unique C-termini of α2δ affect binding to Nrxn1α, we replaced the sequences of both α2δ with a GPI anchor from NCAM (Fig. 6A and E). We found that GFP-Nrxn1α binds to HA-α2δ−1-GPI (Fig. 6F) and HA-α2δ−3-GPI (Fig. 6G), similar to the native HA-α2δ. These findings indicate that membrane anchoring at least of Nrxn1α is necessary for their interaction and is best achieved through co-secretion from the same cell.

### Mint2 indirectly connects Nrxn to α1 and decreases α2δ glycosylation

**Fig. 7:**
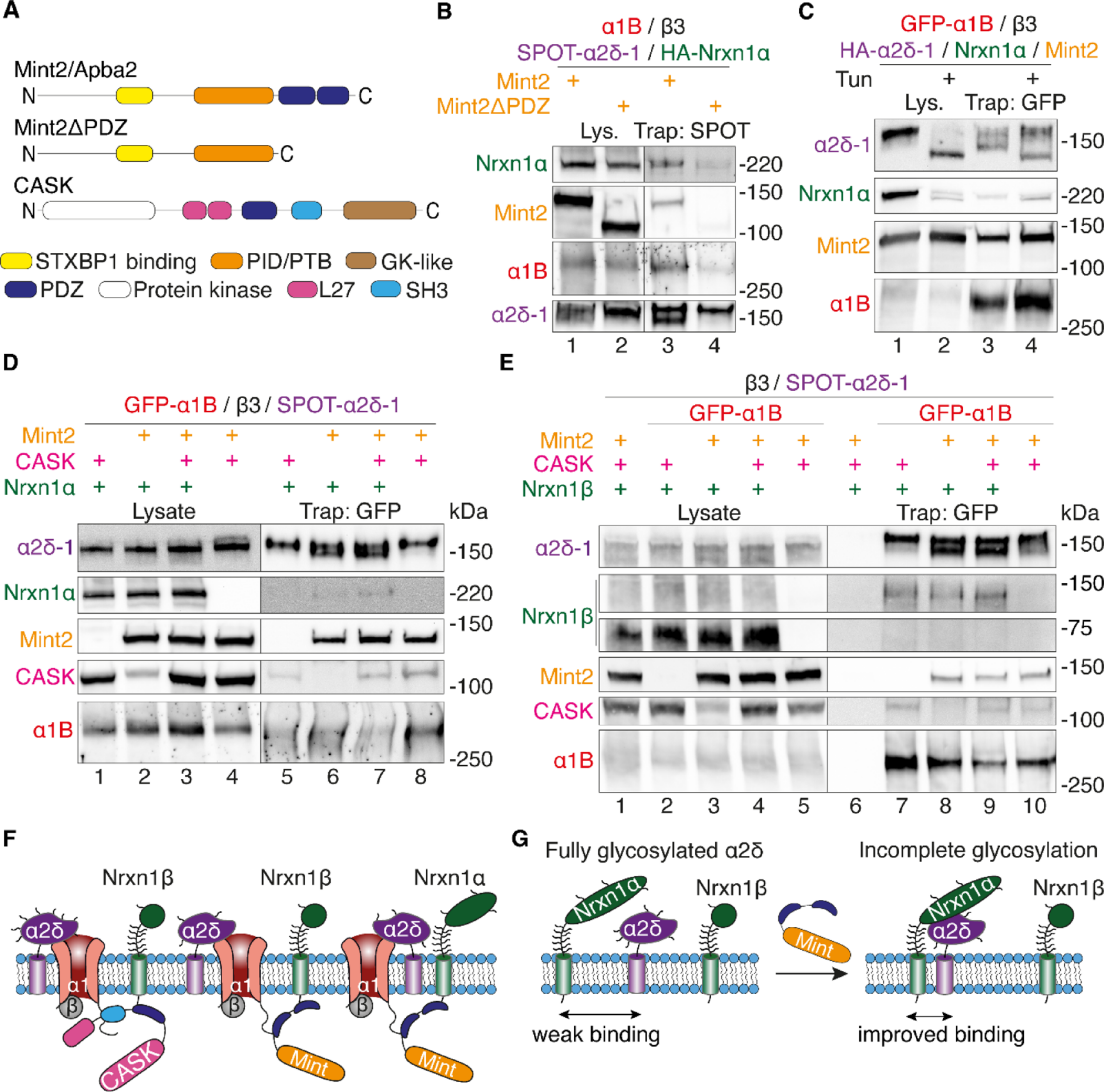
Mint2 and CASK differentially connect Nrxn1α and Nrxn1β to VGCCs (**A**) Schematic overview of the domain organization of the scaffold proteins Mint2 and CASK. Protein and domain lengths are illustrated to scale based on UniProt data (Mint2 Accession ID: O35431; CASK Accession ID: Q62915). Additionally, a truncated version of Mint2 lacking both PDZ domains (Mint2ΔPDZ) was used. (**B**) SPOT-α2δ-1, α1B, β3, and HA-Nrxn1α were transfected into HEK293 cells, and SPOT trapping was performed in the presence of either Mint2 or Mint2ΔPDZ. (**C**) HEK293 cells were treated for 48 hours with or without 2.5 μg ml-1 Tunicamycin, an inhibitor of N-glycosylation. A GFP nanobody-mediated trap of GFP-α1B/β3 in complex with α2δ-1, Mint2, and Nrxn1α. (**D**) GFP-α1B binds SPOT-α2δ-1, Mint2/Nrxn1α, Mint2/CASK, but not CASK/Nrxn1α. (**E**) Similar to D, using HA-Nrxn1β instead of HA-Nrxn1α, resulting in GFP-α1B/β3 binding to Mint2/Nrxn1β and CASK/Nrxn1β. (**F**) Mint1/2 indirectly link Nrxn1α and Nrxn1β to α1 subunit, independently of α2δs. Additionally, Nrxn1β can connect to α1 via CASK. (**G**) Mints in collaboration with Nrxn1α or Nrxn1β prevents complete N-glycosylation of α2δs within the secretory pathway and thereby increase Nrxn1α::α2δ complex formation. Original, uncropped blots are presented in the supplementary data file.

Our biochemical data showed no binding of Nrxn1β to α2δ subunits (Fig. 4B–C). This was surprising because we and others had previously demonstrated that deleting βNrxn in neurons, similar to αNrxn, impacts presynaptic Ca^2+^ transients^49,50^. To clarify this, we examined whether alternative, α2δ-independent pathways connect Nrxn to the VGCC core complex. Earlier studies indicated that the intracellular scaffold proteins CASK and Mint2 (APBA2) (Fig. 7A) can link α- and β-Nrxn to the α1 pore-forming subunits^43^^,44^^,88^. The neuron-specific Mint2 protein contains a tandem PDZ domain; PDZ1 binds the α1B subunit, while PDZ2 is thought to bind Nrxn^42,43^^,44^^,89^^,90^. To test this with our recombinant VGCC complexes, we used SPOT-Trap with SPOT-α2δ−1 as bait. This experiment co-precipitated full-length Mint2 (Fig. 7B, panel 2, lane 3), but a Mint2ΔPDZ construct lacking both PDZ domains did not bind (Fig. 7B, panel 2, lane 4). This underscores the necessity of Mint2 PDZ domains for α1 binding. Notably, we also observed increased precipitated Nrxn1α with full-length Mint2 (Fig. 7B, panel 1, lane 3) compared to Mint2ΔPDZ, which showed a lower Nrxn1α signal (Fig. 7B, panel 1, lane 4). These results suggest that SPOT-α2δ−1 may be part of a large VGCC complex where Nrxn1α is indirectly linked via Mint2 (α2δ−1/α1Β/Mint2/Nrxn1α), or it could form a direct α2δ−1/Nrxn1α complex. When Mint2ΔPDZ is present, the formation of this large complex is inhibited, resulting in a reduced amount of both Nrxn1α directly bound to α2δ−1 (α2δ −1/Nrxn1α; Fig. 7B, panel 1, lane 4) and of α1Β alternatively bound to α2δ−1 (α2δ−1/α1Β; Fig. 7B, panel 3, lane 4), as previously observed (Fig. 2).

Interestingly, with a full-length Mint2 but not with Mint2ΔPDZ, we consistently observed a second band for α2δ−1 below 150 kDa (Fig. 7B, panel 4, lane 3). Since Mint is linked to protease^91^, we investigated whether the lower-kDa variant of α2δ−1 results from proteolysis or a change in glycosylation. It has been reported that HEK293 cells produce non-processed α2δ^92^, which are not adequately cleaved into α2- and δ chains and linked by a single cysteine bridge^93^, possibly making them more prone to proteolysis. To check this, we incubated and purified the GFP-α1B::α2δ complex under reducing conditions with DTT and confirmed proper cleavage of the cysteine bridge of α2δ bound to GFP-α1 (Supplementary Fig. S5). To test the other possibility, we performed GFP-Traps of GFP-α1B co-transfected with α2δ−1, Nrxn1α, and Mint2 in the presence or absence of Tunicamycin, a blocker of N-glycosylation (Fig. 7C). Tunicamycin decreased the MW of α2δ−1 in lysates from above 150 kDa (Fig. 7C, panel 1, lane 1) to about 125 kDa (Fig. 7C, panel 1, lane 2). Since Mint2 without Tunicamycin reduced the MW of α2δ−1 to approximately 140 kDa, these data suggest reduced but not completely eliminated N-glycosylation of α2δ−1 (Fig. 7C, panel 1, lane 3).

Finally, we examined whether CASK acts similarly to Mint2 (Fig. 7D). α1B successfully precipitates CASK as expected, but surprisingly, it does not precipitate Nrxn1α (Fig. 7D, panel 2, lane 5). Additionally, CASK does not reduce α2δ−1 glycosylation (Fig. 7D, panel 1, lane 5). In contrast, Mint2 prevents complete N-glycosylation of α2δ−1 only when Nrxn1α is present (Fig. 7D, panel 1, lanes 6 & 7). Since the C-terminal regions of Nrxn1α that bind Mint2 and CASK are identical to those in Nrxn1β, we repeated the GFP-α1B trap with Nrxn1β (Fig. 7E). As expected, Mint2 connected α1B to Nrxn1β (Fig. 7E, panel 2, lanes 8–9) and reduced N-glycosylation of α2δ−1 only when Nrxn1β was present (Fig. 7E, panel 1, lanes 8 & 9 compared to lane 10). Similarly, CASK linked α1B to Nrxn1β (Fig. 7E, panel 2, lane 7), but CASK did not decrease glycosylation of α2δ−1. Experiments with Mint1 showed similar effects, effectively decreasing N-glycosylation of α2δ−1 (Supplementary Fig. S6). These results demonstrate that Nrxn1β can bind to Ca_V_2.2 indirectly with equal efficiency through CASK, Mint1/2, or both (Fig. 7E, panel 2, lane 9). Conversely, Nrxn1α binds only through Mint2 (Fig. 7 F). Additionally, we found that Mints, together with Nrxn1α or Nrxn1β, have a new role in disrupting N-glycosylation of α2δ, thus promoting complex formation (Fig. 7G).

## Discussion

Here, we used recombinant α1, β, and α2δ subunits of Ca_V_2.1 (P/Q-type) and Ca_V_2.2 (N-type) VGCCs, along with the synaptic organizer molecules Nrxn1α and Nrxn1β, and the intracellular scaffold proteins CASK and Mint, to assess their ability to form physical complexes. We found that α2δ−1, compared with α2δ−3, preferentially binds to α1A, the pore-forming subunit of Ca_V_2.1. We observed that the extracellular domains of Nrxn1α, but not Nrxn1β, bind to the α2δ−1 and α2δ−3 auxiliary subunits. Moreover, neither Nrxn can directly interact with α1A or α1B pore-forming subunits. Therefore, α2δ subunits bind exclusively to either α1 pore-forming subunits or to Nrxn1α and Nrxn2α (Supplementary Fig. S7) and do not form shared complexes. These results exclude α2δ subunits as the physical link in a large VGCC/Nrxn complex, indicating that they instead toggle between α1 pore-forming subunits and Nrxn1α. Instead, we established that Mint1/2 is a stable mediator of an indirect intracellular association between α1 subunits and Nrxn1α or Nrxn1β, independent of α2δ. At the same time, Mints serve as enhancers of Nrxn1α/α2δ complex formation by inhibiting the full glycosylation of α2δ subunits. Additionally, CASK can associate Nrxn1β, but not Nrxn1α, with α1 pore-forming subunits without affecting the glycosylation of α2δ subunits. Important questions arise from our findings:

First, is the use of recombinant proteins expressed in heterologous cells suitable for studying complexes between neuronal proteins? For Nrxn and VGCCs, which are expressed in multiple isoforms and show overlapping patterns in neurons, a bottom-up approach is the best way to fully understand their molecular interactions. Overexpressing recombinant proteins in HEK293 cells simplifies the study of specific Ca^2+^ channel complexes and enables sensitive biochemical and functional assays. In our study, this method allowed us to analyze competitive binding (Figs. 1C−E and 2), assess isoform-specific differences (Fig. 4B-C), map binding epitopes (Fig. 4D-E), and examine the effects of additional factors like glycosylation and scaffold molecules (Figs. 5, 6 and 7). Conversely, even the most comprehensive study based on brain lysates using standard immunoprecipitation (IP) with numerous antibodies against Ca_V_2.x α1 and β core subunits and mass spectrometry (LC-MS/MS) failed to co-precipitate any α2δ proteins at ratios relevant to the core subunit^54^. In that same study, only tiny amounts (less than 0.05% of the respective α1 reference) of CASK or Nrxn1α, and no Mints at all, were retrieved among the 200 proteins in the nano-environment of the Ca_V_2 channels^54^, illustrating the limitations of IP-based approaches for analyzing specific complexes as we aimed to do in our study. There are additional potential pitfalls associated with standard IP methods for studying α2δ proteins: (i) α2δ−3 often interacts with the Fc fragments of Fc-fusion proteins, as we previously observed when testing a commonly used Nrxn1α-Fc construct alongside unfused Fc control^19^. (ii) α2δ−1 frequently binds to protein A–beads used in antibody-based IPs^94^ because we observed that a pre-clear step to remove endogenous antibodies resulted in the loss of α2δ−1 from the lysate (Supplementary Fig. S8, lane 2). To address these technical challenges, we established a magnetic nanobody-based precipitation (“trap”) method for proteins co-expressed in HEK293 cells. This technique enabled us to test specific complexes by targeting epitope-tagged α1, α2δ, or Nrxn variants with nanobodies and to analyze their binding preferences using non-tagged bait variants as negative controls.

Second, what is the binding epitope in Nrxn1α/α2δ complexes? We found that Nrxn1α binds to both α2δ1 (Fig. 5E) and α2δ3 (Fig. 5F) with similar low binding capacities, consistent with the transient interactions we observed for α2δ in single-particle tracking experiments on the surfaces of cultured HEK293 cells and primary hippocampal neurons^19^^[,95^. Accordingly, the weak interactions are mediated by a broad extracellular region with little sequence specificity on Nrxn1α because both α2δ proteins bind to the extracellular domains LNS1, LNS2 and LNS3, and α2δ−1 additionally binds to LNS5 (Fig. 4). This differs from a previous study that identified LNS1 and LNS5 of Nrxn1α as interaction sites for α2δ−3 using immunoprecipitation^20^. Although binding to LNS4 remains untested, we expect it will not alter the overall conclusion given the observed redundancy in LNS domain binding. We also found that both α2δ and Nrxn1α need to be secreted together, with at least Nrxn anchored to the membrane, suggesting they interact in *cis* (Fig. 6), consistent with our earlier analysis of their functional interaction^19^. Other reports have shown different spatial arrangements between Nrxn1α and α2δ, such as in *trans* or as secreted molecules^20,96^, which we could not confirm in our recombinant system. Our results, which show that α2δ−1 and α2δ−3 binding to Nrxn1α is equally weak (Fig. 5, C-D lane 8) and requires membrane-bound Nrxn1α (Fig. 6C-D), have implications for the role of ectodomain shedding as a regulatory signaling system. Nrxns ectodomain is cleaved by transmembrane proteases ADAM10^22^, ADAM17^22^ and γ-secretase^97^ and might still be bound to Nlgn at synapses^98^. Consequently, a soluble Nrxn1α ectodomain will not adhere to α2δ, but is expected to influence postsynaptic receptors and modulate postsynaptic signaling; however, there persists a potential retrograde signaling pathway of the cleaved soluble cytosolic domain of Nrxns^99^. Additionally, we found that glycosylation of Nrxn1α and α2δ regulates their interaction because removing O-glycans from the stalk region of Nrxn1α increases binding (Fig. 5A-F). The observation that α2δ−3 has fewer N-glycosylation sites than α2δ−1 (Fig. 5G) and binds more effectively to non-glycosylated Nrxn1α (Fig. 5A-F) further suggests that the sugar moieties attached to both proteins might physically block each other. This may also explain why α2δ−1 uses an additional interaction site on the LNS5 of Nrxn1α to counteract its higher overall N-glycosylation. Moreover, Nrxn1α binding does not depend on an intact MIDAS region in α2δ because a mutant α2δ construct^16,63^ that cannot bind Ca_V_2.1 (Fig. 1J) can still bind Nrxn1α (Supplementary Fig. S3). Therefore, although the interaction sites are not identical, α1 core subunits and Nrxn1α compete for binding to α2δ. As a result, co-expressed Nrxn1α reduces the formation of complexes such as α1A/α2δ−3 (Fig. 2C), α1B/α2δ−1 (Fig. 2D), and α1B/α2δ−3 (Fig. 2E). However, the α1A/α2δ−1 complex remains mostly unaffected by Nrxn1α (Fig. 2A) because α2δ−1 binds to α1A three times more strongly than α2δ−3, although both bind equally well to α1B (Fig. 1F). These clear binding preferences explain the dominant effect of overexpressed Ca_V_2.1 with α2δ−1, which displaces endogenous Ca_V_2.2^100^. The observation that overexpressing Ca_V_2.1 with α2δ−3 protects Ca_V_2.2 can now be explained by the higher binding preference of α2δ−3 for α1Β compared to α1A. Similarly, recent studies on how α2δ−1 and α2δ−3 differentially influence Ca_V_2.1 and Ca_V_2.2, using α2δ overexpression or deletion, demonstrate a preferential association of Ca_V_2.1 with α2δ−1 and Ca_V_2.2 with α2δ−3^101^. At specialized mature synapses, presynaptic α1A’s preference for α2δ−1 subunits may be disrupted by unidentified factors that lead to a transition to α2δ−3, enhancing the surface expression of Ca_V_2.1 and altering α1A gating characteristics in auditory spiral ganglion neurons^102^.

Third, how do Nrxns and Mints influence the posttranslational processing of α2δ subunits? Since Mints together with Nrxn1α or Nrxn1β prevent the complete N-glycosylation of α2δ (Fig. 7), they likely interact at the level of the Golgi apparatus. This is an unexpected scenario because Nrxn1α binds extracellularly to α2δ, but not directly to α1 subunits, and Mints bind via their PDZ1 and PDZ2 domains to the cytosolic C-termini of both Nrxn variants and α1 subunits^42,90^, but not to the GPI-anchored α2δ proteins lacking a cytosolic part^87^. It could be that Mint1/2 inhibits full N-glycosylation by bringing Nrxn and α2δ into close contact via its interaction with α1 subunits, but it remains unclear how exactly Mint1/2 manages this arrangement during trafficking through the secretory pathway. Alternatively, an as-yet-unidentified transmembrane protein directly links Nrxns, Mint1/2, and α2δ subunits. For example, presenilin has a non-proteolytic scaffolding function^103^. It binds both Mints^104^ and Nrxns^97^, but whether presenilin also binds to α2δ subunits remains unknown. In support of this, the transmembrane protease ADAM17 is part of the Mint-binding presenilin/γ-secretase complex. It cleaves Nrxns within the cysteine loop of the extracellular stalk region^105^, thereby retaining the full-length structure of Nrxns via a cysteine bridge connecting the two chains. Similarly, ADAM17 processes α2δ into α_2_ and δ chains in the Golgi compartment, thereby maintaining the whole structure through interchain disulfide bonds^92^. Thus, Nrxns, Mints, and α2δ might form complexes during these maturation steps, thereby preventing complete N-glycosylation of α2δ by rendering glycosylation sites inaccessible. Interestingly, the heparan sulfate conjugation of Nrxn, which likely occurs at the cis-to-medial Golgi compartmen^106^, does not influence Nrxn1α/α2δ complex formation (Fig. 5C- D).

Fourth, how does the binding of Nrxn1α to α2δ subunits influence neurotransmission? Our current data show that Nrxn1α does not directly interact with α1 pore-forming subunits but binds equally well to both α2δ−1 and α2δ−3. Therefore, the initial idea that α2δ−1 recruits Nrxn1α into the synaptic VGCC complex, leading to increased Ca^2+^ influx^19^, and that Nrxn1α has the opposite effect on α2δ−3 by pulling the subunit away from the VGCC core^20^ was overly simplistic. In fact, only 10% of α2δ is bound to α1 pore-forming subunits^54^, which aligns well with VGCC-independent roles of α2δ subunits^16,107,108^ and their high surface mobility^95^. In addition, our observation of a higher binding preference of α2δ−1 to α1Α (Fig. 1) explains earlier findings that the surface mobility of α2δ−3 is higher than that of α2δ−1 and that the α1 pore-forming subunits have the lowest mobility^19,95,109^. These and other findings are best explained by a non-static “pinball”-model of weak associations in which α2δ subunits move like a ball among various VGCC core subunits (pins) and Nrxn1α in the active zone. Nrxn1α has the highest surface mobility and acts as a paddle, keeping α2δ in close proximity to the α1 core to ensure effective function. As a consequence of stronger binding of α2δ−1 to α1Α and competitive binding to α1B (Fig. 1E), there is more mobile, unbound α2δ−3 than α2δ−1, consistent with its higher surface mobility. In the absence of the Nrxn framework, such as in α-Nrxn triple-knockout neurons, overexpressed α2δ−1 subunits are less confined on average, resulting in higher surface mobility^19^. Overexpressing α2δ−3, in turn, can displace endogenous α2δ−1 from α1Β or engage in α1-independent associations with Munc13-1^110^, or Rim^111^, and thus appear more confined^19^. As a benefit of the redundancy of binding LNS domains (Fig. 4D-E), a single SNP in human Nrxn1α might not be sufficient to alter this system. However, this will be answered once the complex structure of Nrxn1α and α2δ is solved.

Fifth, what is the role of the intracellular link between Nrxn and α1 pore-forming subunits via scaffolding molecules? As proposed earlie^44^, we here validate that Mints can bridge Nrxn1α and Nrxn1β to the C-terminus of Ca_V_ α1 subunits (Fig. 7 D-E). In addition, CASK can connect Nrxn1β, but not Nrxn1α, to Ca_V_ α1 subunits (Fig. 7D-E), a surprising discrepancy, as the C-termini of Nrxn1α and Nrxn1β are identical. We therefore suggest that the different sizes of the extracellular domains of Nrxn1α and Nrxn1β likely determine the selective recruitment of CASK to Nrxn1β. It has been shown that extracellular Neuroligin induces an ordered, dense layer of Nrxn1β in HEK293 cells, whereas Nrxn1α remains disperse^112^. Similarly, CASK can form dense oligomers with Nrxn1β^45^, in which CASK adopts a PALS conformation that uses its PDZ and SH3 domains, which are required for binding to Ca_V_ α1^44^. In our pulldown experiments with nanobodies against α1 subunits, we found α1B/CASK/Nrxn1β complexes, where Nrxn1β presumably binds to the CASK PDZ domain^113^ and its SH3 domain to α1^53^. The SH3 binding sites (PxxP) are closer to the α1B pore than the C-terminal Mint-binding PDZ motif. This proximity may only allow the smaller Nrxn1β to integrate into VGCC complexes via CASK. Although many active zone molecules have been assigned specific roles, it remains unclear whether and how CASK and Mint regulate neurotransmission. Consistent with the finding that CASK, together with Mint and Stxbp1, regulates insulin secretion^114^, we observed a reduced number of dense-core vesicles (DCVs) in β-Nrxn-deficient neurons^115^, suggesting that β-Nrxns/CASK/Ca_V_ complexes may be involved in DCV exocytosis. Neuronal Mint proteins are primarily localized to endosomes and the Golgi apparatus and have been shown to affect axonal transport of Nrxn^42^. Our finding that Mint and Nrxn change N-glycosylation of α2δ implicates a Golgi-associated regulatory role for Mints. Since Nrxn is required to bring a CASK/Liprin complex to the synaptic surface^116^, Mint can join that complex^116^ and connect with Stxbp1^117^, Rim-binding protein, and Rim as part of the multi-protein Ca_V_ complex^118^. Nrxns, both on their own and by binding to Leukocyte common antigen-related receptor protein tyrosine phosphatases (LAR-RPTPs), have been proposed as major presynaptic adhesion proteins to maintain bidirectional signaling within nanocolumns across the synaptic cleft^119^, and CASK and Mint could be involved in linking Nrxn to the release machinery.

### Methods

#### Animals

Brains of adult wildtype mice of either sex (C57BL/6J, Jackson Labs; RRID: IMSR_JAX:000664) were used for lysis and immunoprecipitation. Mice were maintained at the central animal facility in Münster under standard housing conditions with food and water available ad libitum on a 12 h light/dark cycle. Adult animals of about 30 g were anesthetized by intraperitoneal injection of a mixture of 3.0 mg ketamine (#K1884, Sigma-Aldrich; RRID: SCR_008988) and 0.3 mg xylazine (X1251, Sigma-Aldrich; RRID: SCR_008988), decapitated, and their brains were quickly removed on ice. All experiments were performed at the University of Münster following government regulations for animal welfare and approved by the Landesamt für Natur, Umwelt und Verbraucherschutz (LANUV, Düsseldorf, NRW, Germany), license numbers 81-02.04.2019.A119 and 81-02.04.2021.A216.

### Cell culture

HEK293tsA.201 cells (HEK293, #96121229, ECACC; RRID: CVCL_2737) were grown in complete Dulbecco’s modified eagle medium (DMEM; #D5769, Sigma Aldrich) supplemented with 10% fetal bovine serum (#FBS-12 A, Capricorn) and 1% Penicillin/Streptomycin (#15070-063, ThermoFisher) at 37 °C in a humidified 5% CO_2_ atmosphere (CO_2_ Incubator, Sanyo). Cells were passaged at 85–90% confluency every 3–4 days. FreeStyle 293-F cells (293-F, # R79007, Invitrogen; RRID: CVCL_D603) were cultured in FreeStyle 293 Expression Medium (#12338-018, Gibco) at 37 °C in a humidified 7% CO_2_ atmosphere under constant shaking (120 rpm). Cells were maintained in 125 ml Erlenmeyer flasks (#781011, NEST) and, if their viability exceeded 70%, seeded at 12*10^6^ cells in 40 ml every 3-–4 days.

### Antibodies

Commercially available antibodies for immunoblotting: Rabbit *anti-*Ca_V_2.1 (SynapticSystems 152–203; 1:1,000; RRID: AB_2619841), rabbit *anti-*Ca_V_2.2 (SynapticSystems 152–313; 1:1,000; RRID: AB_2619846), mouse *anti-*α2δ−1 (SantaCruz sc-271697; 1:1,000; RRID: AB_10708582), rabbit *anti-*α2δ−3 (Thermofisher Scientific #PA5-87802; 1:1,000; RRID: AB_2804420), rabbit *anti-*β3 (AlomoneLabs ACC-008; 1:10,000; RRID: AB_2039787), mouse *anti-*HA (BioLegend 901501; 1:1,000; RRID: AB_2565006), mouse *anti-*SPOT (ChromoTek 28a5; 1:1,000; RRID: AB_2892245), rabbit *anti-*GFP (Abcam ab290; 1:10,000; RRID: AB_303395), mouse *anti-*Actin (SantaCruz SC-56459; 1:1,000; RRID: AB_830981), rabbit *anti-*Nrxn1/2/3 (SynapticSystems 175-003; 1:1,000; RRID: AB_10697815), rabbit *anti-*Mint2 (Merck M-3319; 1:3,000; RRID: AB_477178) and mouse *anti-*CASK (BioLegend 830601; 1:5,000; RRID: AB_2564940). HRP-conjugated secondary antibodies: goat *anti-*rabbit IgG (H + L)-HRP conjugate (Bio-Rad Laboratories #170–6515; 1:15,000; RRID: AB_11125142) and goat *anti-*mouse IgG (H + L)-HRP conjugate (Bio-Rad Laboratories #170–6516; 1:15,000; RRID: AB_11125147).

Purification of mouse *anti-*β_4_ antibody: A 125 ml Erlenmeyer flask was supplied with 30*10^6^ 293-F cells in 30 ml, and cells were transfected according to the manufacturer’s instructions. Briefly, 37.5 µg plasmid DNA were diluted in a total volume of 600 µl OptiPRO SFM (#12309-019, Gibco) and mixed with another tube containing 37.5 µl FreeStyle MAX Reagent (#16447-100, Gibco) diluted in 600 µl OptiPro SFM. The mixture was incubated for 10 min and added to the cells drop-wise. One week after transfection, the medium was collected, and 100 µl equilibrated Protein A sepharose (#17513801, Cytiva) was added and rotated (12 rpm) at 4 °C overnight. The beads were washed with 50 mM Tris (pH 7.5; #4855.2, Roth) and 150 mM NaCl (#3957.1, Roth) three times (10 min each), followed by acidic elution in 100 mM glycine (pH 2.7; #3908.2, Roth) and neutralization with 1 M Tris (pH 8). Using multiple centrifugal filter steps (#88513, ThermoFisher), the buffer was exchanged to PBS. Protein concentration was measured by 280 nm absorption (BioSpectrometer, Eppendorf) using an extinction coefficient of 0.630957 for IgG (ProtParam). Protein was diluted to 1 mg ml^− 1^, stored at 4 °C, and used at 1:1,000. The specificity of the antibody was validated in HEK293tsA.201 cells expressing either β3 or β4.

Purification of sfNG1-9-ALFA-LgBit: HEK293-F cells were transfected and processed as described above. sfNG1-9-ALFA-LgBit-pFc (for plasmid construction, see section below) bound by protein A beads were eluted by cleavage from Fc-tag with HRV 3 C protease. Shortly, beads were resuspended in 50 mM Tris pH7.4, 150 mM NaCl, and HRV 3 C protease (#71403, Merck) was added according to the manufacturer’s instructions. The following day, Protein A beads were removed by centrifugation at 11,000 x g for 30 s. NaCl concentration was increased to 300 mM and at 4 °C for 30 min Ni-NTA beads removed 3 C protease from supernatant. Following centrifugation (11,000 x g for 30 s), the supernatant was concentrated to yield 400 µg ml^− 1^ ALFA-sfNG1-9.

### Plasmids

pCMV5-EGFP-Nrxn1α, pCMV-EGFP-Nrxn1αΔ54, and pCMV-EGFP-Nrxn1β have been described before^28^. All Nrxn stalk domain substitution constructs were derivates of pCMV5-EGFP-Nrxn1α. The stalk domain of pCMV-L13^120^ was deleted by PCR using primer MM23-03: GAG GTC AAT TGA GGC ACT TCA CCG ACC AG and MM23-04: CTA AGT CAC GTC GGC ATG GTG GTG GGG ATT introducing sites for MfeI and BmgBI restriction digestion. Neuroligin1 stalk domain was PCR amplified from full-length Nlgn1 cloned in pCMV5 vector using the primer MM23-01: GCC TCA ATT GAC CTC GAC AAC AAC TAA AGT GCC and MM23-02: CAT GCC GAC GTG ACT TAG CTC TGT GGA GTA GTC CC TTT, CD8 stalk domain was amplified from pCMV5-CD8 using primers MM23-09: GCC TCA ATT GCC AGC GAA GCC CAC CAC G and MM23-10: CAT GCC GAC GTG ATC ACA GGC GAA GTC CAG CC, Nrxn1αΔHSΔCysA stalk domain was amplified from pCMV-L13ΔHSΔCysA using primers MM23-05: GCC TCA ATT GGG ACC AGG AGG ACC ACC ACC AGG AGG ACC AGG ACA CGT CGG G and MM23-06: CAT GCC GAC GTG AGT GGT ACT GCT AGA CTC CCG, synthetic PG-linker was created by self-annealing primers MM23-07: AAT TGG GAC CAG GAG GAC CAC CAC CAG GAG GAC CAG GAC AC and MM23-08: GTG TCC TGG TCC TCC TGG TGG TGG TCC TCC TGG TCC. Amplified stalk domains were cloned into pCMV-L13-NoStalk via MfeI and BmgBI sites and, subsequently, cloned into pCMV5-EGFP-Nrxn1α via SbfI and XmaI for final constructs. Untagged pCMV5-Nrxn1α was generated by NheI digest of pCMV5-EGFP-Nrxn1α to remove the EGFP-cassette and subsequent religation. Secreted HA-Nrxn1α-stalk-ECD was cloned by digesting pCAG-Nlgn1SP-HA-Nrxn1α-TEC-Fc^121^ with EcoRI and HindIII, blunting overhangs by refill PCR and blunt-end ligation. Full-length wildtype pCAG-Nlgn1SP-HA-Nrxn1α(-SS4) was a gift from Peter Scheiffele (Addgene_58266).

To generate constructs for triple split superfolderNeongreen (sfNG), sequences encoding for sfNG1-9, sfNG10, and HiBiT-sfNG11 were purchased from Biomatik (Ontario, Canada) in pBSK Vector. For pbA-sfNG10-α2δ−1, sequences were cloned into pbA-SPOT-α2δ−1 (gift from Martin Heine, University of Mainz) via AscI and BglII to replace the SPOT sequence. Similarly, sfNG10-α2δ−3 was cloned into pbA-SPOT-α2δ−3 (gift from Martin Heine, University of Mainz) via NotI and BsrGI restriction enzymes. The HiBiT-sfNG11-Nrxn1α fragment was cloned into pCMV-L2^120^ via HindIII and SacII. Finally, LgBiT domain was amplified from pcDNA3.1-LgBit (Addgene_162588; discontinued) with primers MM22-83: CAA TGC TCG A-GG GTT CCG GTG GCG GCA GTG GTG GTG TCT TCA CAC TCG AAG ATT TCG T and MM22-84: GTA CCT CGA GGC TAG CGC TGT TGA TGG TTA CTC GGA A, and the PCR product cloned into pCMV-Nrxn1αSP-sfNG1-9-pFc via XhoI to generate pCMV-sfNG1-9-LgBit-pFc. To introduce a N-terminal ALFA-tag, primers MM23-108: TCG ACA AGC GGC GGG AGC CGT CTC GAG GAG GAG CTG AGA AGA AGA CTG ACC GAA GGC GGC AGC AT and MM23-109: CGA TGC TGC CGC CTT CGG TCA GTC TTC TTC TCA GCT CCT CCT CGA GAC GGC TCC CGC CGC TTG, were self-annealed and cloned into pCMV-sfNG1-9-LgBit-pFc via SalI and ClaI to generate pCMV-sfNG1-9-ALFA-LgBit-pFc.

Constructs expressing N-terminally GFP-tagged Ca_V_2.1^122^, Ca_V_2.2^123^ and auxiliary subunits β3^124^, HA-α2δ−1 and HA-α2δ−3 were described before^101^. The MIDAS sequence DVSGS of HA-α2δ−3 was mutated to AVAGA using QuikChange (#200515-5, Agilent Technologies) with primers MM14-23: GGA TGT TGT CAT TTT GGT GGC CGT CGC TGG CGC CAT GAA AGG ACT CCG CTT G and MM14-24: CAA GCG GAG TCC TTT CAT GGC GCC AGC GAC GGC CAC CAA AAT GAC AAC ATC C. For SPOT-tagged extracellular domains (ECDs), two stop codons were placed after the omega site^125^ in the plasmids of SPOT-α2δ−1 and SPOT-α2δ−3 via NEB Q5 mutagenesis; SPOT-α2δ−1 with MM21-03: TGA TGA GTT TCT GGT TTA AAC CCT TCC and MM21-06: ACC ACC ACA GTC GGT ATA ATC CTC CAG, and SPOT-α2δ−3 with MM21-04: ACC CCC ACA CTC TCT TGC and MM21-05: TGA TGA GCC TCG AGT CTT CAG GCC CAG G. Neuronal cell adhesion molecule (NCAM)-sequence was purchased from Biomatik (Canada) in pBSK and cloned into HA-α2δ−1 and HA-α2δ−3 via NheI and Bsu36I to generate HA-α2δ−1-GPI and HA-α2δ−3-GPI. CASK, Mint2, and Mint2ΔPDZ were cloned into pCMV5 plasmid. pmEGFP-C1 (Addgene_36412) and pcAG-HA-Nrxn1α-LNS1, pcAG-HA-Nrxn1α-LNS2, pcAG-HA-Nrxn1α-LNS3, pcAG-HA-Nrxn1α-LNS5 (Addgene_128105, _128106, _128107, _128109). HA-LNS6(-SS4) and HA-LNS6(+ SS4) were described previously^38^. All restriction enzymes were purchased from New England Biolabs and used according to the manufacturer’s instructions. Plasmids were verified by sequencing (Eurofins Genomics, Germany).

### Immunoprecipitation by trap assays

HEK293 cells were seeded at a density of 1.4*10^6^ in 10 cm plates (#83.3902, Sarstedt). The following day, cells were transfected with a total of 16 µg DNA. Additional proteins, such as Mint2 and CASK, were added in equal ratios. Twenty-four hours after transfection, cells were provided with fresh DMEM, grown for another 48 h, and harvested. Cell pellets were resuspended in 1 ml HYP Buffer (20 mM HEPES pH 7.5, 2 mM CaCl_2_, Proteinase Inhibitor Cocktail IV (PIC IV, 1:1,000; #ab201118, Abcam), incubated on ice for 15 min, homogenized with a dounce glass tissue grinder and centrifuged at 300 x g and 4 °C for 5 min. One volume of DL buffer (20 mM HEPES, 2 mM CaCl_2_, 600 mM NaCl, 1% Digitonin, PIC IV 1:1,000) was added to the supernatant, the mixture sonicated in a water bath for 30 s and rotated (12 rpm) at 4 °C for 1 h. After the addition of 1 volume IP buffer (20 mM HEPES, 2 mM CaCl_2_, 300 mM NaCl, PIC IV 1:1,000), a final centrifugation step was performed at 18,000 x g and 4 °C for 20 min. The supernatant was collected, one aliquot was taken off as lysate, and the remainder was subjected to magnetic agarose-conjugated *anti-*GFP (#gtma, Proteintech), *anti-*SPOT- (#etma, Proteintech), or *anti-*ALFA-tag (#N1505, NanoTag) nanobody beads according to manufacturer’s instructions. Briefly, the “Trap” started, when 20 µl of equilibrated *anti-*SPOT, *anti-*GFP, or *anti-*ALFA nanobodies were added to supernatant and rotated (12 rpm) at 4 °C overnight. The beads were washed (20 mM HEPES, 2 mM CaCl_2_, 150 mM NaCl) three times (5 min each), resuspended in Sample Buffer (0.0625 M Tris pH 6.8, 10% glycerol, 2.3% SDS, 2.5% β-mercaptoethanol, 50 mM DTT), and boiled for 10 min.

### Western blot analysis

Protein samples were separated by SDS-PAGE (4–15% Q-Page™ TGN gels; #QP4510, SmoBio) and transferred to nitrocellulose (#1704158, Bio-Rad). The membrane was blocked with 5% normal goat serum (NGS; GOA-1 A, Capricorn) in 5% milk (#T145.2, Roth) in PBS-T (PBS with 0.05% Tween; #9127.1, Roth) and incubated with primary antibody at 4 °C overnight. The following day, the membrane was washed with PBS-T three times (10 min each) and incubated with horseradish peroxidase (HRP)-conjugated secondary antibodies at RT for 1 h. The membrane was washed with PBS-T as before, and HRP activity was detected using an enhanced chemiluminescent (ECL) substrate (#WBKLS0500, Merck). For subsequent detection of additional antibodies, the membranes were washed with PBS-T (15 min), blocked as before, and incubated again with primary antibodies at 4 °C overnight. The following day, sample detection was carried out with HRP-conjugated antibodies as described above. This procedure was repeated until all proteins of interest were analyzed. Protein bands were visualized on an ECL ChemoStar (Intas) with ChemoStar Imager v.6.0.9 and, where applicable, subjected to densitometric quantification via ImageJ (NIH) software, and normalized to either an internal standard or to signals of proteins of interest in the lysate.

### Fluorescence microscopy

HEK293 cells were seeded on 13 mm glass coverslips (#41001113, Assistent) coated with 0.1 mg ml^− 1^ poly-L-lysine (#P-2636, Sigma) at a density of 4*10^5^ cells/plate in 6 cm plates (#83.3901, Sarstedt). The following day, cells were transfected with 4 µg of cDNA encoding α1:β3:α2δ:Nrxn1α in a 2:1:1:2 ratio via the calcium phosphate method^126^. After 24 h, cells were provided with fresh DMEM and grown for another 24 h. Cells were labeled with 1 µg ml^− 1^ SPOT-label AlexaFluor 568 (#ebAF568, Proteintech) in DMEM for 1 h. Cells were washed with PBS (#PBS-1 A-P19, Capricorn) and fixed with 4% paraformaldehyde (PFA) for 15 min. Following three PBS washes, cells were permeabilized with 0.3% Triton X-100 (#3051.3, Roth) in PBS for 30 min. Before antibody staining, cells were blocked with 5% NGS (#GOA-1 A, Capricorn) in PBS at room temperature (RT) for 1 h. Next, cells were incubated with primary *anti-*Nrxn123 or *anti-*β3 at 4 °C overnight. Following PBS washes, secondary anti-rabbit AlexaFluor 568 (#A-11035, Invitrogen, RRID: AB_2534093) or anti-rabbit AlexaFluor647 (#21245, Invitrogen, RRID: AB_2535813) in 5% NGS in PBS was added for 1 h. Afterward, cell nuclei were stained with Hoechst 33,342 (#H3570, Invitrogen) for 15 min, and coverslips were washed in PBS and mounted onto a clean glass slide using fluorescence mounting medium (#S3023, Dako). Samples were analyzed on a spinning disc LSM 5 Duo (Carl Zeiss, Jena) equipped with 100x/1.46 NA oil immersion objective and a Hamamatsu EM-CCD 512 digital camera. Image acquisition was performed using VisiView software (v5.0.0.23) along the Z-axis in 0.7 μm steps, and parameters were held constant across samples. For background corrections, fluorescence intensities of control slides were subtracted from maximum intensity in Z projections or single Z slices.

### Software and statistics

Software used in the presented study includes VisiView (v5.0.0.23), ImageJ (v1.53a), DNAStar SeqBuilder (v17.4.1), DNAStar SeqMan Ultra™ (v17.4.1), Adobe Illustrator (v25.2), Adobe Photoshop (v22.2.0), GraphPad Prism (v10), and PyMOL (3.1, open-source). Web applications: Glycam (glycam.org), NetOGlyc-4.0 (healthtech.dtu.dk), O-GlcNAcPRED-DL (oglcnac.org).

Due to the small overall sample size (*n* < 5) across repeated experiments, non-parametric statistical tests were applied to all quantifications, as specified in the figure legends, and *p* < 0.05 was considered significant. Analyses were performed using GraphPad Prism 10.6.1 (GraphPad Software, San Diego, CA, USA). For all experiments, data were reported as biological replicates pooled from multiple cells (e.g., lysates). No statistical methods were used to predetermine the sample size.

## Supplementary Information

Below is the link to the electronic supplementary material.

Supplementary Material 1

Supplementary Material 2

## Data Availability

All data generated or analyzed during this study are included in this published article and its supplementary information files. Please send requests for materials to M.M. (Markus.Missler@uni-muenster.de).
