# Supplementary material for: Synaptic Ca2+ channels and neurexins are linked through direct and indirect binding complexes
Source: Sci Rep. 2026 Jul 15;16:22268. doi: 10.1038/s41598-026-62077-2 (PMC13373219; doi:10.1038/s41598-026-62077-2)
Supplement: Supplementary file 1 — Supplementary Material 1 [file 41598_2026_62077_MOESM1_ESM.pdf]

Supplementary Information

Synaptic Ca^{2+} channels and neurexins are linked through direct and indirect binding complexes

Authors:

Nils Hohaus[1], Carsten Reissner[1,*], and Markus Missler[1,*]

Affiliations:

[1]Institute of Anatomy and Molecular Neurobiology, University of Münster, Germany

[*]Joint last authors (e-mails: Markus.Missler@uni-muenster.de, Carsten.Reissner@uni-muenster.de)

Supplementary Material:

Supplementary Figure S1:	Competitive binding assay shows α2δ transmembrane domains do not determine α1A preferences for α2δ
Supplementary Figure S2:	β3 subunits do not bind Nrxn1α.
Supplementary Figure S3:	Mutations in α2δ-MIDAS motif do not abolish Nrxn1α binding.
Supplementary Figure S4:	Expression and α2δ binding of isolated HA-LNS domains.
Supplementary Figure S5:	Precipitated α2δ-3 bound to α1B has undergone proteolytic maturation into in α2 and δ chains.
Supplementary Figure S6:	Mint1 and Nrxn1α prevent complete glycosylation of α2δ-1.
Supplementary Figure S7:	Nrxn2α binds to α2δ-1.
Supplementary Figure S8:	Protein A beads precipitate endogenous α2δ-1 from mouse brain lysate.

Supplementary Information – Supplementary Figures

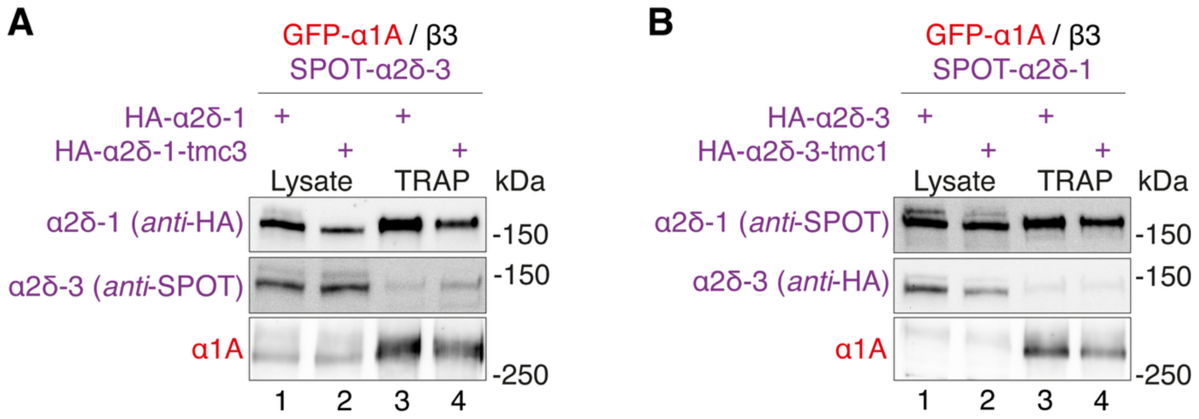

Supplementary Figure S1 – Competitive binding assay shows α2δ transmembrane domains do not determine α1A preferences for α2δ. A HEK293 cells were transfected with GFP-α1A, β3 (not shown), SPOT-α2δ-3, and either HA-α2δ-1 or an α2δ-1 construct where its transmembrane domain and cytosolic tail were replaced by the corresponding sequences of α2δ-3 (HA-α2δ-1-tmc3). GFP-α1A binds HA-α2δ-1-tmc3 better than α2δ-3. **B** Similar to *A*, but with α2δ-3-tmc1 carrying the C–terminus of α2δ-1. Original, uncropped blots are presented in the supplementary data file.

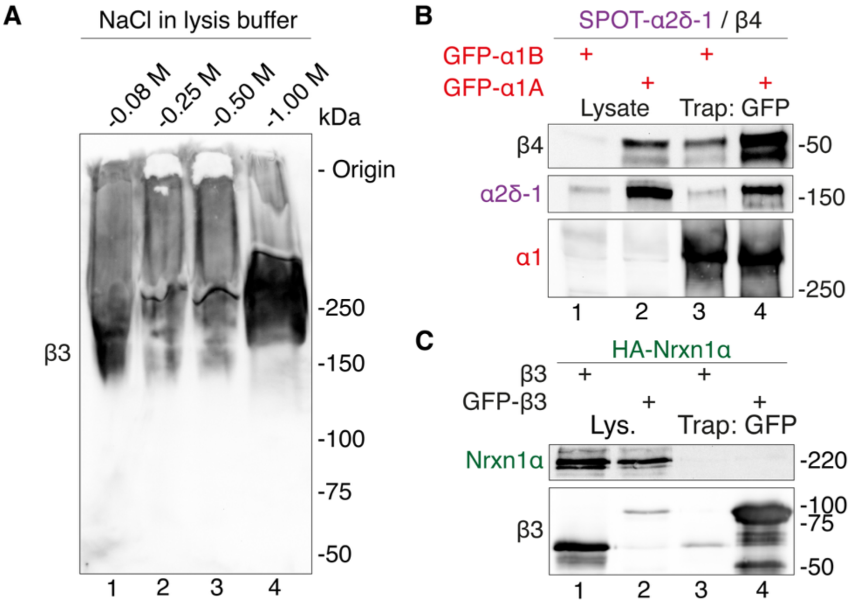

Supplementary Figure S2 – β3 subunits do not bind Nrxn1α. **A** HEK293 cells transfected with β3 subunits, lysed with triton and incubated with increasing concentrations of NaCl. Native PAGE blots labeled with *anti*-β3 show dissociation of β3 oligomers with 1M NaCl. **B** SDS-western blot of GFP-traps prepared from HEK293 cells, α2δ-1 and β4 co-transfected with either GFP-α1B or GFP -α1A. ***C*** HA-Nrxn1α co-transfected with non-labeled β3 or GFP-β3 in HEK293. GFP-trap to investigate the precipitation of Nrxn1α by GFP-β3. Lys.: Lysate. Original, uncropped blots are presented in the supplementary data file.

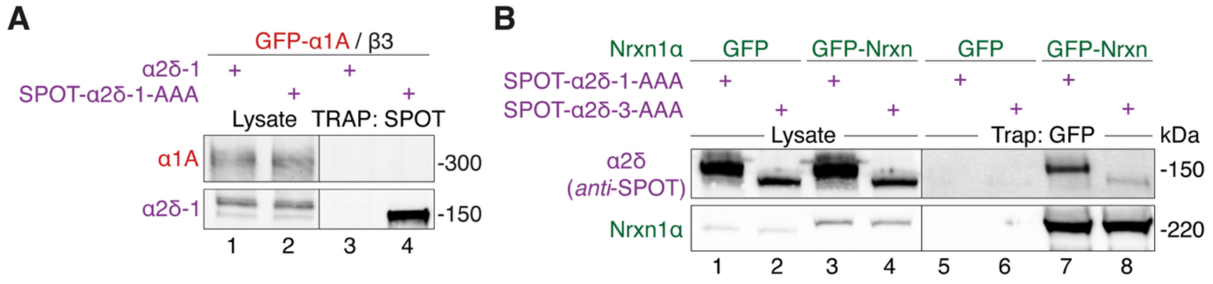

Supplementary Figure S3 – Mutations in α2δ-MIDAS motif do not abolish Nrxn1α binding. A SPOT-Traps of lysate from HEK293 cells transfected with GFP-α1A, β3 (not shown) and either non-tagged wildtype α2δ-1 as negative control or SPOT-α2δ-1-AAA to demonstrate the lack of α2δ-1-AAA binding to α1A subunit. Blots were probed with *anti*-α1A and *anti*-α2δ-1. **B** GFP co-transfected with non-tagged Nrxn1α as control (lane 1-2) or GFP-Nrxn1α (lane 3-4) were transfected together with α2δ-AAA MIDAS mutants SPOT-α2δ-1 or SPOT-α2δ-3 into HEK293 cells, and lysates were subjected to GFP trap to investigate binding of α2δ MIDAS motif mutants to Nrxn1α. Note the difference of α2δ-1 and -3 available in lysate (panel 1-4), corresponding to apparently decreased precipitation of α2δ-3-AAA. Blots were probed with *anti*-SPOT (for α2δ) and *anti*-Nrxn1/2/3. Original, uncropped blots are presented in the supplementary data file.

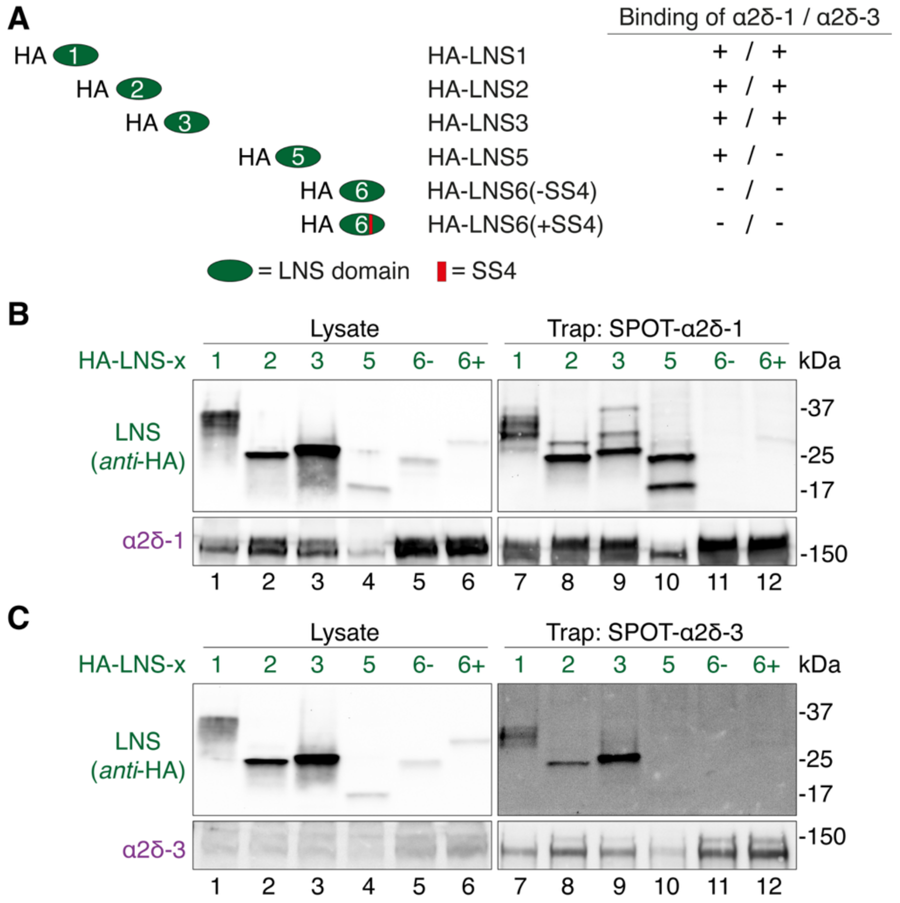

Supplementary Figure S4 – Expression and α2δ binding of isolated HA-LNS domains. **A** Schematic representation of Nrxn1α's HA-LNS domains that were used to test precipitation by SPOT-α2δ-1 (B) or SPOT-α2δ-3 (C) via SPOT trap. Blots were probed with anti-HA (for HA-LNS domains) and anti-SPOT (for α2δs). Original, uncropped blots are presented in the supplementary data file.

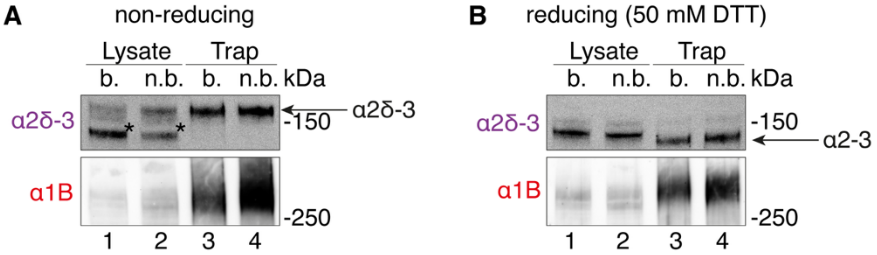

Supplementary Figure S5 – Precipitated α2δ-3 bound to α1B has undergone proteolytic maturation into in α2 and δ chains. GFP-α1B and HA-α2δ-3 were co-expressed in HEK293 cells and precipitated by *anti*-GFP nanobodies. Samples were either boiled at 99°C for 10 min (b.) or incubated at 30°C for 20 min (n.b.). **A** Using non-reducing sample buffer, α2δ-3 shows two bands in lysates (lanes 1-2), but only α2δ-3 with a MW above 150 kD binds to α1B (lanes 3–4). **B** Under reducing conditions, lower bands of α2-3 indicate the separation of α2 and δ chains (B, lanes 3–4, upper panel). Samples are N-glycosylated. Bands marked with asterisk (*) are likely dissociated α2-3 chains comparable to bands seen under reducing conditions (B, upper panel, lanes 1–2). N-terminally HA-tagged α2δ-3 and α2-3 were labeled with *anti*-HA (1:1,000). Original, uncropped blots are presented in the supplementary data file.

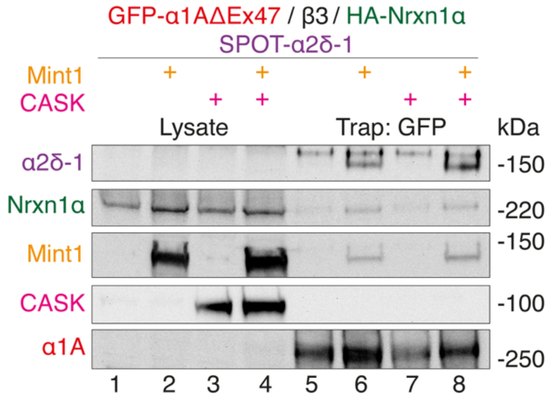

Supplementary Figure S6 – Mint1 and Nrxn1α prevent complete glycosylation of α2δ-1. GFP-trap of GFP-α1A in complex with β3 (not shown) and α2δ-1 (lane 5) precipitates Mint1 and Nrxn1α (lanes 6 & 8), but not CASK (lanes 7 & 8). Lower bands (lanes 6 & 8, 1st panel) indicate reduced N-glycosylation. Note, that Mint1 binds to α1A missing exon 47 (ΔEx47). Original, uncropped blots are presented in the supplementary data file.

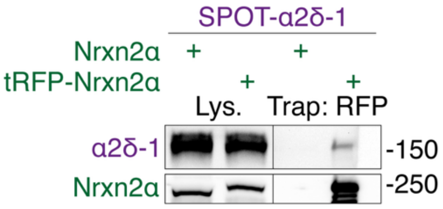

Supplementary Figure S7 – Nrxn2α binds to α2δ-1. Unlabeled Nrxn2α as control (lane 1) or tRFP-Nrxn2α as bait were transfected into HEK293 cells together with SPOT-α2δ-1, and lysates were subjected to RFP trap to investigate precipitation of α2δ-1 by Nrxn2α. Blots were probed with *anti*-α2δ-1 antibody and *anti*-Nrxn1/2/3 (Nrxn2α). Original, uncropped blots are presented in the supplementary data file.

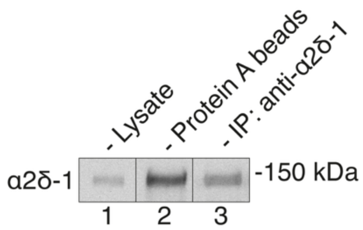

Supplementary Figure S8 – Protein A beads precipitate endogenous α2δ-1 from mouse brain lysate. A single wildtype mouse brain was lysed (lane 1). Endogenous antibodies were pre-cleared from lysate via incubation with untreated Protein A beads (lane 2) and, subsequently, α2δ-1 was precipitated using *anti*-α2δ-1 antibody (SantaCruz, sc-271697) bound to Protein A beads. Probing blots for α2δ-1 revealed bands at about 140 kDa in the lysate and IP (lane 1 and 3, respectively), but crucially the strongest signal was observed with the untreated Protein A beads used in the pre-clear (lane 2). Original, uncropped blots are presented in the supplementary data file.
