# Supplementary material for: Synaptic Ca2+ channels and neurexins are linked through direct and indirect binding complexes
Source: Sci Rep. 2026 Jul 15;16:22268. doi: 10.1038/s41598-026-62077-2 (PMC13373219; doi:10.1038/s41598-026-62077-2)

Supplementary Data

Synaptic Ca^{2+} channels and neurexins are linked through direct and indirect binding complexes

Authors:

Nils Hohaus[1], Carsten Reissner[1,*], and Markus Missler[1,*]

Affiliations:

[1]Institute of Anatomy and Molecular Neurobiology, University of Münster, Germany

[*]Joint last authors (e-mails: Markus.Missler@uni-muenster.de, Carsten.Reissner@uni-muenster.de)

Supplementary Material:

Original, uncropped images of Western blots.

Figure 1C

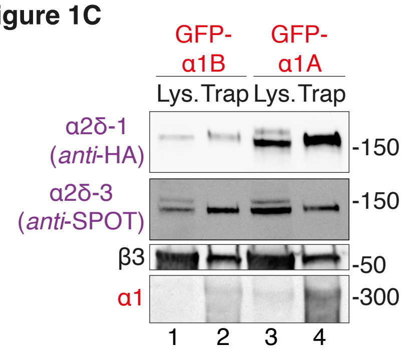

Figure 1C, panel 1, filename: 2022-01-19 12.47.45,5-3.scan_raw.png

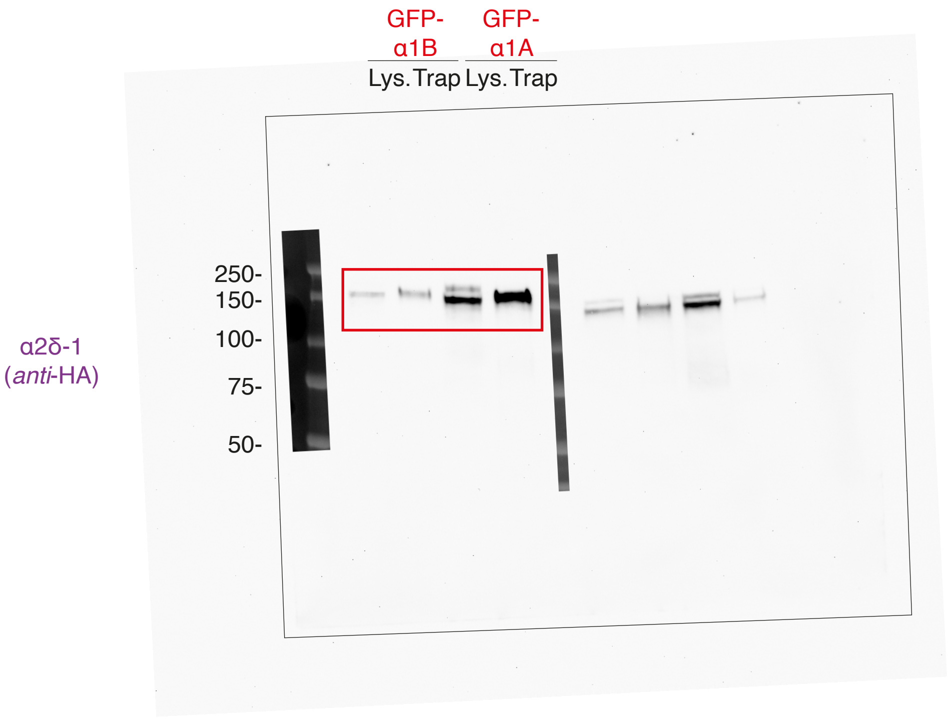

Figure 1C, panel 2, filename: 2022-01-20 11.32.44,5-3.scan_raw.png

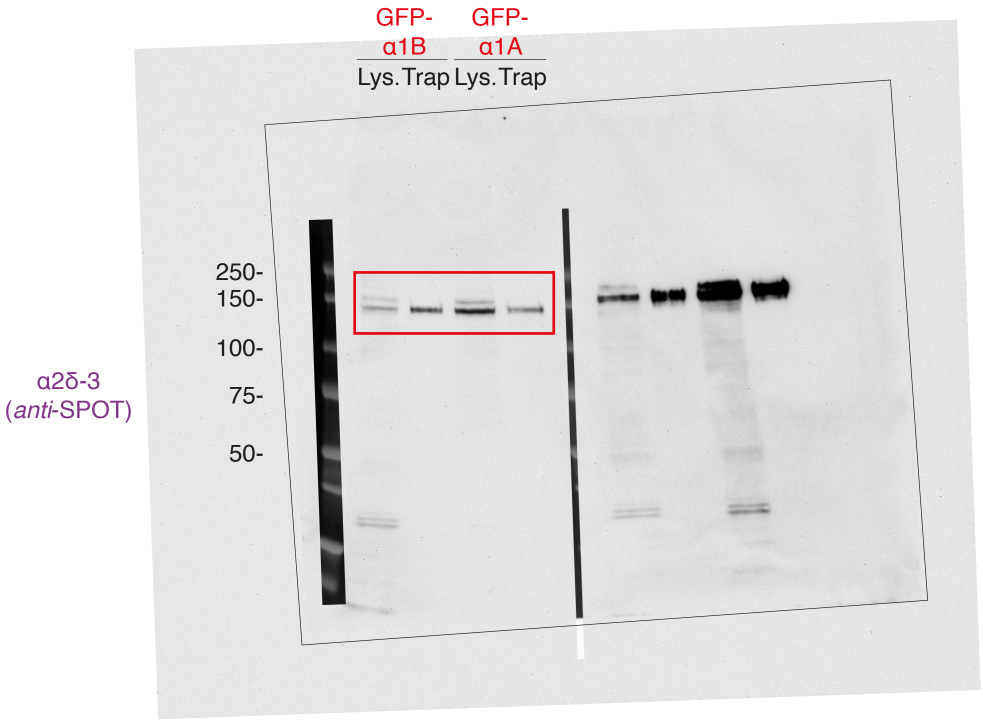

Figure 1C, panel 3, filename: 2022-01-20 11.47.12,1-1.scan_raw.png

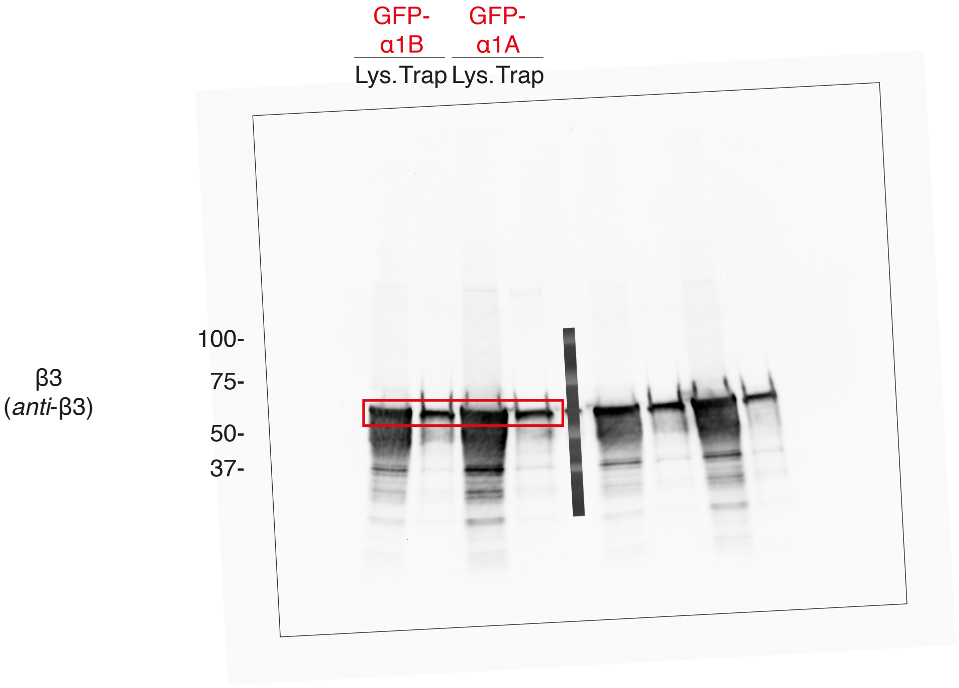

Figure 1C, panel 4, filename: 2022-03-08 11.32.07,3-2.scan_raw Kopie.png

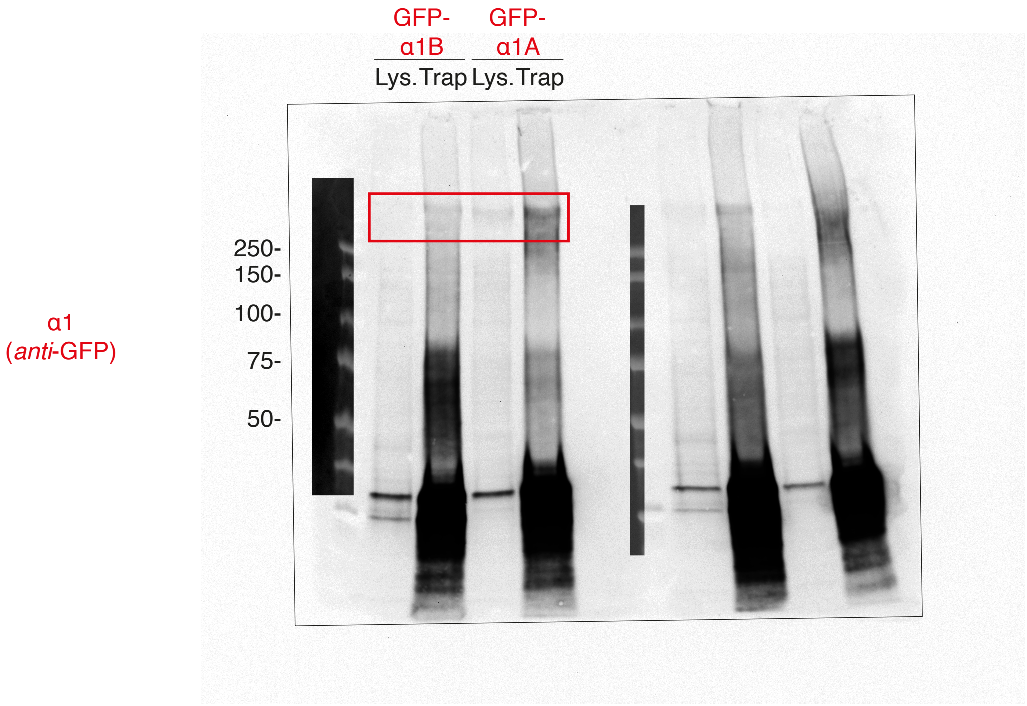

Figure 1D

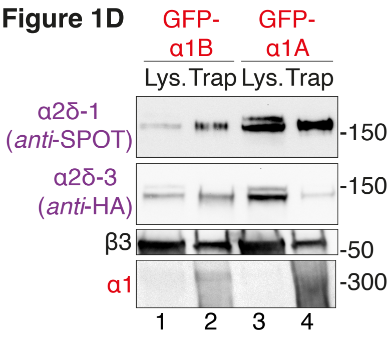

Figure 1D, panel 1, filename: 2022-01-20 11.32.44,3-2.scan_raw_a2d1.png

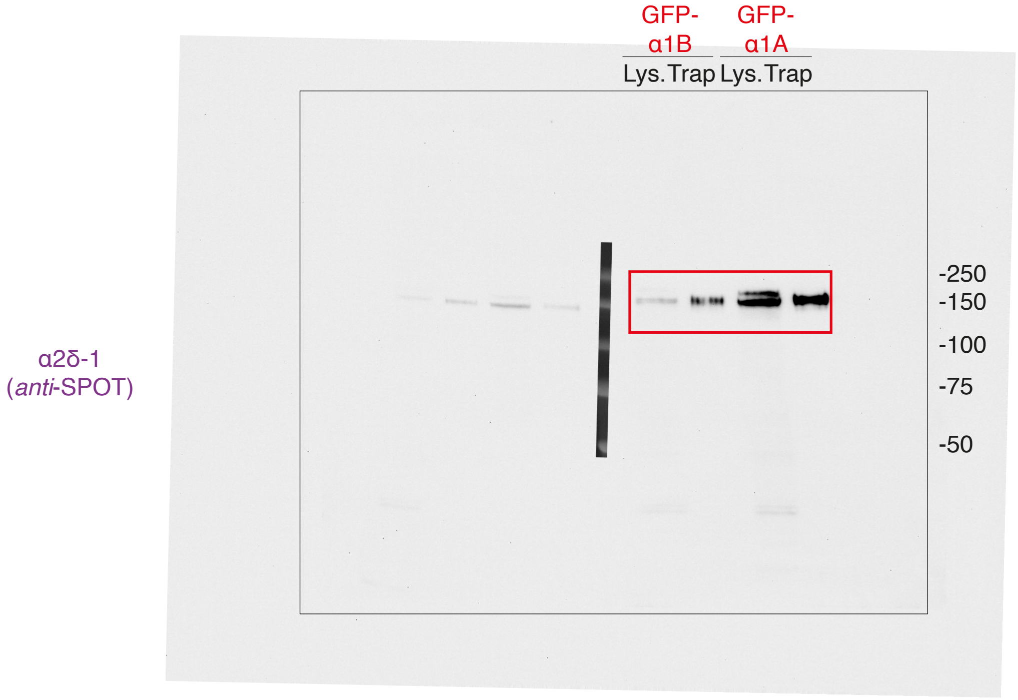

Figure 1D, panel 2, filename: 2022-01-19 12.47.45,5-3. scan_raw.png

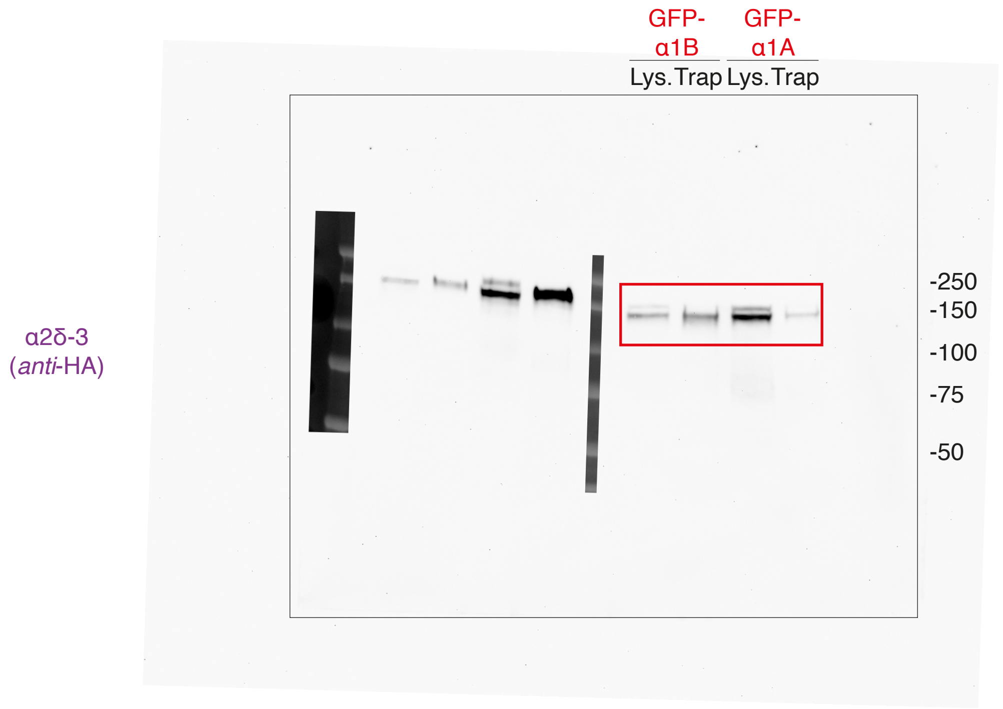

Figure 1D, panel 3, filename: 2022-01-20 11.47.12,1-1.scan_raw.png

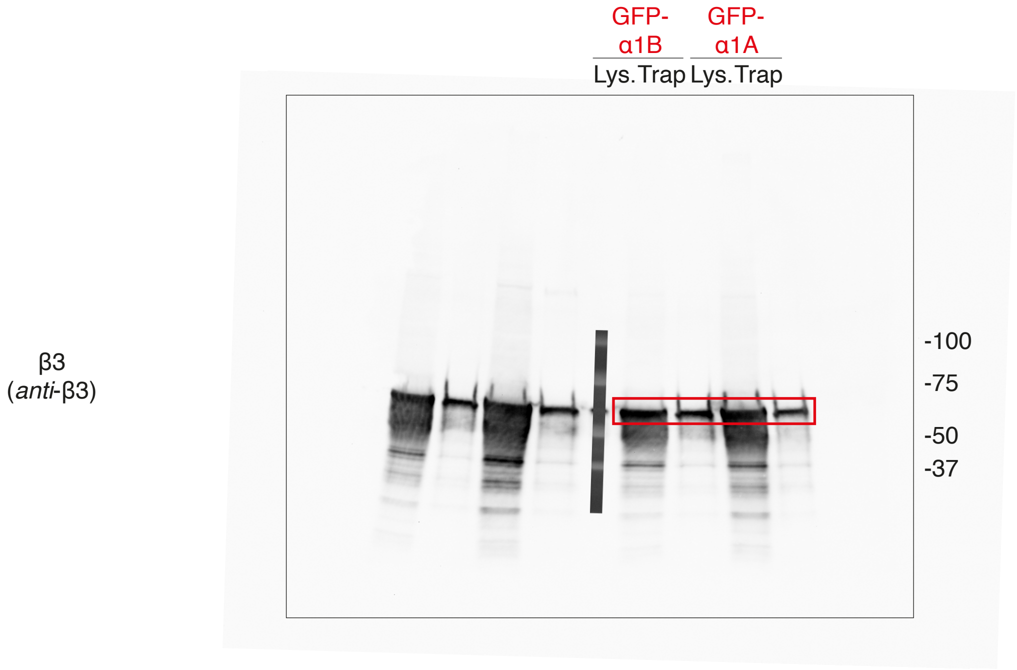

Figure 1D, panel 4, filename: 2022-03-08 11.32.07,3-2.scan_raw Kopie.png

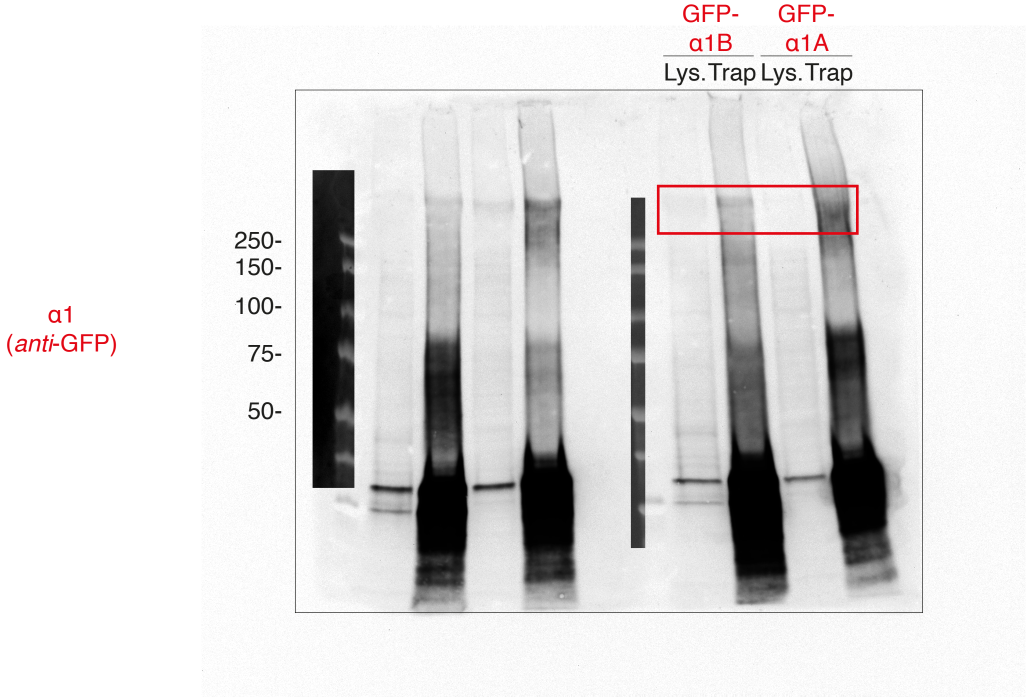

Figure 1F

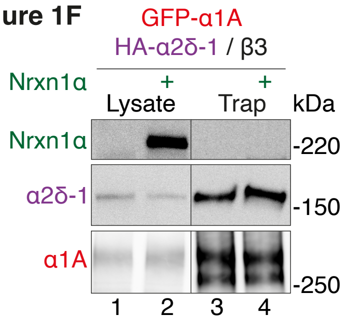

Figure 1F, panel 2, filename: 20210901,3-2.scan_raw.png

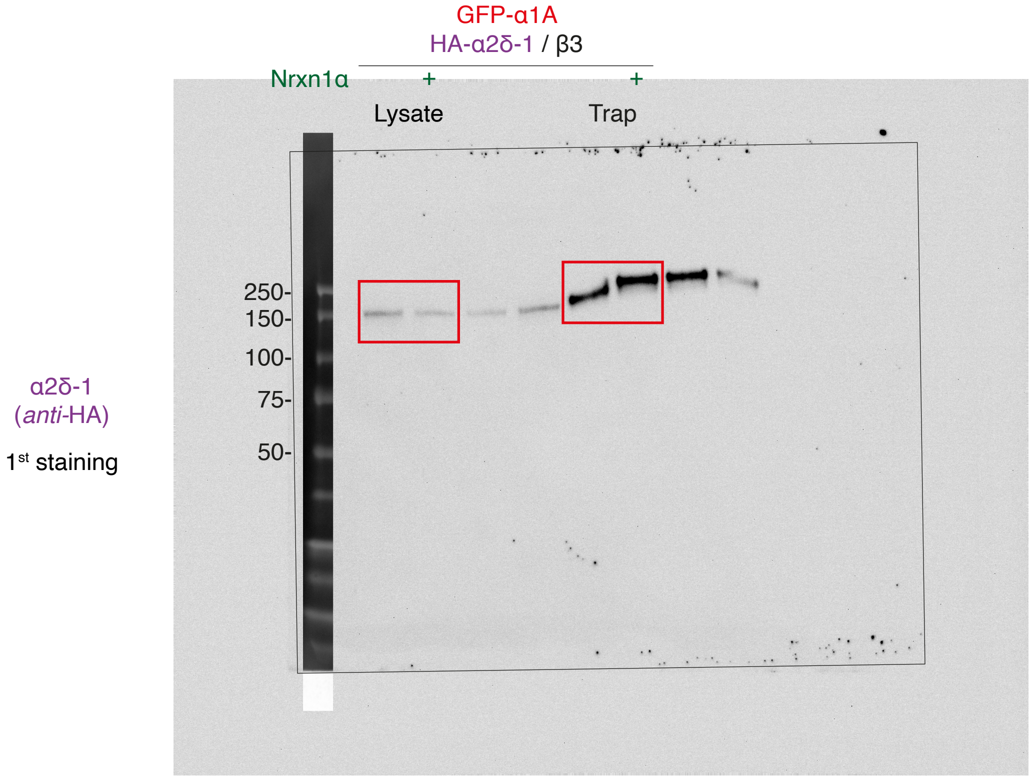

Figure 1F, panel 1, filename: 2021-09-02 11.04.14,3-2.scan_raw_kopie.png

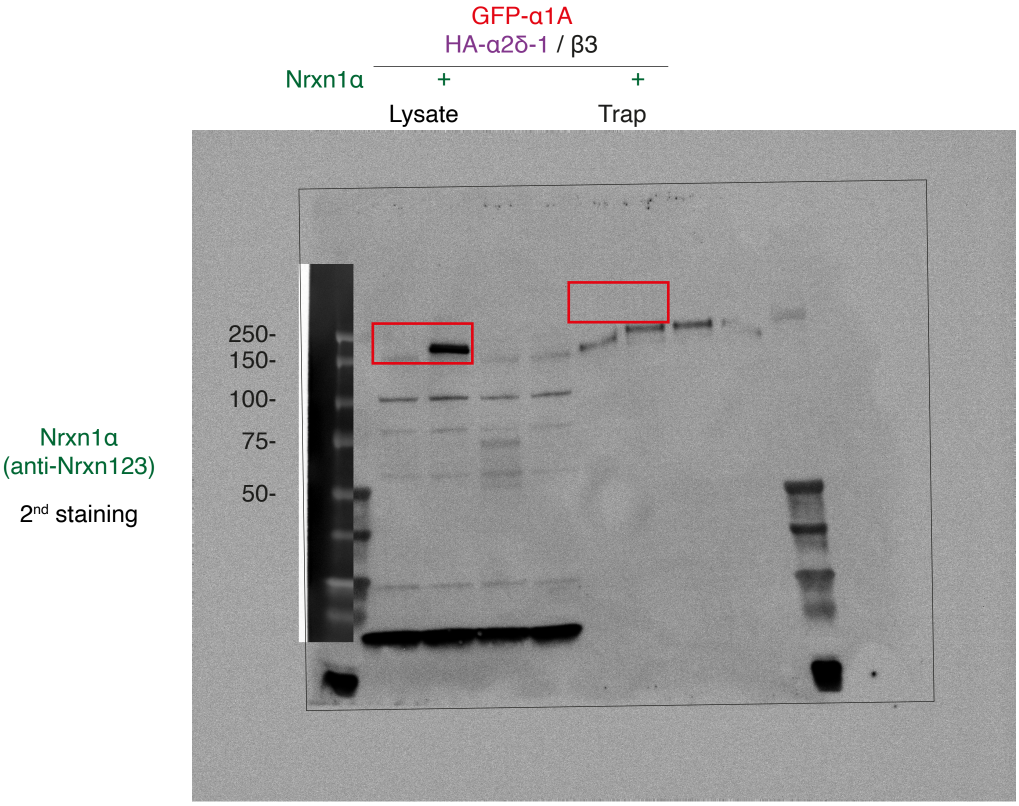

Figure 1F, panel 3, filename: 2021-09-08 10.46.59,3-2.scan_raw.png

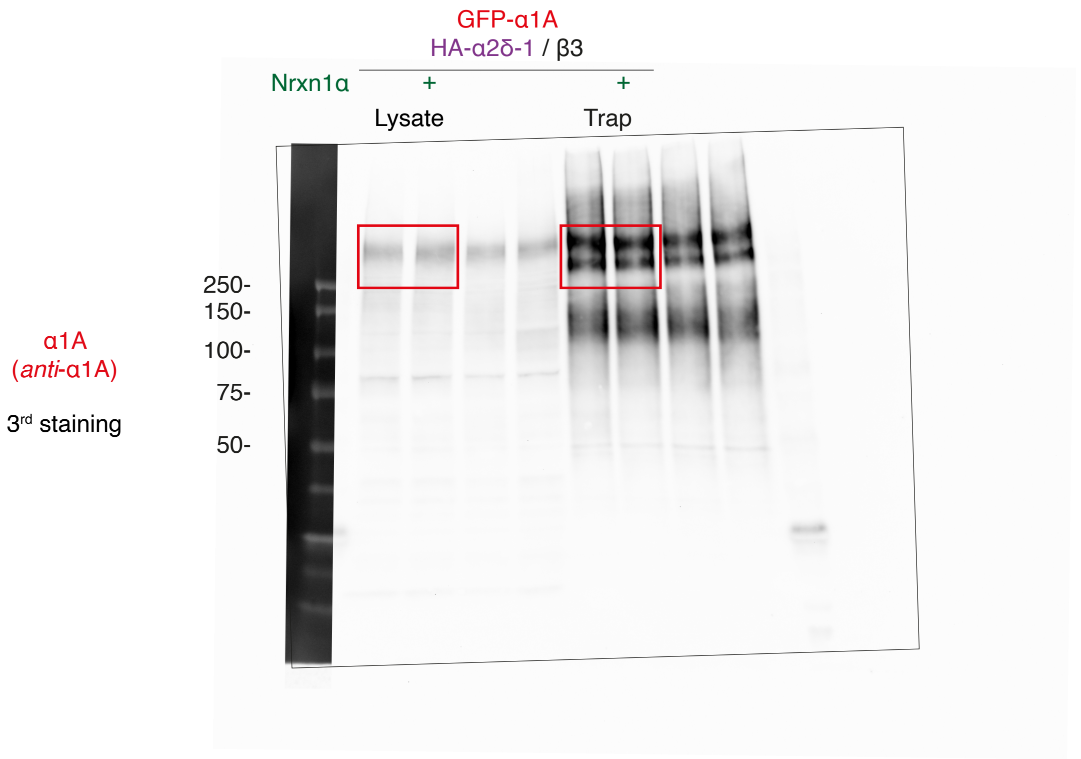

Figure 1G

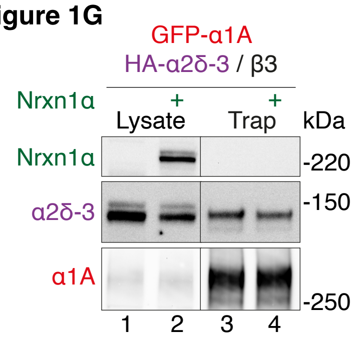

Figure 1G, panel 2, filename: 2021-09-08 11.22.10,3-2.scam_raw Kopie.png

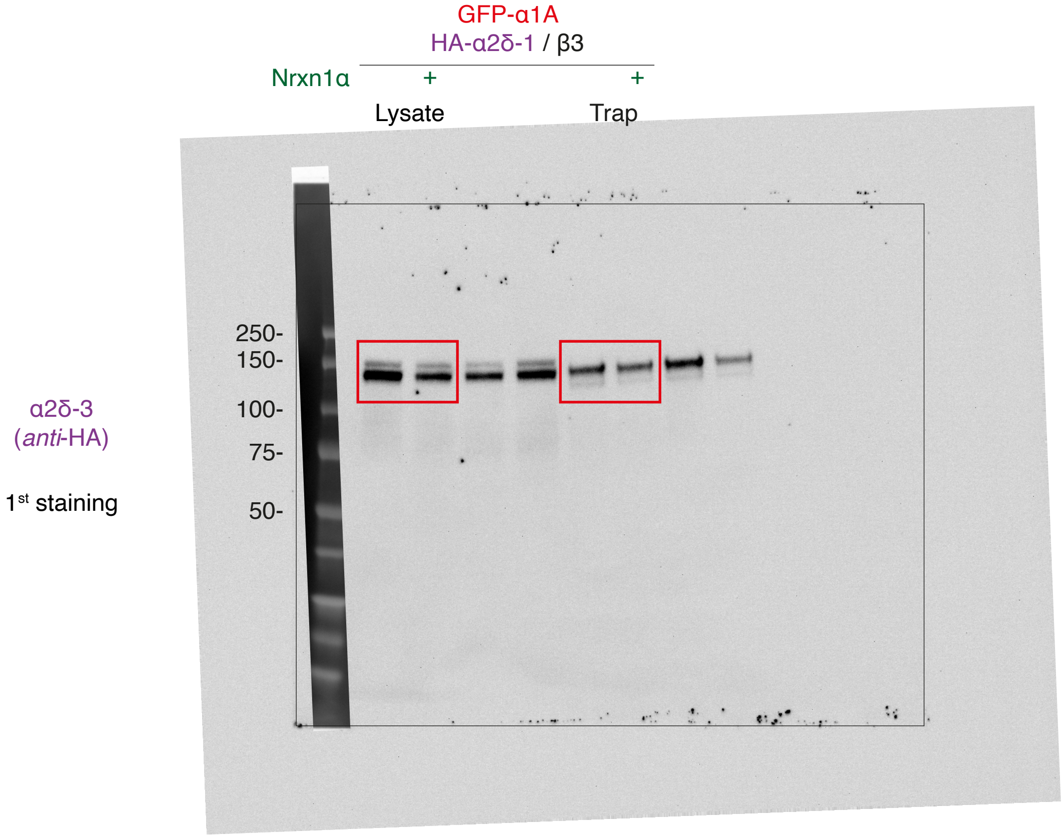

Figure 1G, panel 1, filename: 2021-09-09 11.01.13,3-2. scan_raw.png

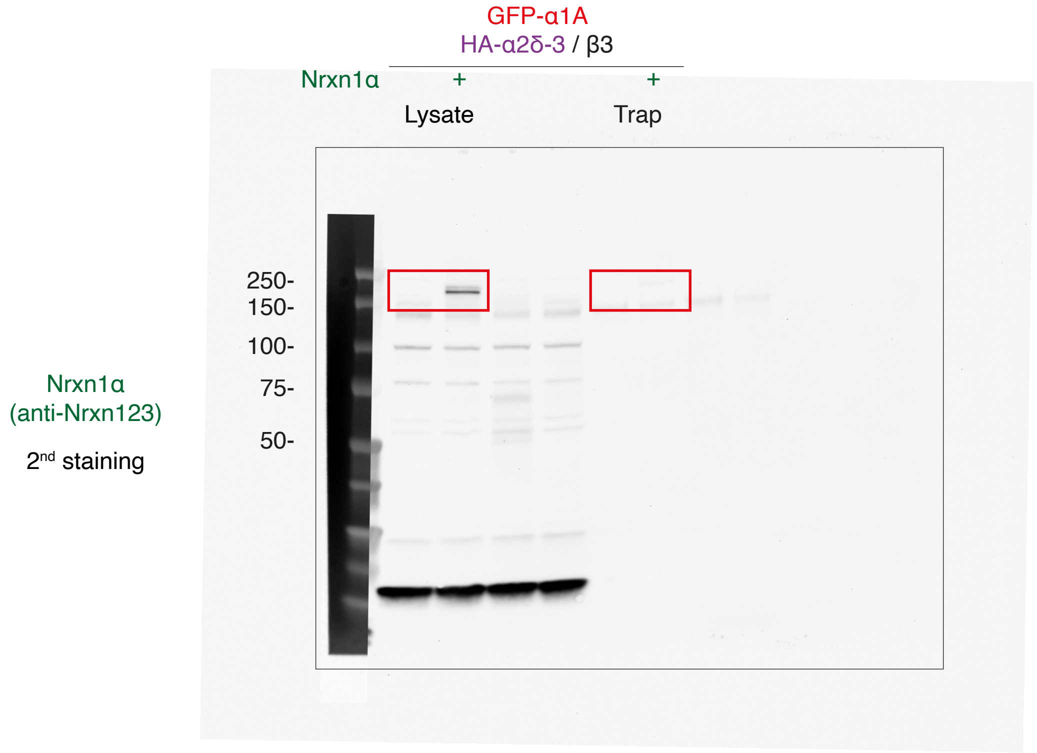

Figure 1G, panel 3, filename: 2021-09-08 10.46.59,3-2.scan_raw.png

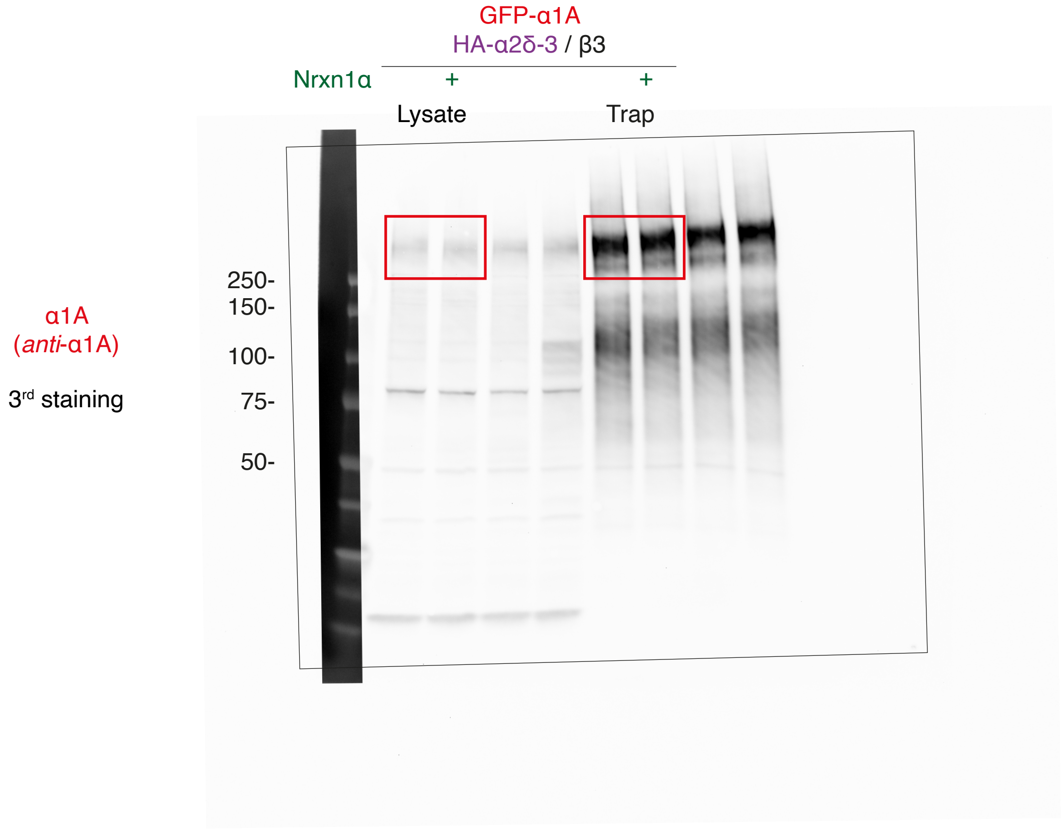

Figure 1H

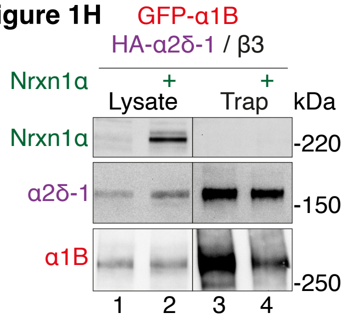

Figure 1H, panel 2, filename: 2022-02-04 14.02.37,5-3.scan_raw.png

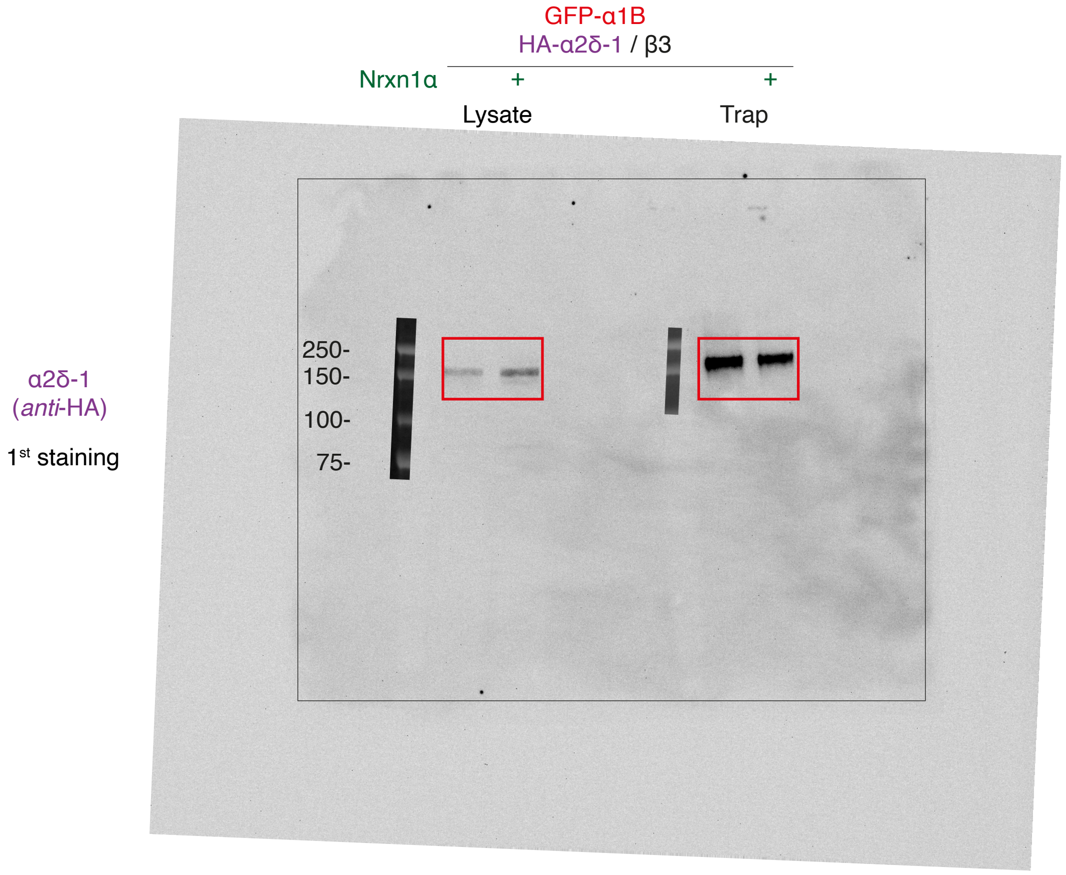

Figure 1H, panel 1, filename: 2022-02-08 12.01.37,5-3.scan_raw.png

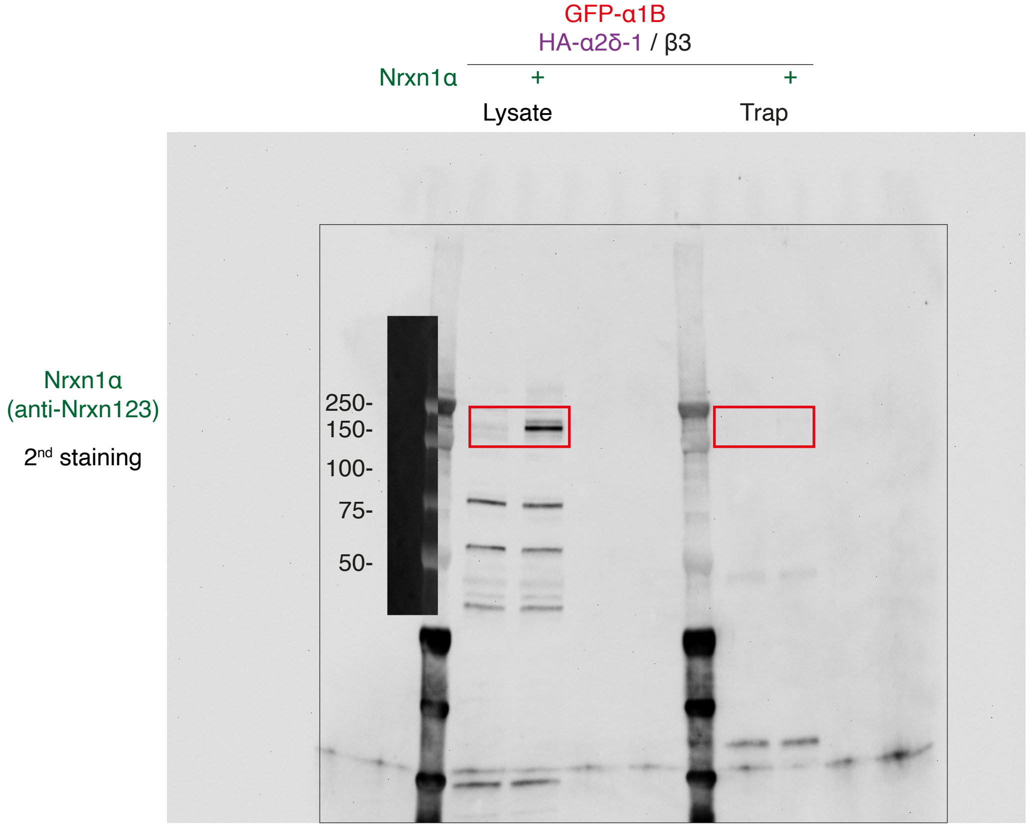

Figure 1H, panel 3, filename: 2022-02-10 12.23.57,1-1. scan_raw.png

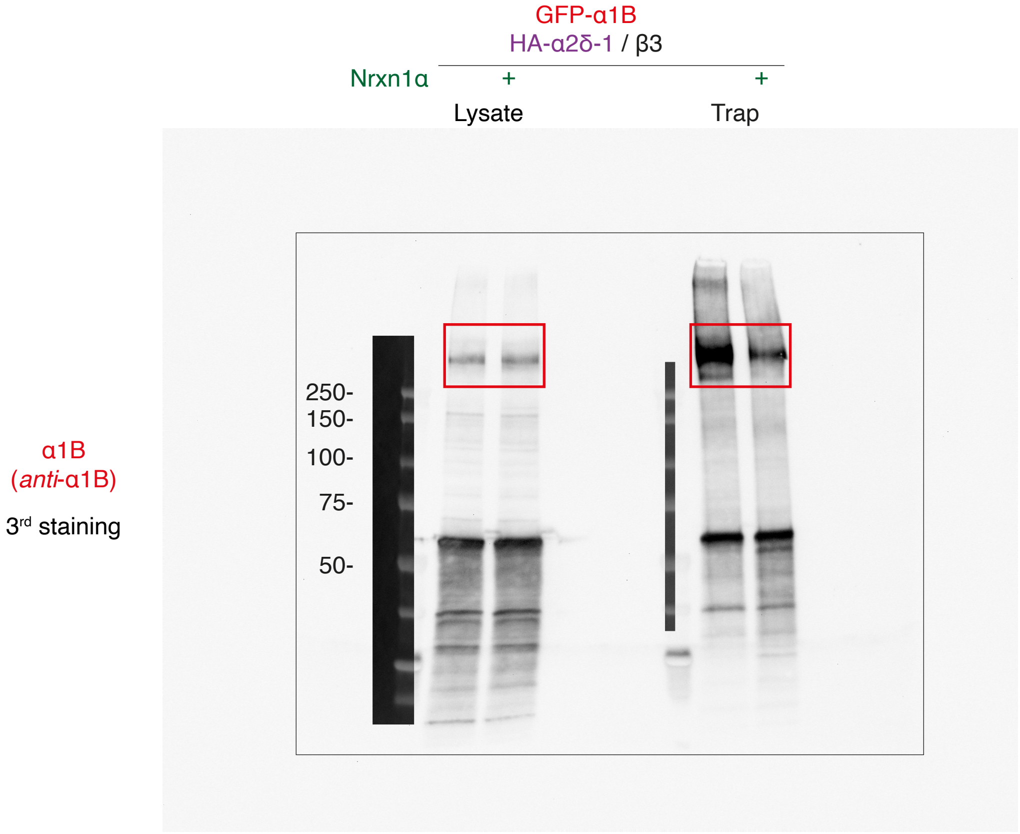

Figure 1I

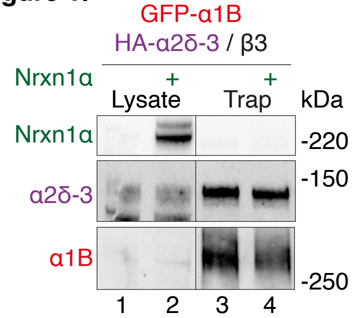

Figure 1I, panel 2, filename: 2021-02-24 12.22.58,5-3. scan_raw.png

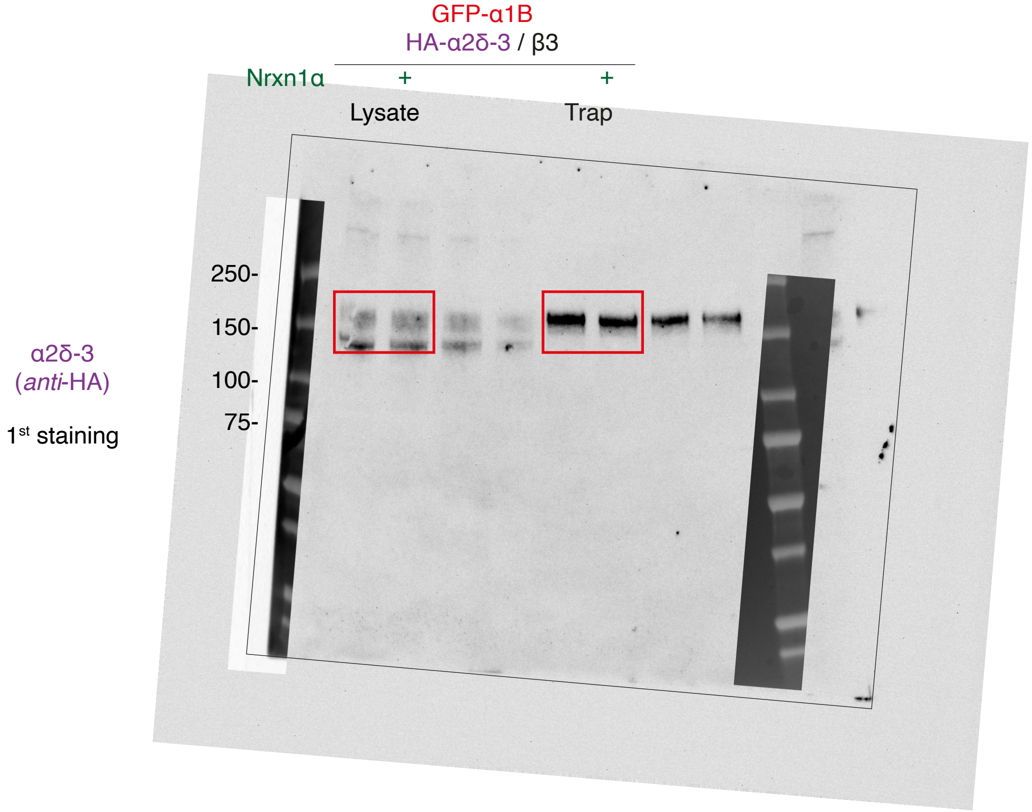

Figure 1I, panel 1, filename: 2021-02-26 13.22.05,5-3. scan_raw.png

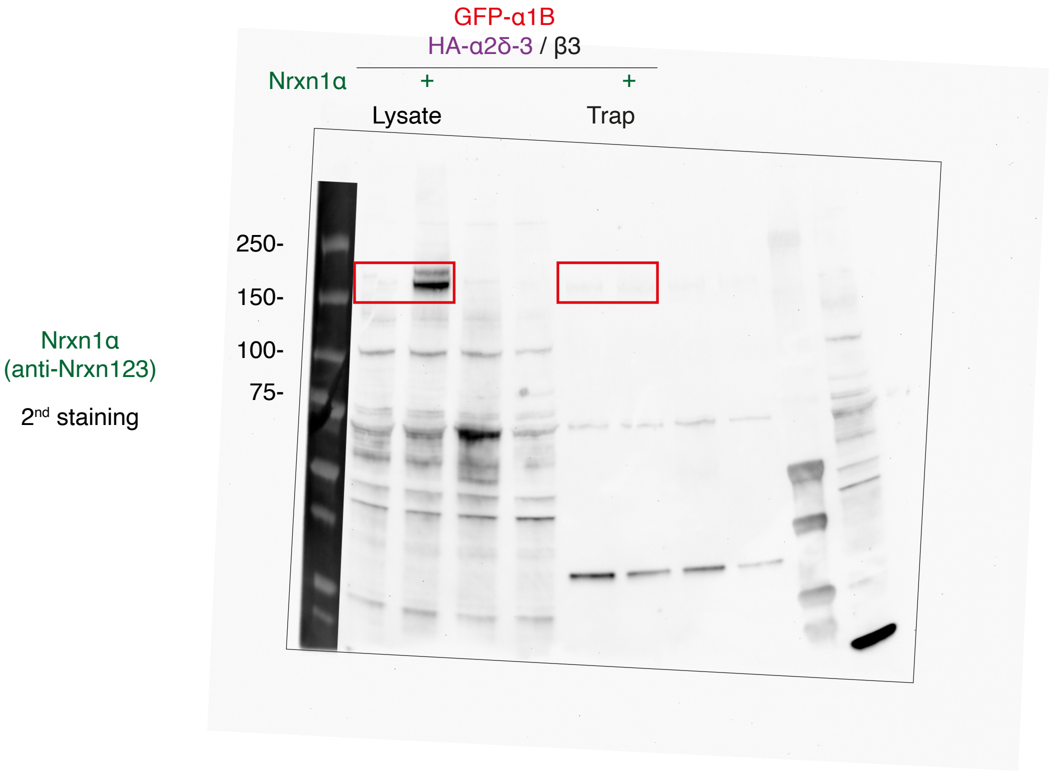

Figure 1I, panel 3, filename: 2021-03-04 13.34.08,5-3. scan_raw.png

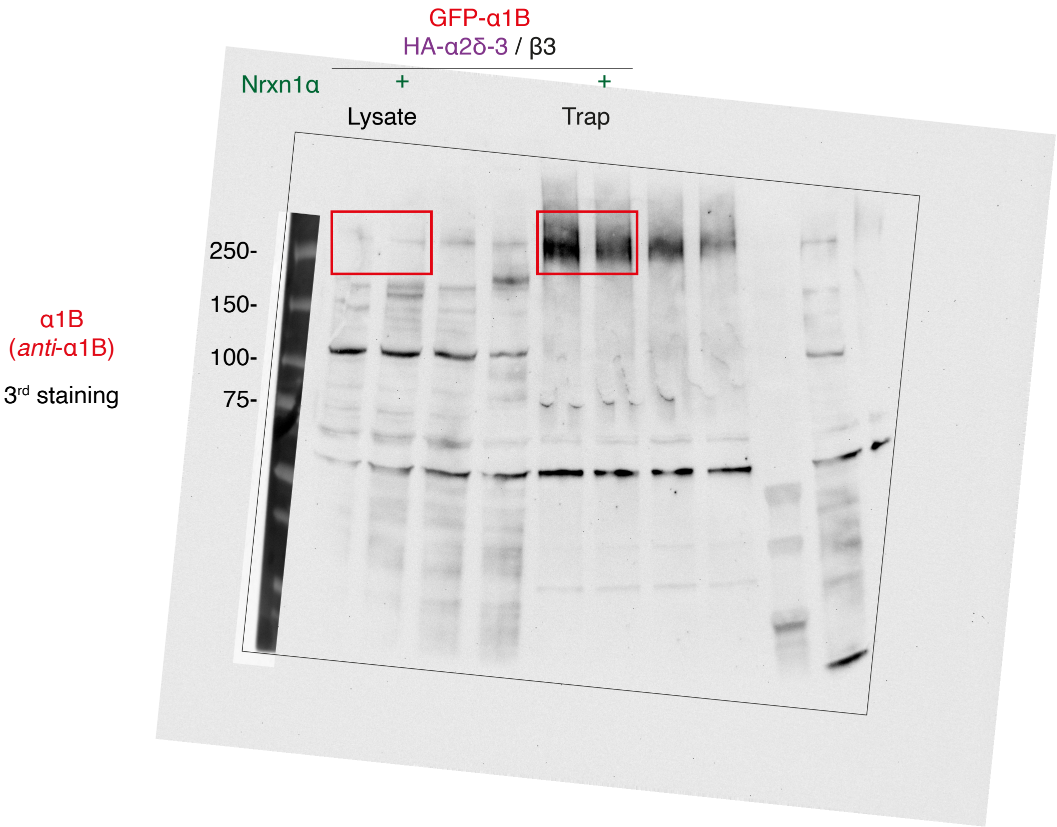

Figure 1J

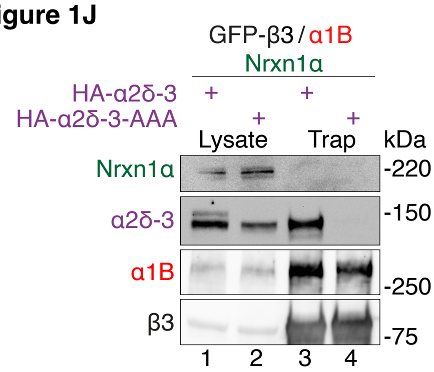

Figure 1J, panel 2, filename: 2021-08-04 10.16.19,3-2.scan_raw_HA-a2d-3.png

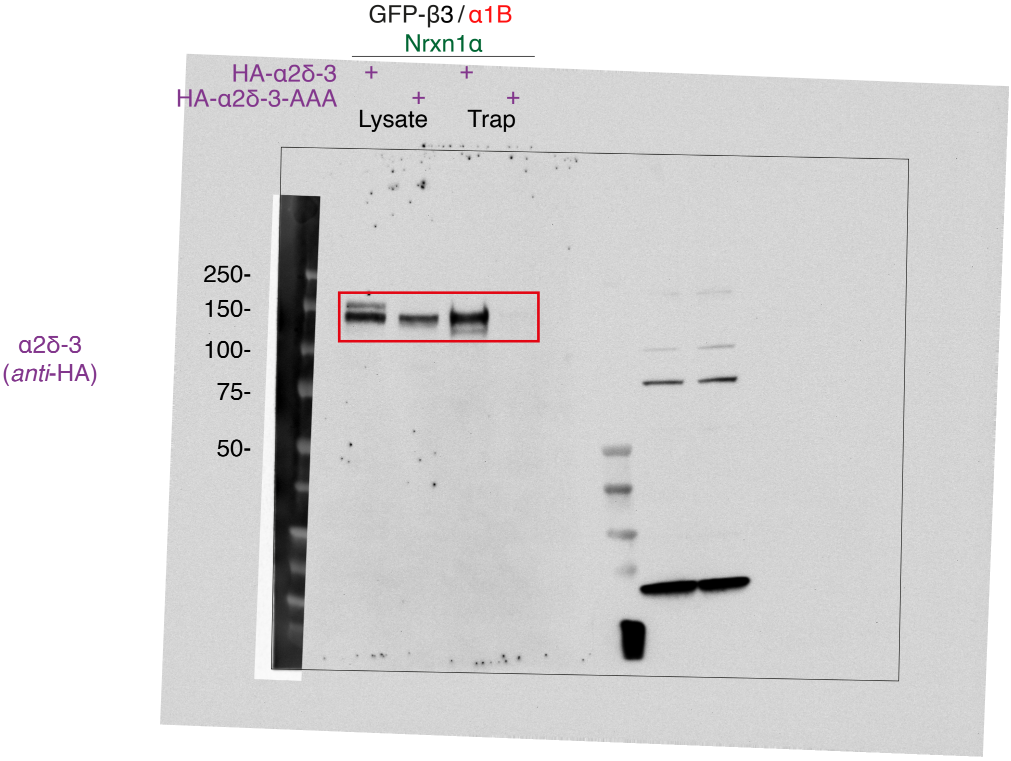

Figure 1J, panel 1, filename: 2021-08-04 10.16.10,5-3.scan_raw.png

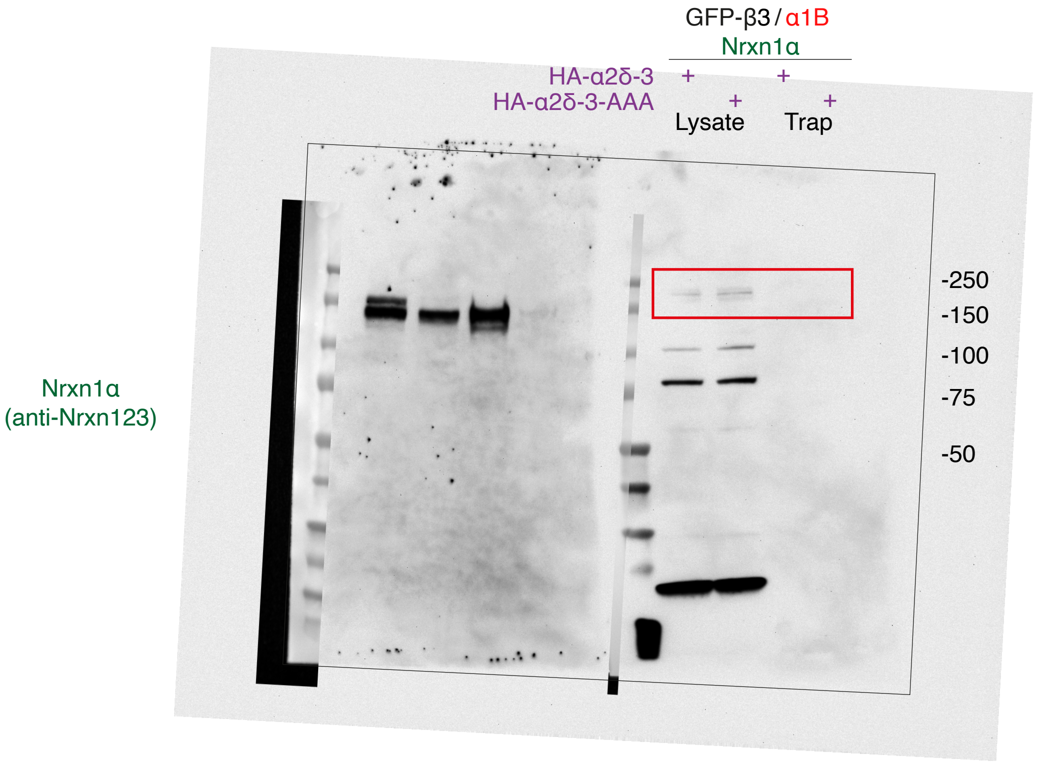

Figure 1J, panel 3, filename: 2021-08-05 10.03.19,5-3. scan_raw_02.png

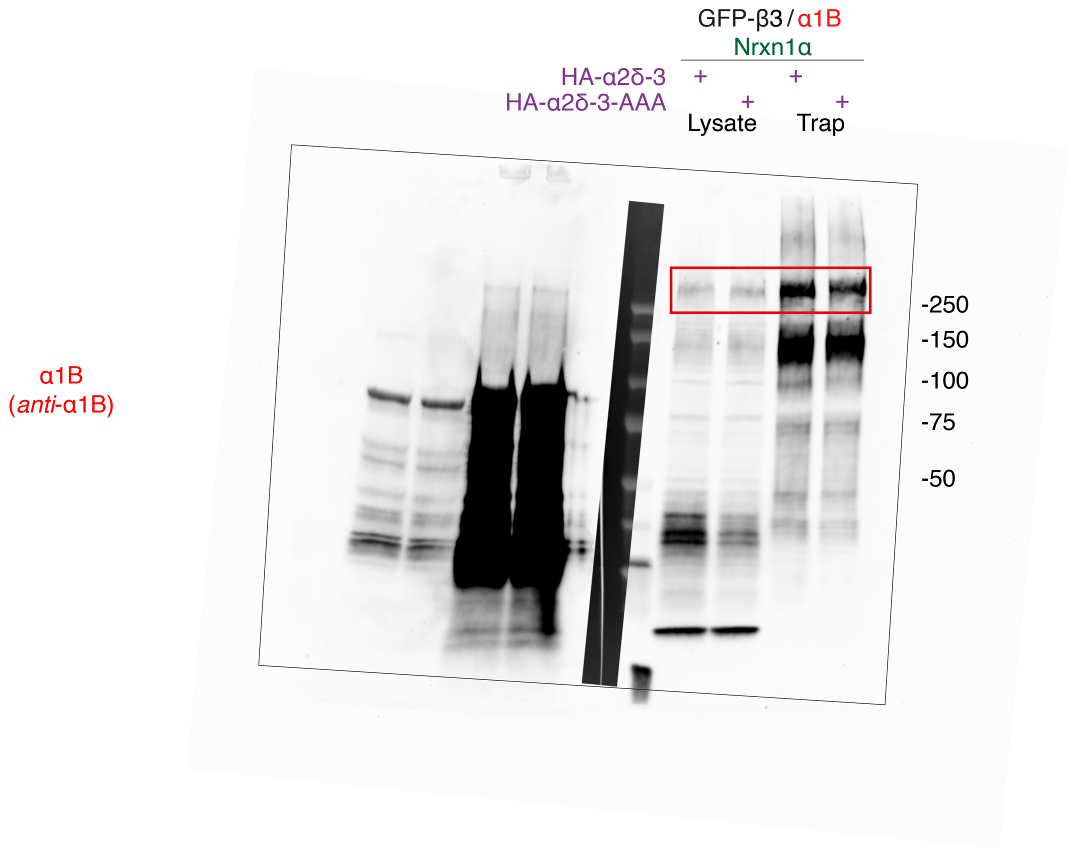

Figure 1J, panel 4, filename: 2021-08-05 10.03.19,2-1. scan+Marker_raw.png

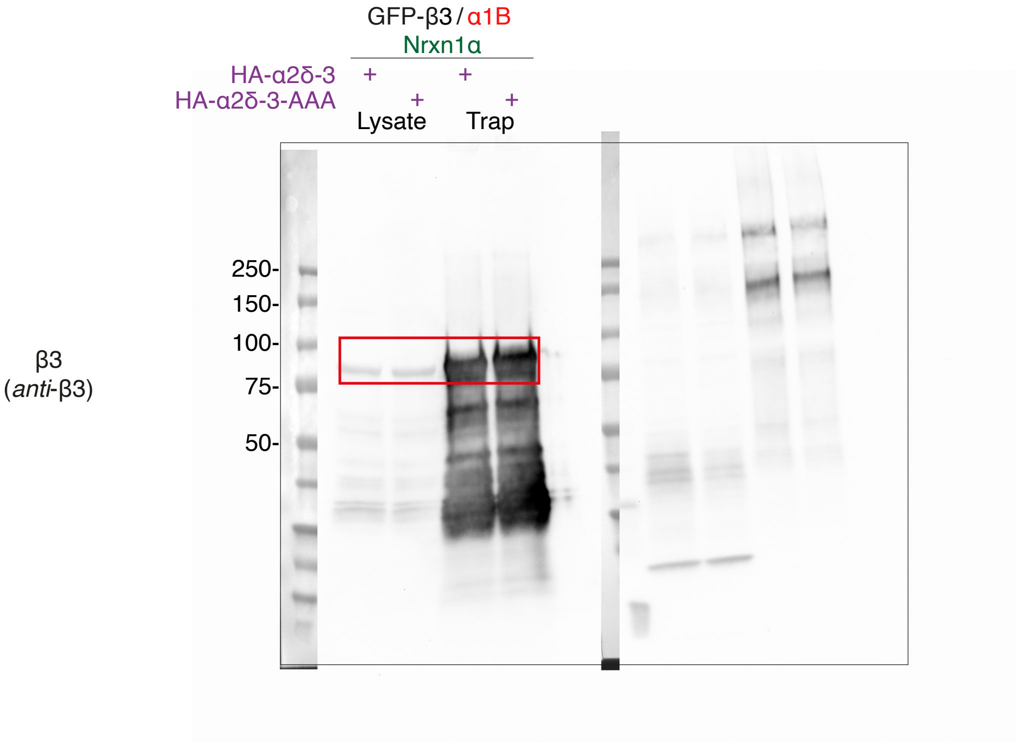

Figure 1K

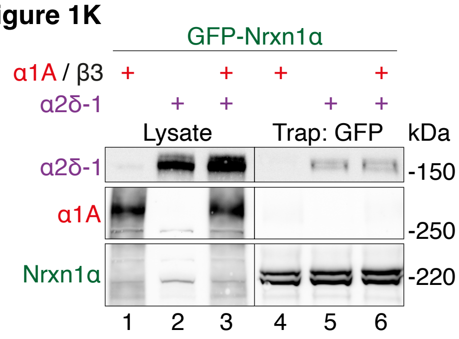

Figure 1K, panel 1, filename: 2023-07-05 10.51.25, 3-2. scan_raw.png

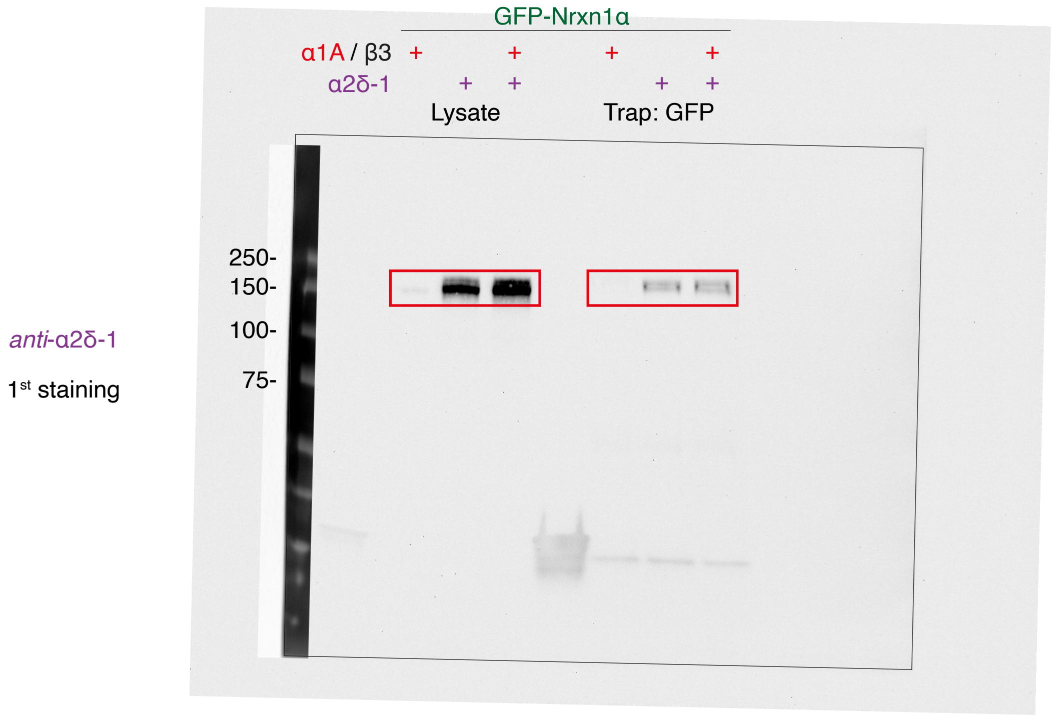

Figure 1K, panel 2, filename: 2023-07-06 10.42.35,3-2. scan_raw.png

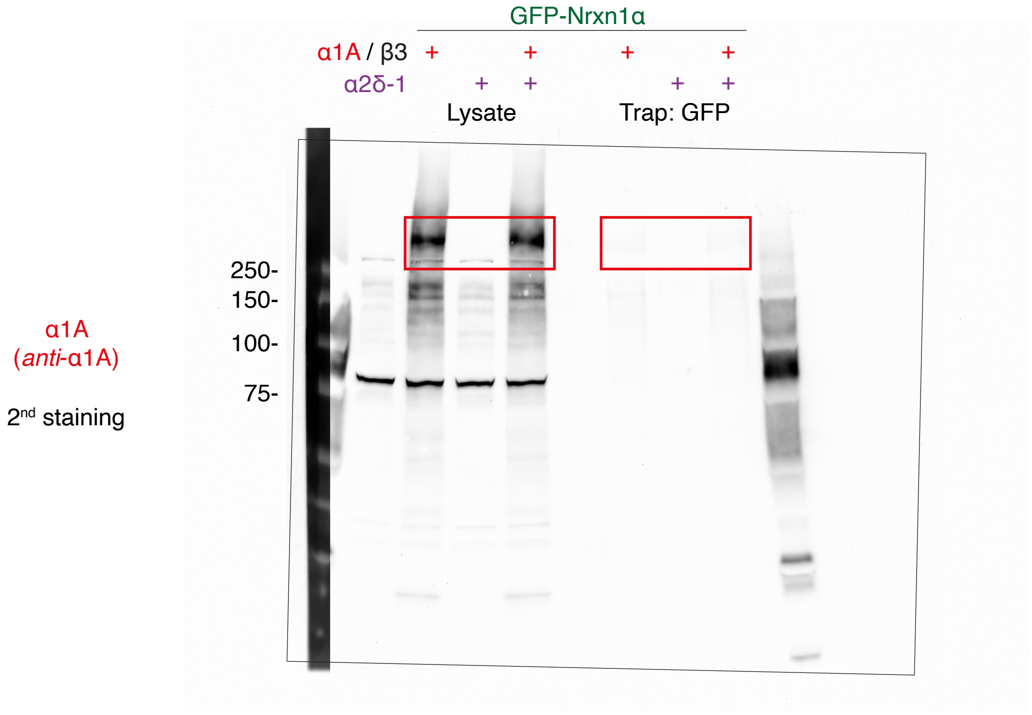

Figure 1K, panel 3, filename: 2023-07-07 10.38.29,3-2. scan_raw.png

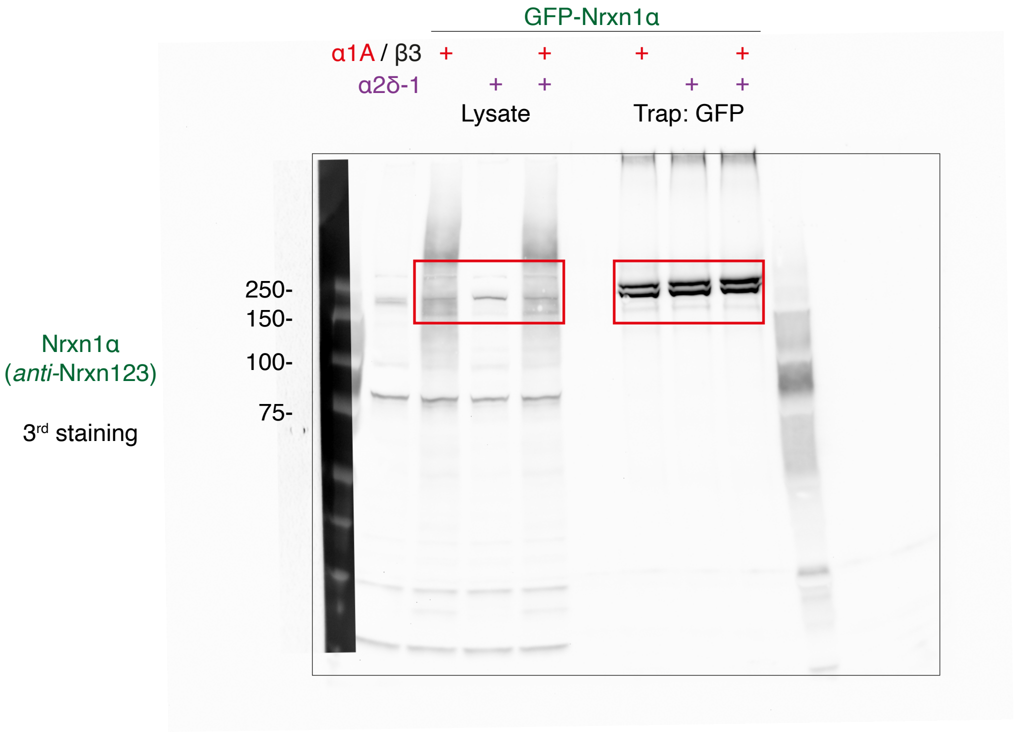

Figure 2B

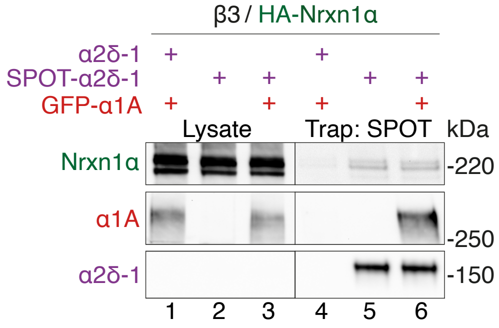

Figure 2B, panel 1 Lysate, filename: 2021-11-04 12.01.20,5-3. scan_raw.png

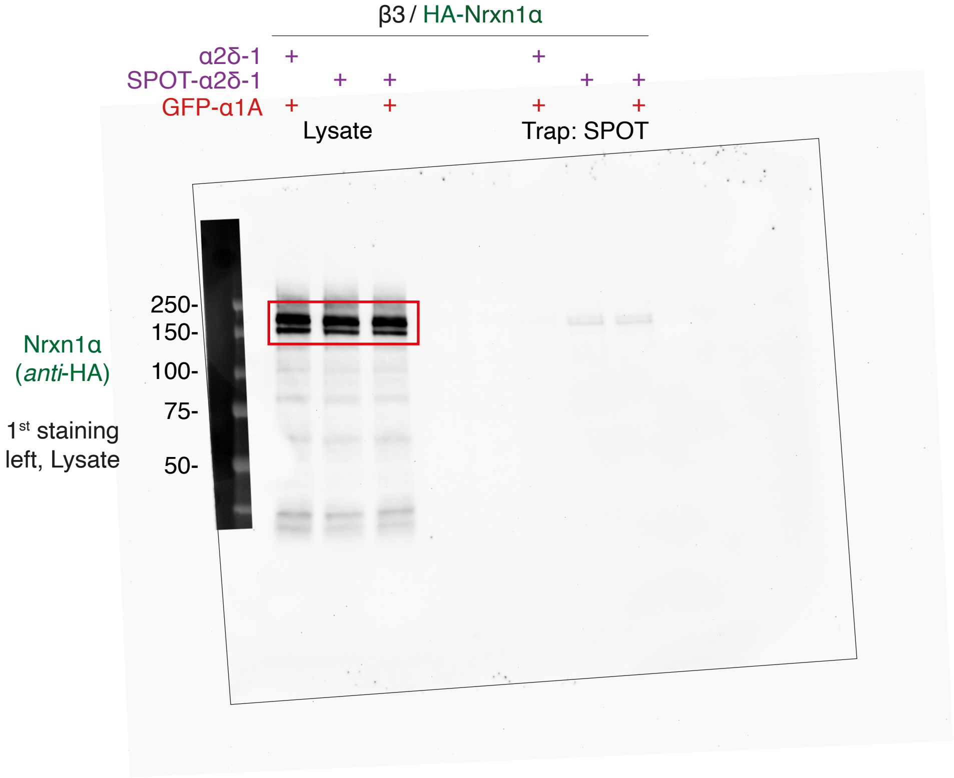

Figure 2B, panel 1 Trap, filename: 2021-11-08 11.21.23,5-3. scan_raw_pushed.png

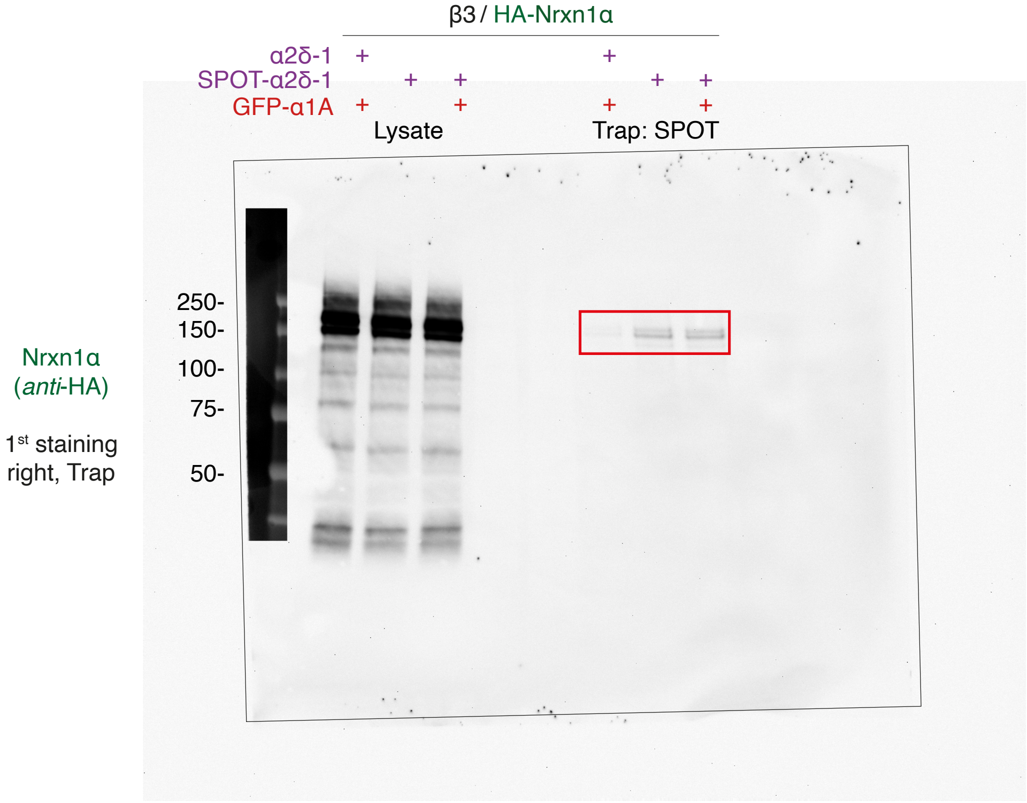

Figure 2B, panel 2, filename: 2021-11-08 11.21.23,5-3. scan_raw.png

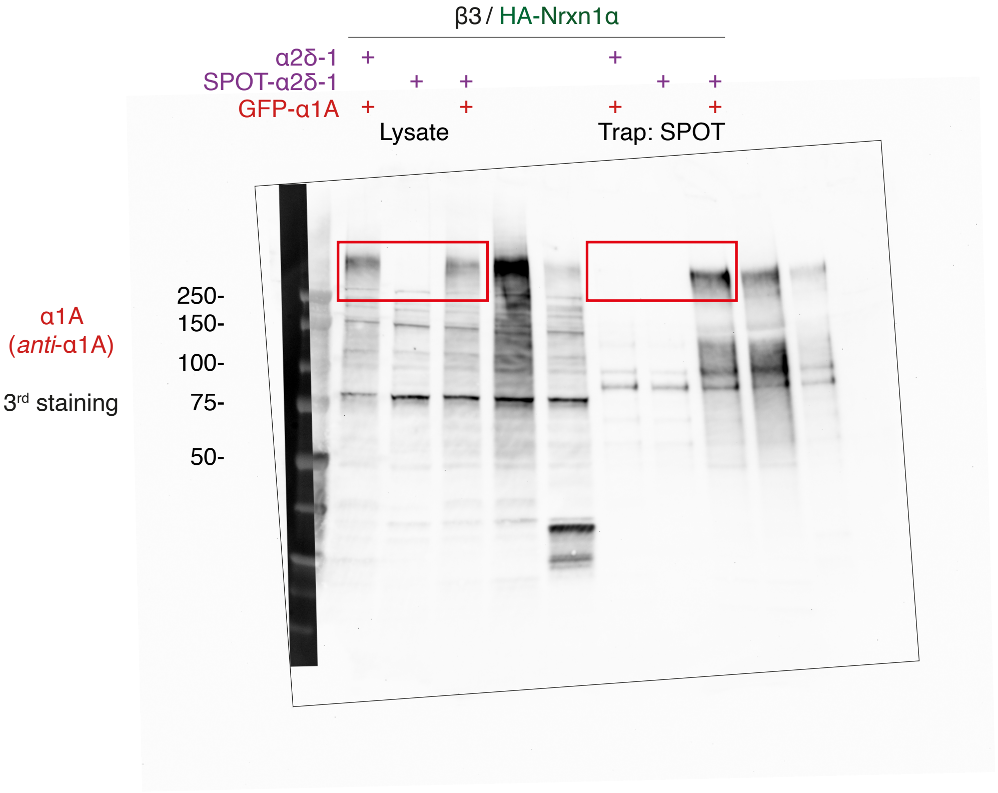

Figure 2B, panel 3, filename: 2021-11-05 12.11.49,3-2. scan_raw.png

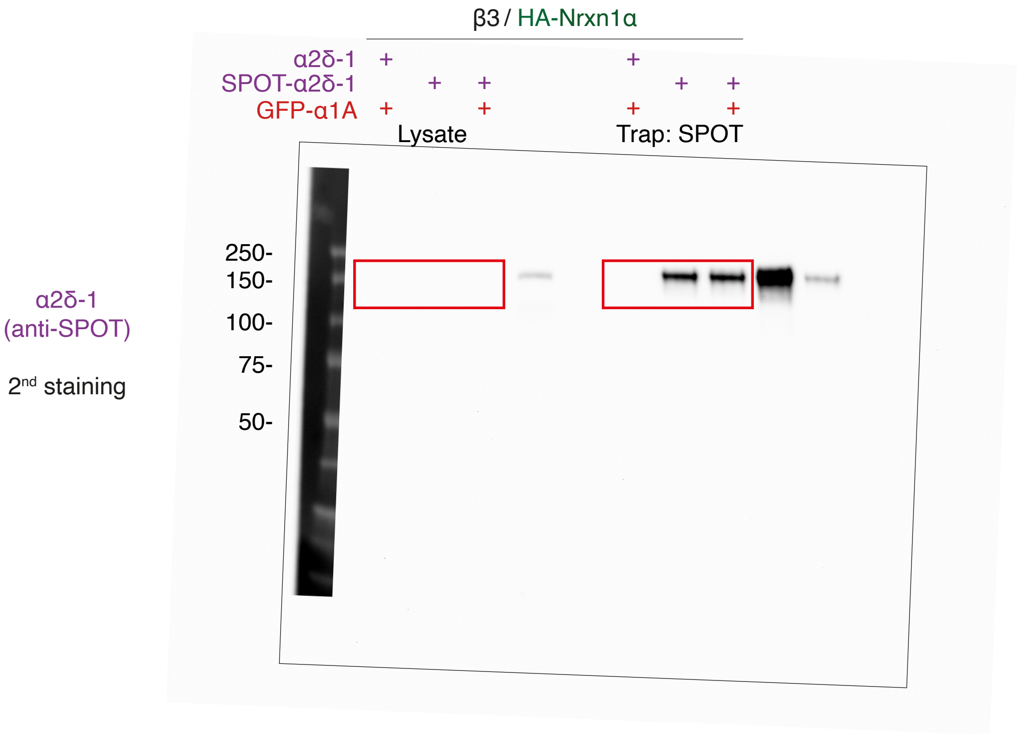

Figure 2C

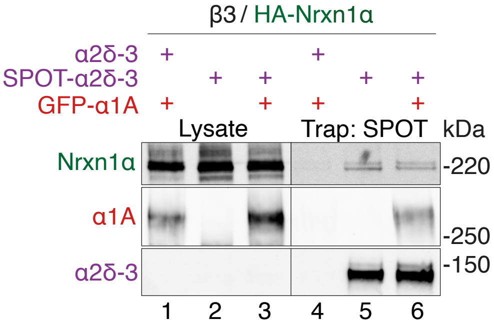

Figure 2C, panel 1 Lysate, filename: 2021-12-08 11.36.08,3-2. scan_raw.png

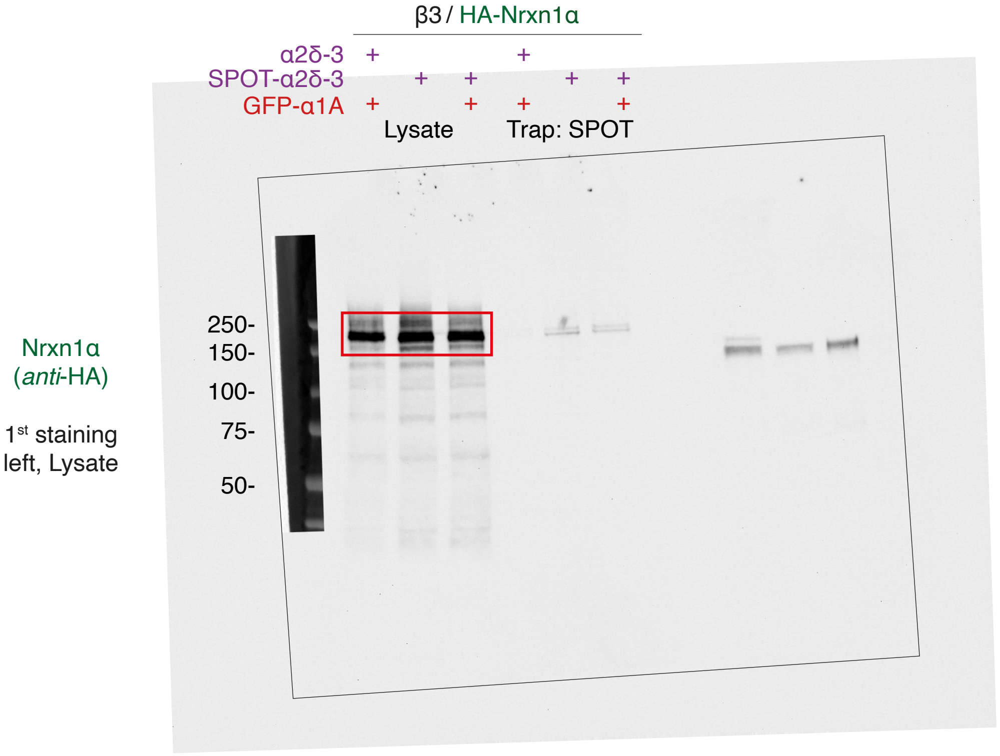

Figure 2C, panel 1 Trap, flename: 2021-12-08 11.36.08,3-2. scan_raw_pushed.png

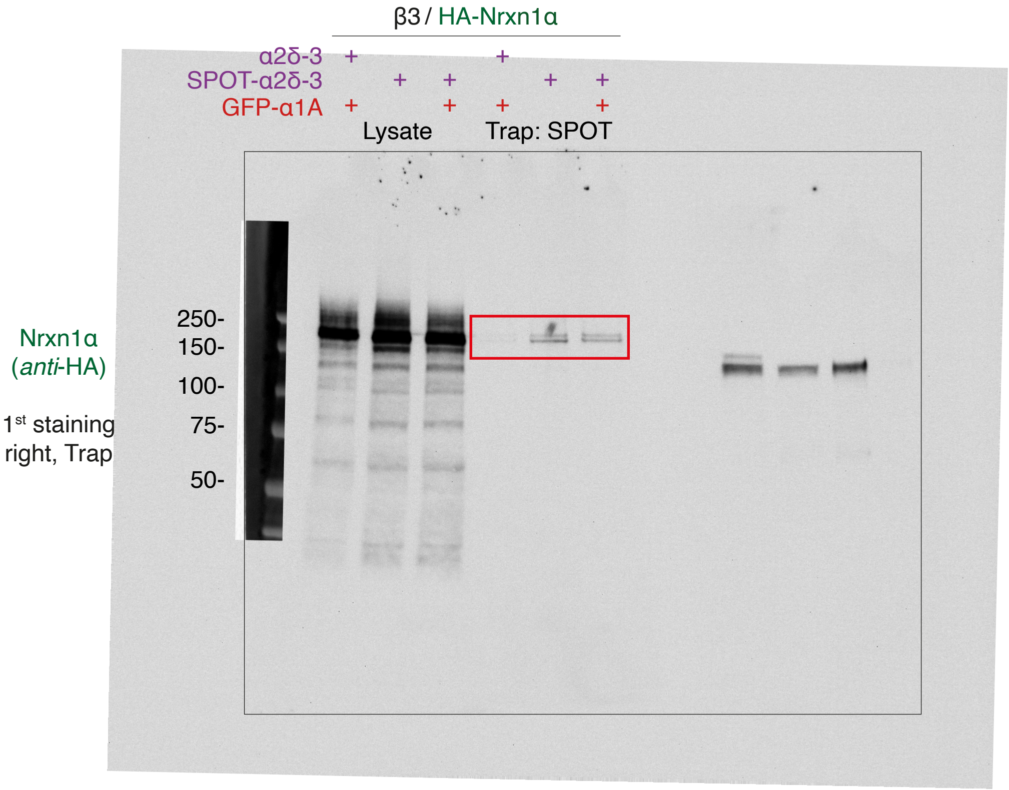

Figure 2C, panel 2, filename: 2021-12-10 11.20.36,5-3. scan_raw.png

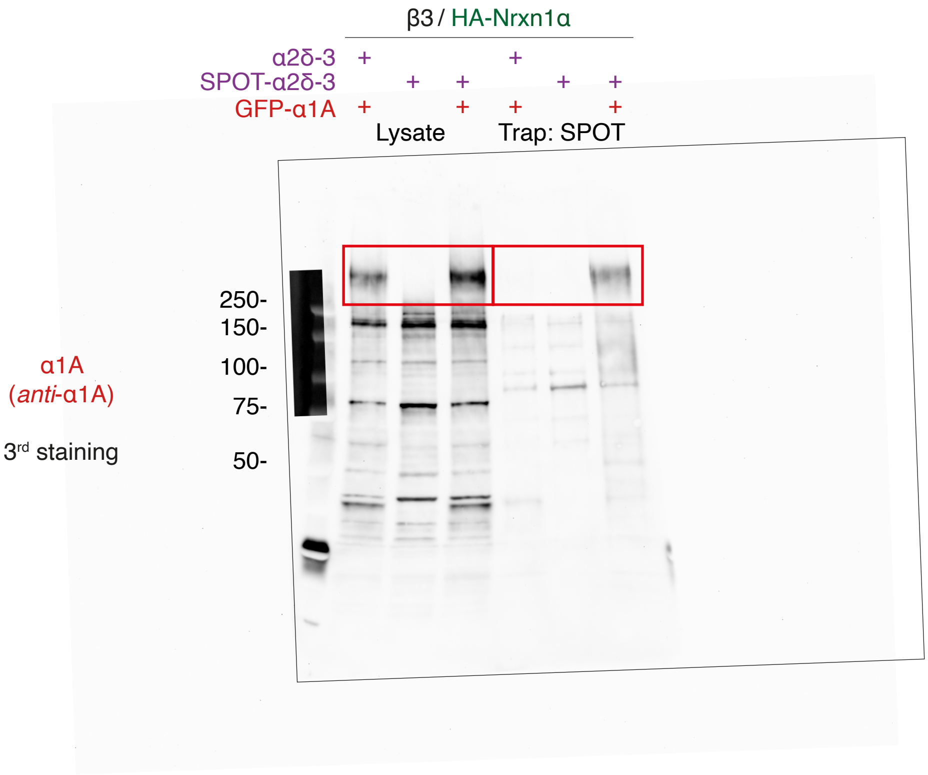

Figure 2C, panel 3, filename: 2021-12-10 11.05.05,3-2. scan_raw.png

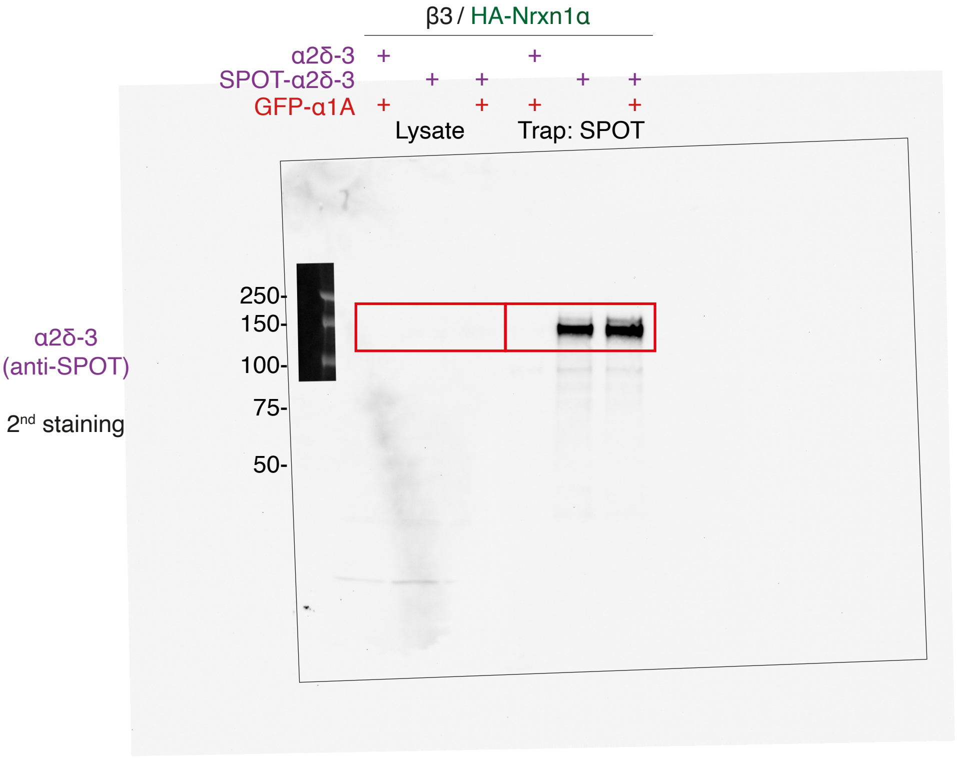

Figure 2D

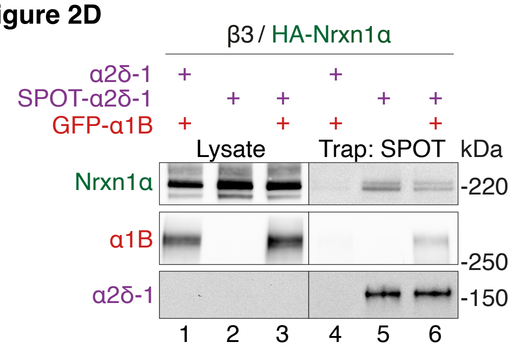

Figure 2D, panel 1 Lysate, filename: 2021-04-08 10.08.16,5-3. scan_raw.png

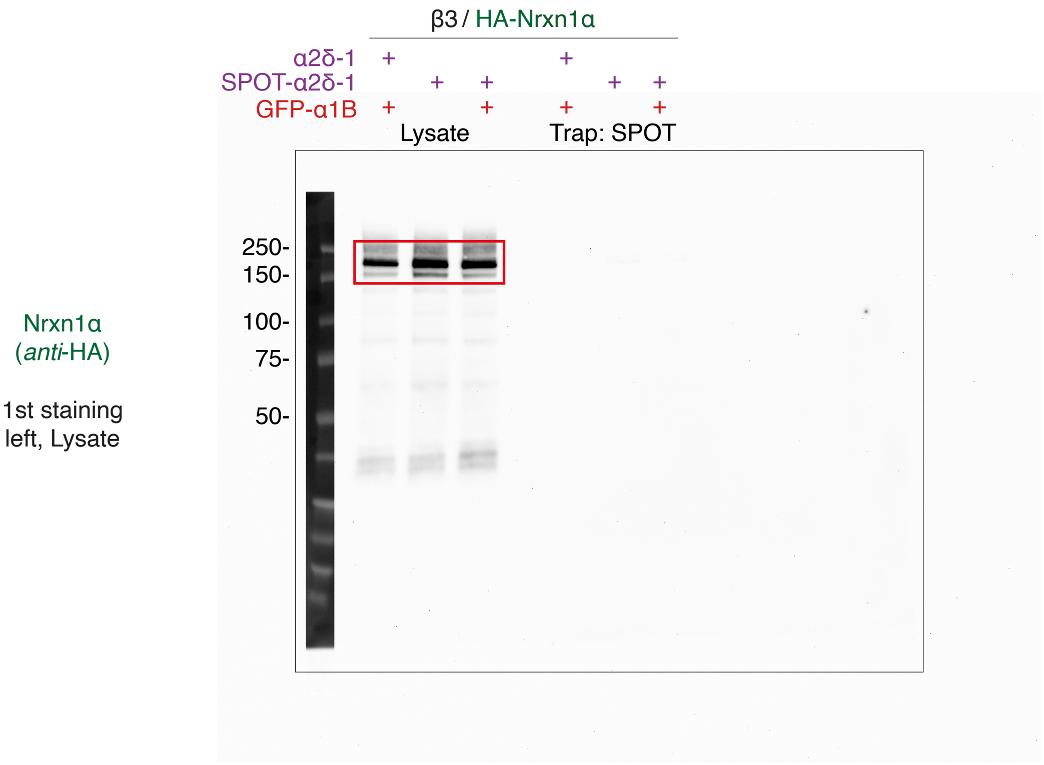

Figure 2D, panel 1 Trap, filename: 2021-04-08 10.08.16,5-3. scan_raw_pushed.png

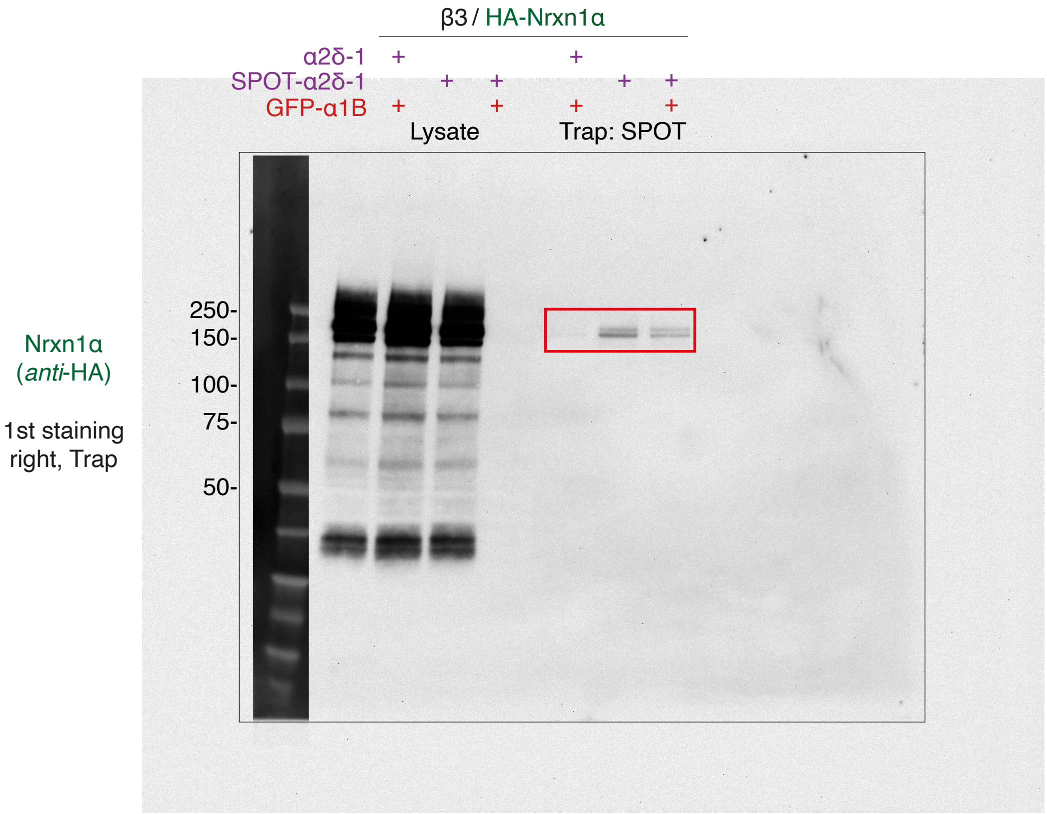

Figure 2D, panel 2, filename: 2021-04-15 16.49.28,5-3. scan_raw.png

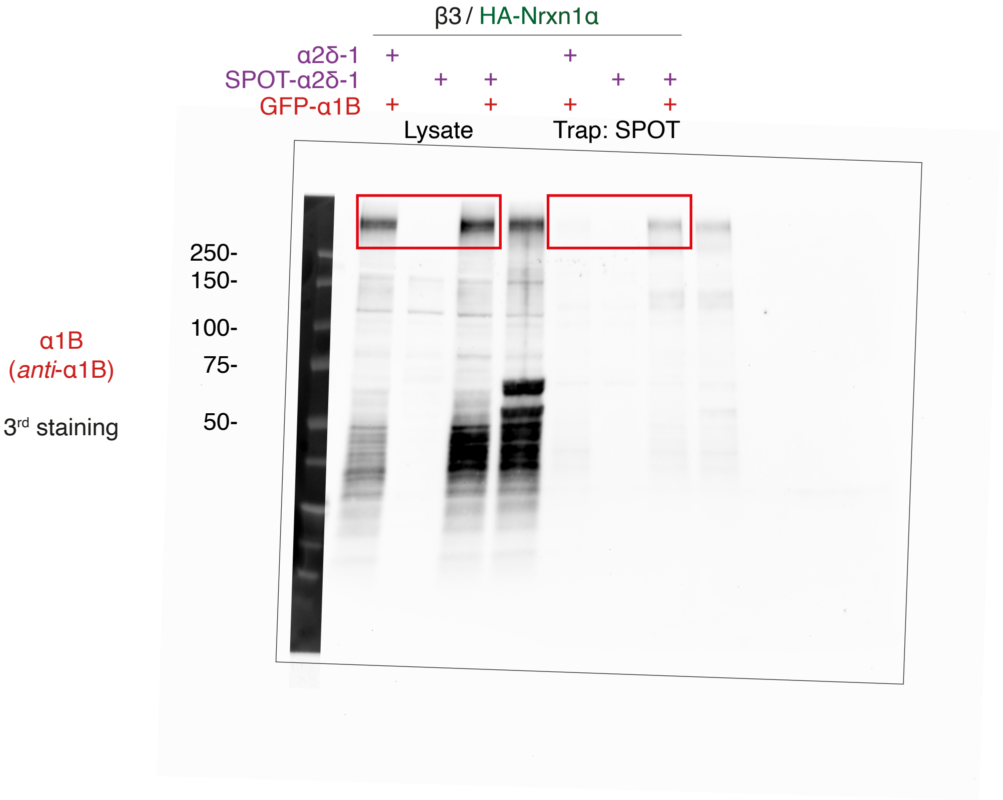

Figure 2D, panel 3, filename: 2021-05-18 11.00.59,3-2. scan_raw.png.png

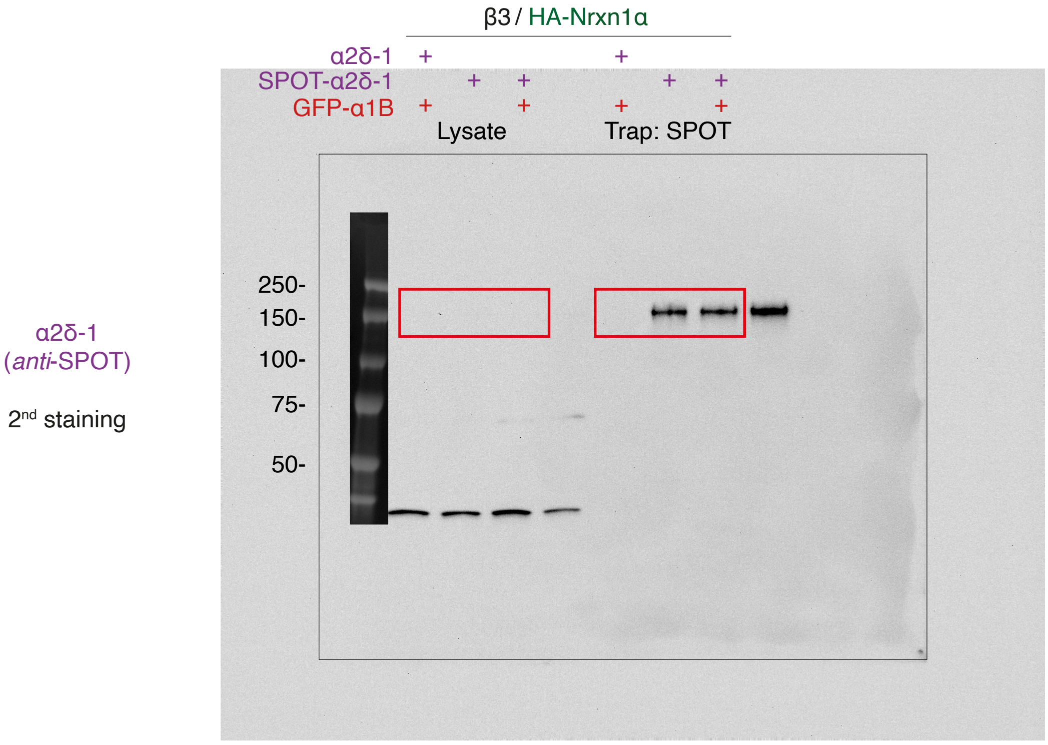

Figure 2E

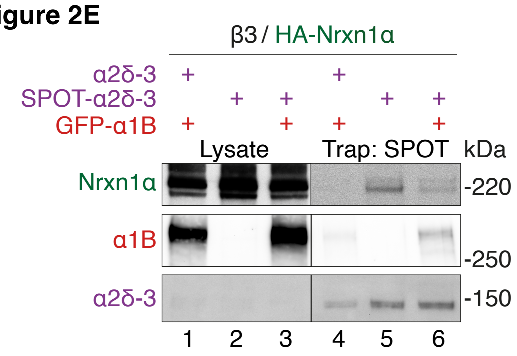

Figure 2E, panel 1 Lysate, filename: 2021-04-08 10.27.30,5-3. scan_raw.png

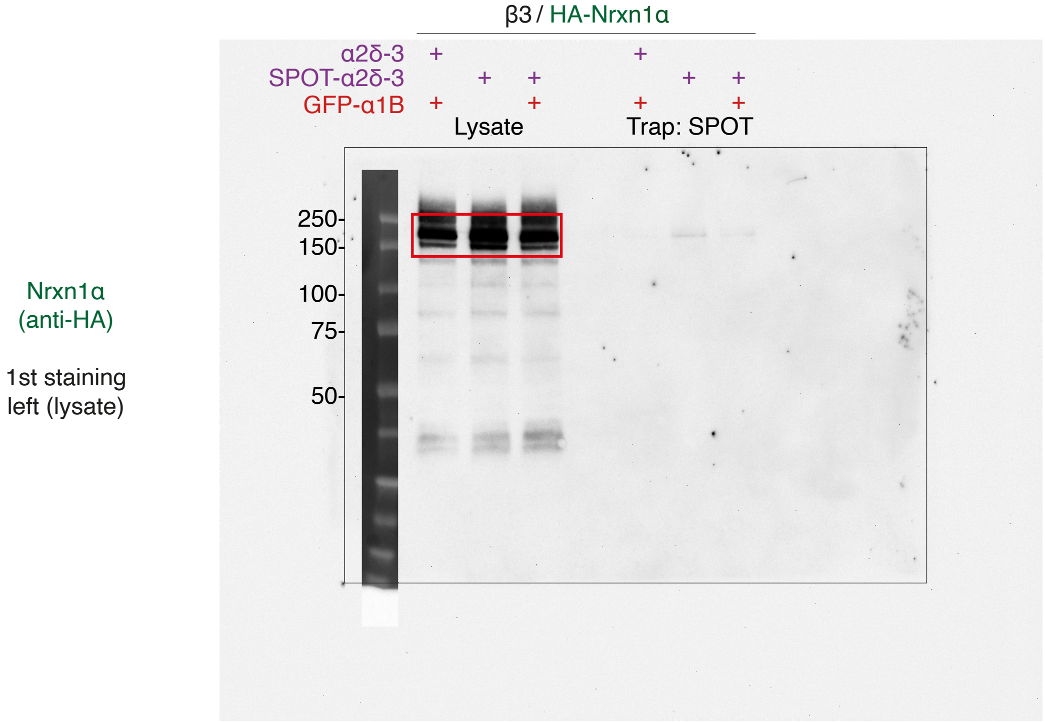

Figure 2E, panel 1 Trap, filename: 2021-04-08 10.27.30,5-3. scan_raw_pushed.png

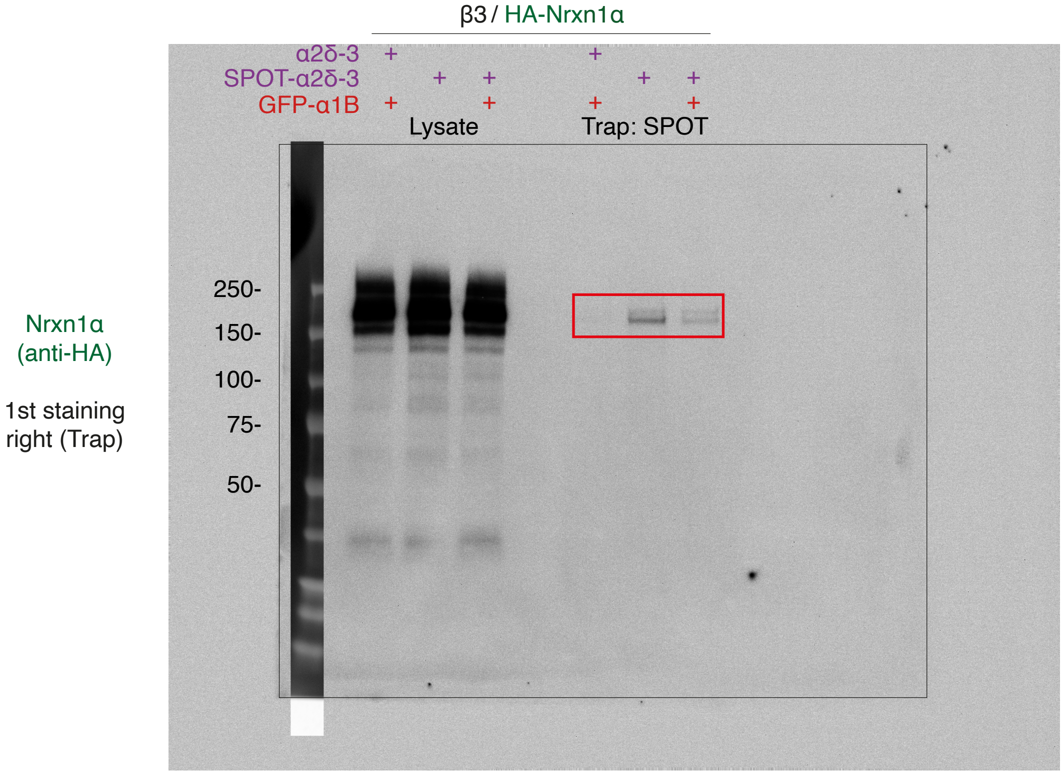

Figure 2E, panel 2, filename: 2021-04-15 17.02.12,5-3. scan_raw.png

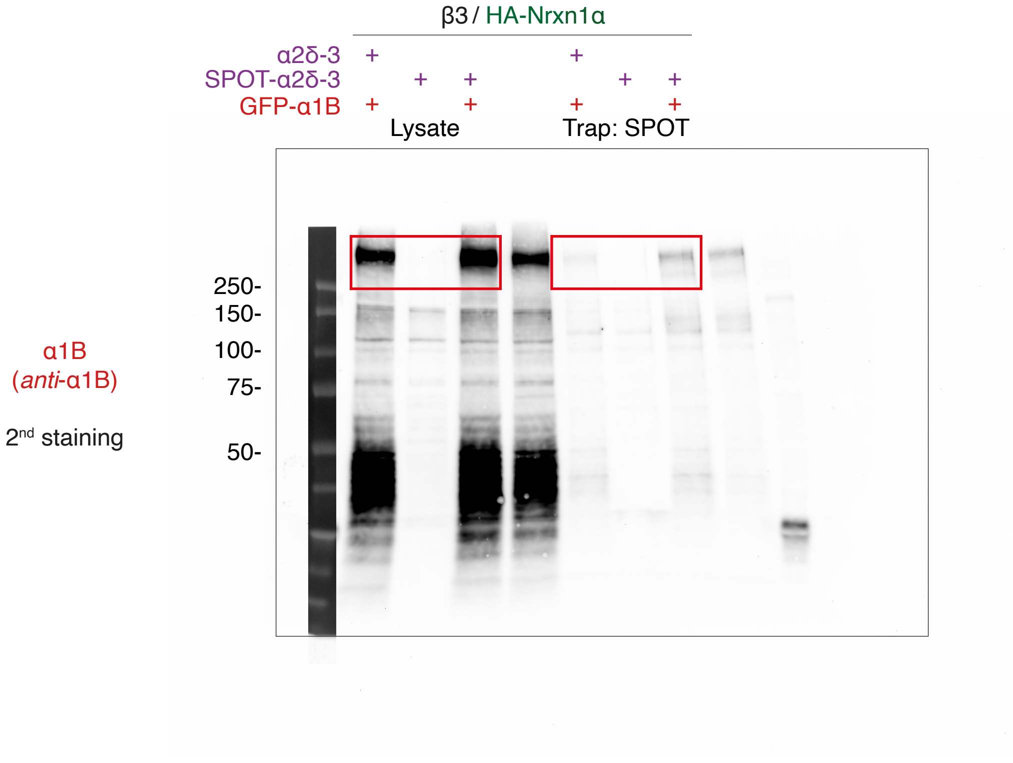

Figure 2E, panel 3, filename: 2021-05-08 10.43.42,5-3. scan_raw.png

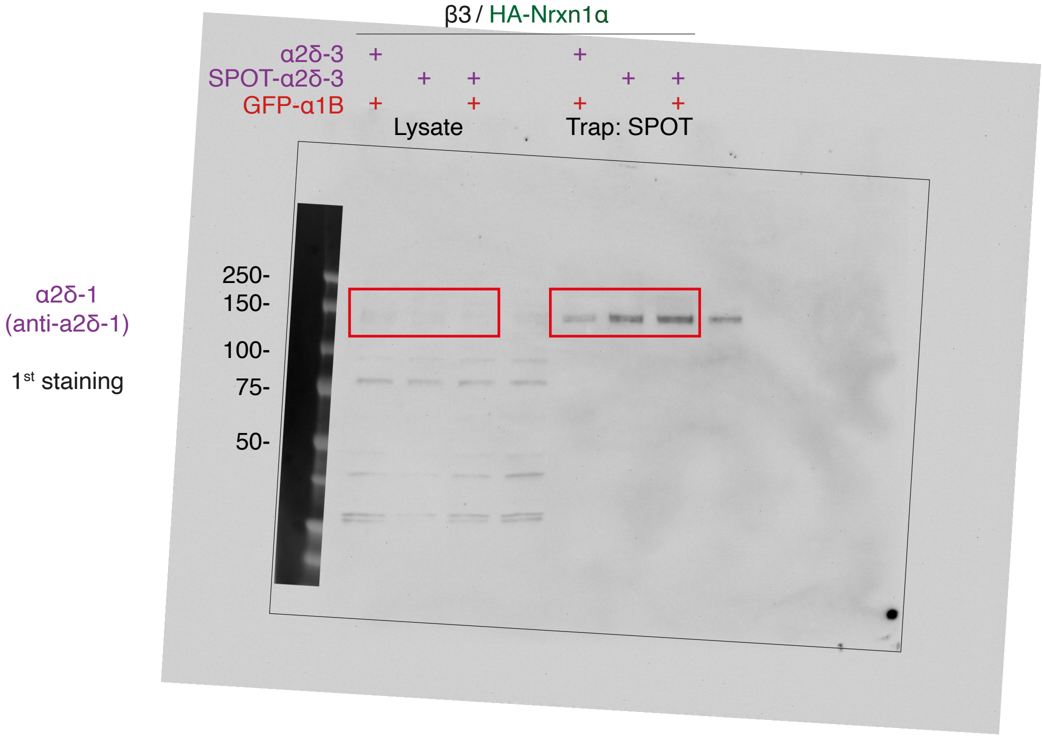

Figure 3B

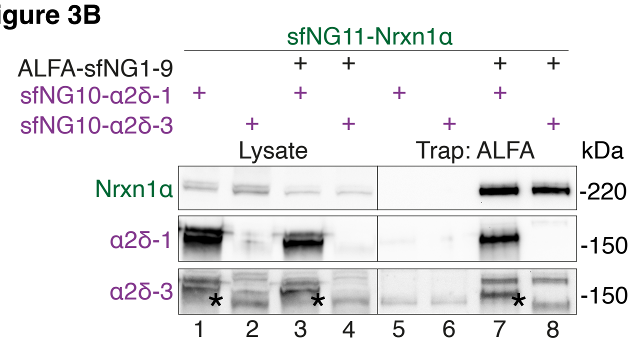

* = band from previous *anti*-α2δ-1 staining

Figure 3B, panel 1, filename: 2024-08-14 10.28.47,3-2. scan_raw.png

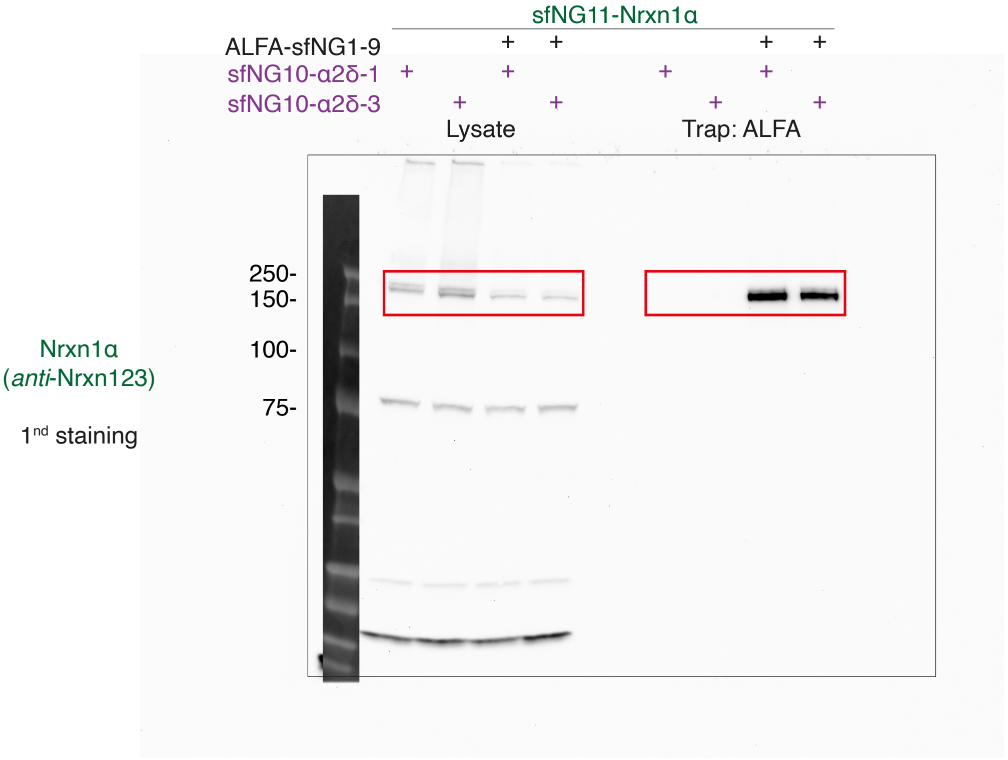

Figure 3B, panel 2, filename: 2024-08-15 11.09.50,3-2. scan_raw.png

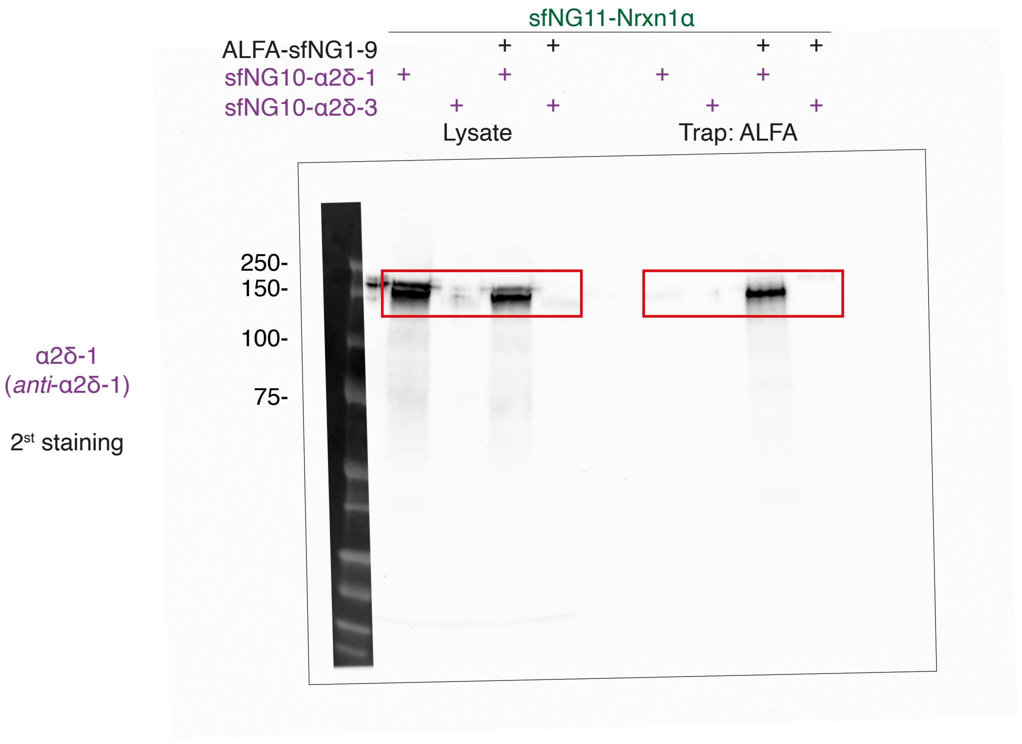

Figure 3B, panel 3, filename: 2024-09-06 11.58.52,3-2. scan_raw.png

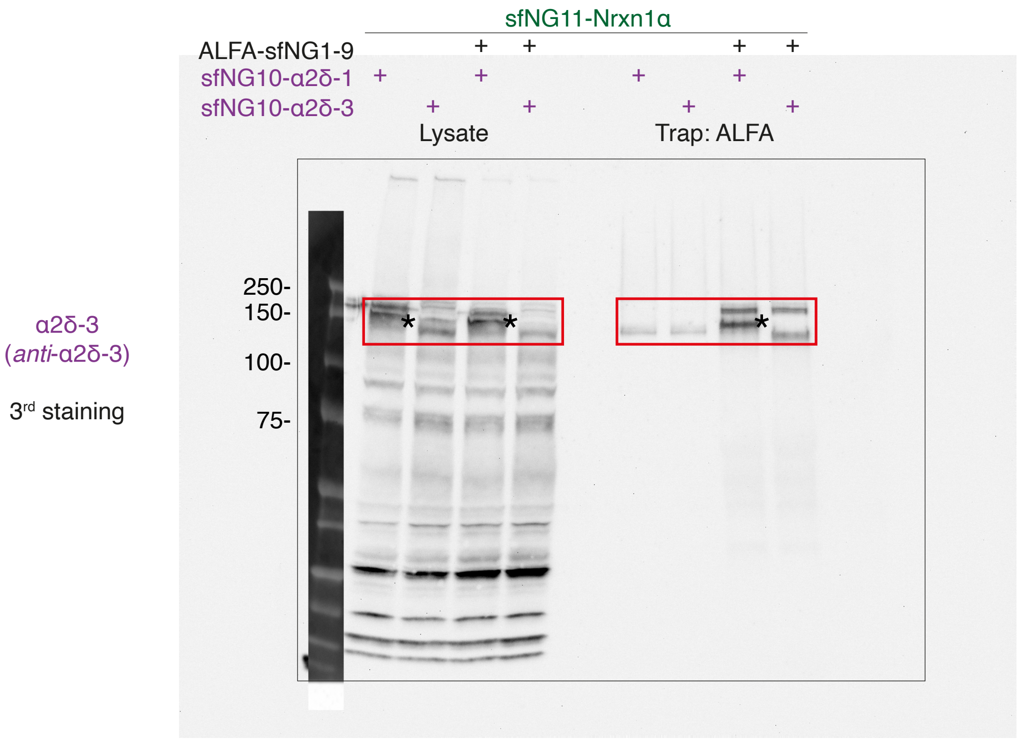

* = band from previous *anti*-α2δ-1 staining

Figure 4B

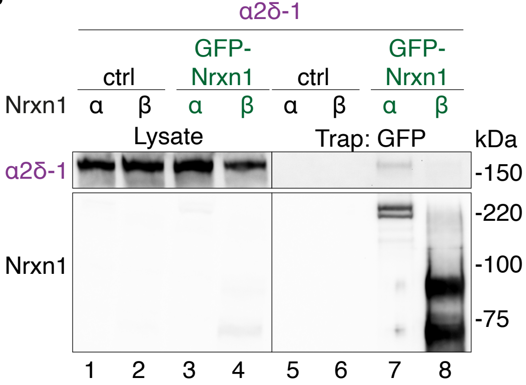

Figure 4B, panel 1, filename: 2024-07-04 10.43.46,5-3. scan_raw.png

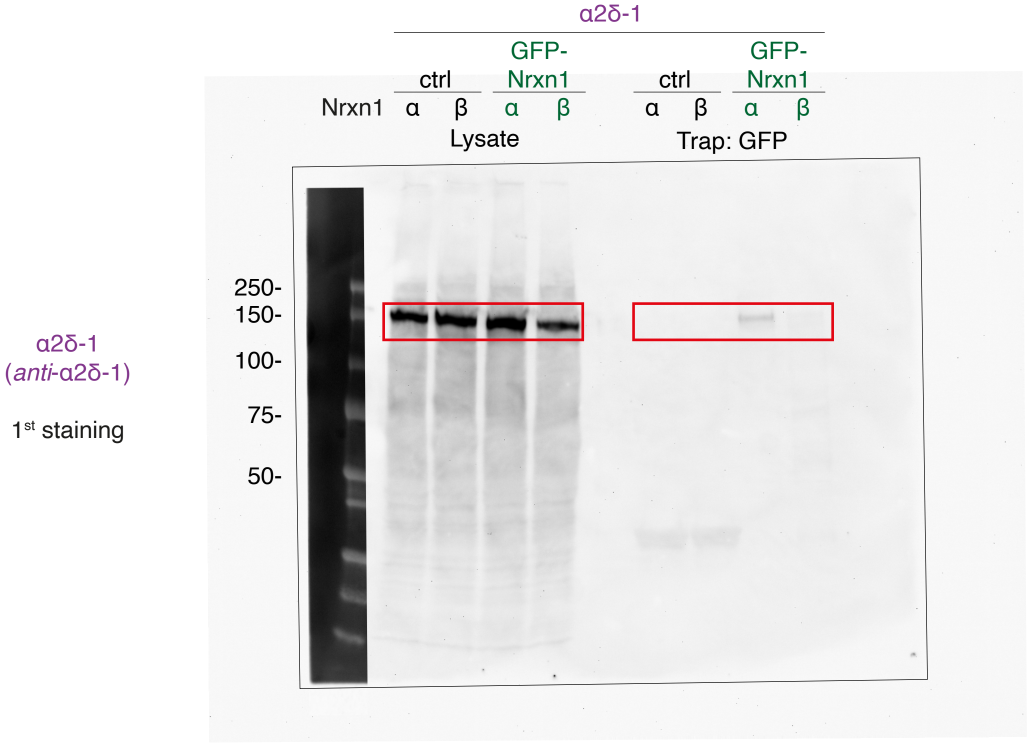

Figure 4B, panel 2, filename: 2024-07-09 10.17.16,1-1. scan_raw.png

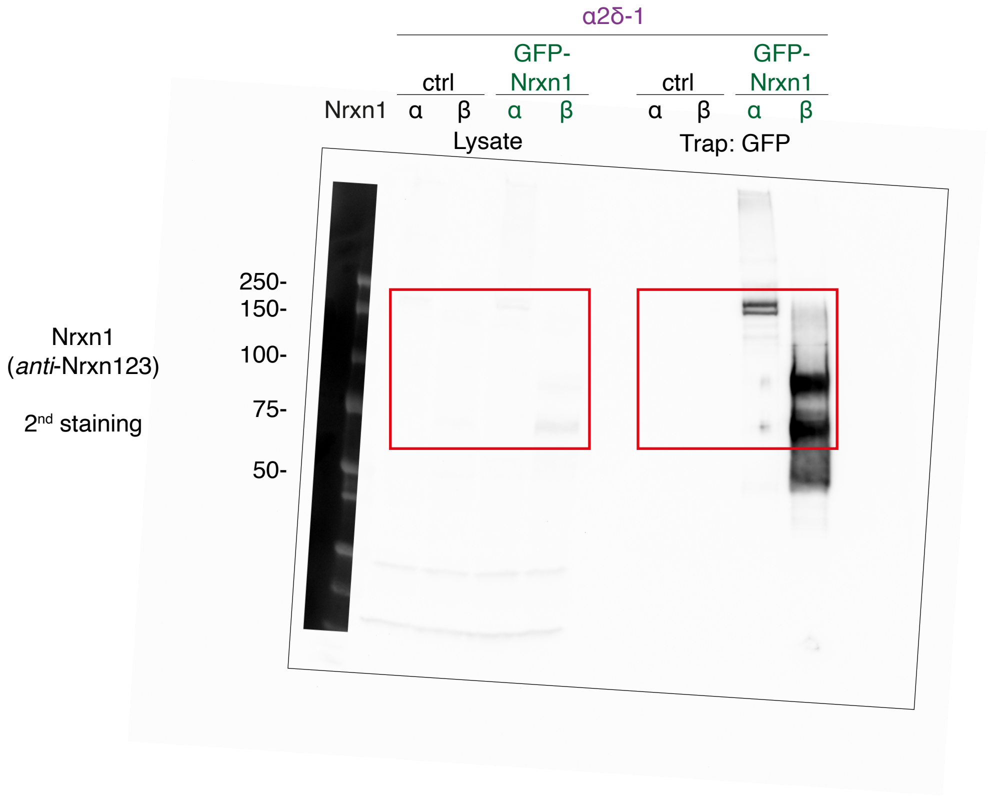

Figure 4C

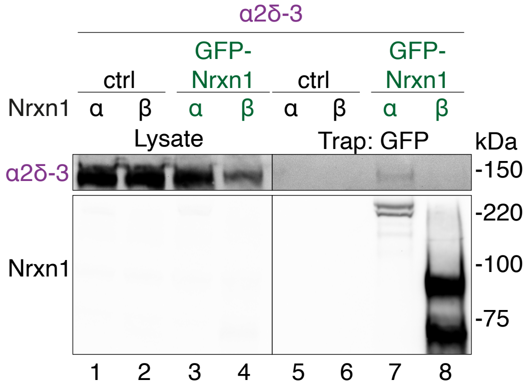

Figure 4C, panel 1 Lysate, filename: 2024-07-03 09.52.37,5-3. scan_raw.png

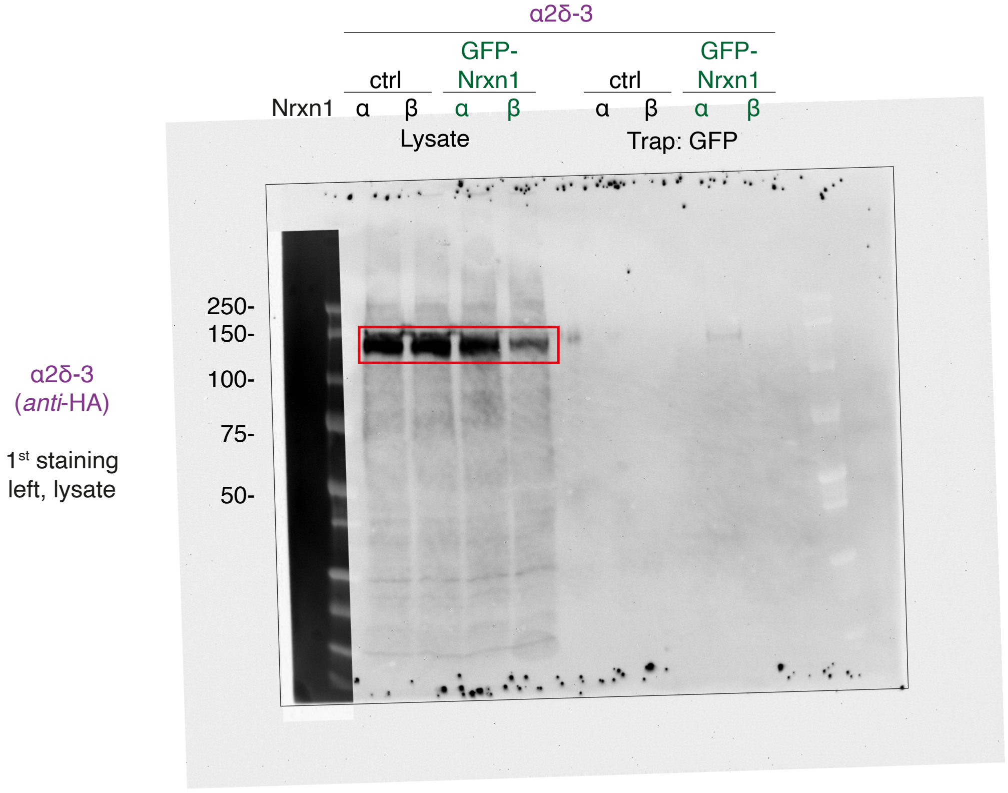

Figure 4C, panel 1 Trap, filename: 2024-07-04 09.52.37,5-3. scan_raw _pushed.png

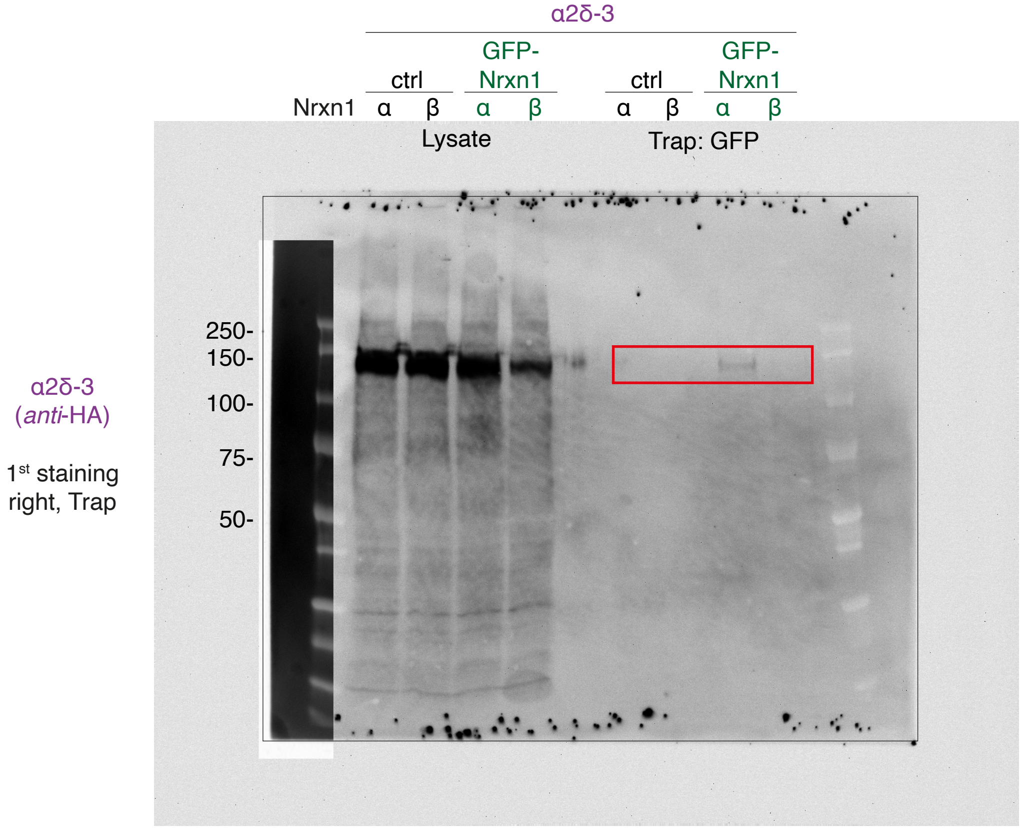

Figure 4C, panel 2, filename: 2024-07-04 11.01.59,3-2. scan_raw.png

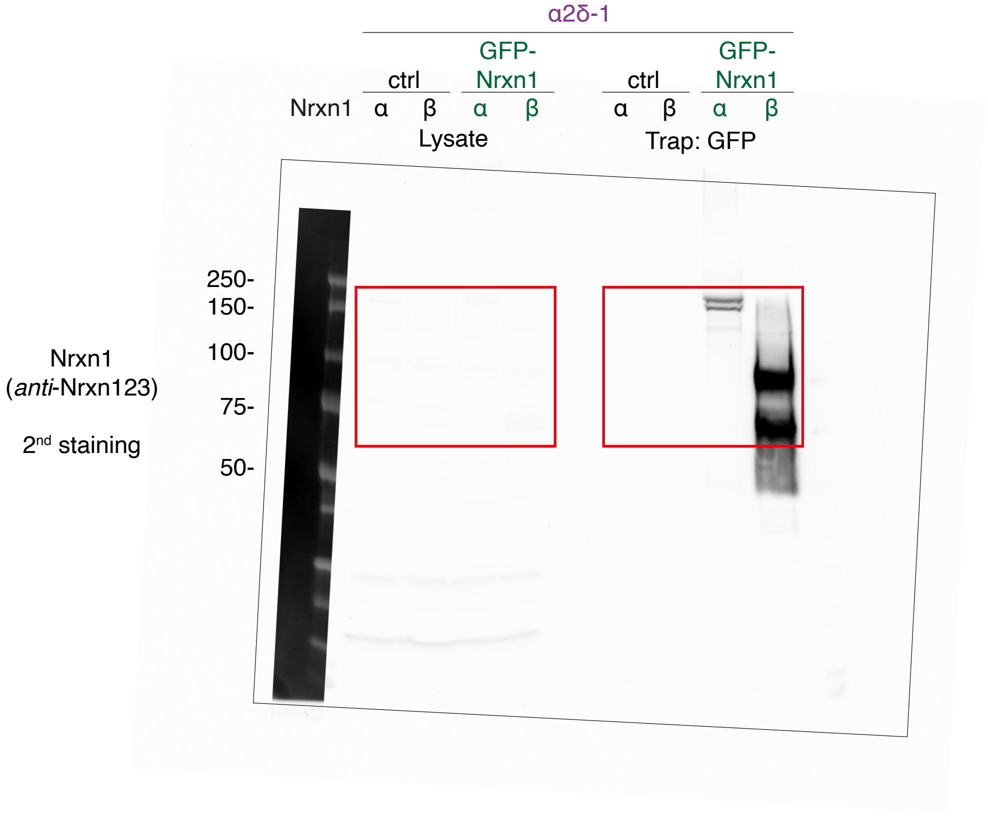

Figure 4D

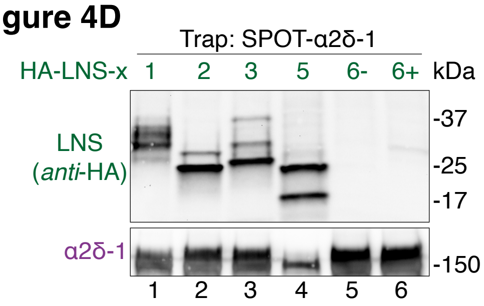

Figure 4D, panel 1, filename: 2024-06-04 10.19.32,3-2. scan_raw.png

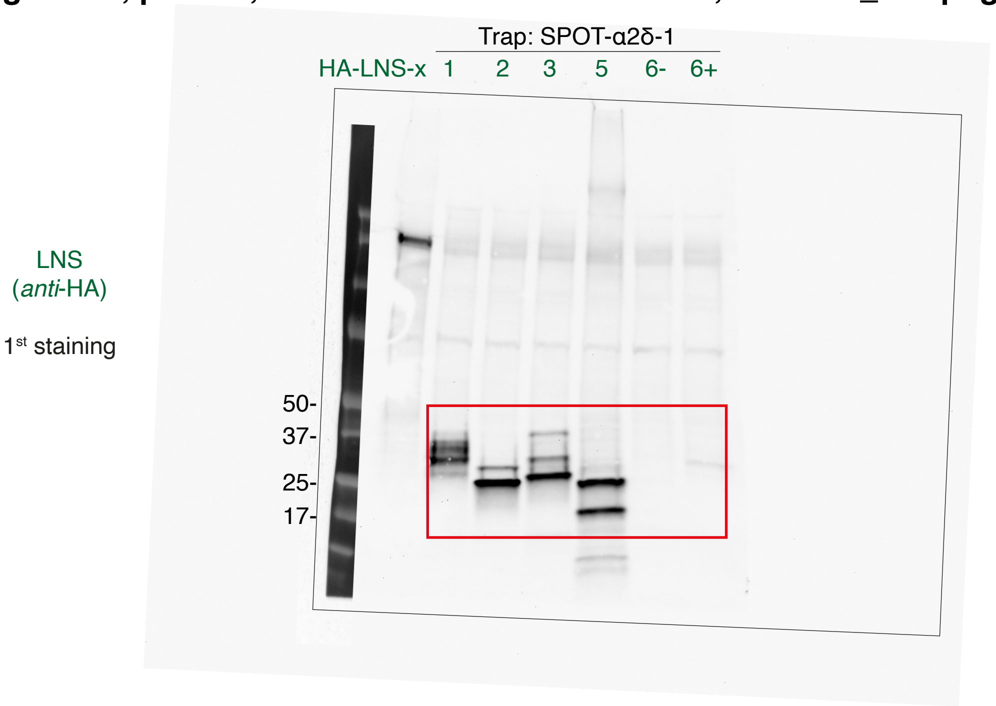

Figure 4D, panel 2, filename: 2024-06-05 11.02.05,1-1. scan_raw.png

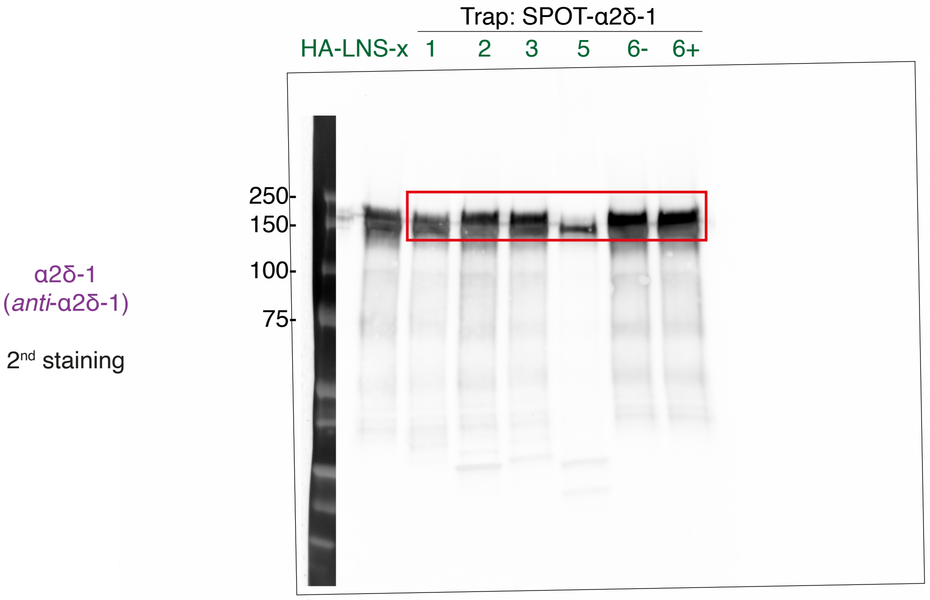

Figure 4E

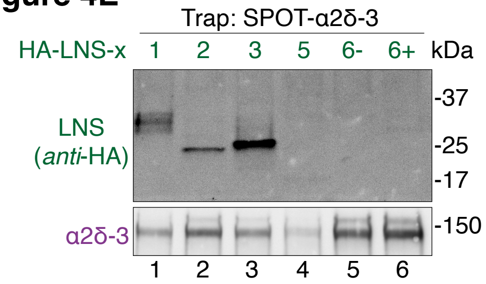

Figure 4E, panel 1, filename: 2024-08-01 11.27.21,5-3. scan_raw.png

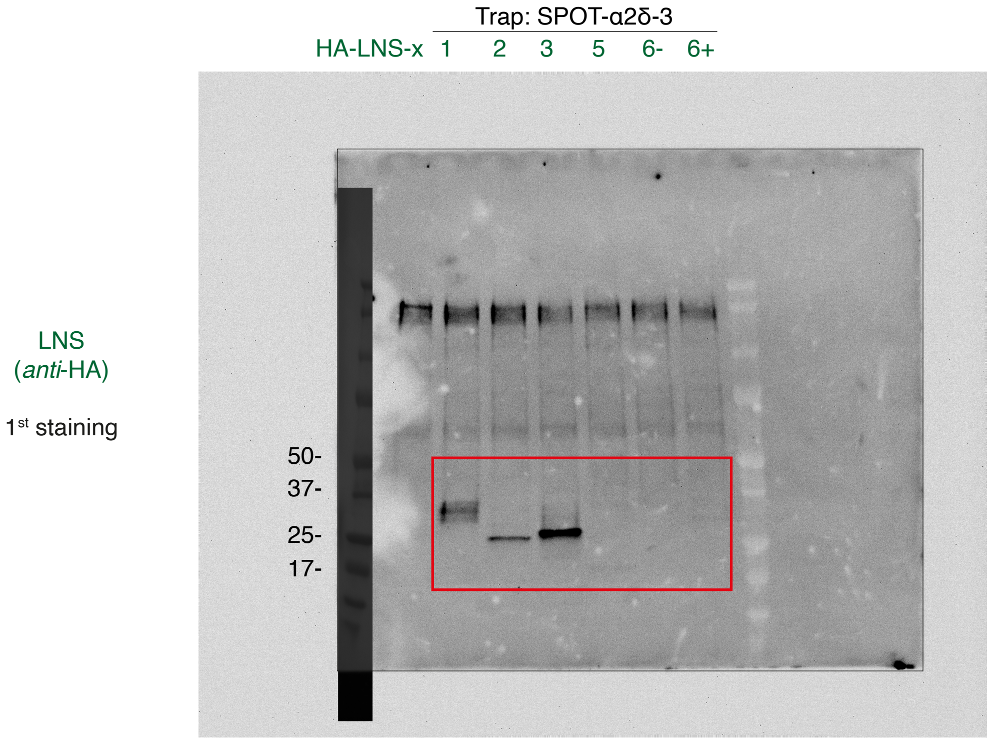

Figure 4E, panel 2, filename: 2024-08-02 09.54.27,4-2. scan+Marker_raw.png

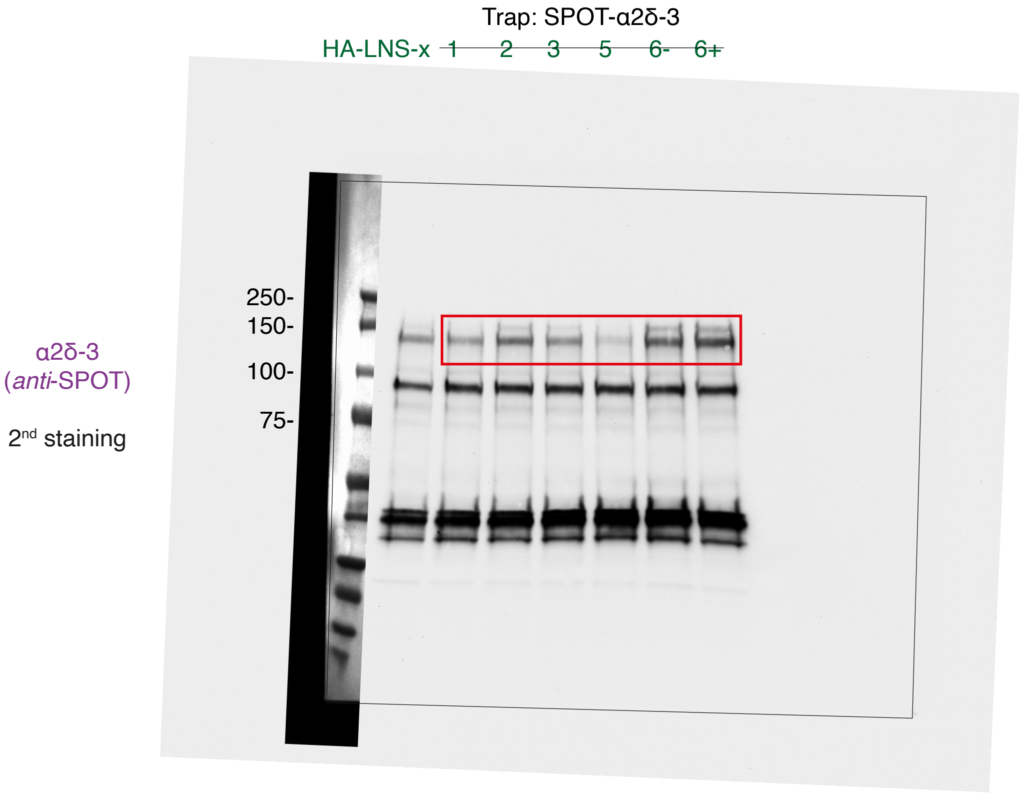

Figure 5C

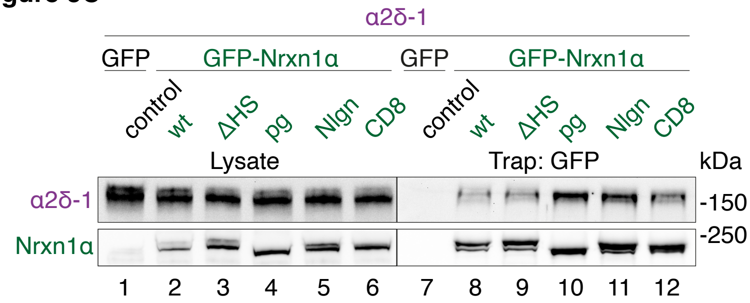

Figure 5C, panel 1 Lysate, filename: 2023-08-30 10.42.22,1-1. scan_raw.png

α2δ-1
(*anti*-α2δ-1)

1st staining, Lysate

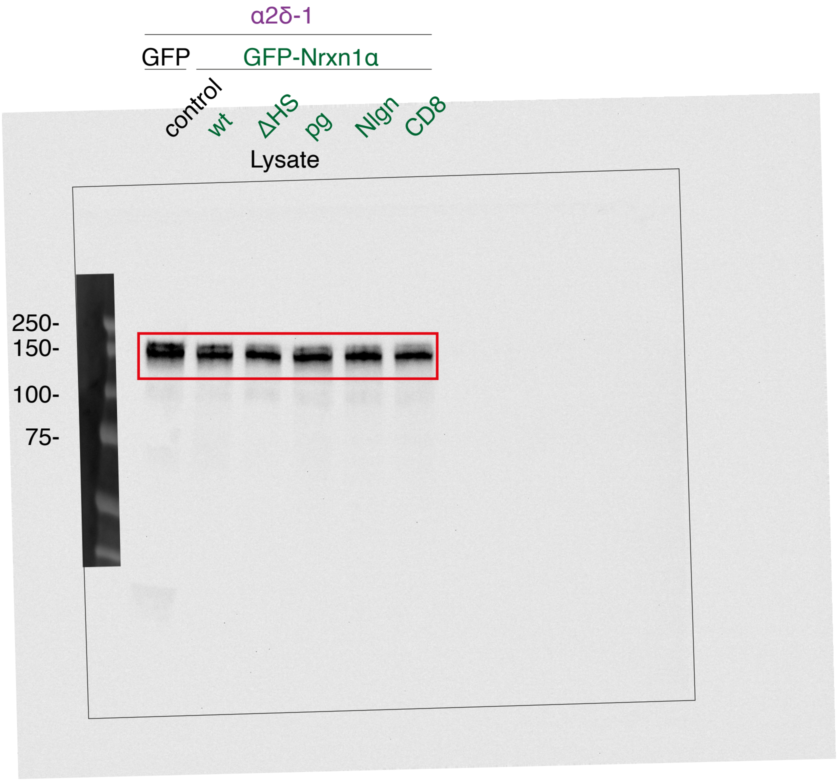

Figure 5C, panel 1 Trap, filename: 2023-08-30 10.57.32,5-3. scan_raw.png

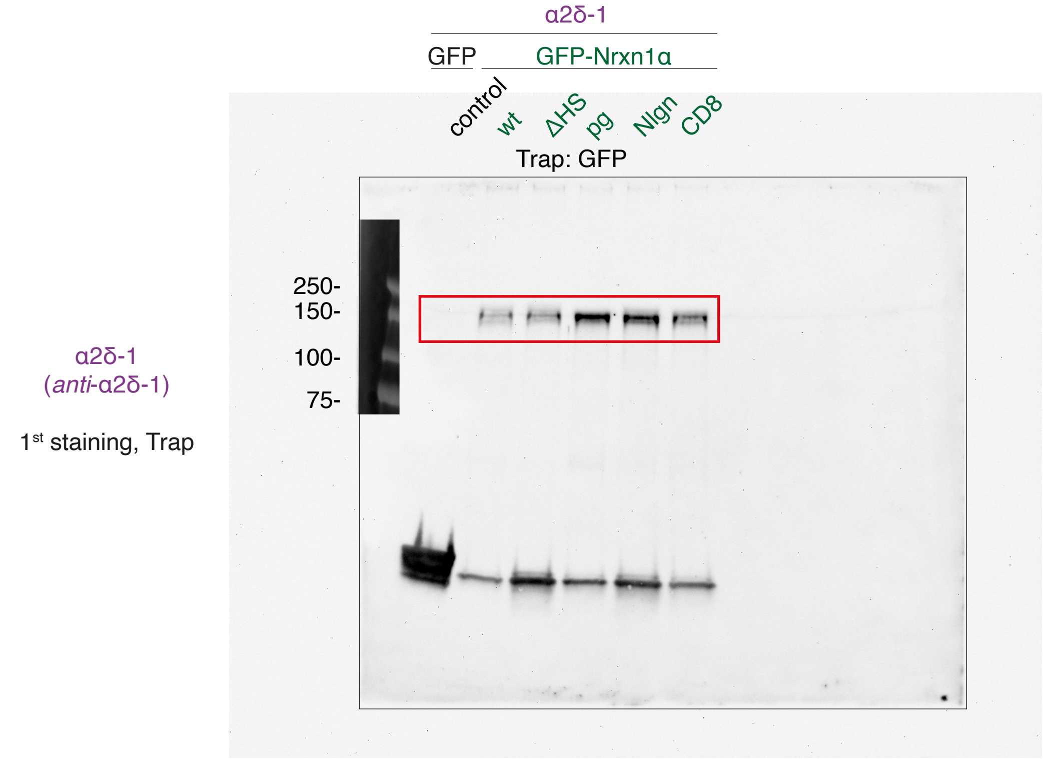

Figure 5C, panel 2 Lysate, filename: 2023-08-31 10.24.16,3-2. scan_raw.png

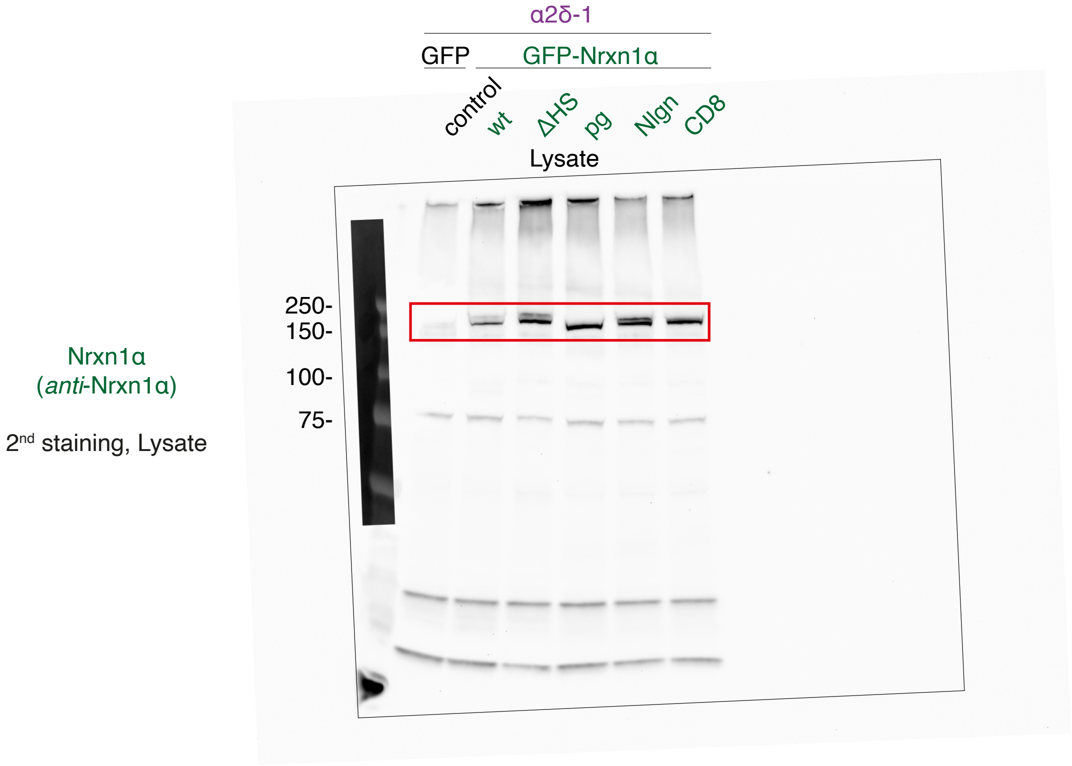

Figure 5C, panel 2 Trap, filename: 2023-08-31 10.24.16,3-2. scan_raw.png

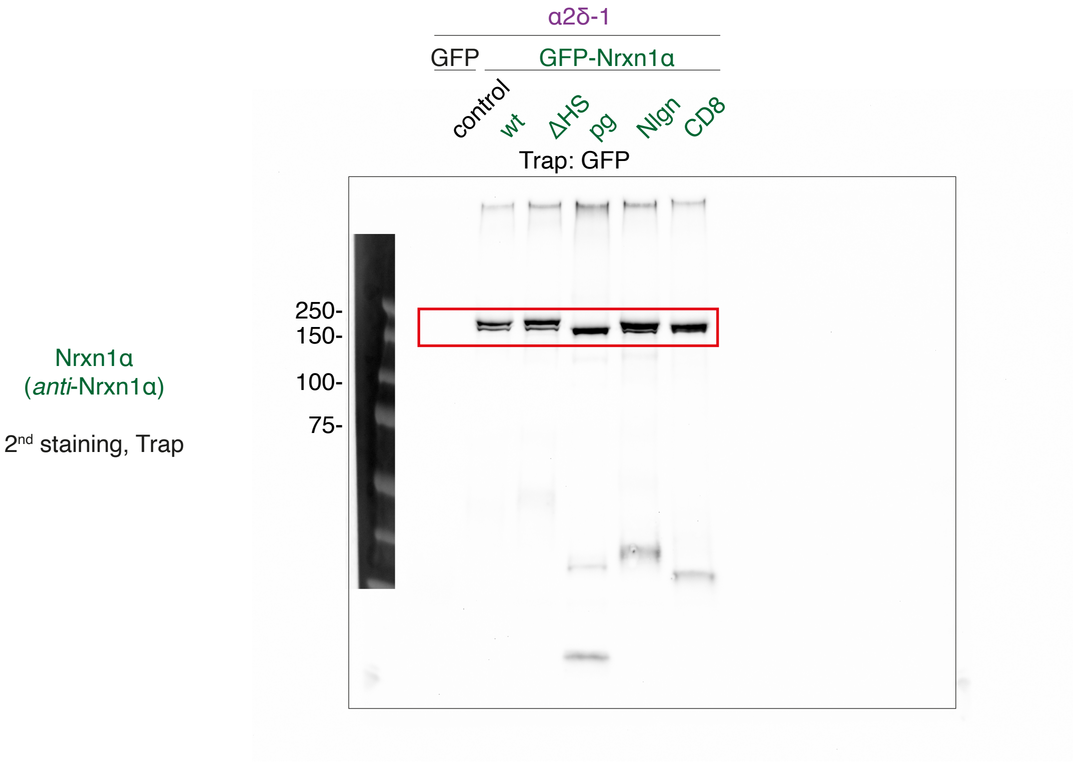

Figure 5D

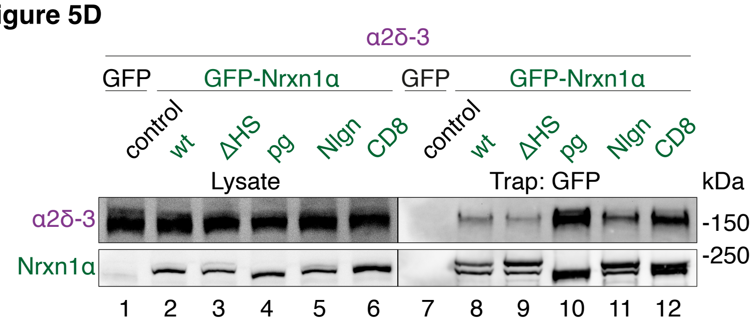

Figure 5D, panel 1 Lysate, filename: 2024-07-24 10.45.47,3-2. scan_raw.png

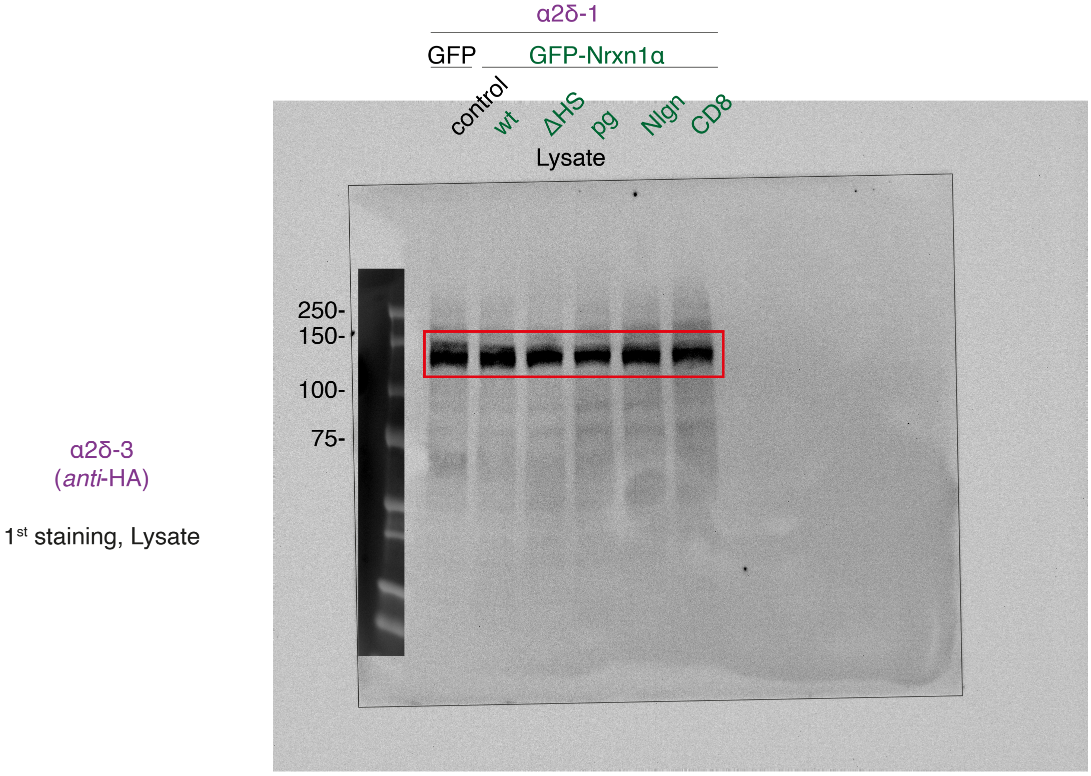

Figure 5D, panel 1 Trap, filename: 2024-07-25 10.17.11,5-3. scan_raw.png

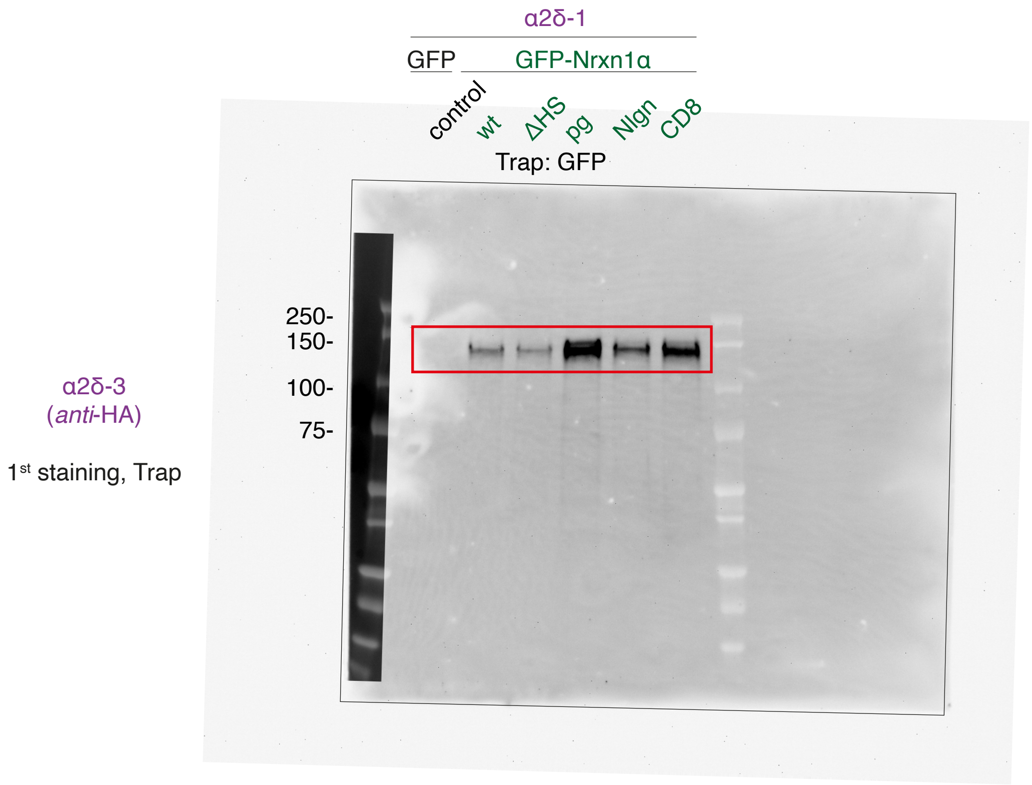

Figure 5D, panel 2 Lysate, filename: 2024-07-25 10.51.17,3-2. scan_raw.png

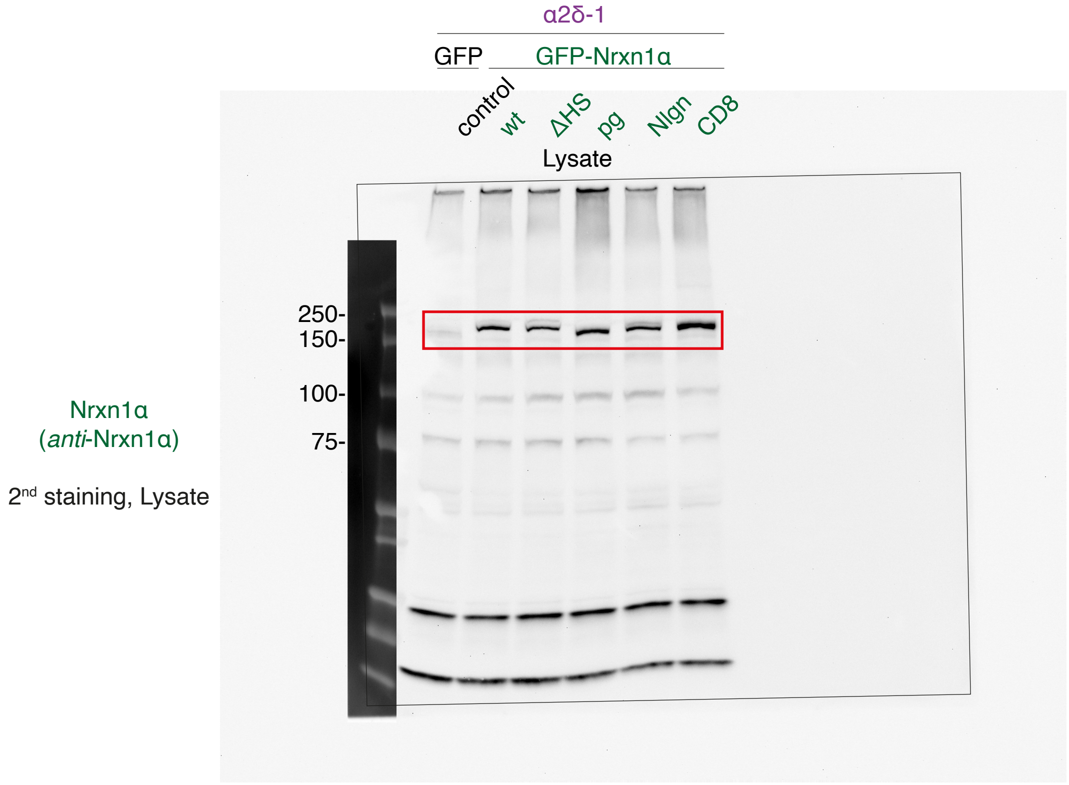

Figure 5D, panel 2 Trap, filename: 2024-07-26 09.54,3-2. scan_raw.png

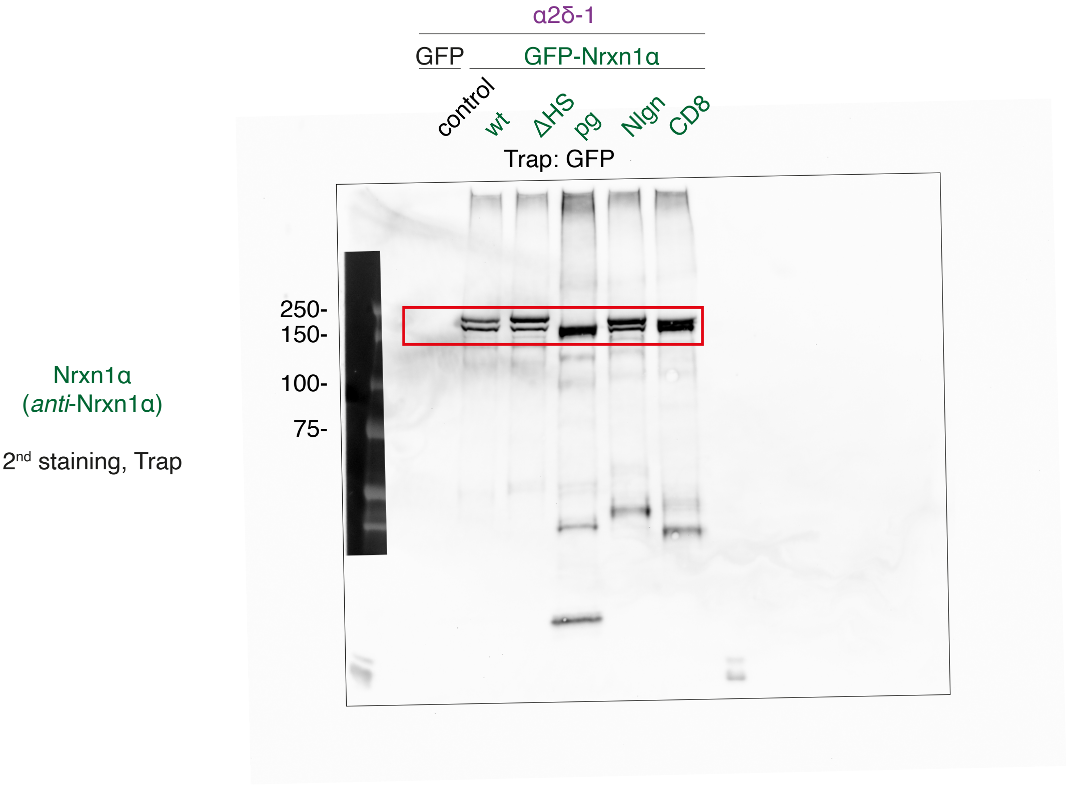

Figure 6B

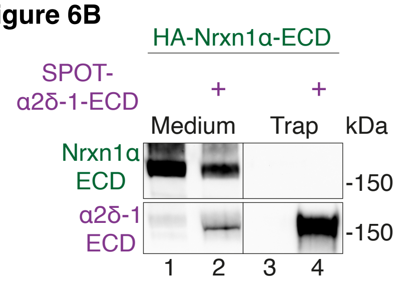

Figure 6B, panel 1, filename: 2024-11-27 11.28.51,5-3. scan_raw.png

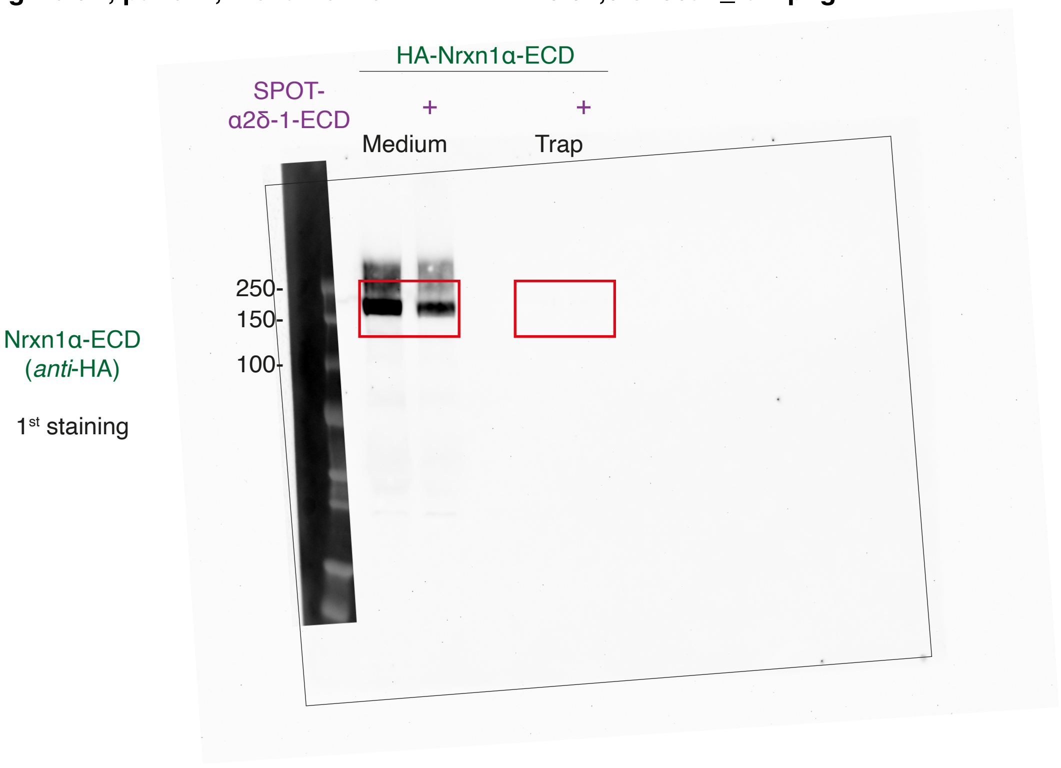

Figure 6B, panel 2, filename: 2024-11-28 10.38.49,1-1. scan_raw.png

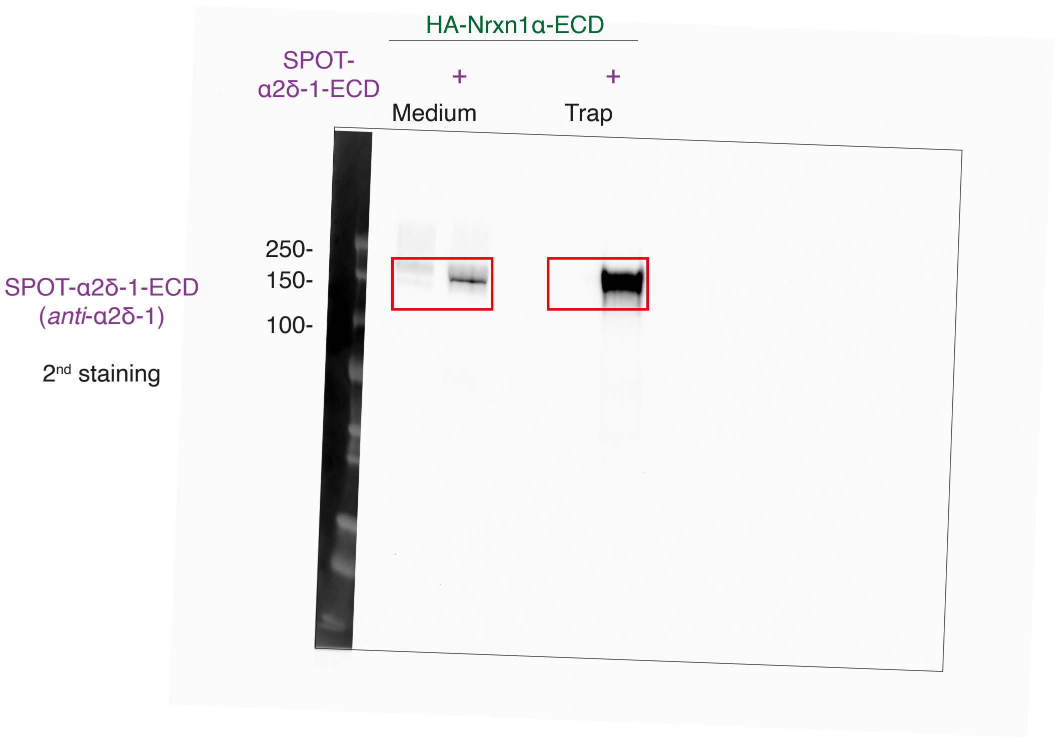

Figure 6C

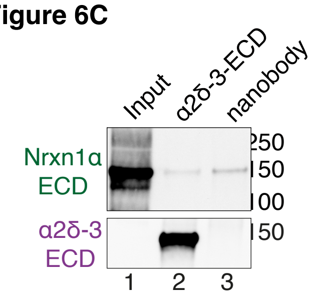

Figure 6C, panel 1, filename: 2021-11-09 12.11.37,3-2. scan_raw.png

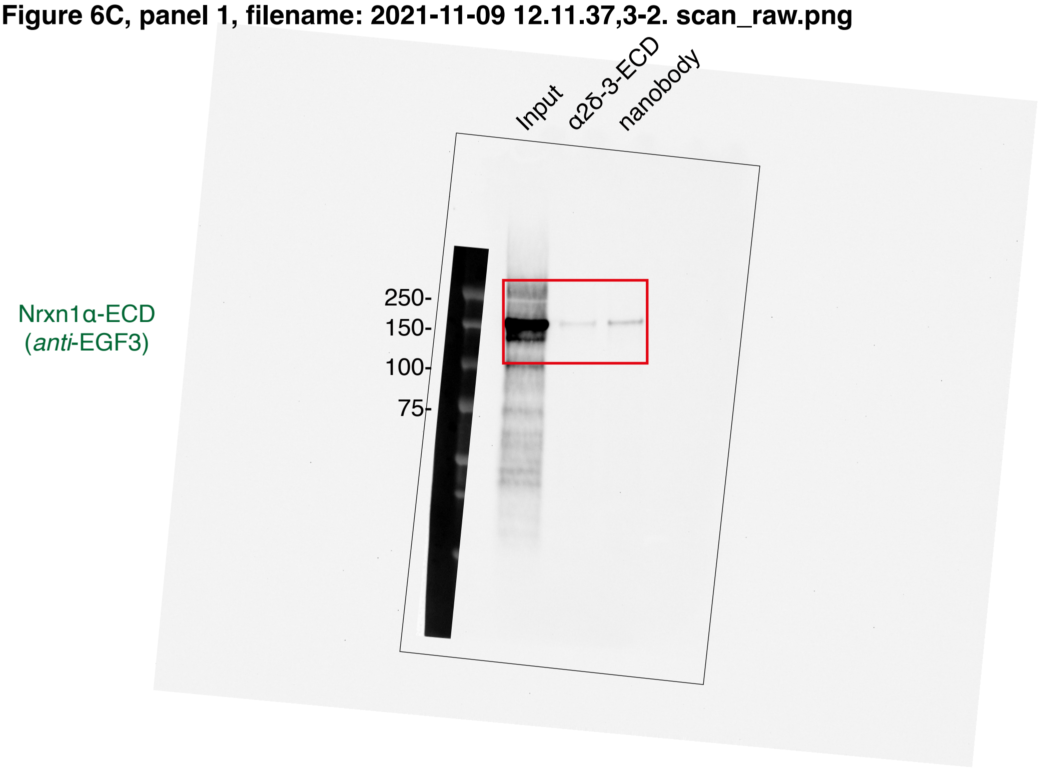

Figure 6C, panel 2, filename: 2021-11-09 11.55.39,3-2. scan_raw.png

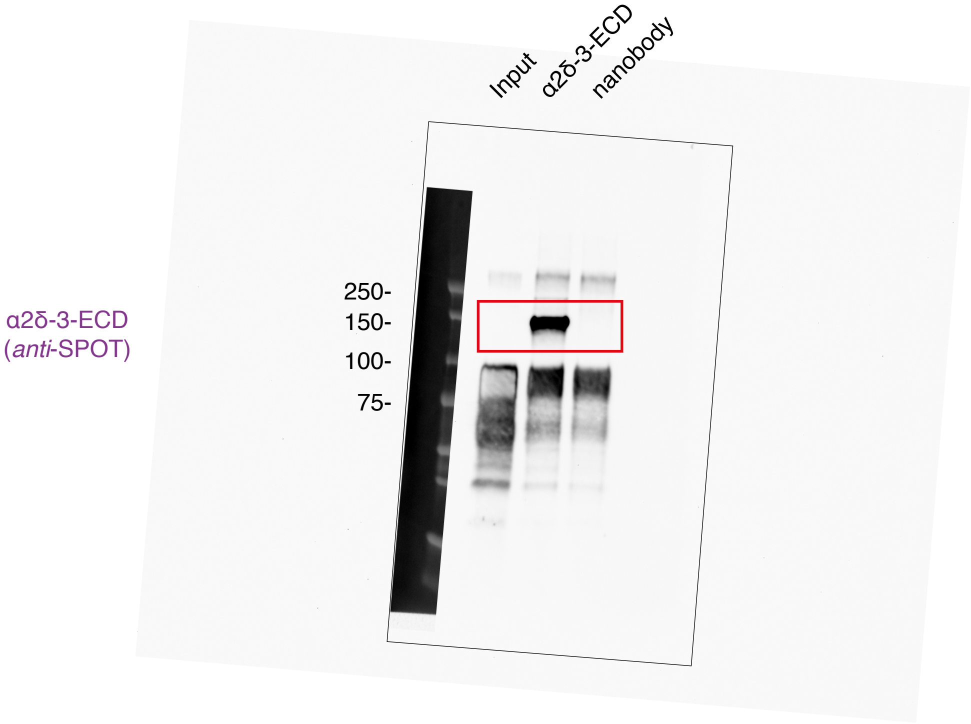

Figure 6D

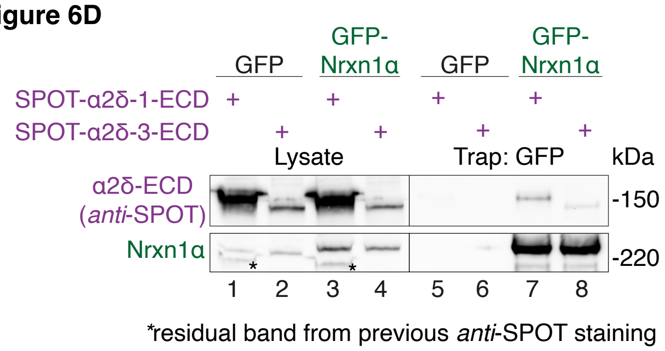

Figure 6D, panel 1, filename: 2023-12-01 11.11.08,5-3. scan_raw.png

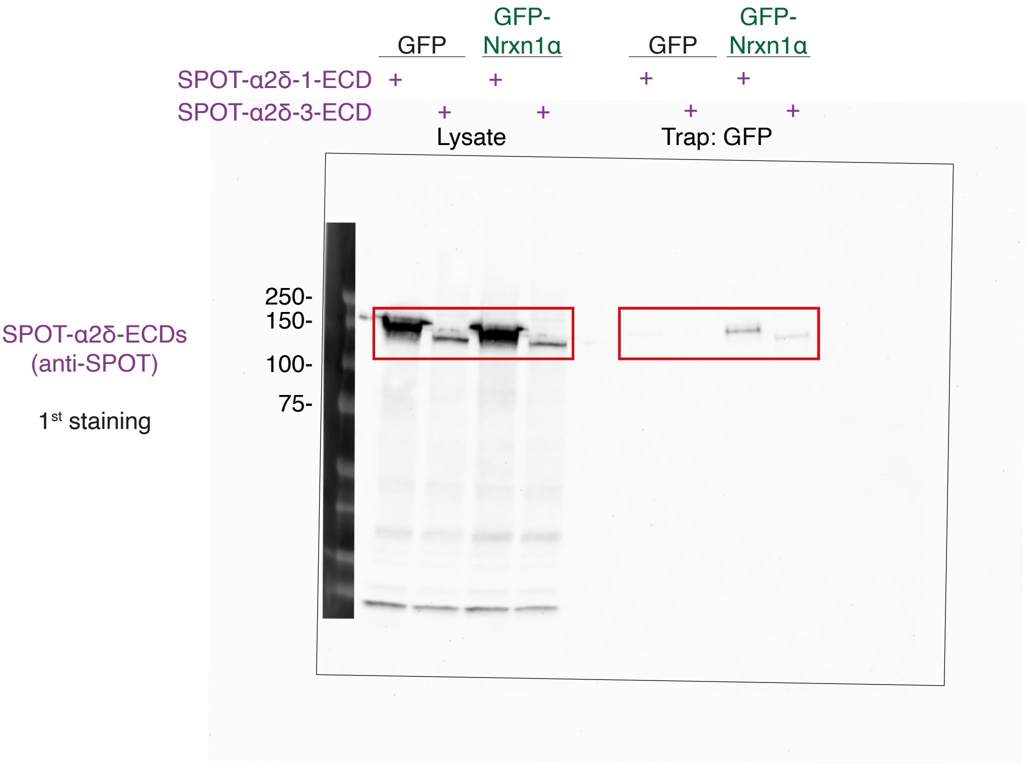

Figure 6D, panel 2, filename: 2023-12-05 11.51.30,4-2. scan+Marker_raw.png

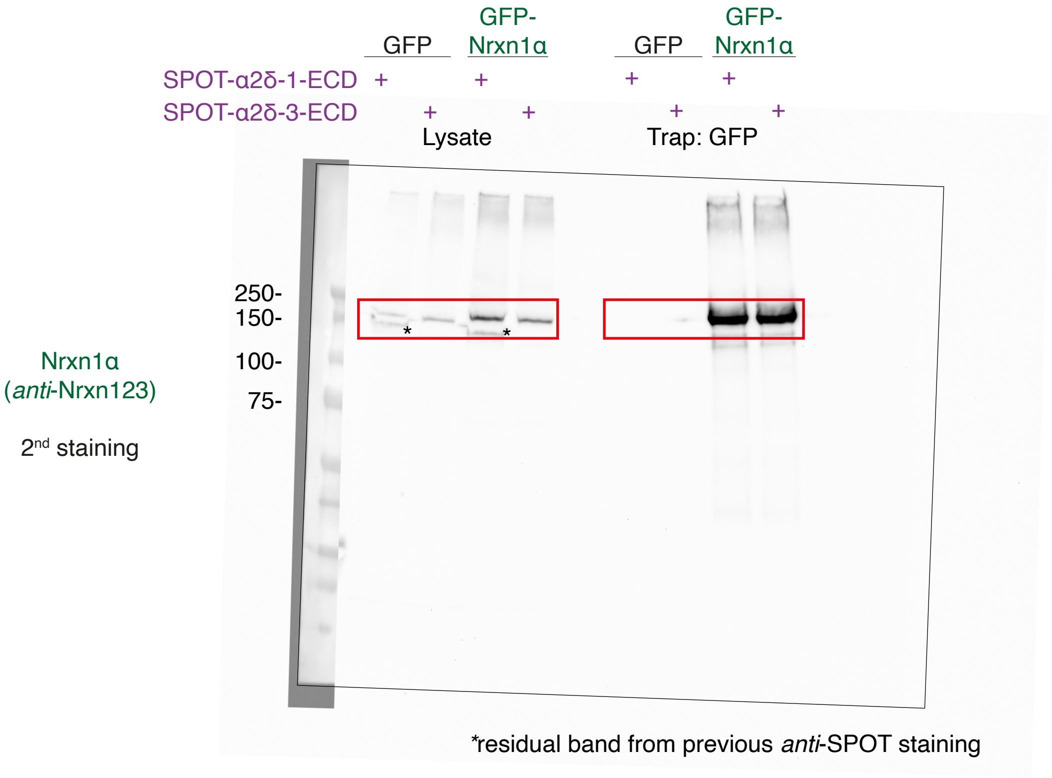

*residual band from previous *anti*-SPOT staining

Figure 6F

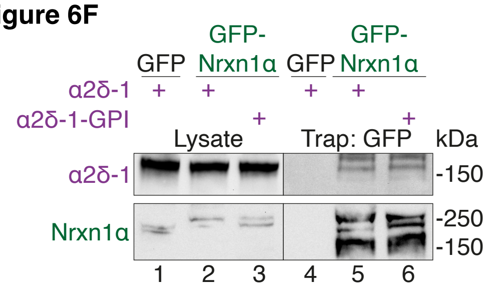

Figure 6F, panel 1 Lysate. filename: 2022-12-16 12.47.03,5-3. scan_raw.png

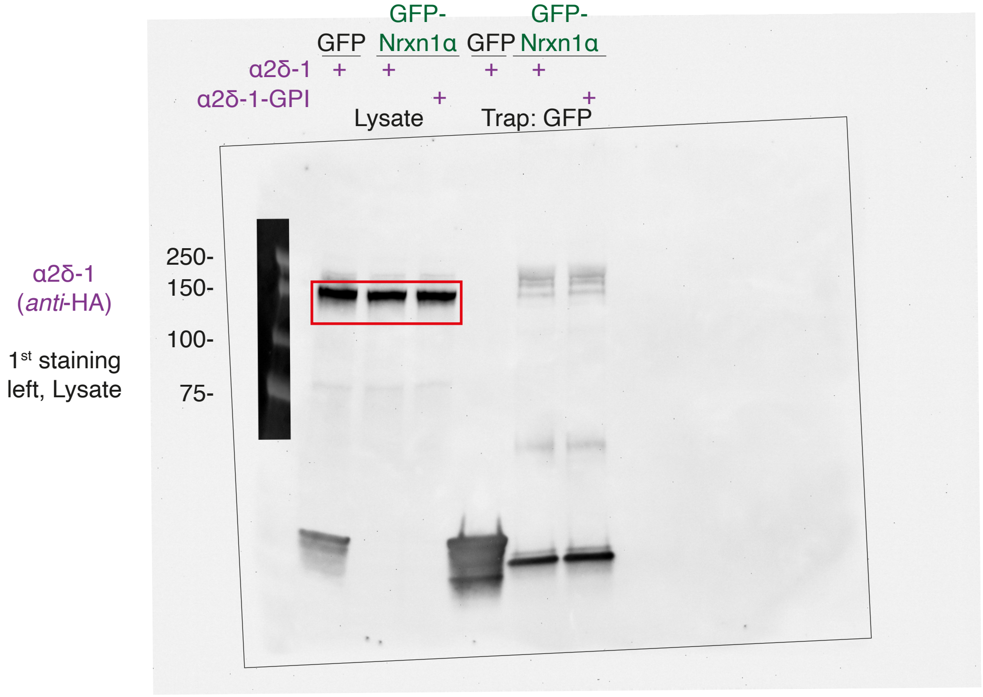

Figure 6F, panel 1 Trap, filename: 2022-12-16 12.47.03,5-3. scan_raw_pushed Kopie.png

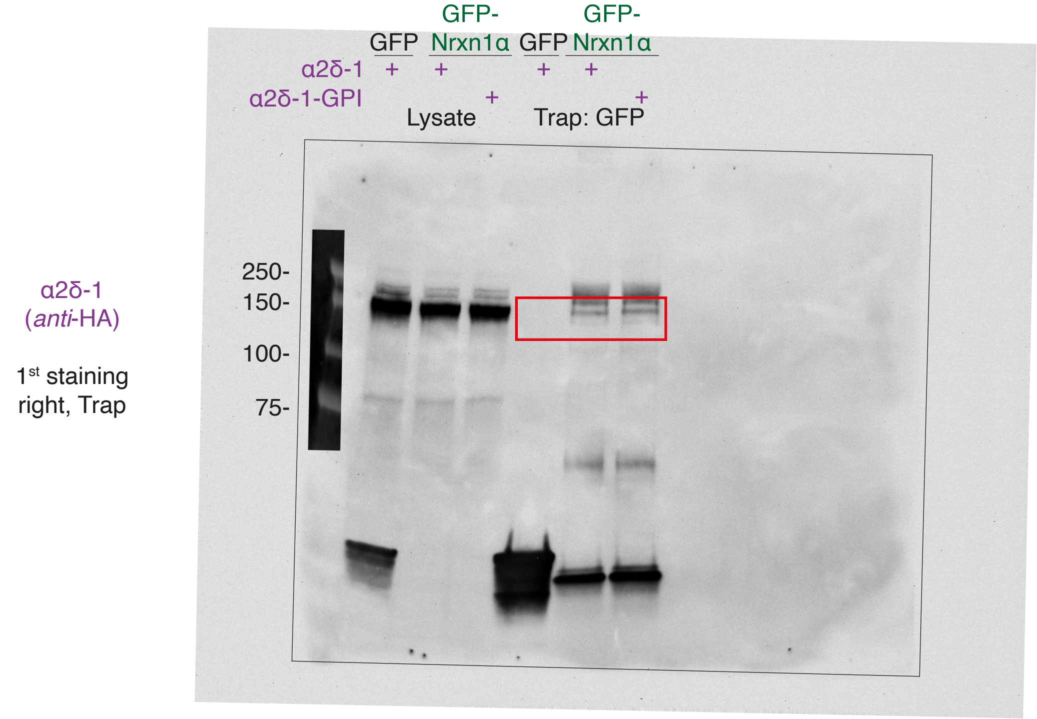

Figure 6F, panel 2, filename: 2023-01-03 11.37.11,3-2. scan_raw.png

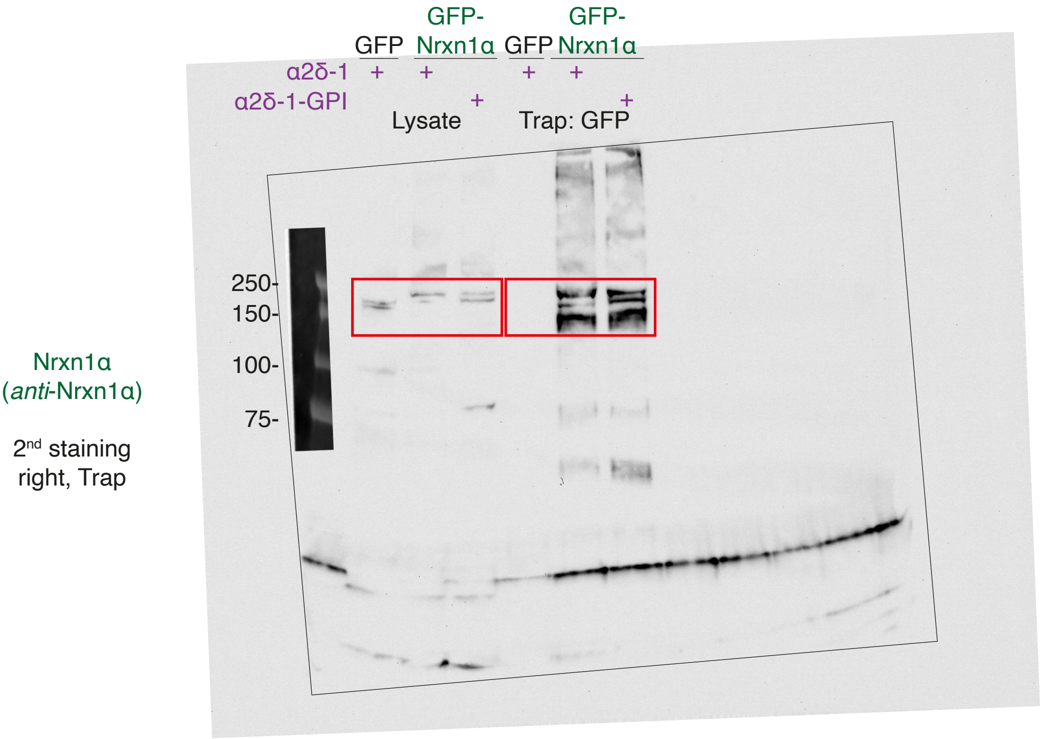

Figure 6G

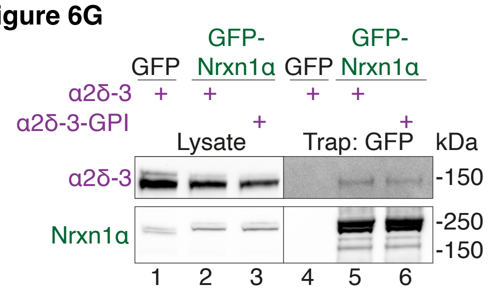

Figure 6G, panel 1 Lysate, filename: 2022-12-16 12.47.03,5-3. scan_raw.png

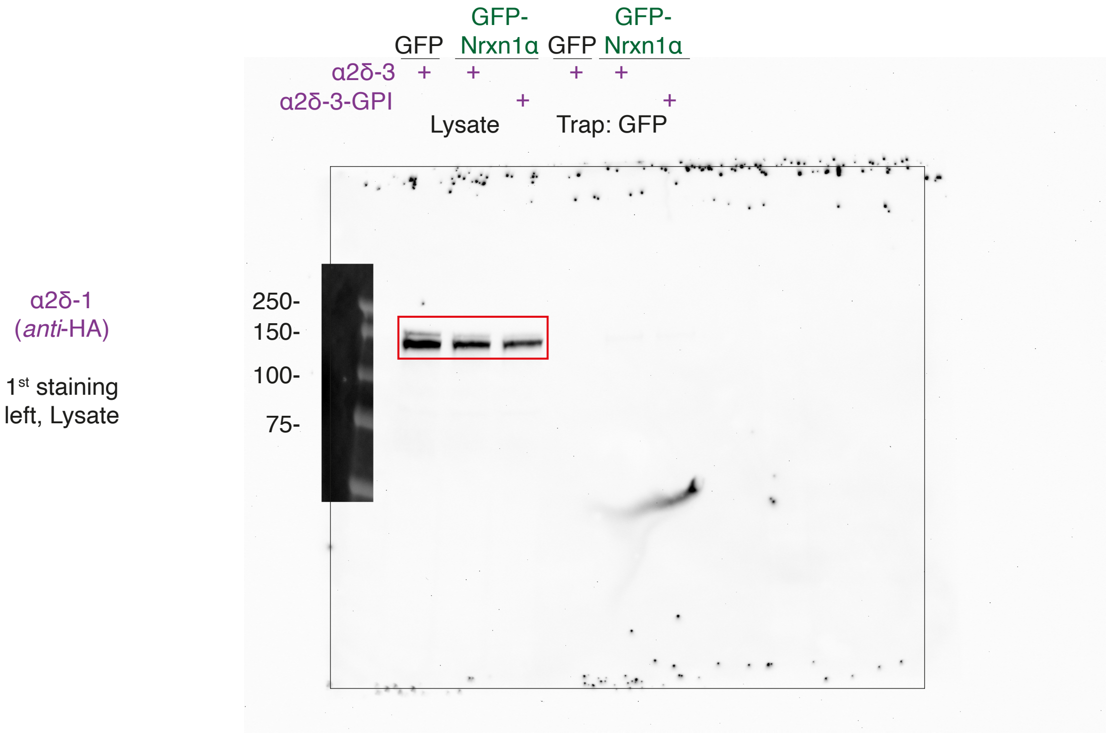

Figure 6G, panel 1 Trap, filename: 2022-12-15 13.09.04,5-3. scan_raw_pushed Kopie.png

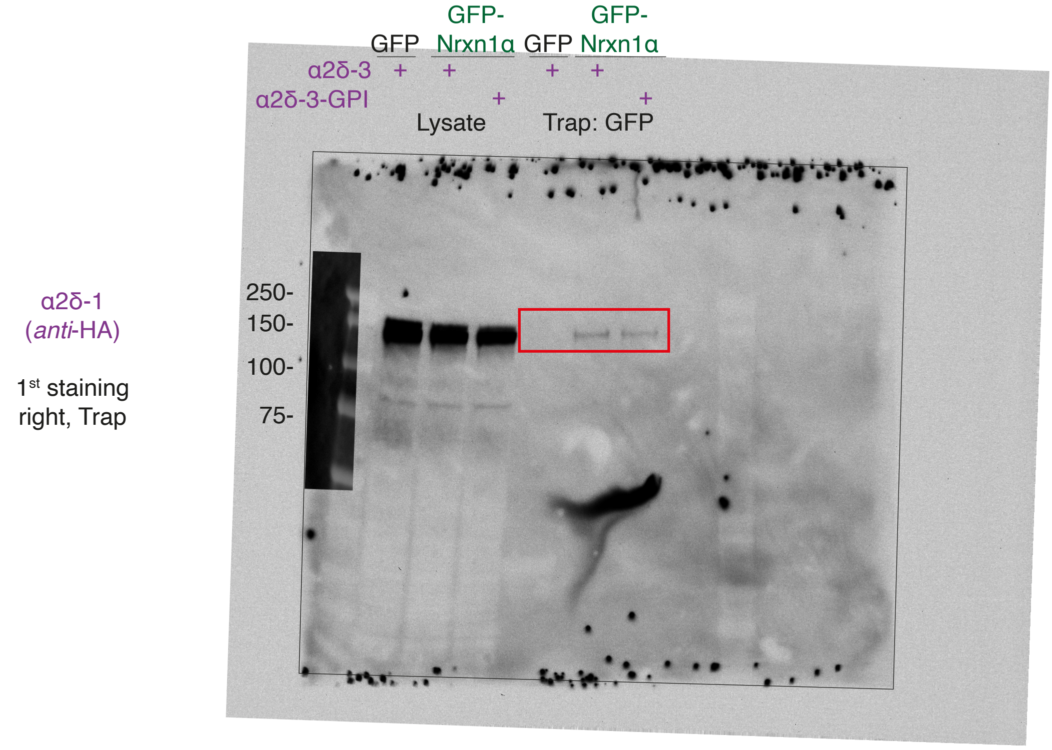

Figure 6G, panel 2, filename: 2022-12-16 13.03.09,3-2. scan_raw.png

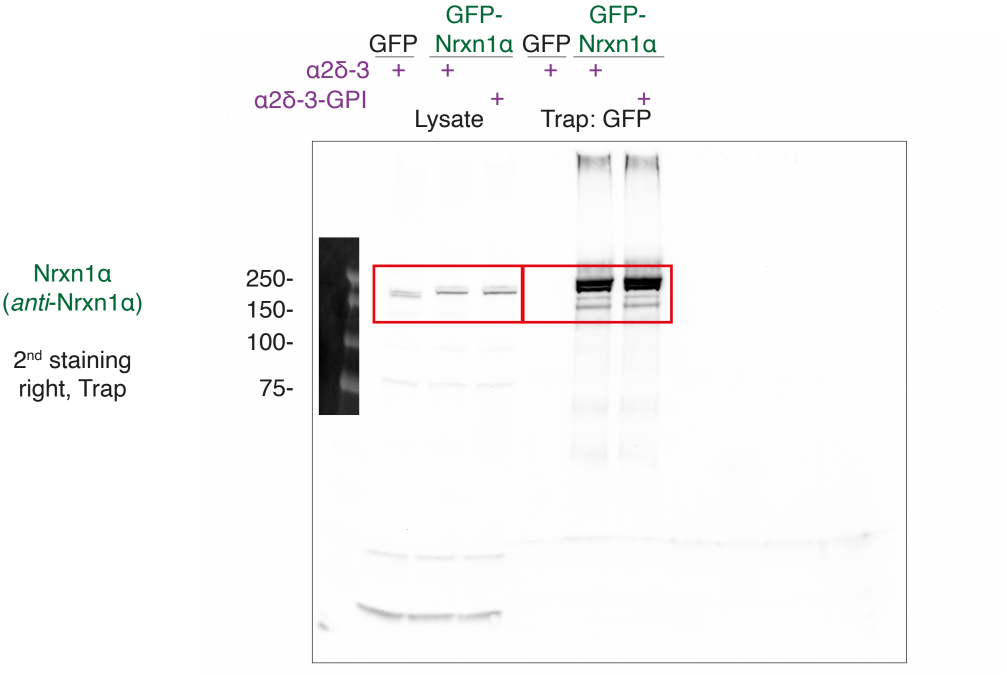

Figure 7B

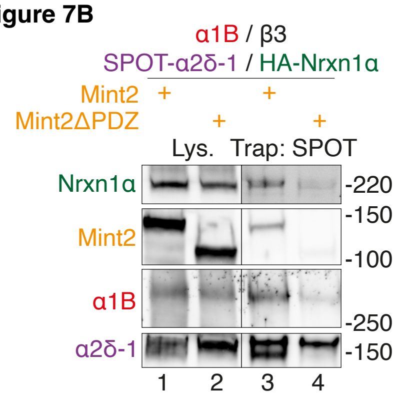

Figure 7B, panel 1 Lysate, filename: 2022-08-10 11.14.22,3-2. scan_raw.png

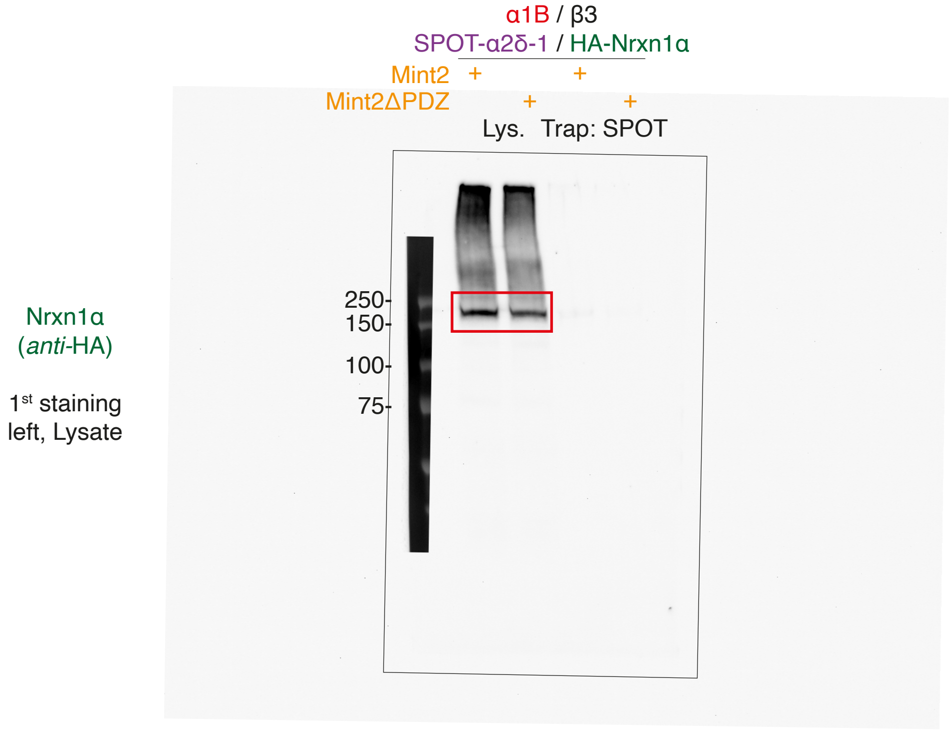

Figure 7B, panel 1 Trap, filename: 2022-08-10 11.14.22,5,3-. scan_raw Kopie.png

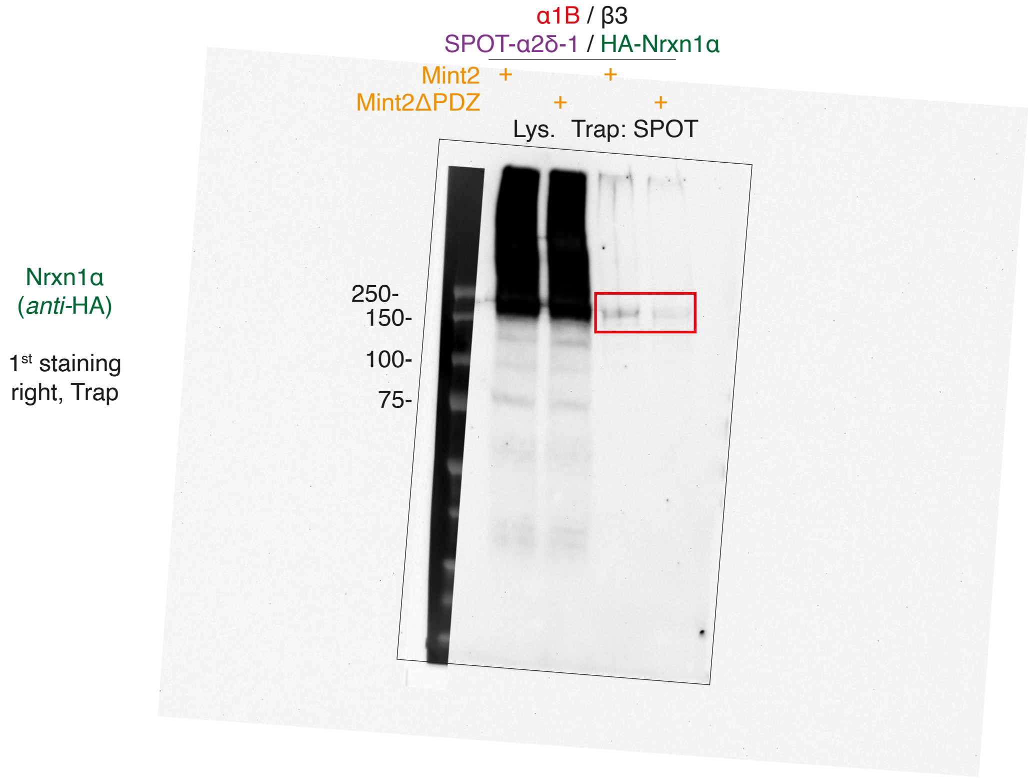

Figure 7B, panel 4, filename: 2022-08-12 10.55.44,3-2. scan_raw.png

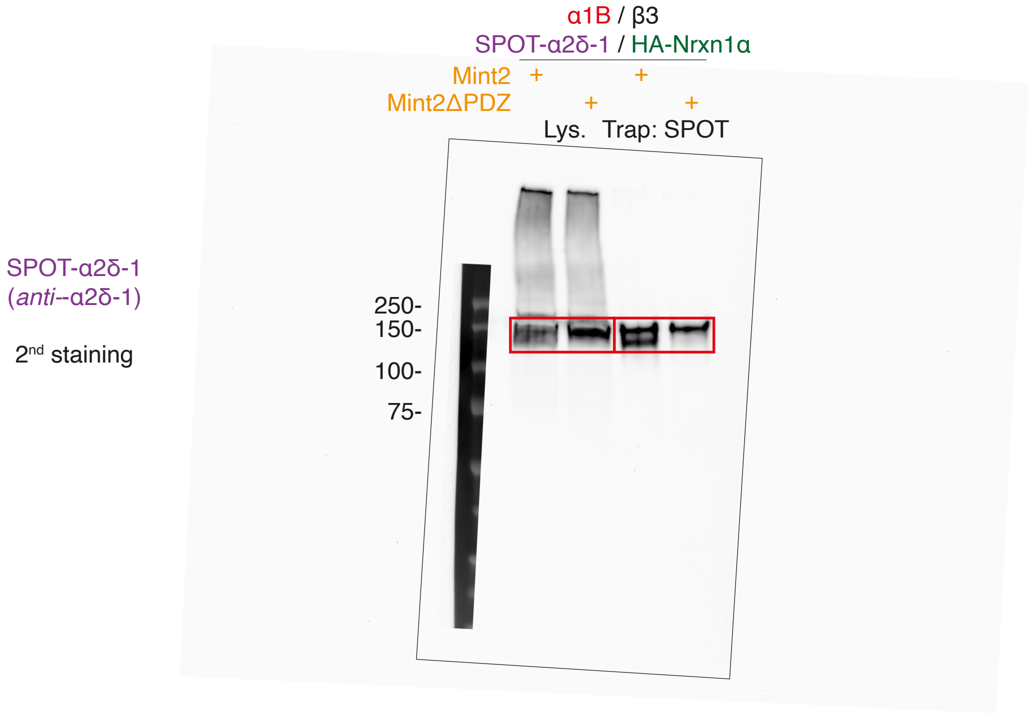

Figure 7B, panel 2, filename: 2022-08-16 11.16.24,1-1. scan_raw.png

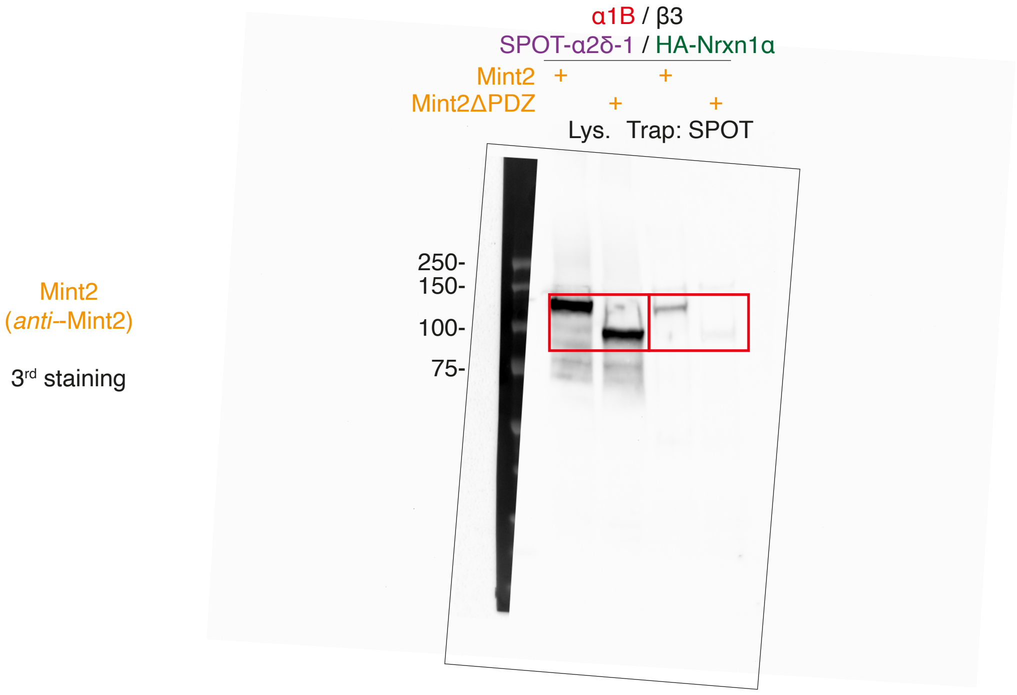

Figure 7B, panel 3, filename: 2022-08-19 10.49.57,3-2. scan_raw Kopie.png

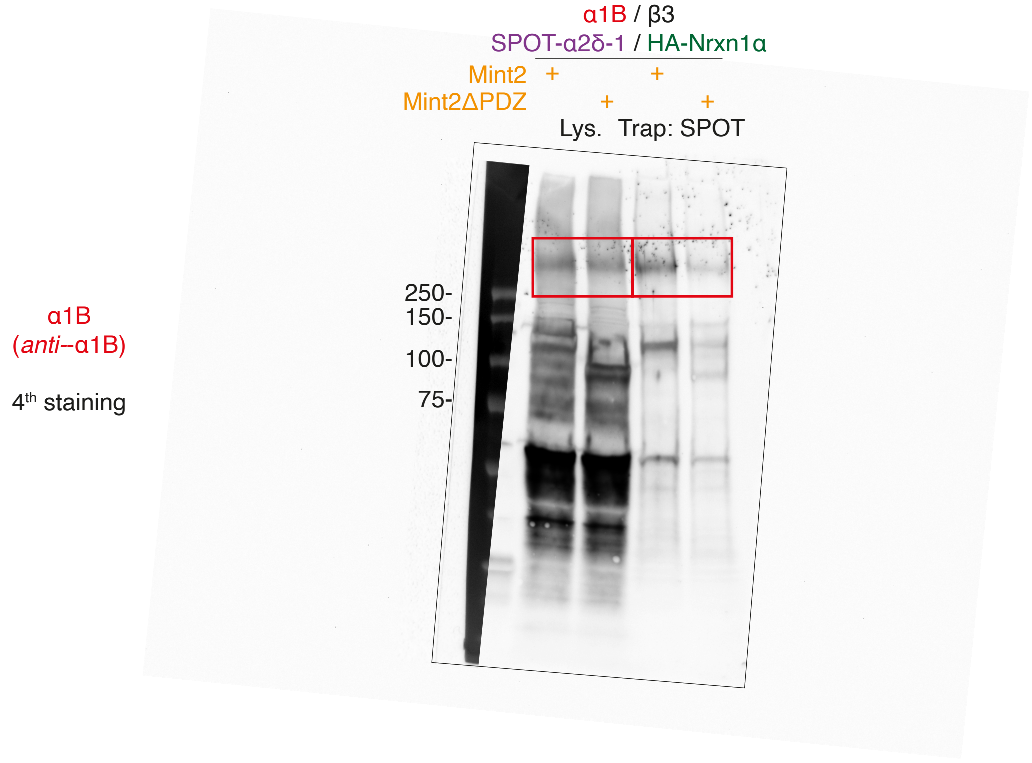

Figure 7C

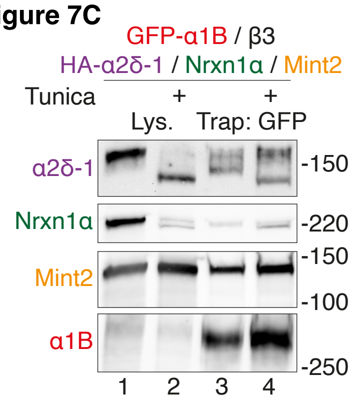

Figure 7C, panel 1, filename: 2022-11-08 11.30.34,5-3. scan_raw.png

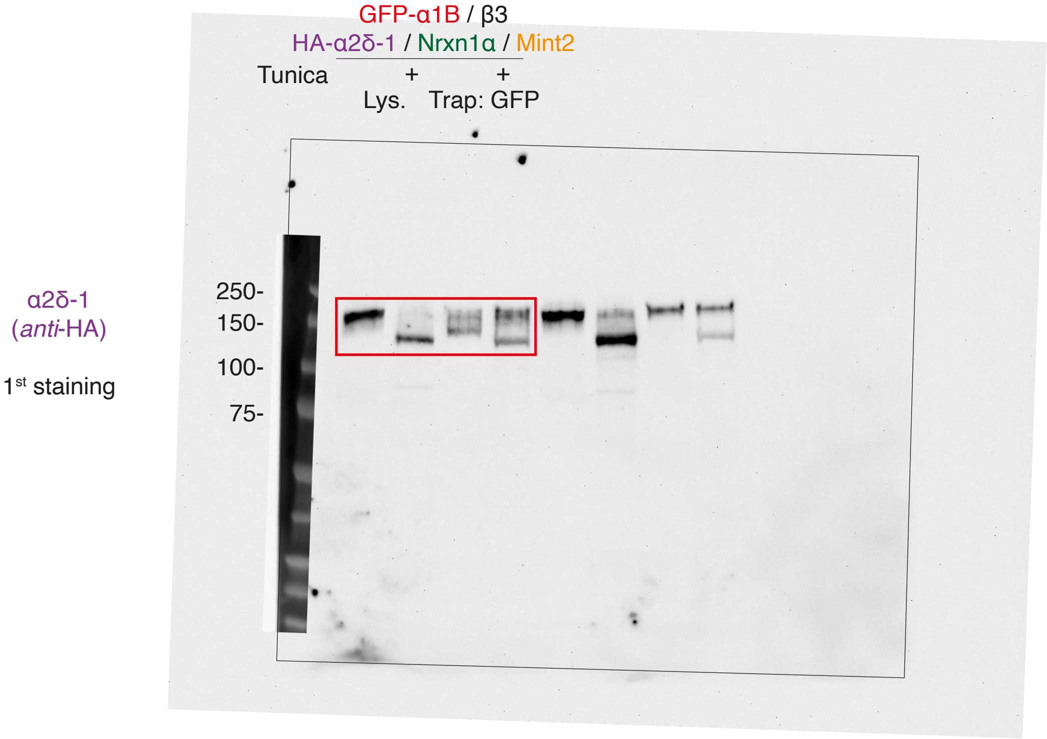

Figure 7C, panel 2, filename: 2022-11-09 13.23.58,3-2. scan_raw.png

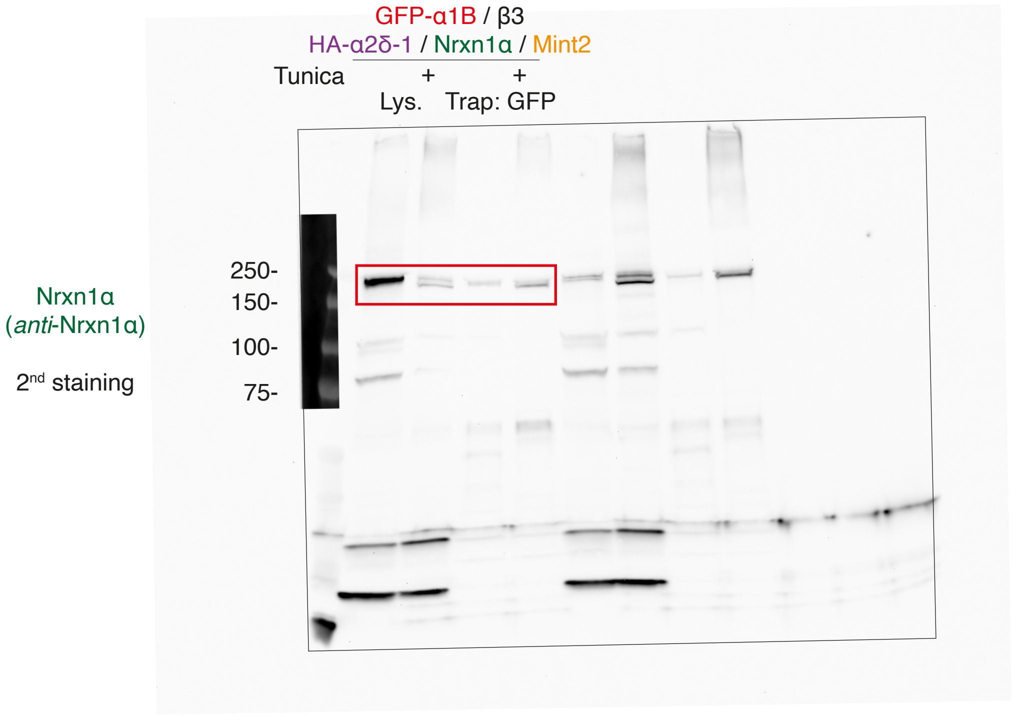

Figure 7C, panel 3, filename: 2022-11-11 12.11.23,1-1. scan_raw.png

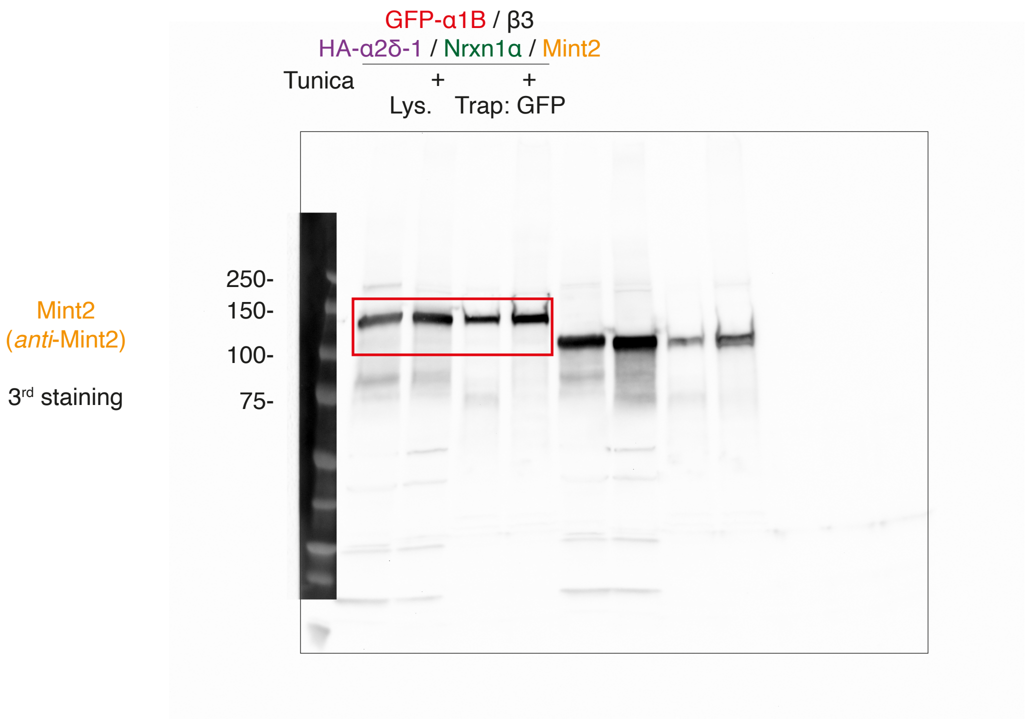

Figure 7C, panel 4, filename: 2022-11-15 12.24.19,1-1. scan_raw.png

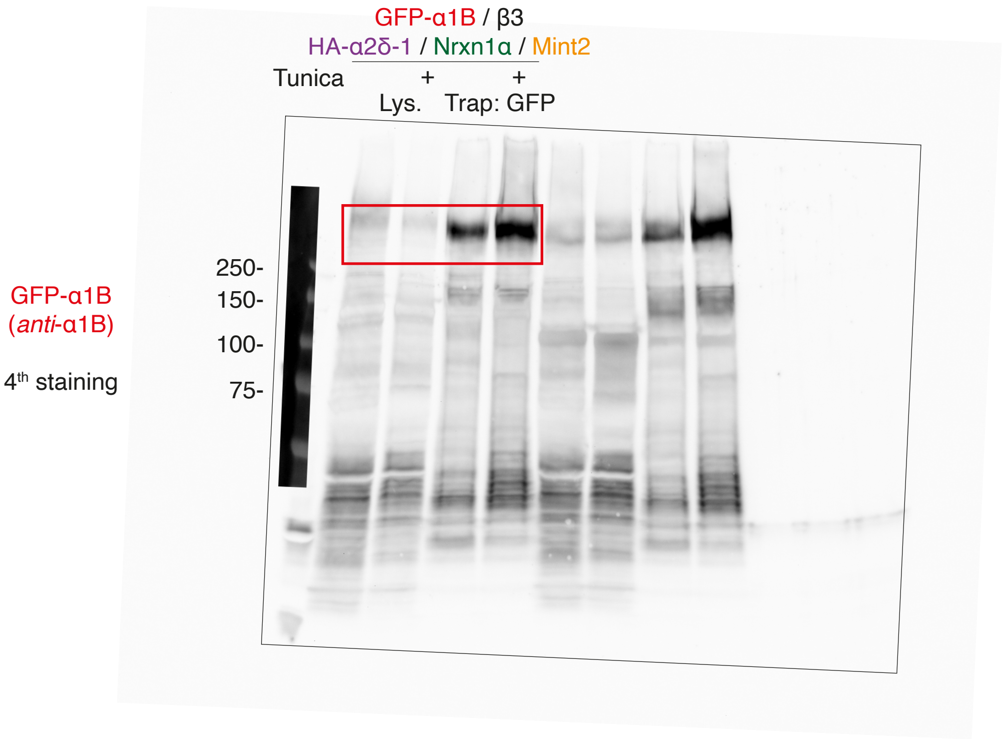

Figure 7D

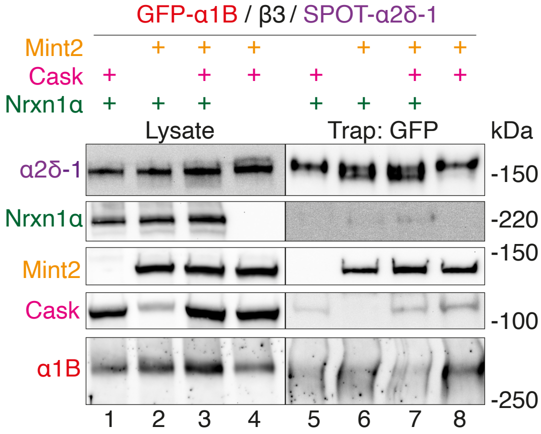

Figure 7D, panel 2 Lysate, filename: 2022-06-15 10.26.47,5-3. scan_raw.png

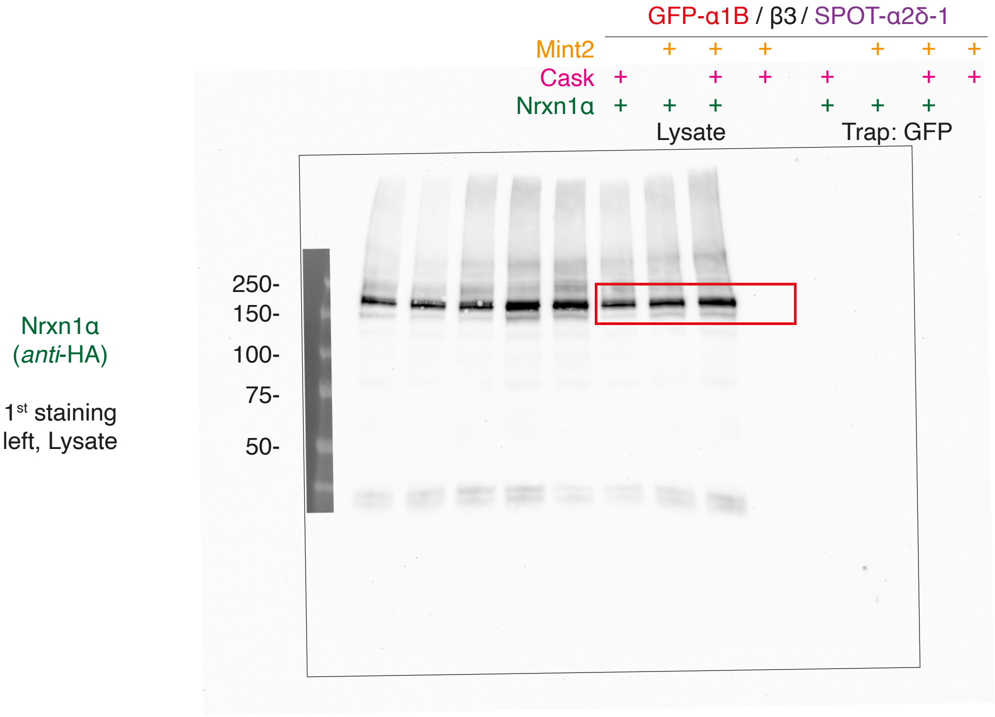

Figure 7D, panel 2 Trap, filename: 2022-06-15 10.47.29,5-3. scan_raw2.png

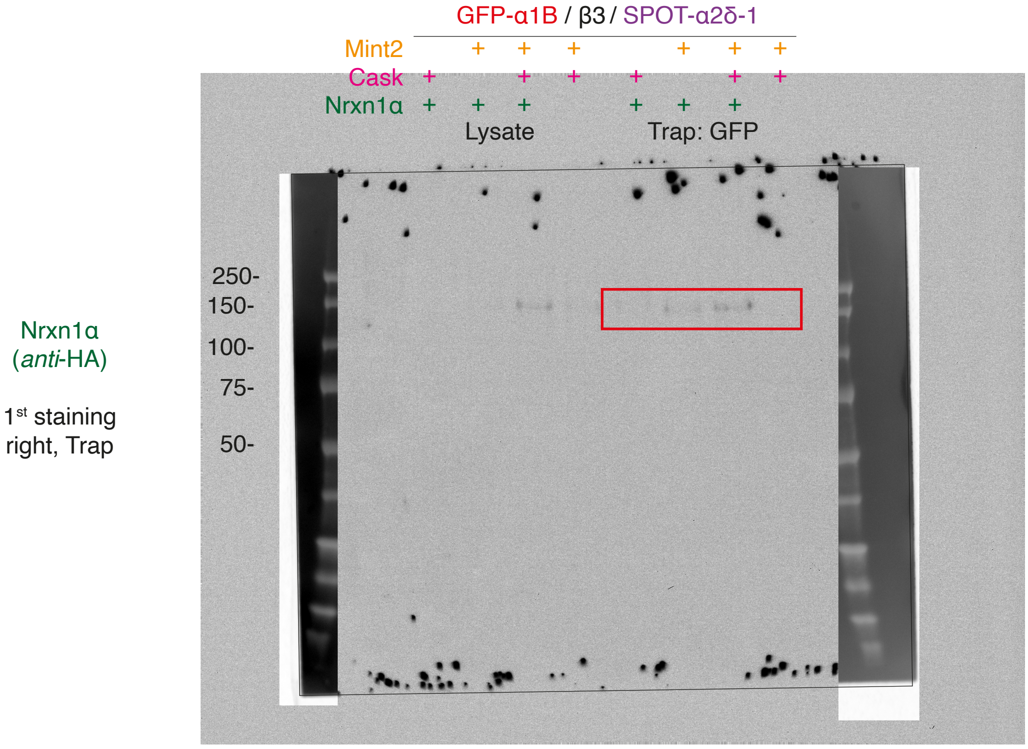

Figure 7D, panel 1 Lysate, filename: 2022-07-07 11.01.25,3-2. scan_raw.png

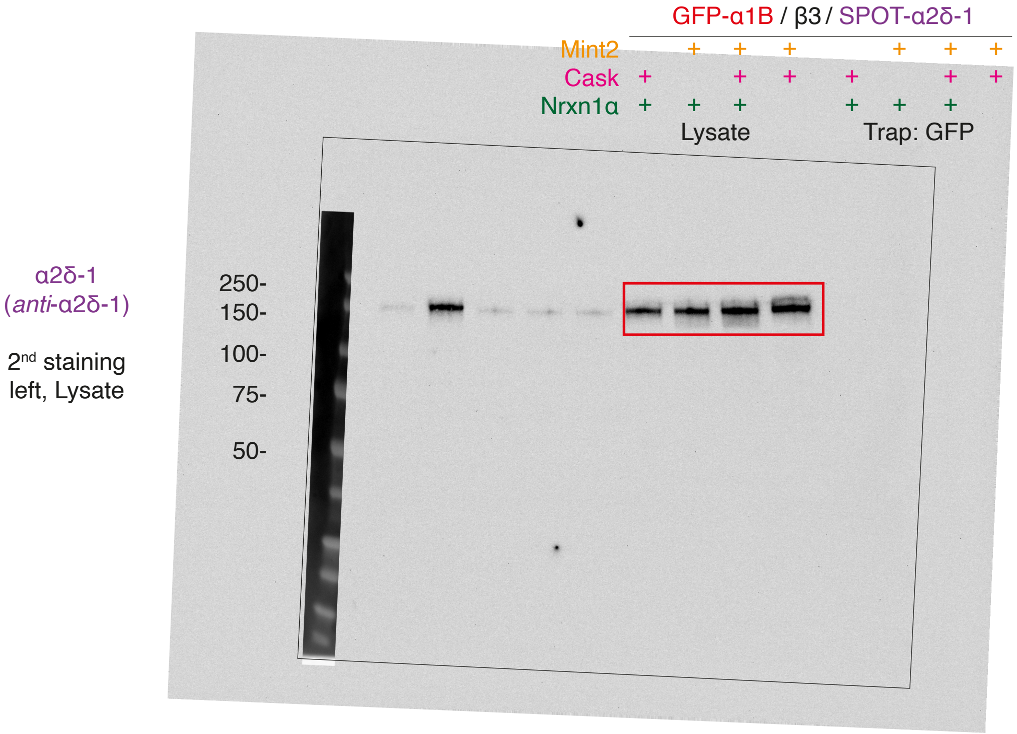

Figure 7D, panel 2 Trap, filename: 2022-06-21 10.12.46,3-2. scan_raw.png

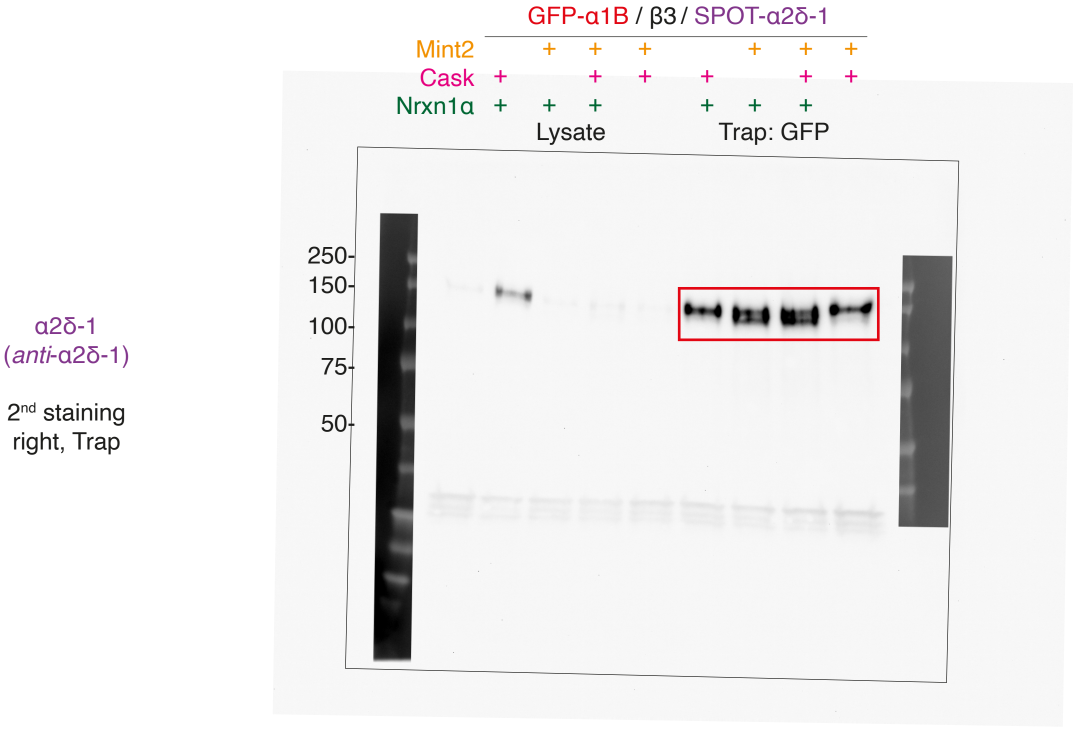

Figure 7D, panel 4 Lysate, filename: 2022-06-22 10.43.21,3-2. scan_raw.png

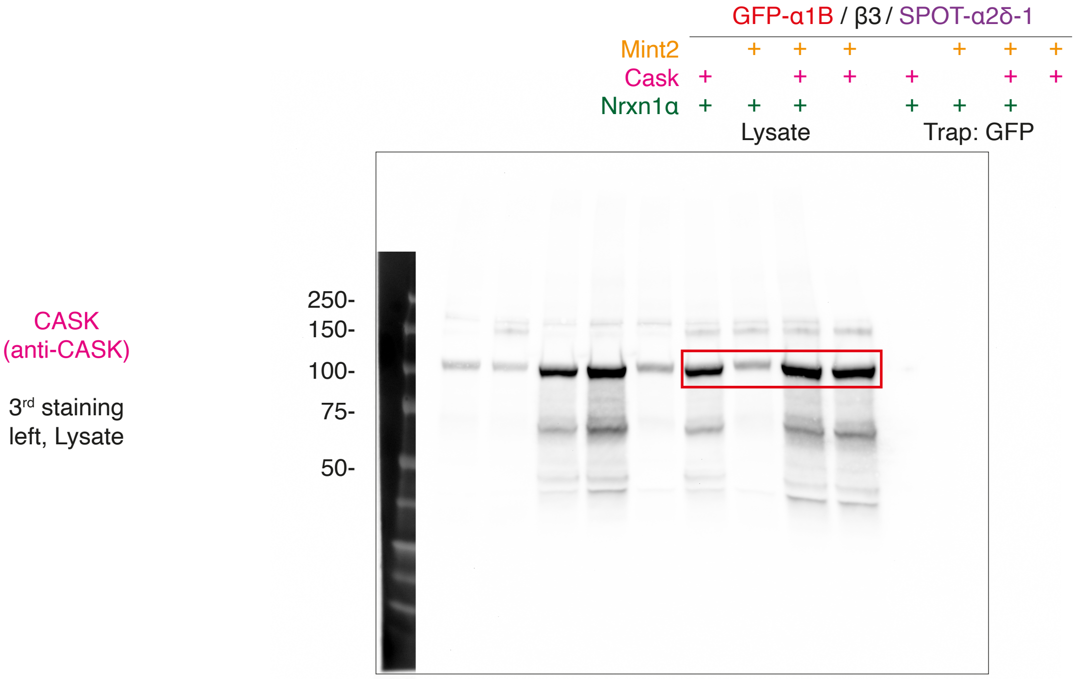

Figure 7D, panel 4 Trap, filename: 2022-06-22 11.01.24,3-2. scan_raw.png

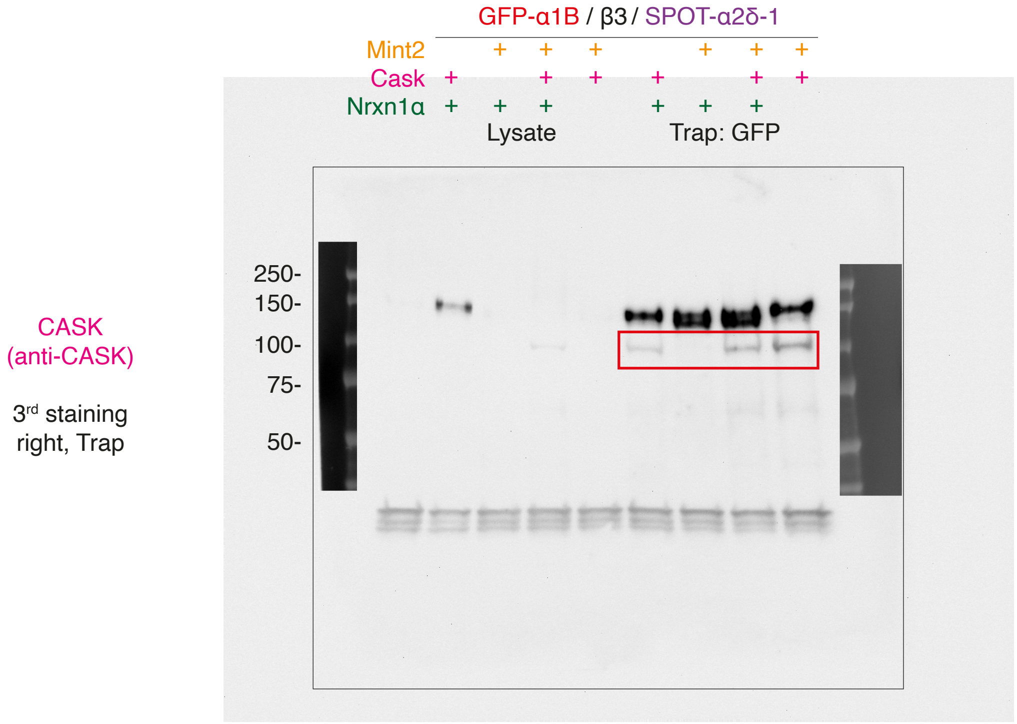

Figure 7D, panel 3 Lysate, filename: 2022-06-24 10.54.28,3-2. scan_raw.png

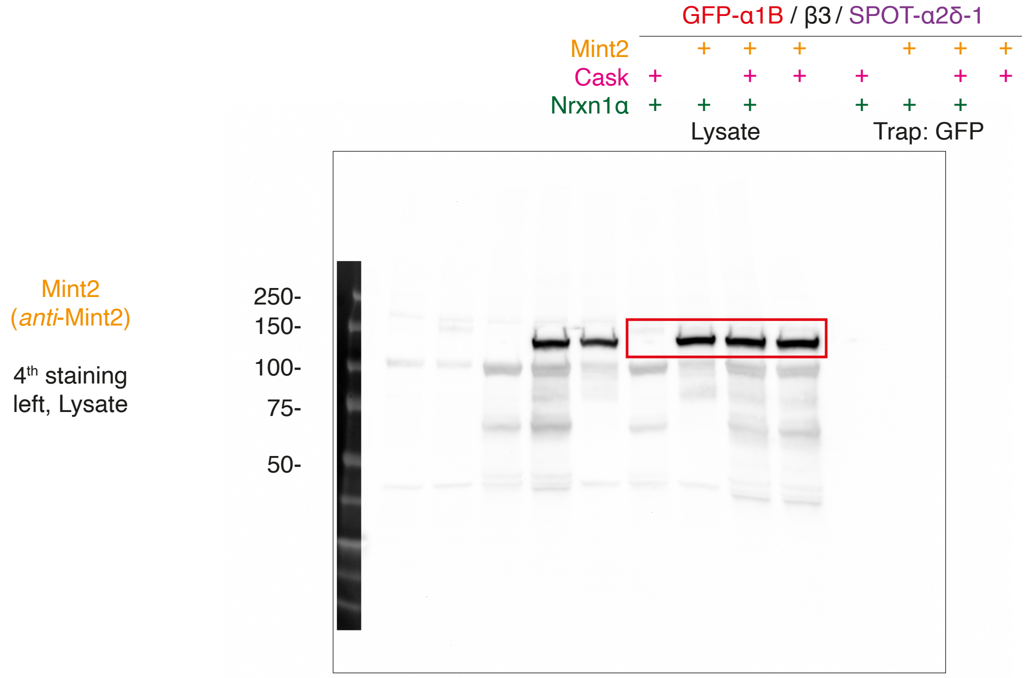

Figure 7D, panel 3 Trap, filename: 2022-07-05 10.19.34,3-2. scan_raw.png

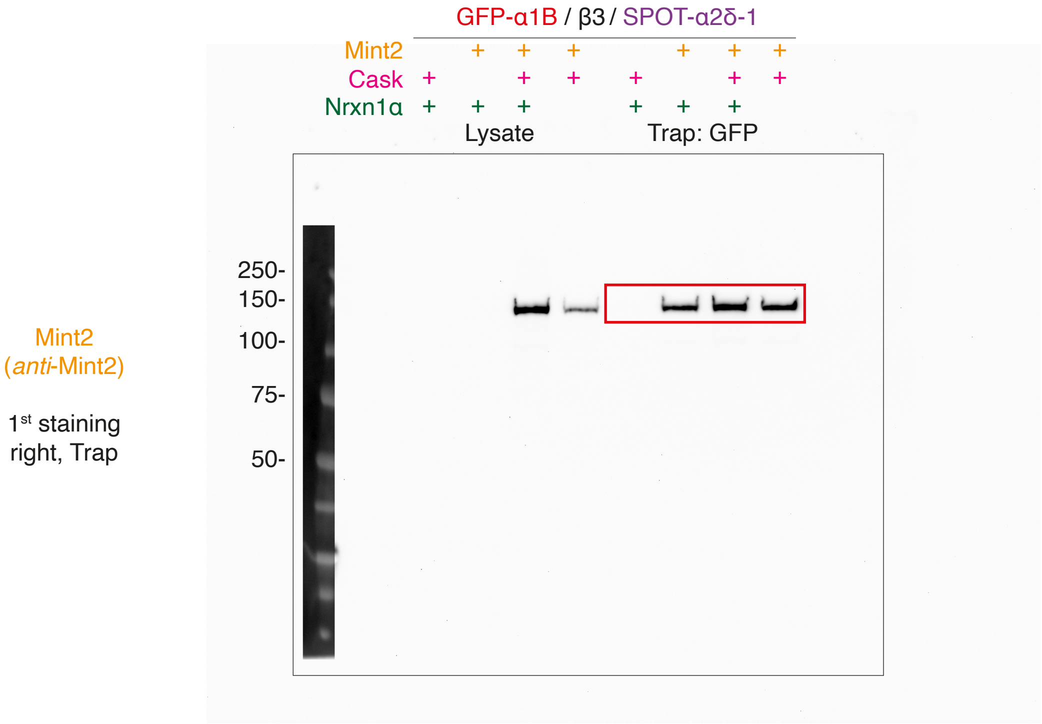

Figure 7D, panel 5 Lysate, filename: 2022-07-08 10.32.52,3-2. scan_raw Kopie.png

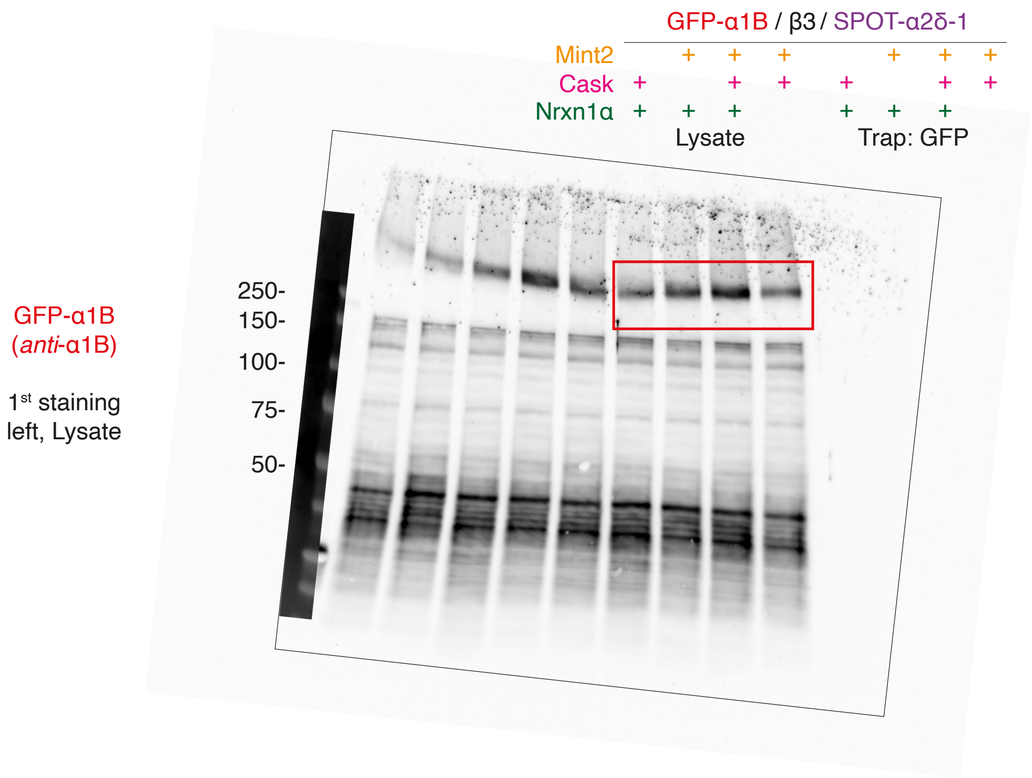

Figure 7D, panel 5 Trap, filename: 2022-06-23 10.41.36,3-2. scan_raw.png

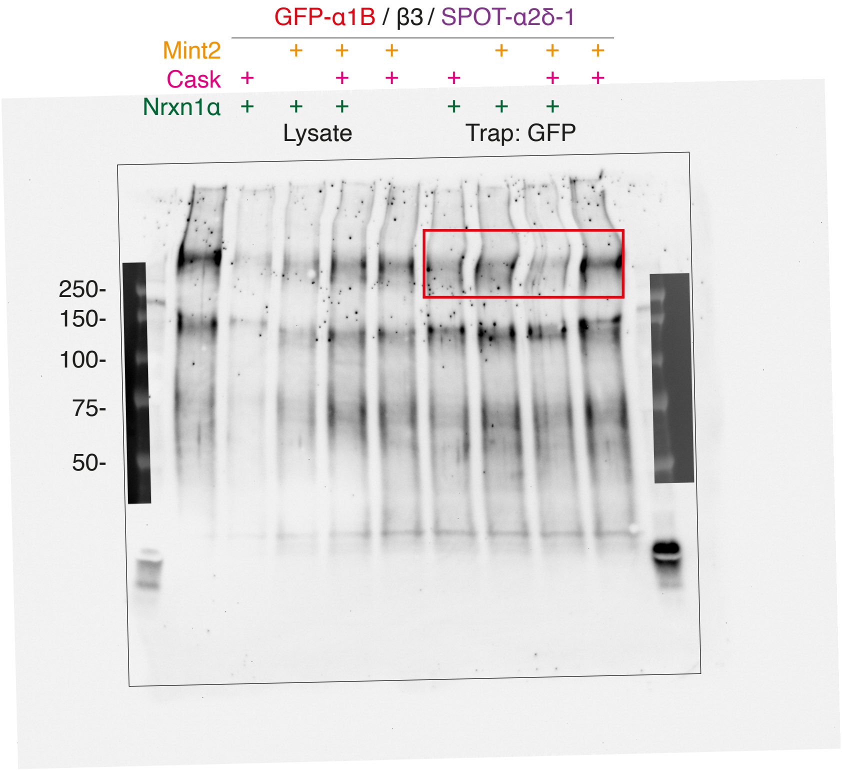

GFP-α1B
(*anti*-α1B)

4th staining
right, Trap

Figure 7E

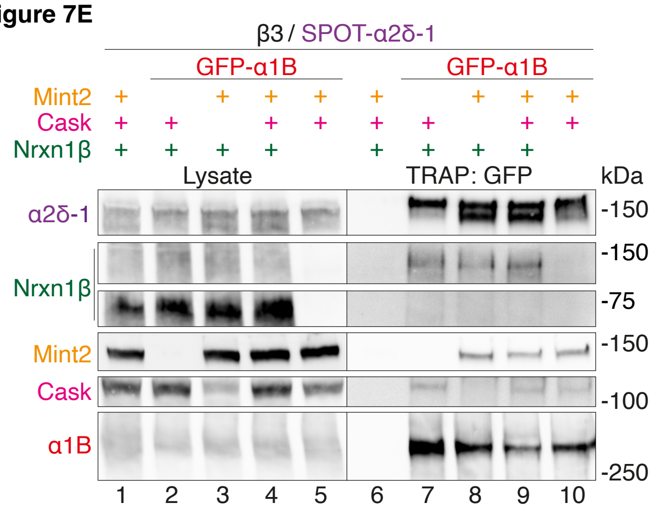

Figure 7E, panel 2+3 Lysate, flename: anti-HA (nrxn1b) ms 1.1000,5-3. scan_raw.png

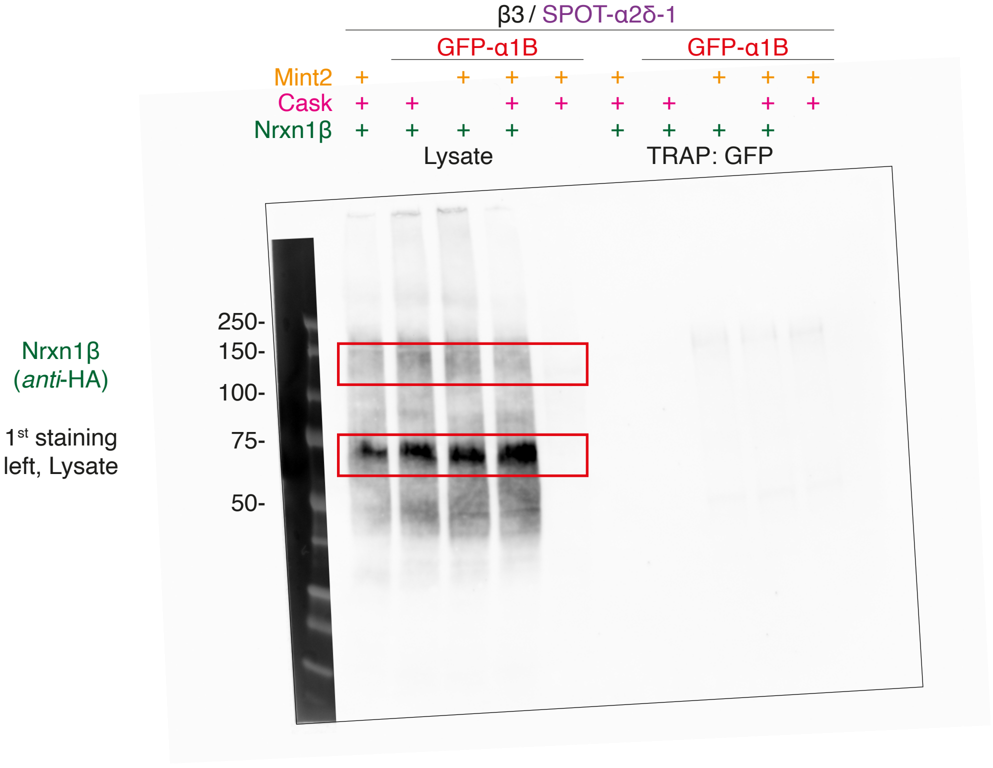

Figure 7E, panel 2+3 Trap, filename: anti-HA (nrxn1b) ms 1.1000,5-3. scan_raw_pushed.png

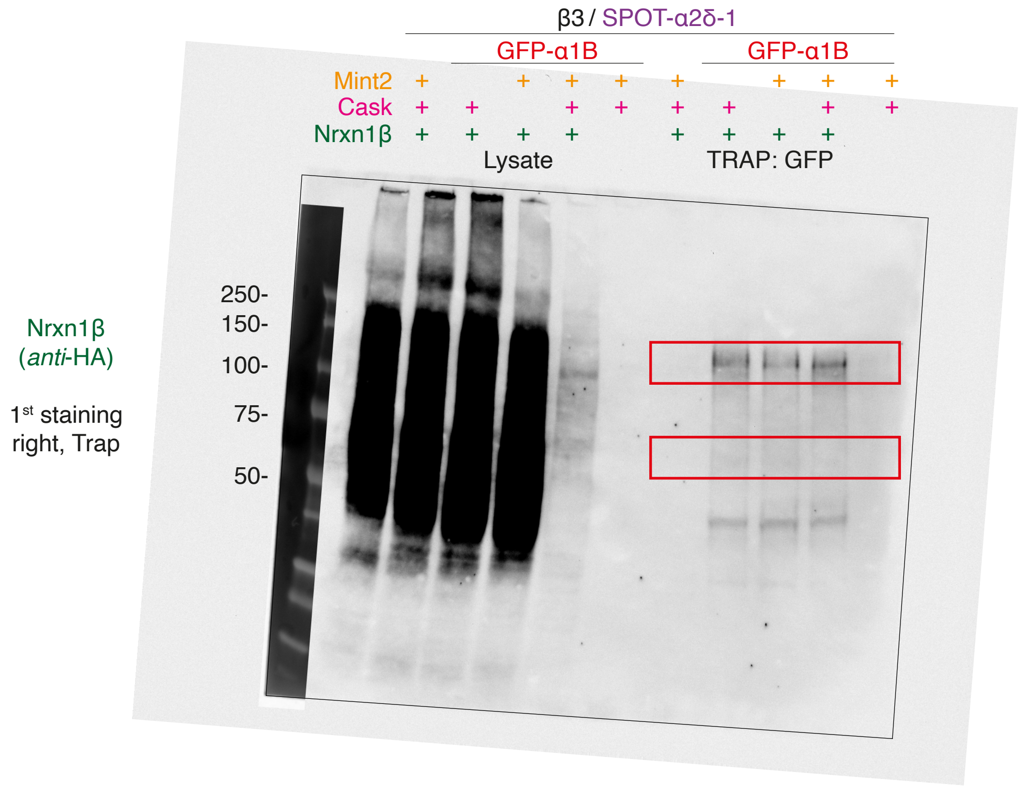

Figure 7E, panel 1, filename: a-2d-1 ms 1.1000,3-2. scan_raw.png

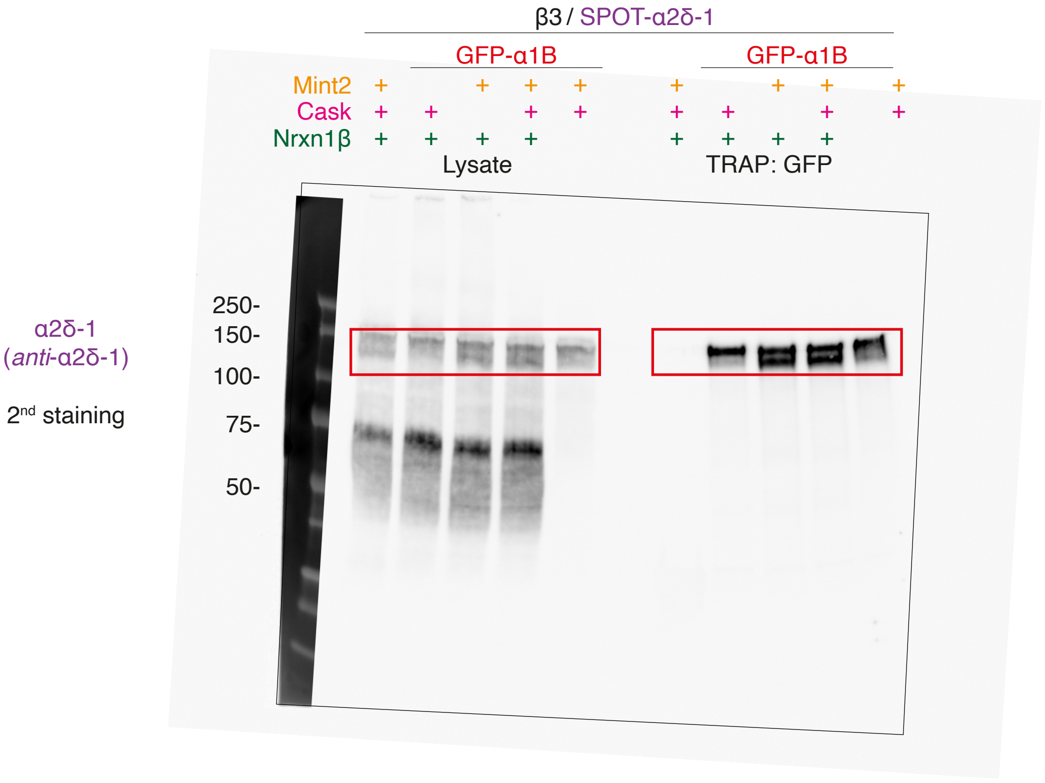

Figure 7E, panel 5 Lysate, filename: 2024-12-18 10.47.59,3-2. scan_raw.png

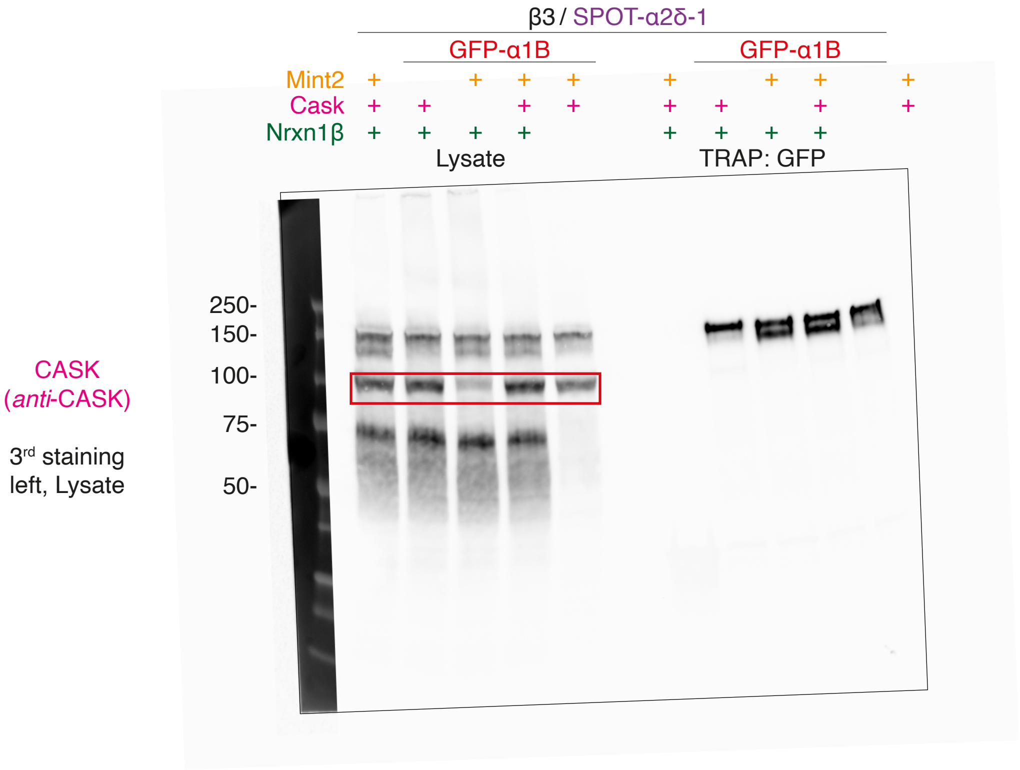

Figure 7E, panel 5 Trap, filename: 2024-12-18 10.47.59,3-2. scan_raw.png

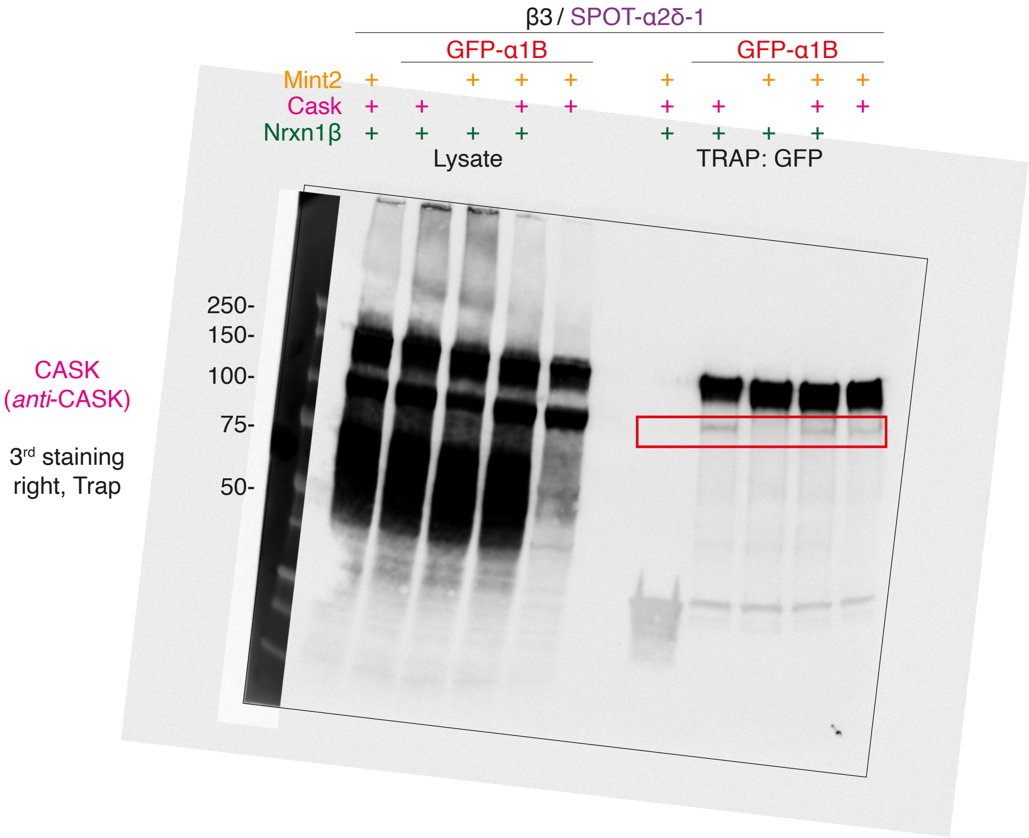

Figure 7E, panel 4, filename: 2024-12-19 10.28.47,2-1. scan+Marker_raw.png

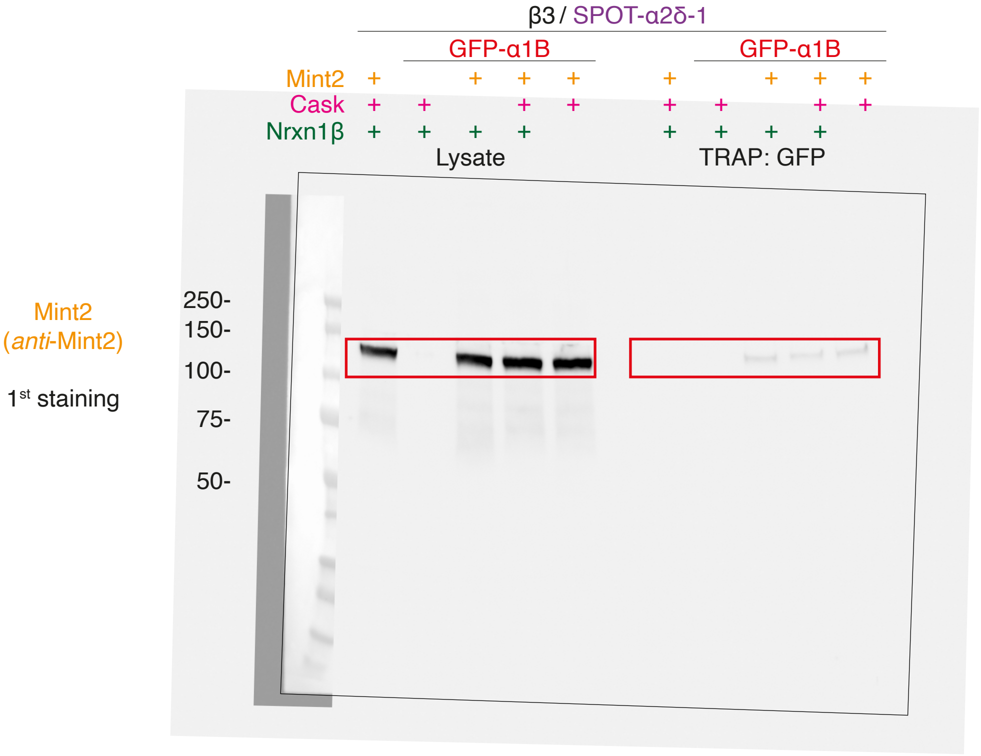

Figure 7E, panel 6, filename: 2024-12-20 09.57.48,3-2. scan_raw.png

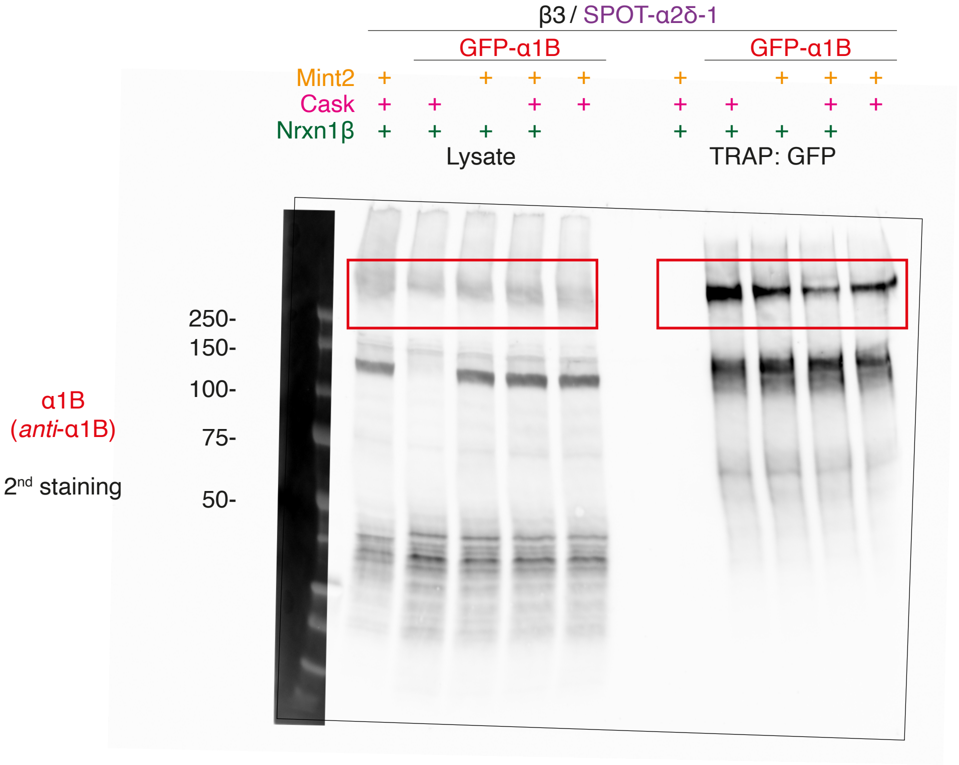

Supp. Figure S1A

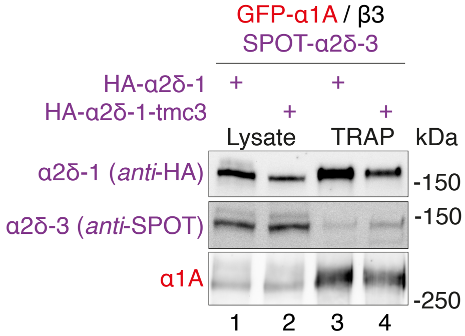

Figure S1A, panel 1, filename: 2023-02-15 11.30.56, 3-2. scan_raw.png

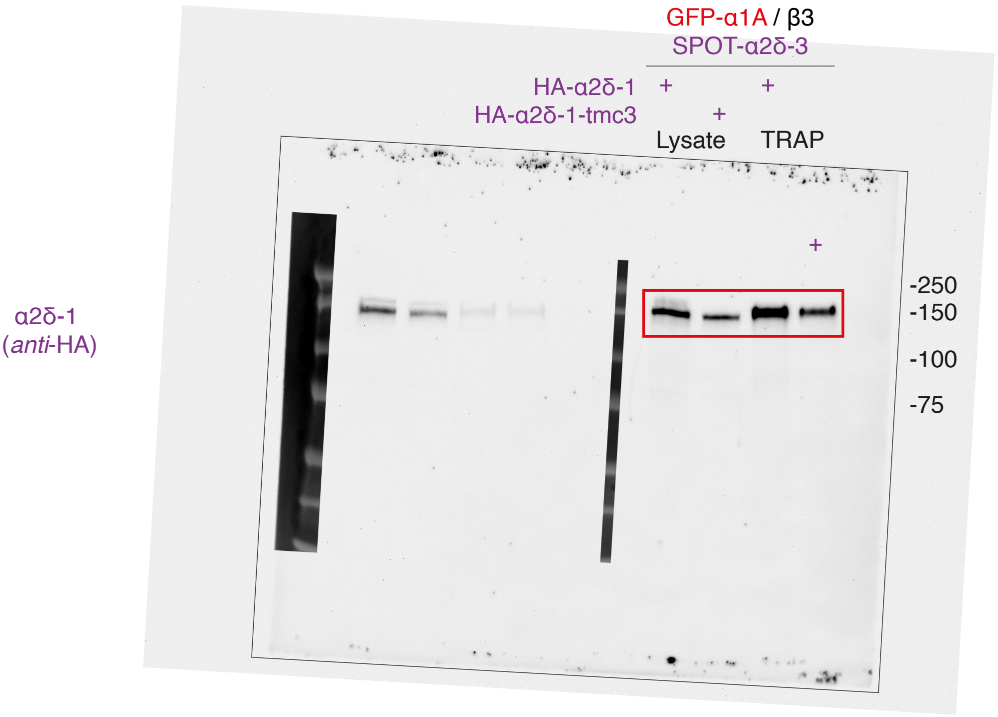

Figure S1A, panel 2, filename: 2023-02-24 12.10.05,3-2. scan_raw.png

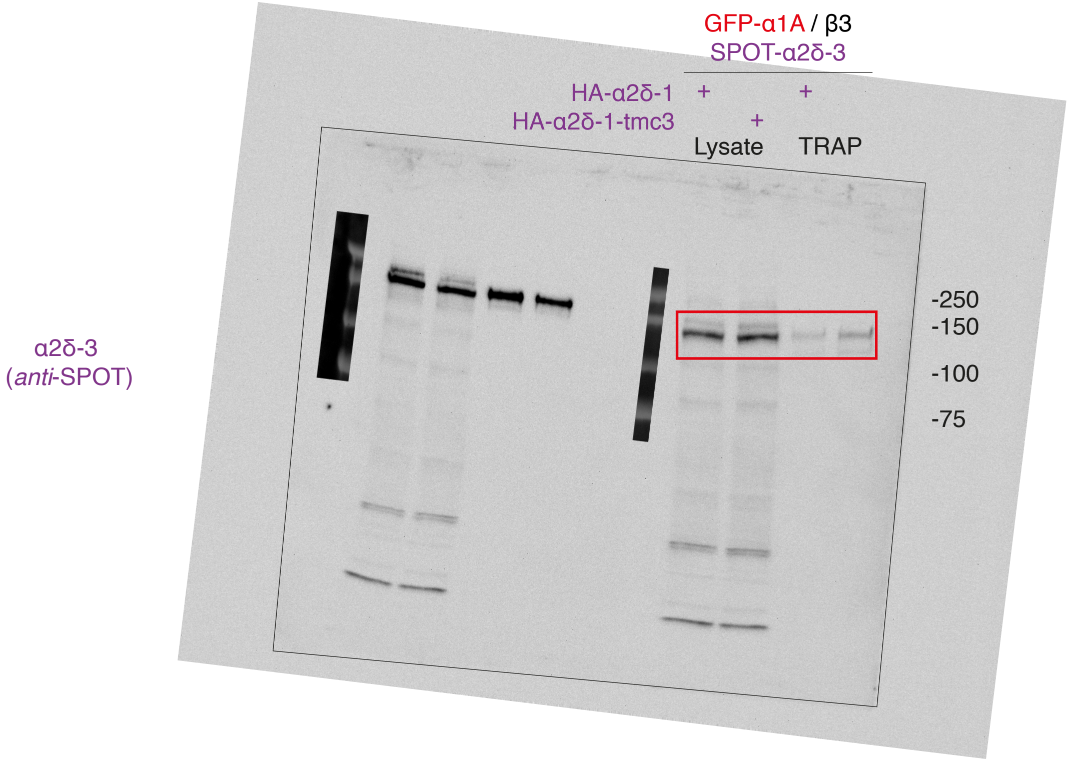

Figure S1A, panel 3, filename: 2023-02-28 11.07.44,5-3. scan_raw.png

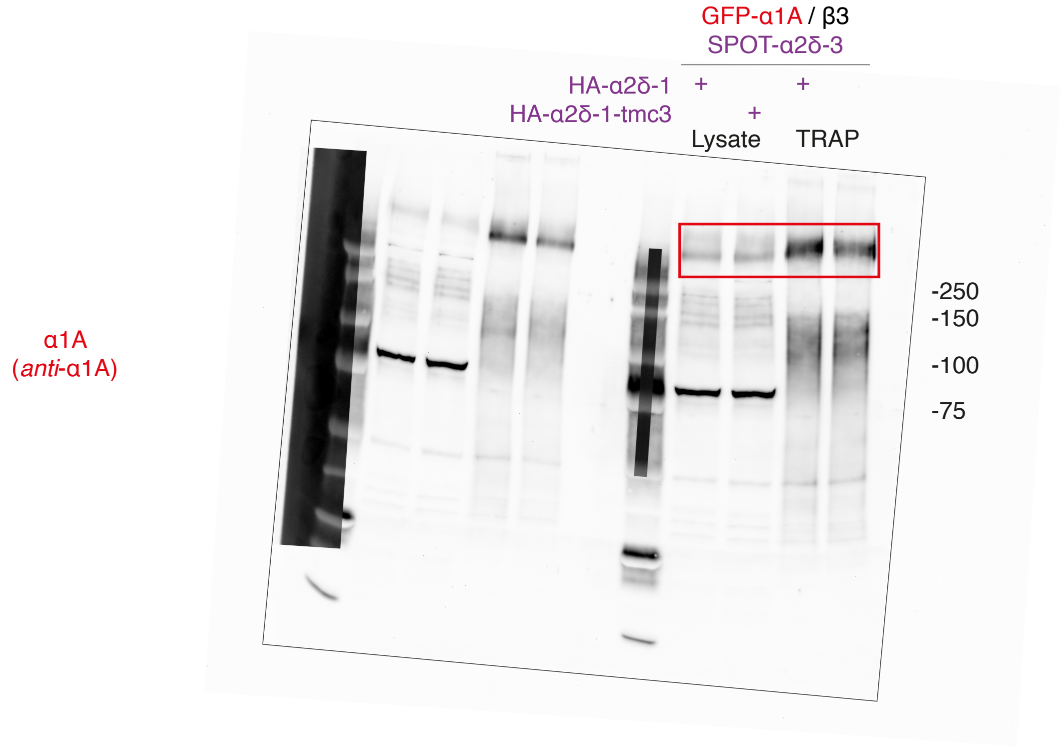

Supp. Figure S2B

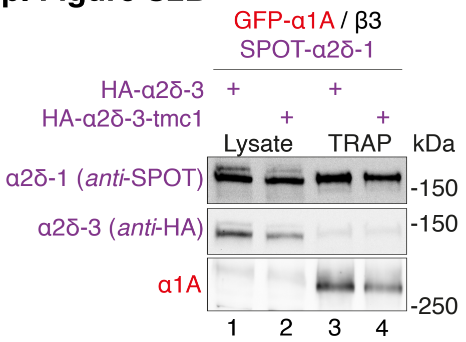

Figure S1B, panel 1, filename: 2023-02-15 11.30.56, 3-2. scan_raw.png

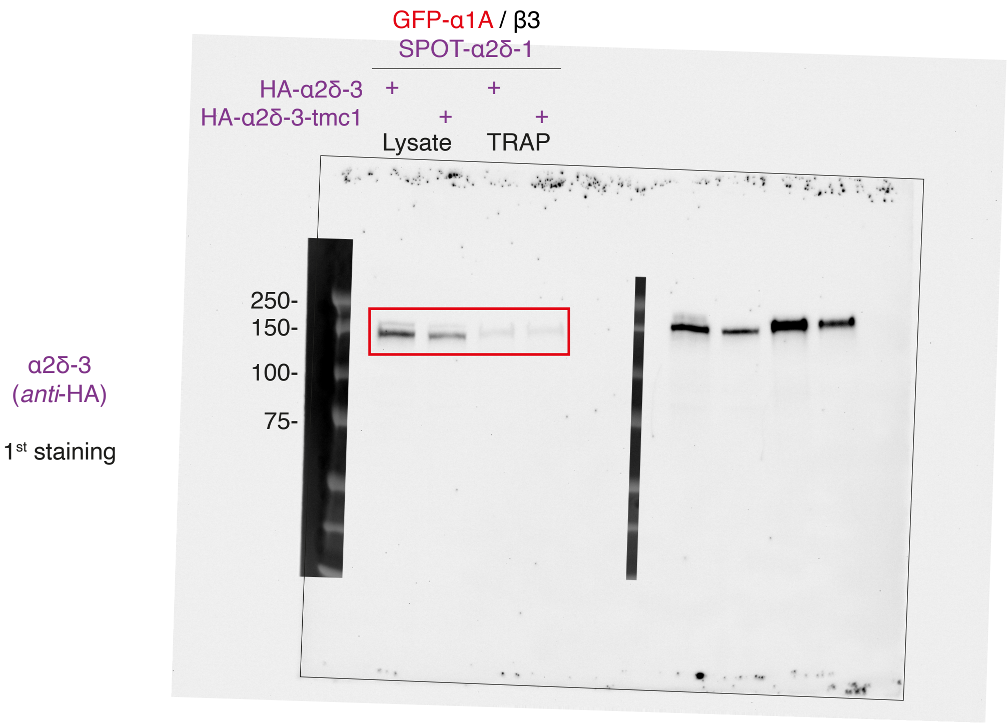

Figure S1B, panel 2, filename: 2023-02-24 12.10.05,3-2. scan_raw.png

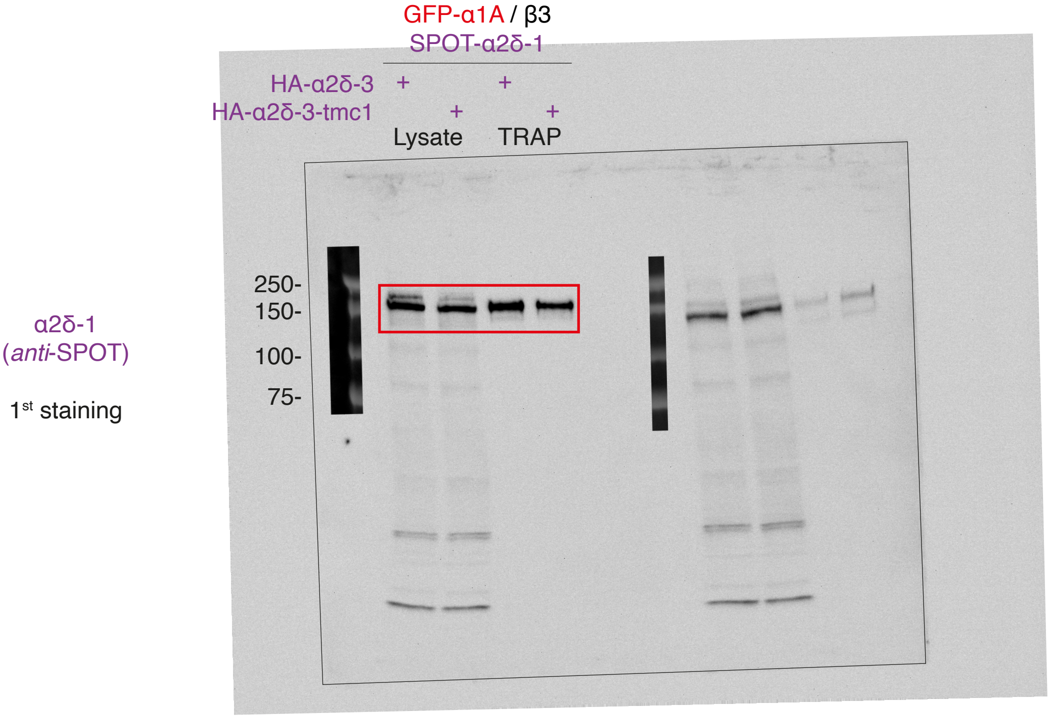

Figure S1B, panel 3, filename: 2023-02-28 11.07.44,5-3. scan_raw.png

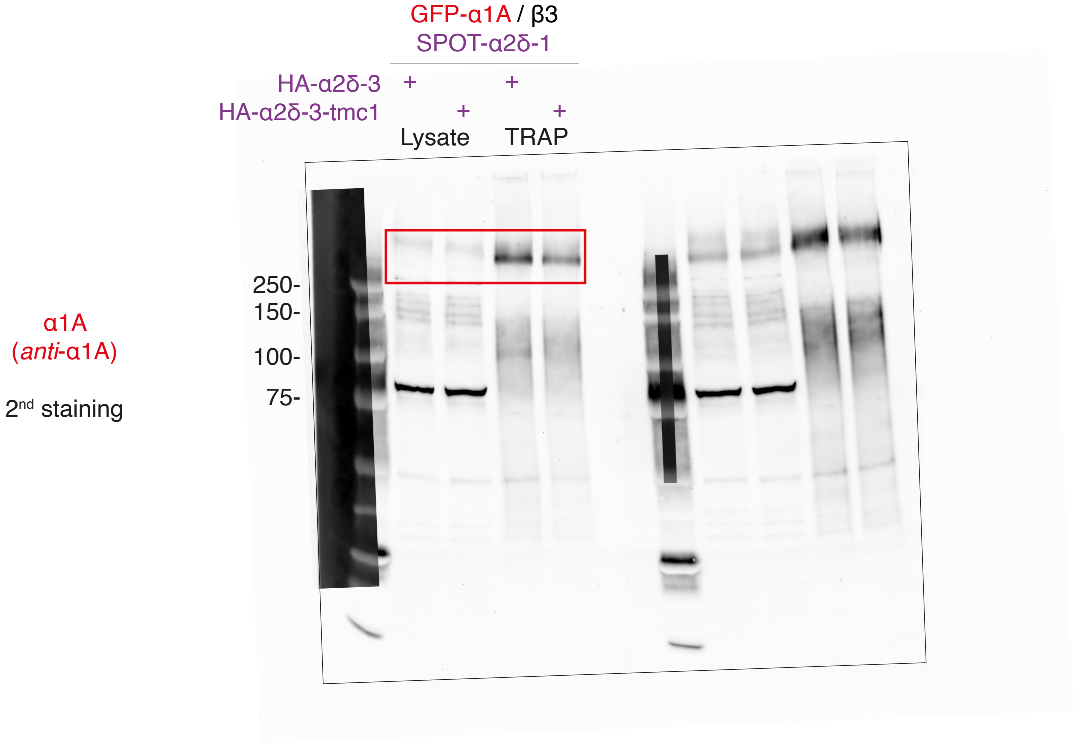

Supp. Figure S2A

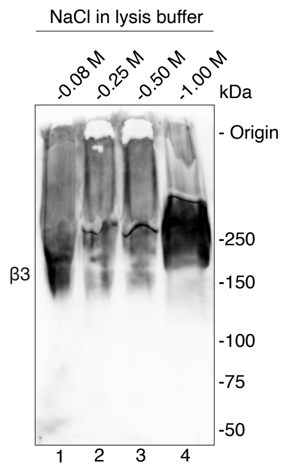

Figure S2A, filename: 2021-08-04 10.33.08,1-1. scan_raw.png

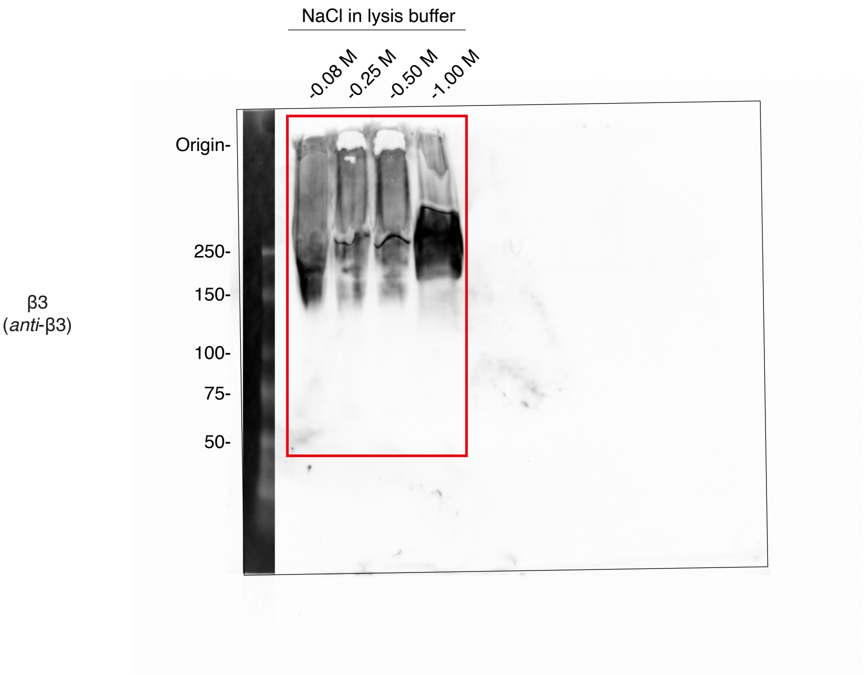

Supp. Figure S2B

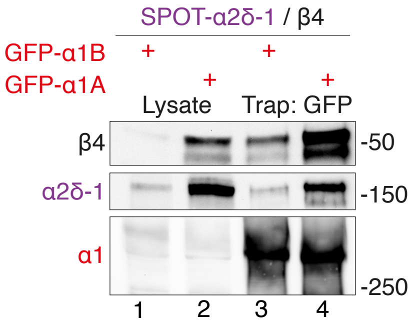

Figure S2B, panel 1, filename: 2023-03-21 11.53.18,5-3. scan_raw.png

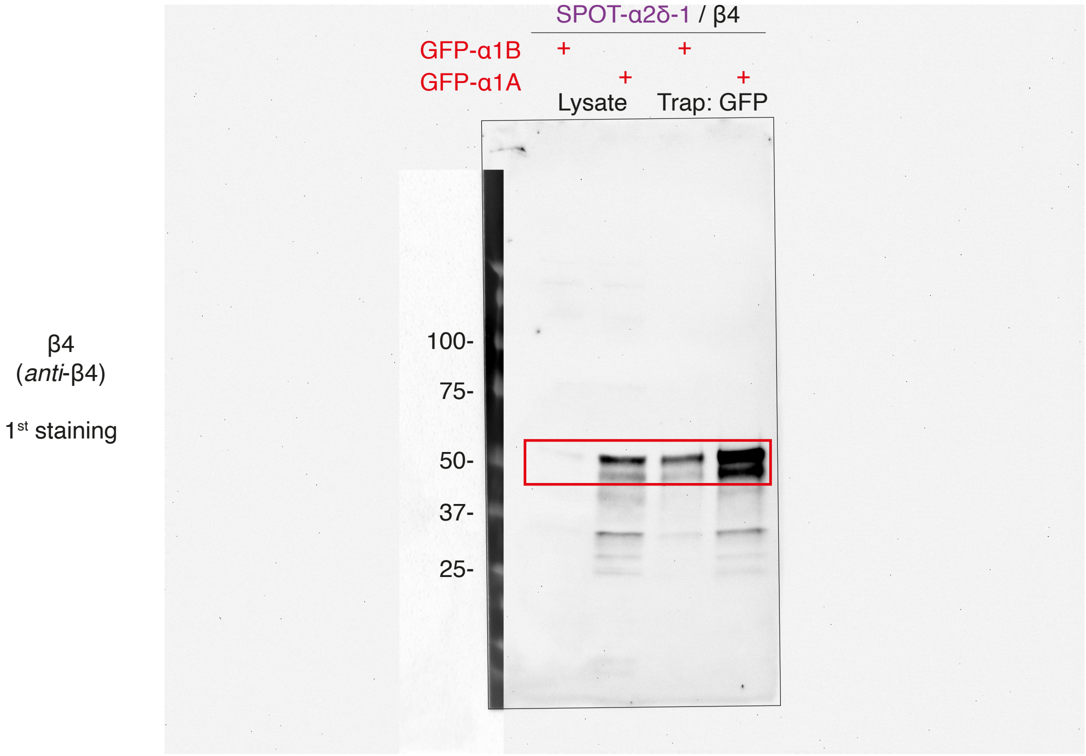

Figure S2B, panel 2, filename: 2023-03-22 11.08.39,3-2. scan_raw.png

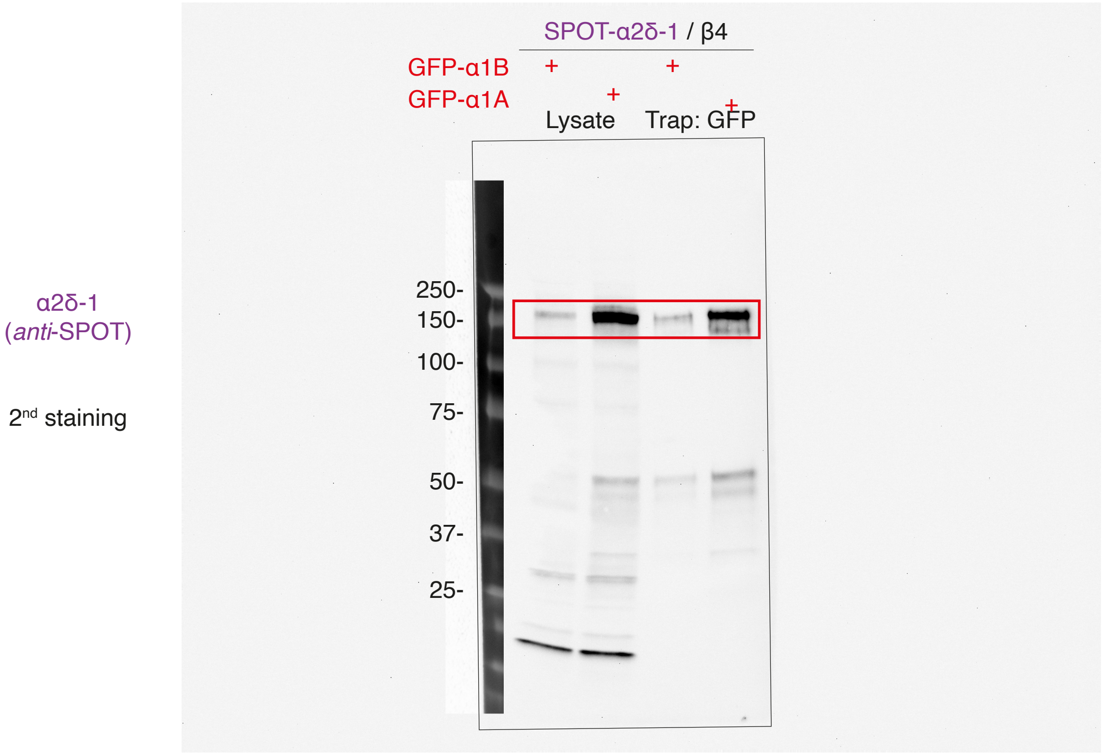

Figure S2B, panel 3, filename: 2023-04-26 10.52.57,5-3. scan_raw.png

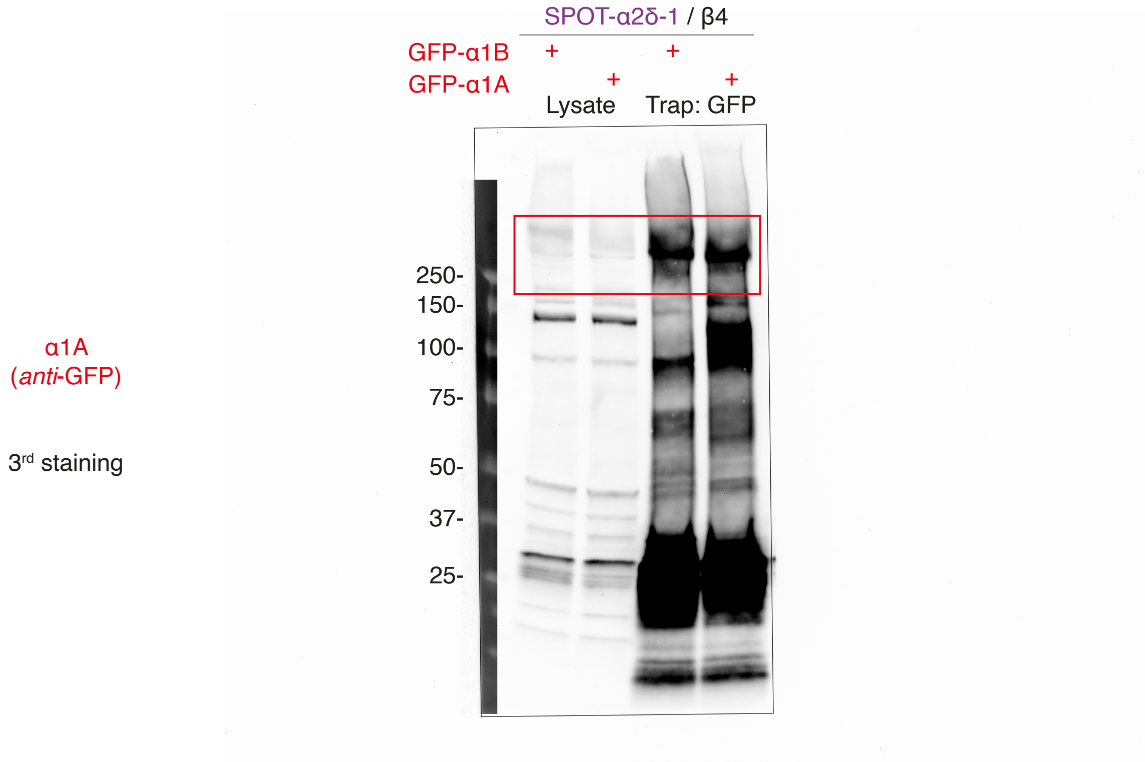

Supp. Figure S2C

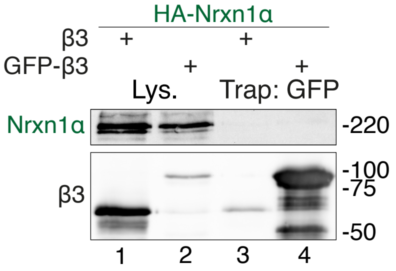

Figure S2C, panel 1, filename: 2021-07-14 10.02.46,3-2. scan_raw Kopie.png

Nrxn1α
(*anti*-HA)

1st staining

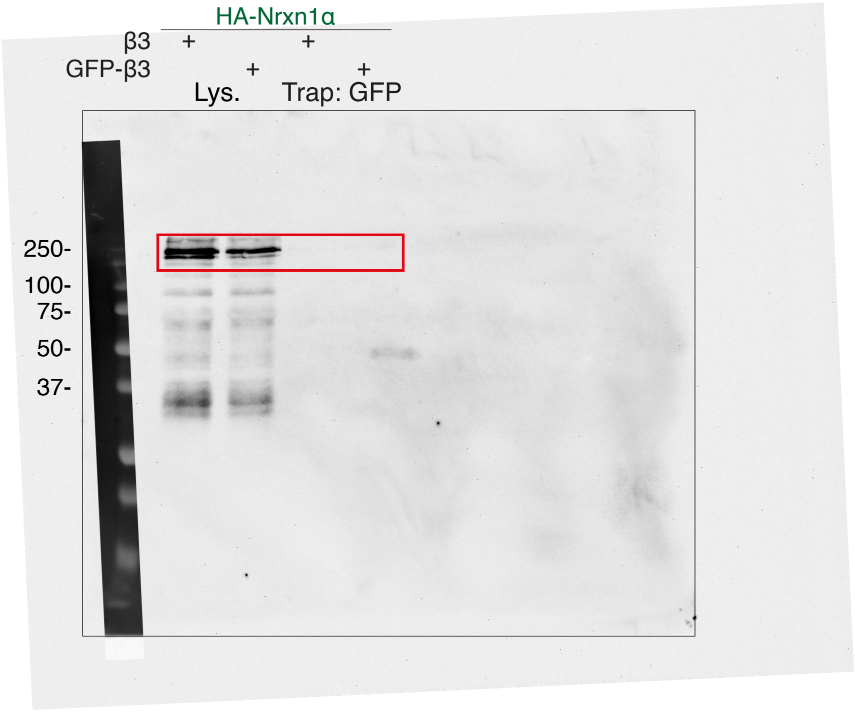

Figure S2C, panel 2, filename: 2021-07-15 10.39.27,3-2. scan_raw.png

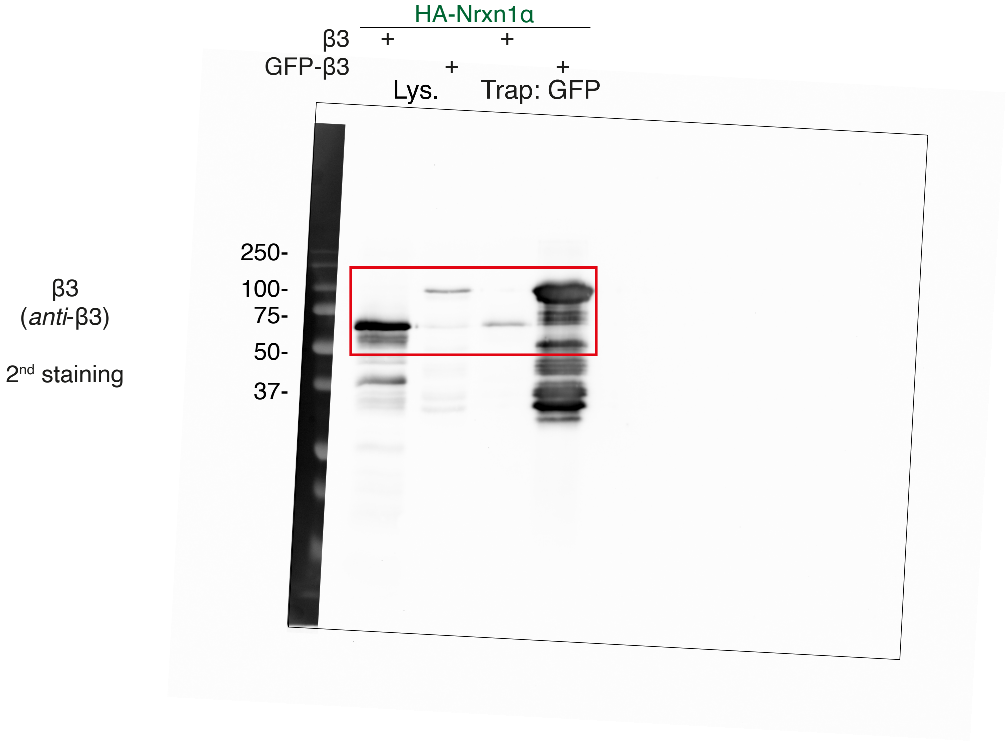

Supp. Figure S3A

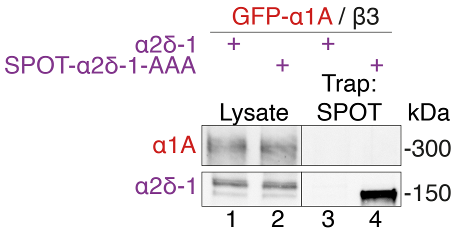

Figure S3A, panel 2, filename: 2022-02-22 13.24.24,1-1. scan_raw.png

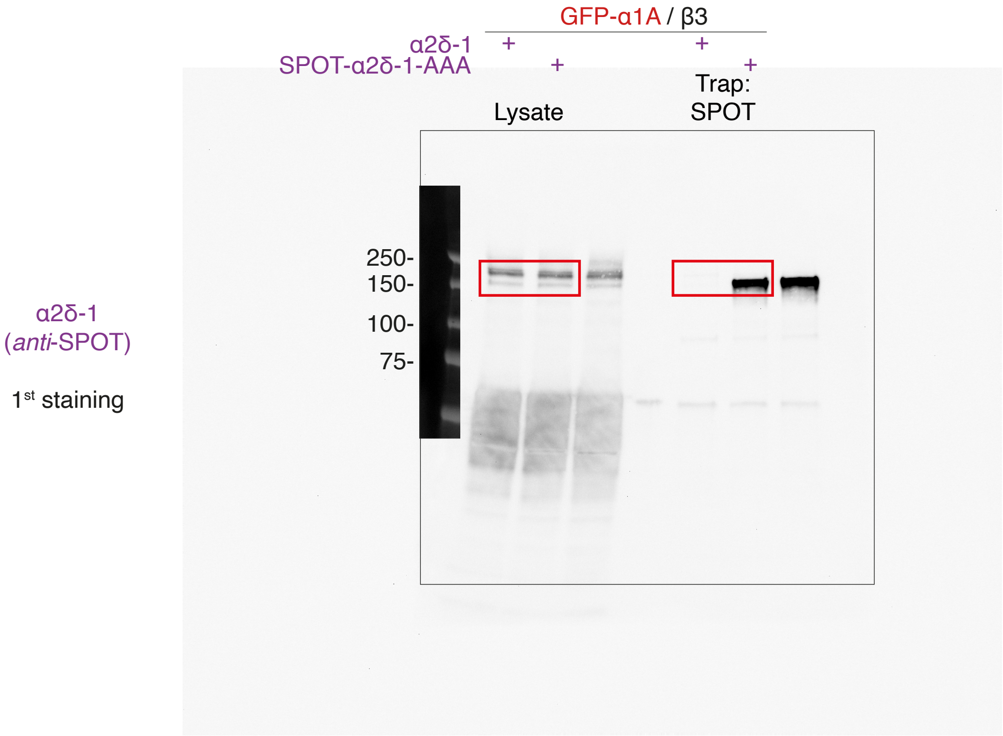

Figure S3A, panel 1, filename: 2022-02-23 12.28.08,3-2. scan_raw Kopie.png

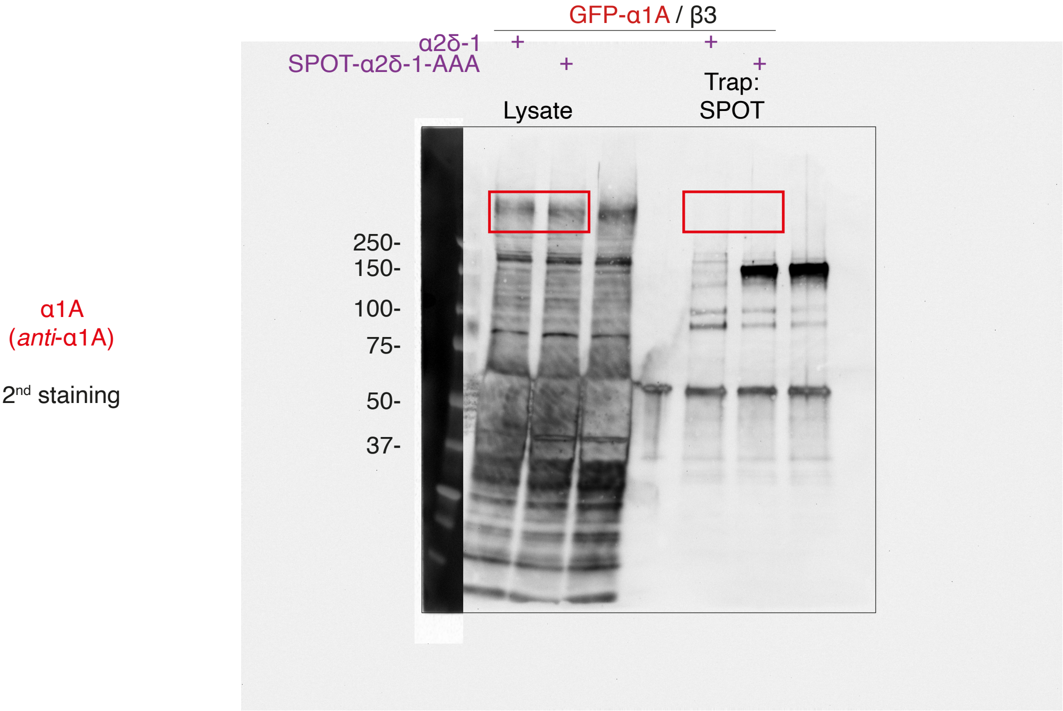

Supp. Figure S3B

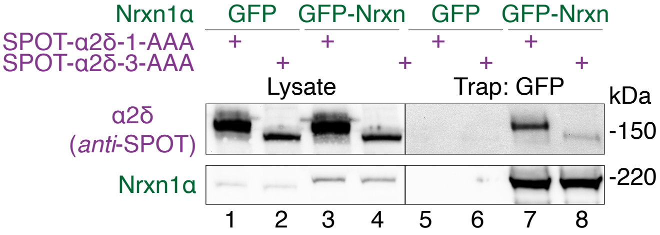

Figure S3B, panel 1 Lysate, filename: 2023-12-01 11.37.00,5-3. scan_raw.png

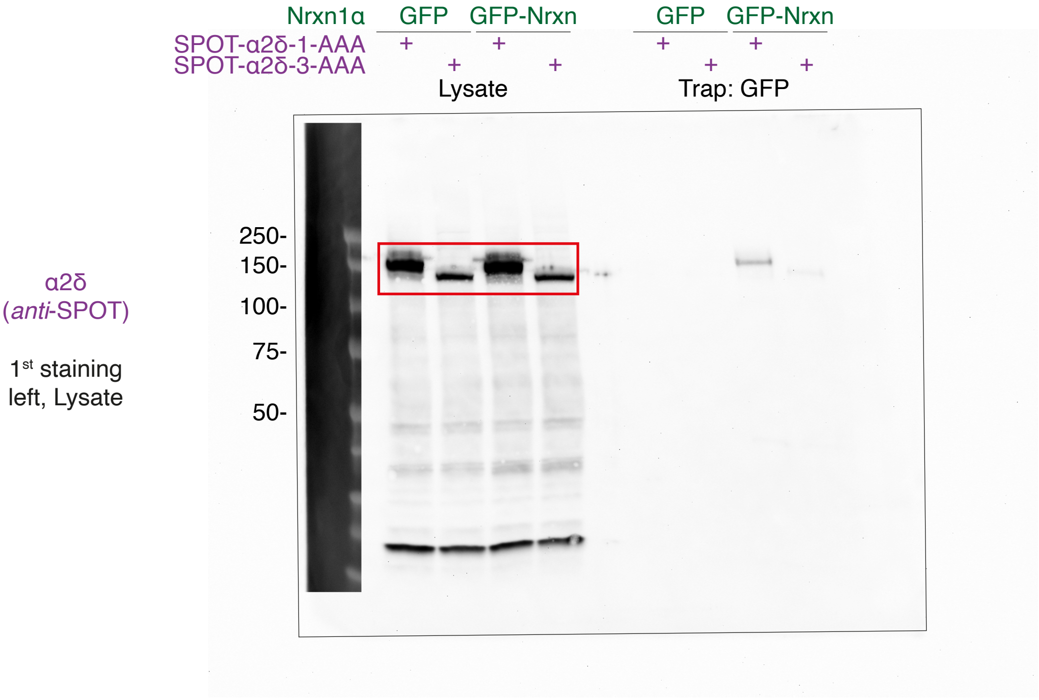

Figure S3B, panel 1 Trap, filename: 2023-12-01 11.37.00,5-3. scan_raw_pushed.png

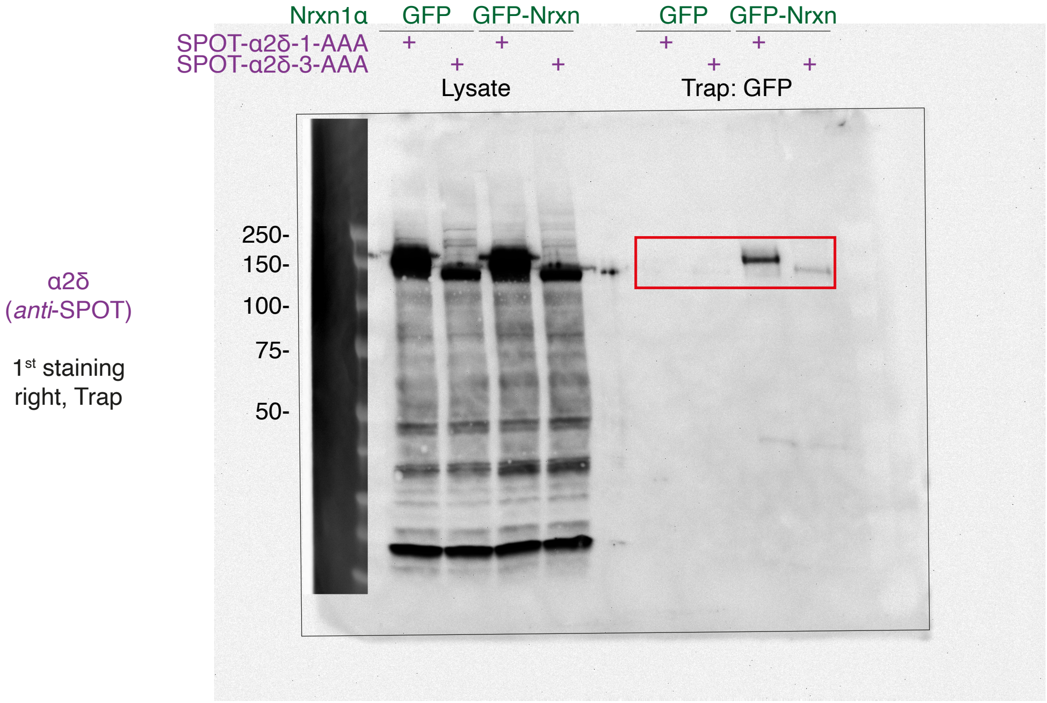

Figure S3B, panel 2, filename: 2023-12-05 12.11.55,1-1. scan_raw.png

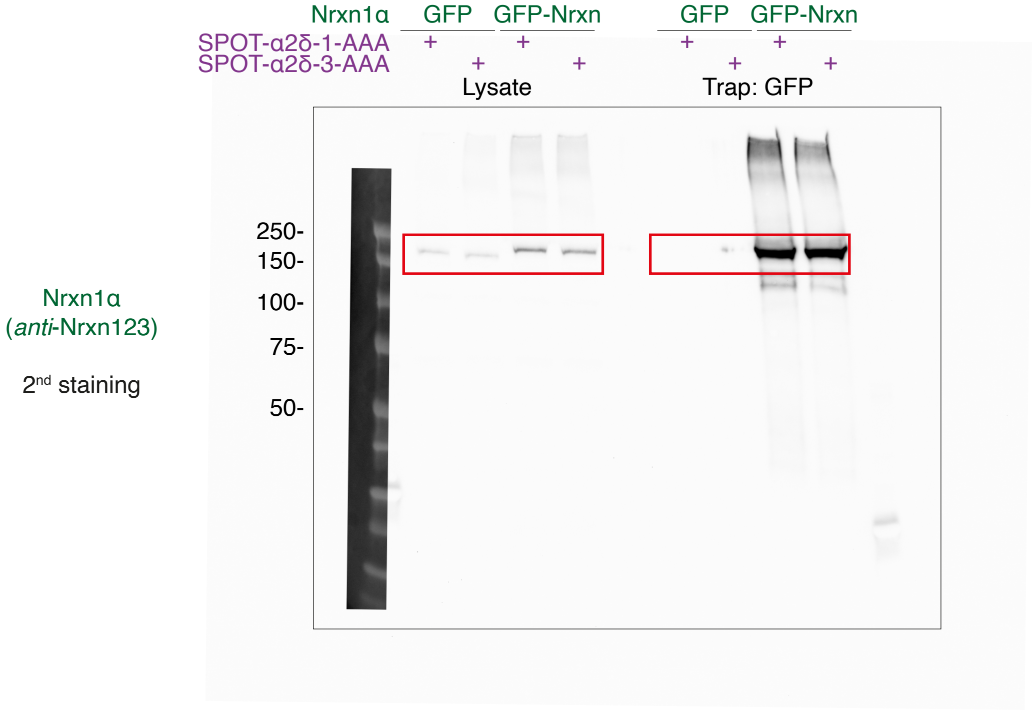

Supp. Figure S4

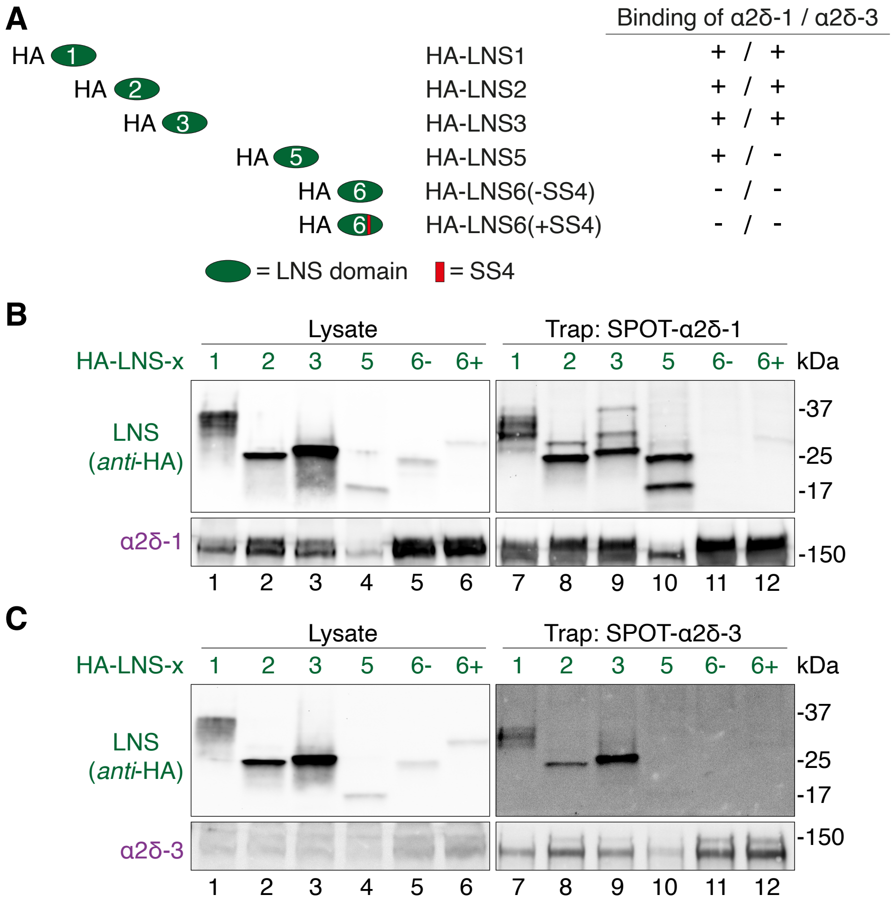

Supp. Figure S4B, panel 1, filename: 2026-04-28 09.37.02,3-2. scan_raw.png

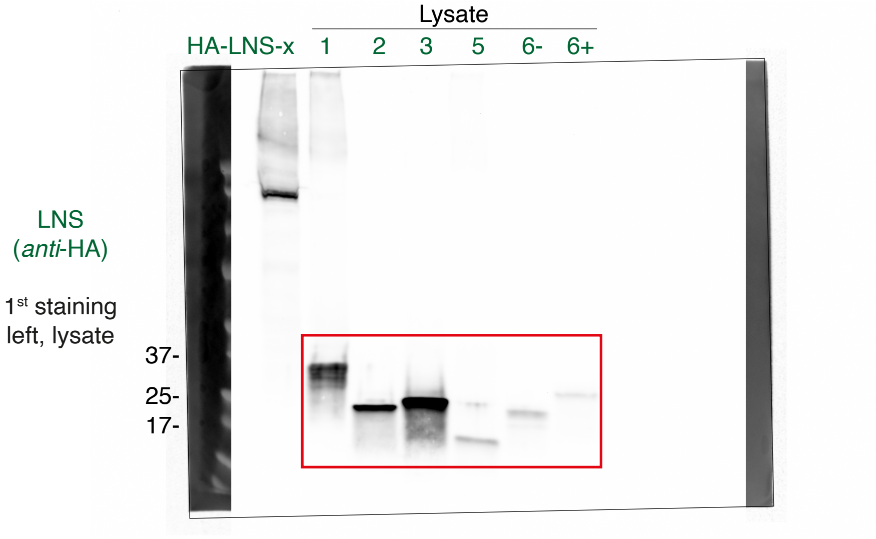

Supp. Figure S4B, panel 1, filename: 2024-06-04 10.19.32,3-2. scan_raw.png

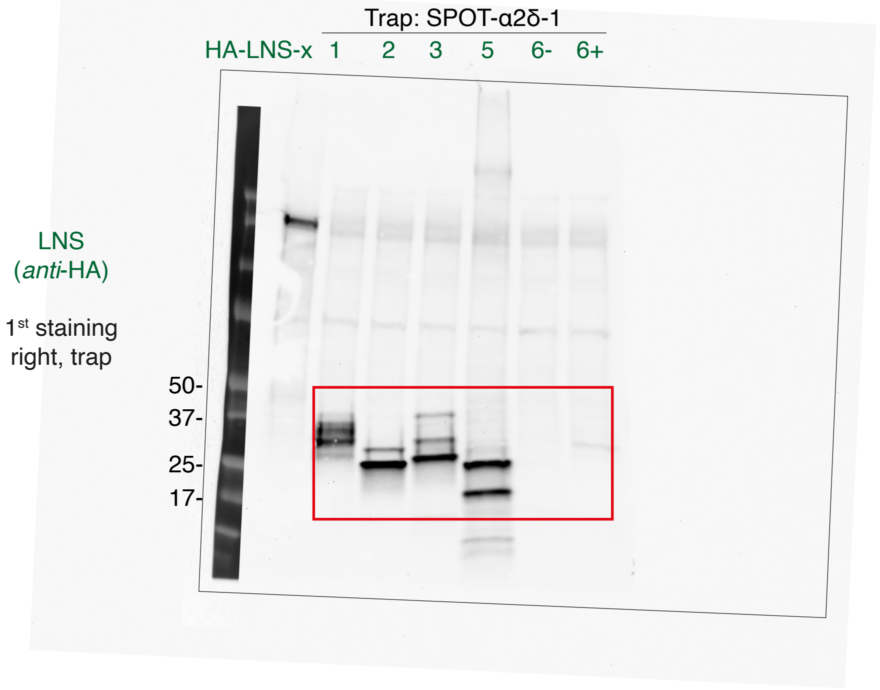

Supp. Figure S4B, panel 2, filename: 2026-04-29 10.43.00,3-2. scan_raw Kopie.png

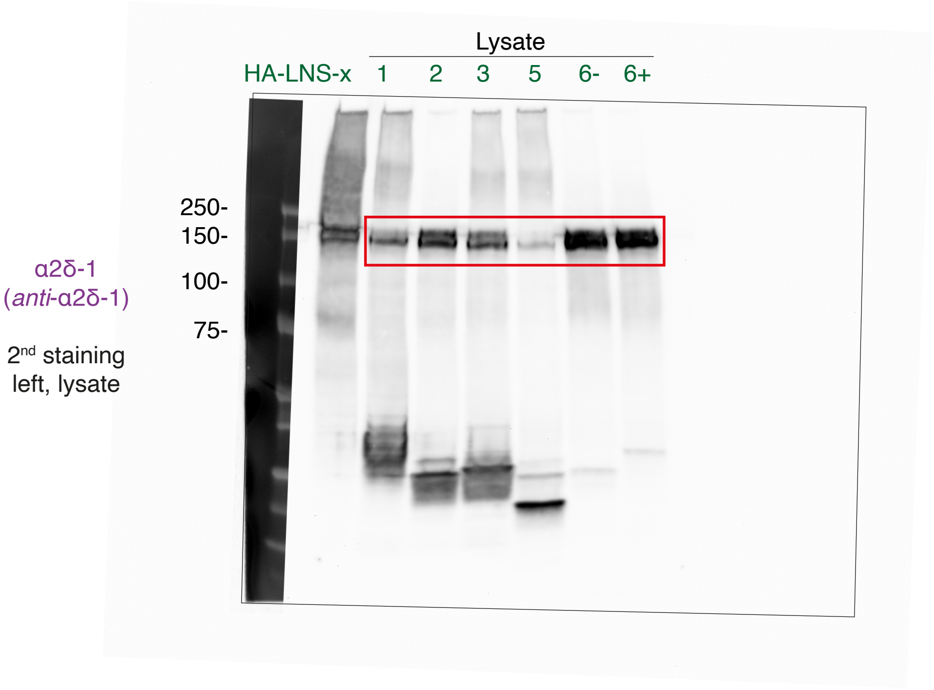

Supp. Figure S4B, panel 2, filename: 2024-06-05 11.02.05,1-1. scan_raw.png

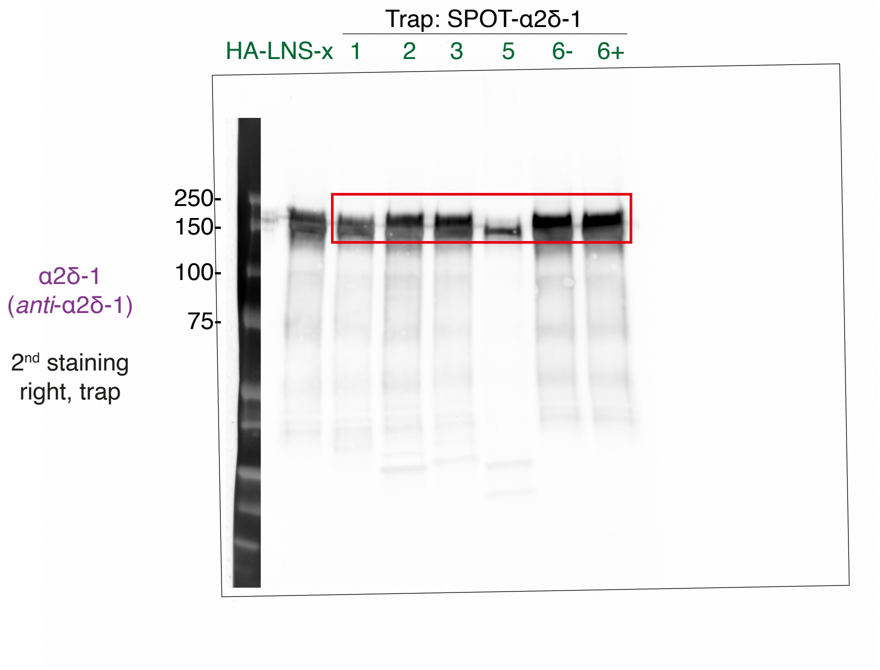

Supp. Figure S4C, panel 1, filename: 2024-08-01 11.04.30,3-2. scan_raw.png

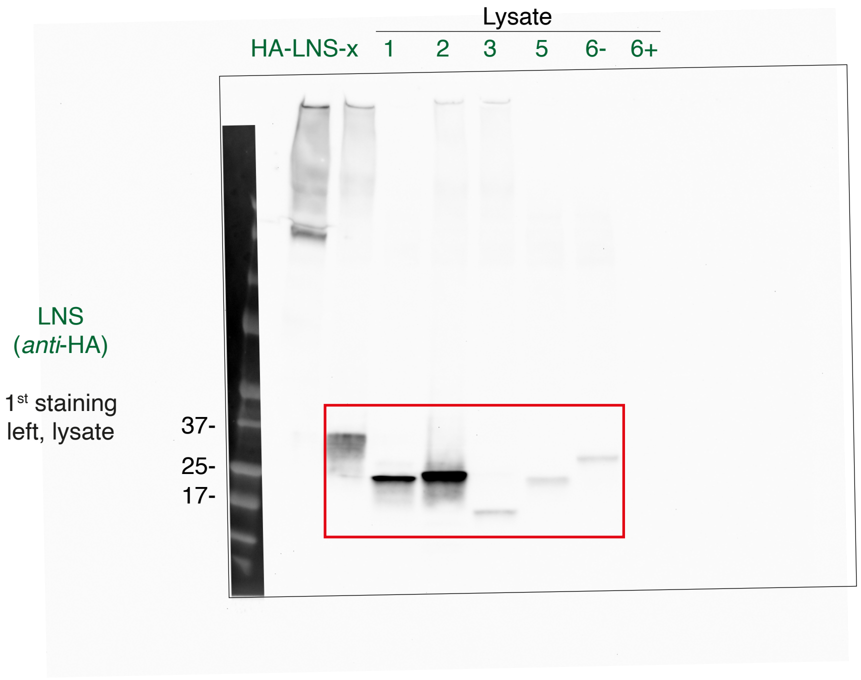

Supp. Figure S4C, panel 1, filename: 2024-08-01 11.27.21,5-3. scan_raw.png

LNS
(*anti*-HA)

1st staining
right, trap

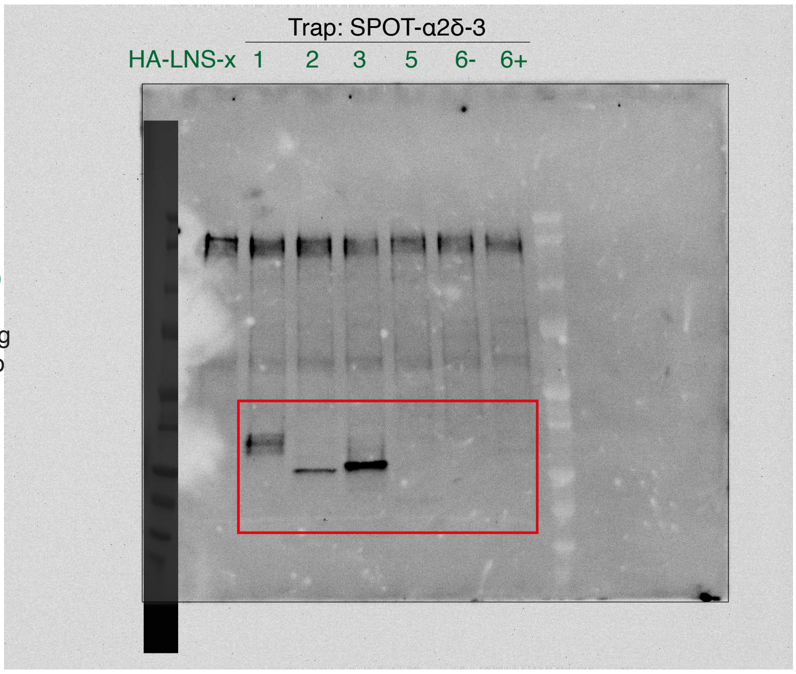

Supp. Figure S4C, panel 2, filename: 2024-08-02 10.10.15,3-2. scan_raw.png

α2δ-3
(*anti*-SPOT)

2nd staining
left, lysate

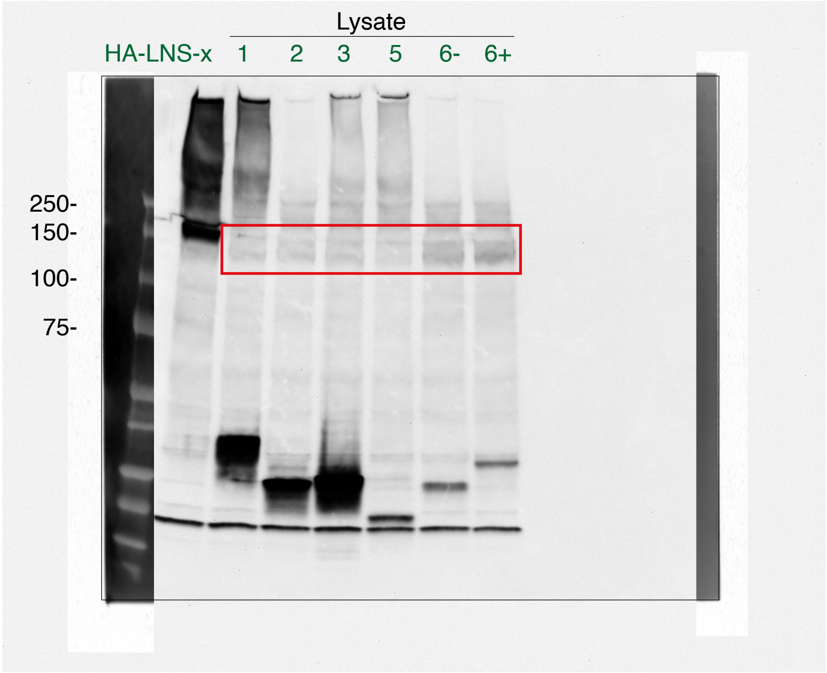

Supp. Figure S4C, panel 2, filename: 2024-08-02 10.10.15,3-2. scan_raw.png

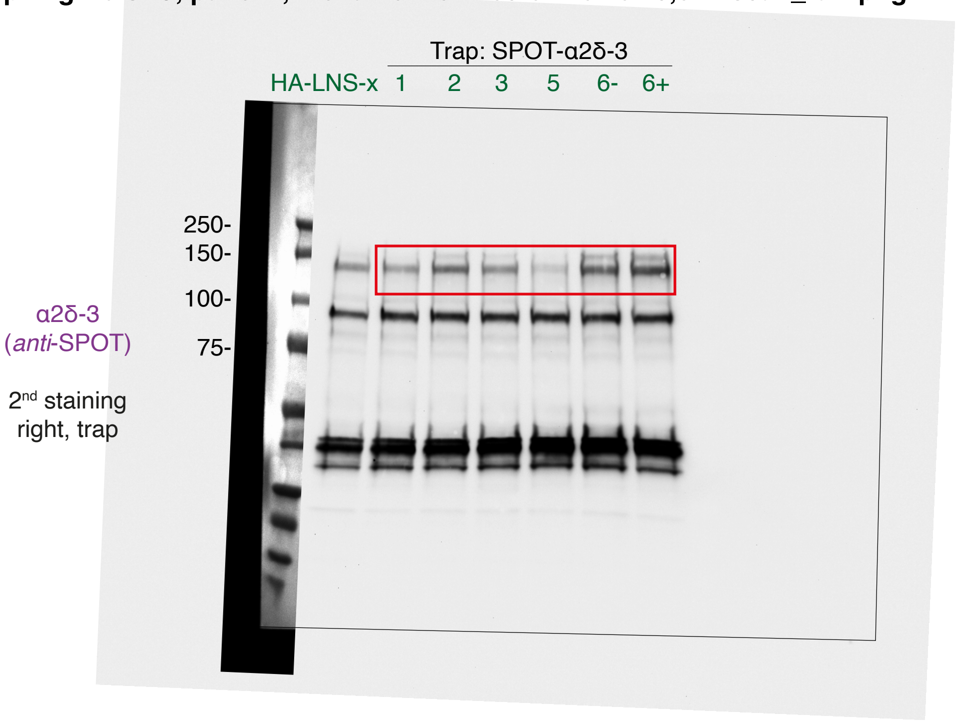

Supp. Figure S5A

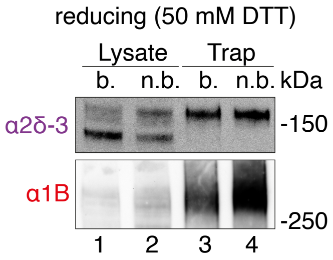

Figure S5A, panel 1, filename: 2021-03-03 13.01.51,5-3. scan_raw.png

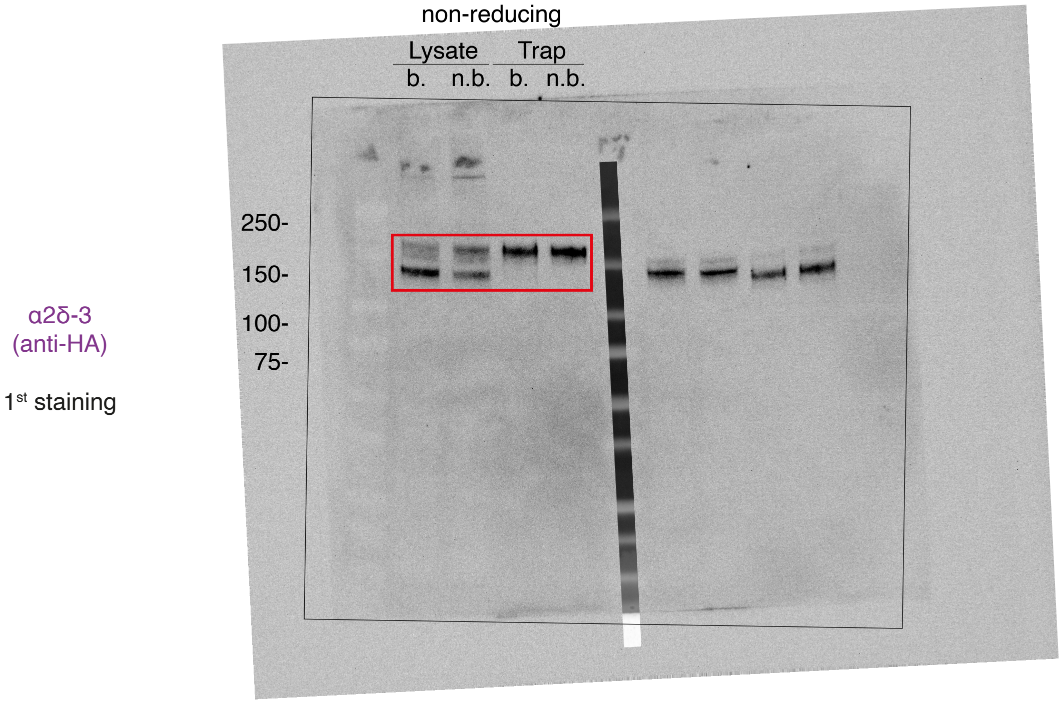

Figure S5A, panel 2, filename: 2021-03-04 12.03.36,5-3. scan_raw.png

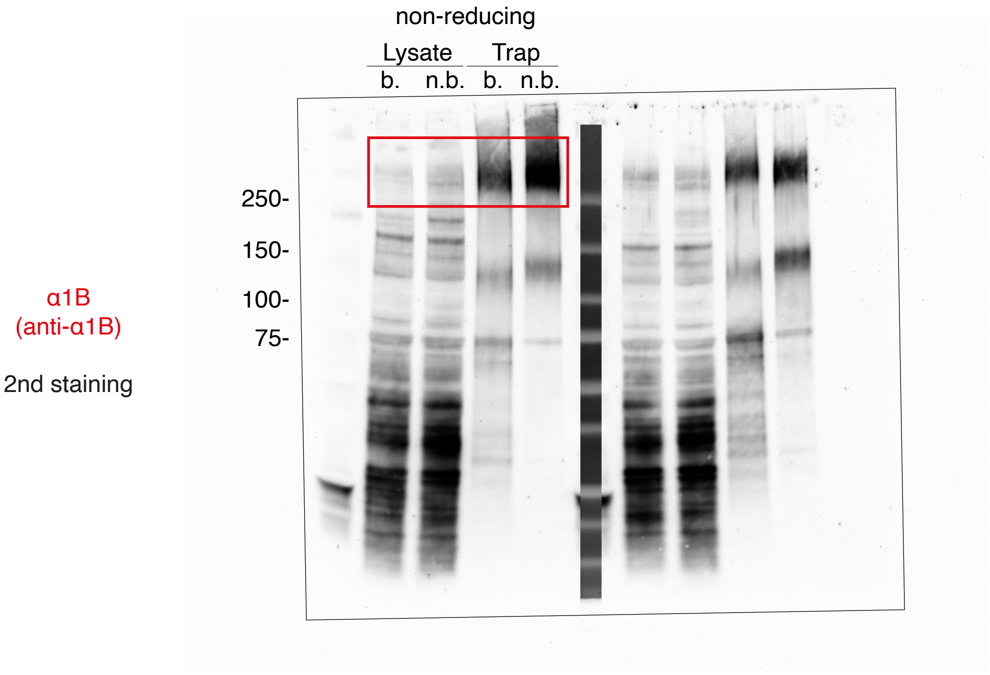

Supp. Figure S5B

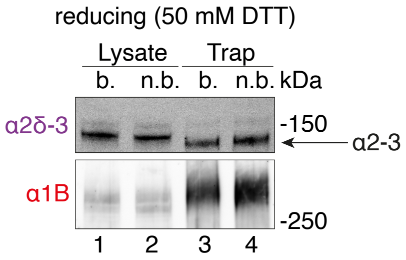

Figure S5B, panel 1, filename: 2021-03-03 13.01.51,5-3. scan_raw.png

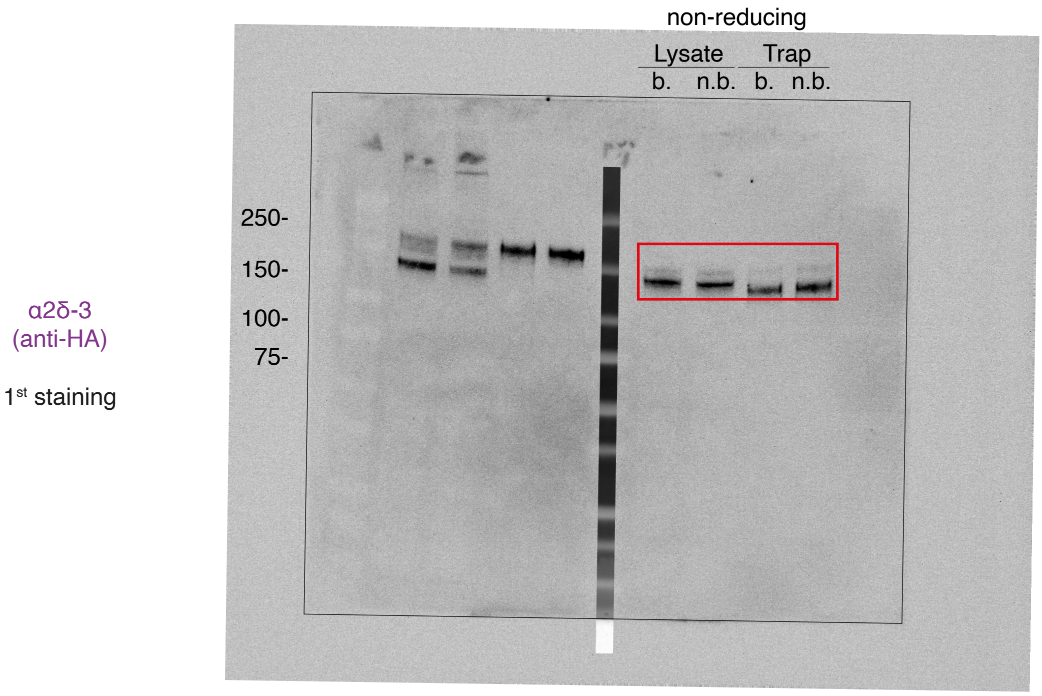

Figure S5B, panel 2, filename: 2021-03-04 12.03.36,5-3. scan_raw.png

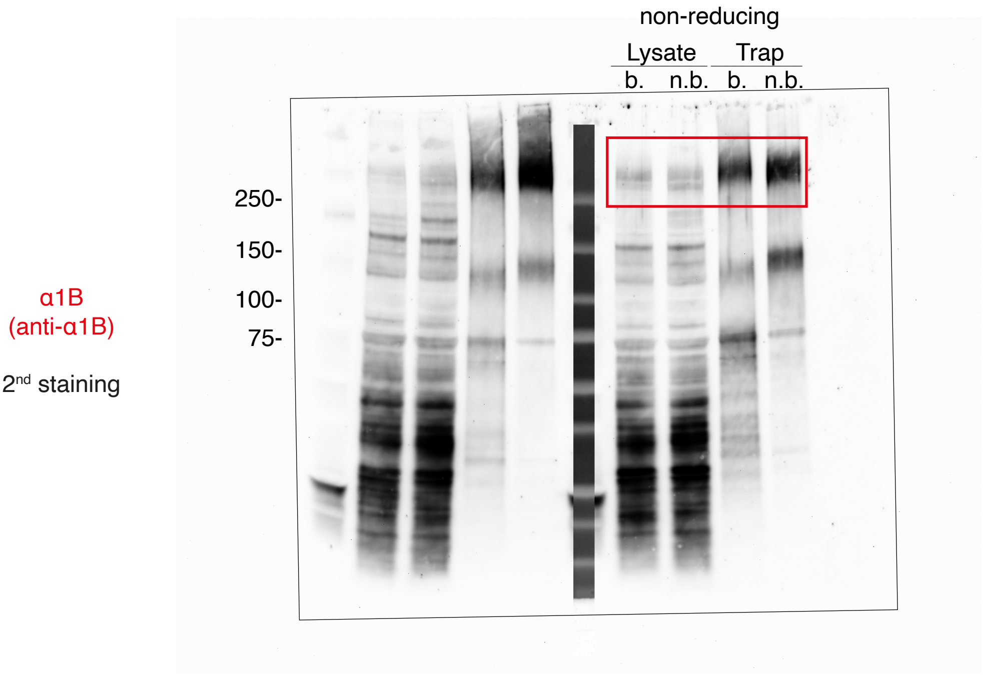

Supp. Figure S6

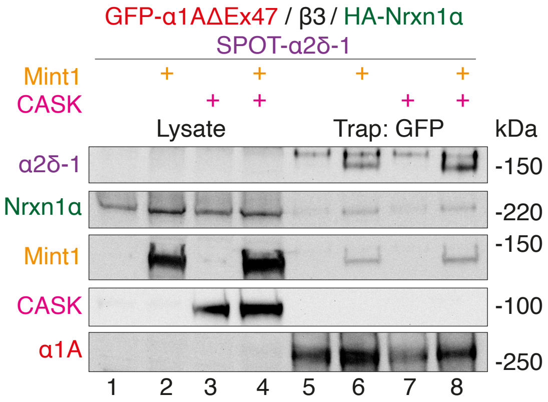

Figure S6, panel 2, filename: 2022-04-05 13.10.36,5-3. scan_raw.png

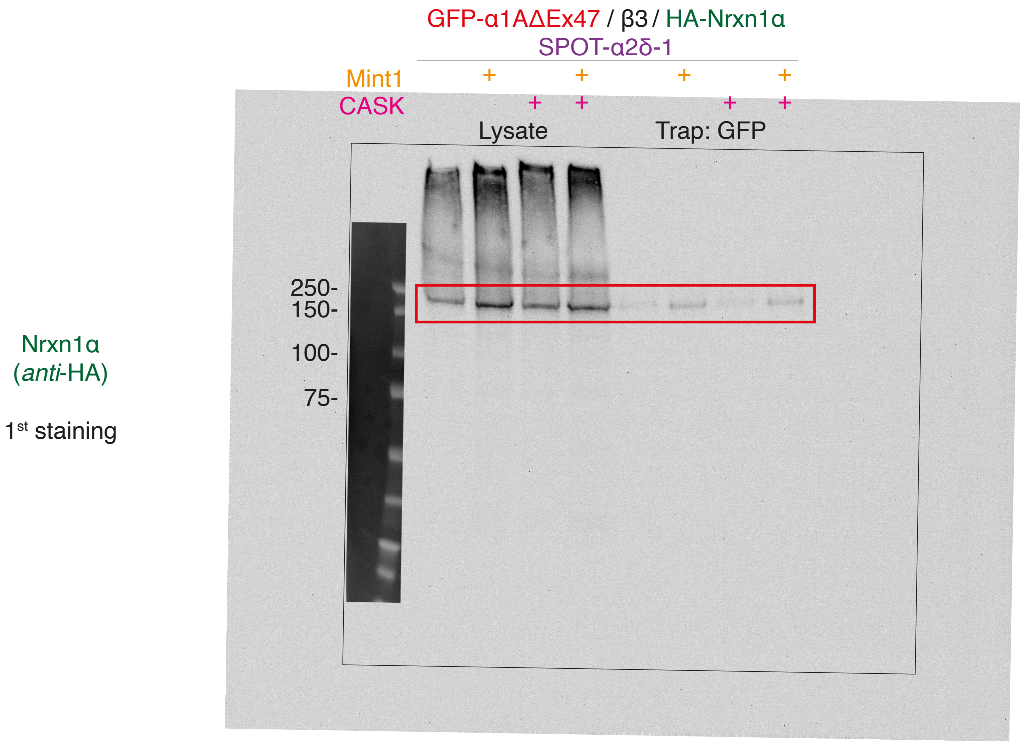

Figure S6, panel 1, filename: 2022-04-16 10.13.38,5-3. scan_raw.png

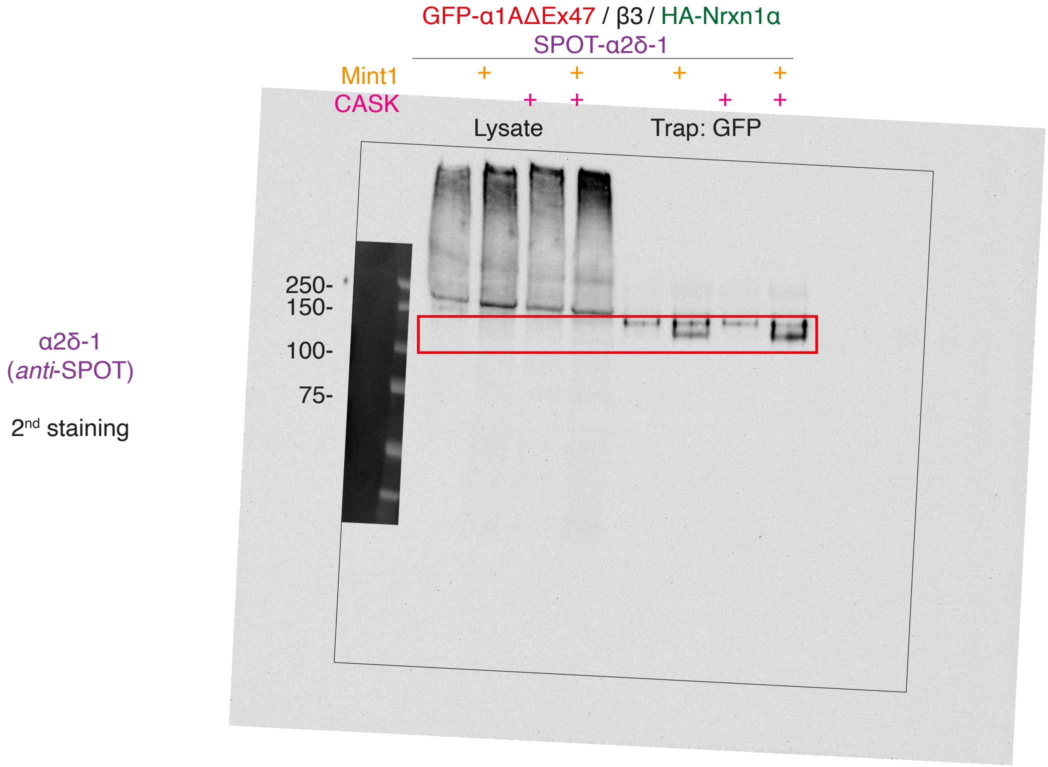

Figure S6, panel 3, filename: 2022-04-07 11.28.24,3-2. scan_raw.png

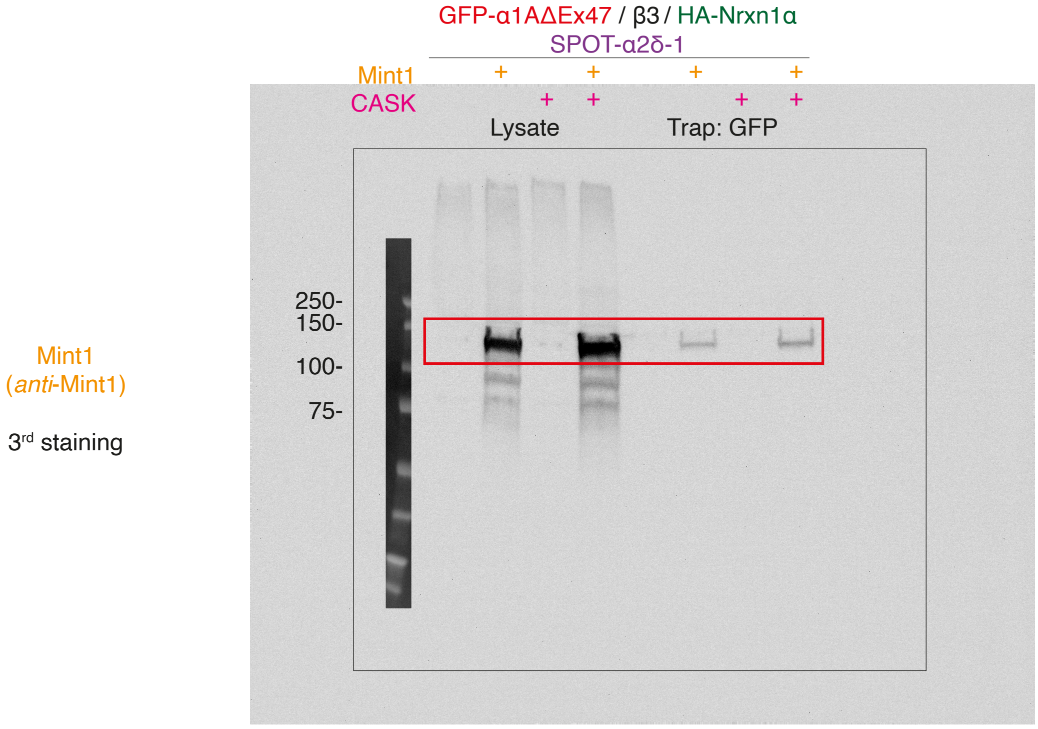

Figure S6, panel 5, filename: 2022-04-20 10.38.51,5-3. scan_raw.png

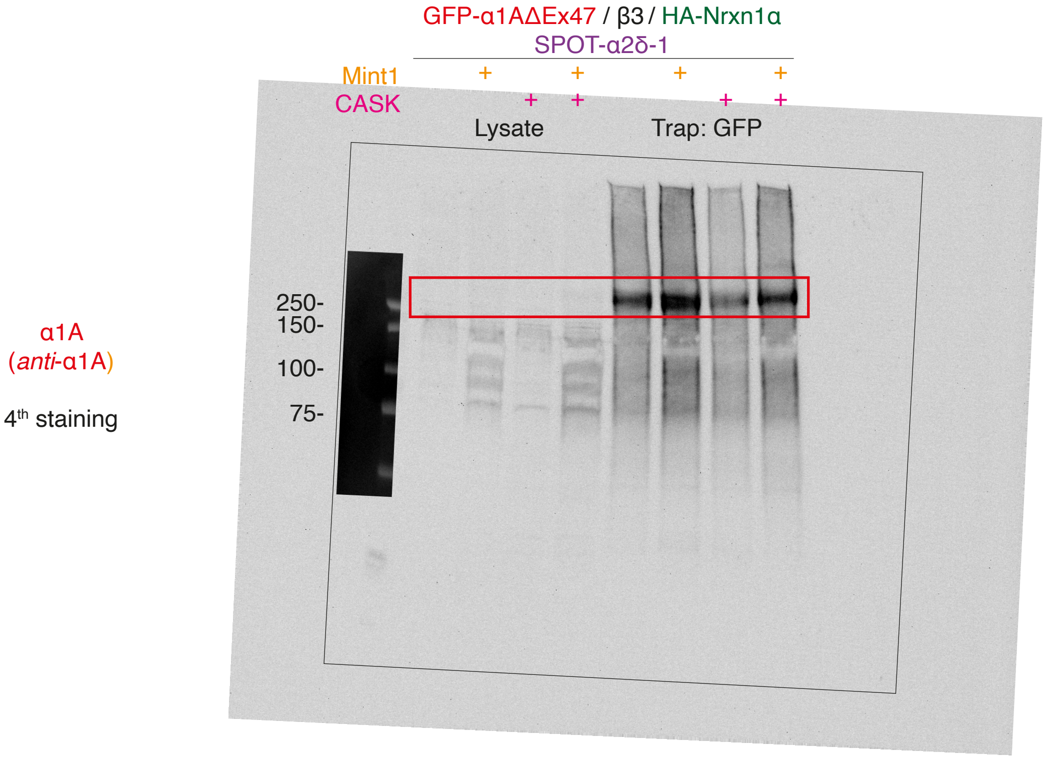

Figure S6, panel 4, filename: 2022-05-17 10.17.09,5-3. scan_raw.png

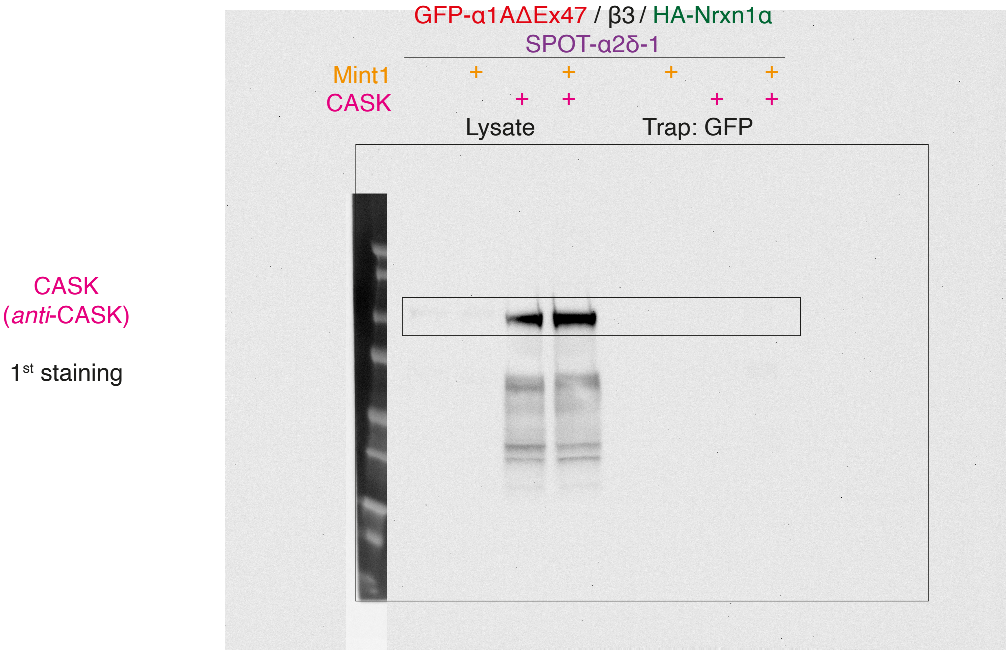

Supp. Figure S7

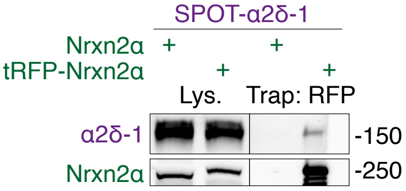

Figure S7, panel 1 Lysate, filename: 2024-04-04 10.33.38,1-1. scan_raw_lysate.png

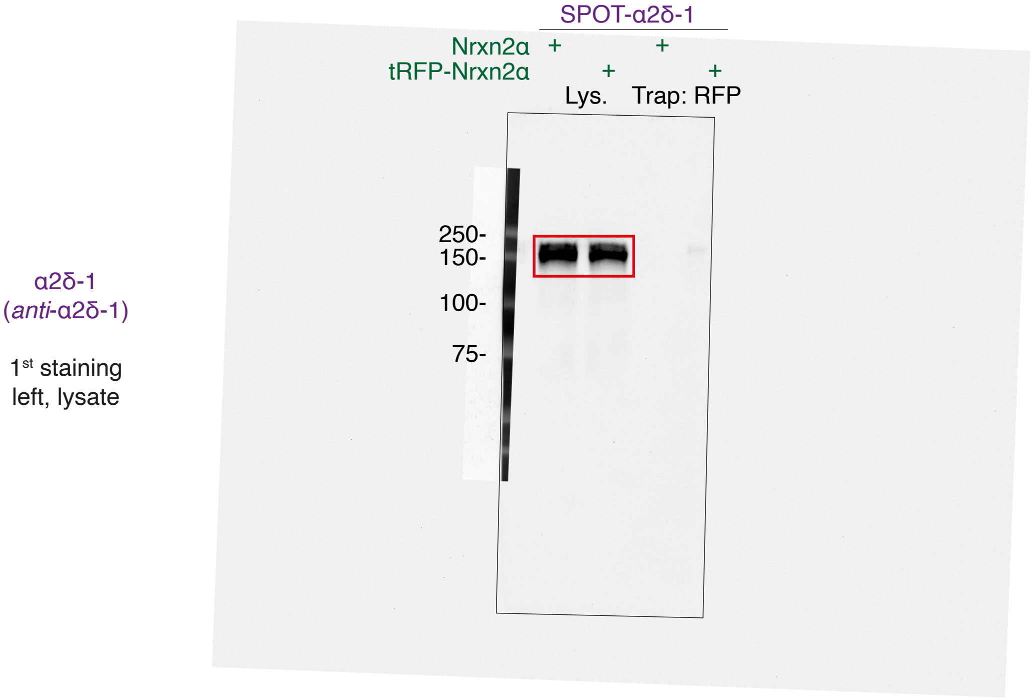

Figure S7, panel 1 Trap, filename: 2024-04-04 10.33.38,5-3. scan_raw_trap.png

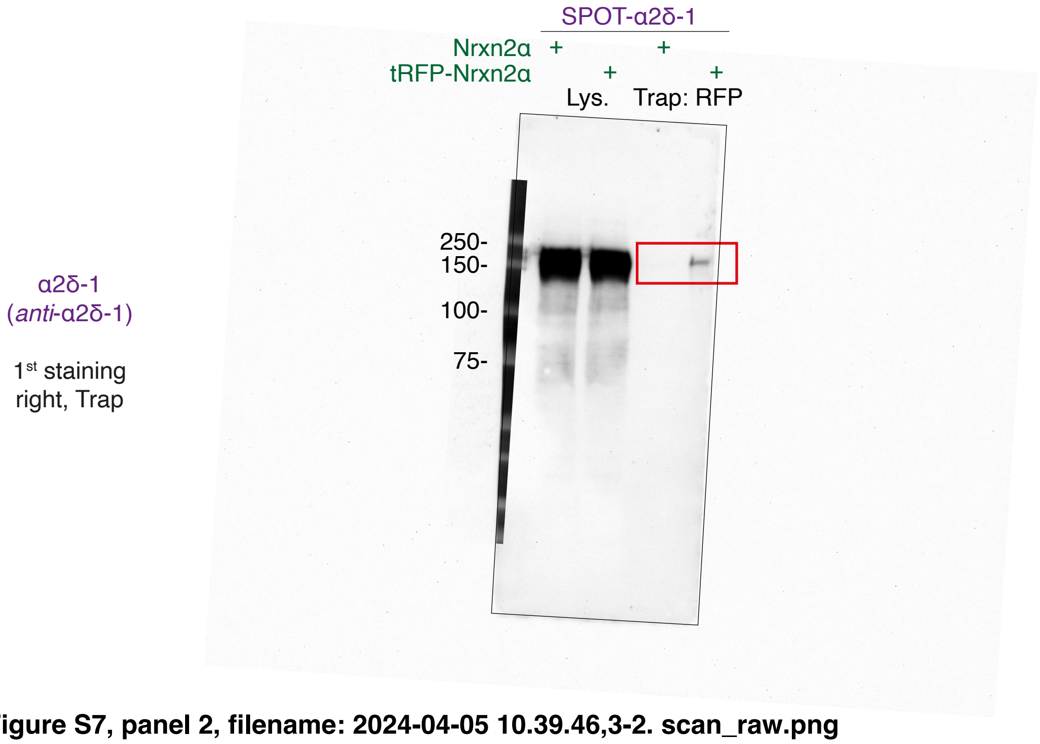

Figure S7, panel 2, filename: 2024-04-05 10.39.46,3-2. scan_raw.png

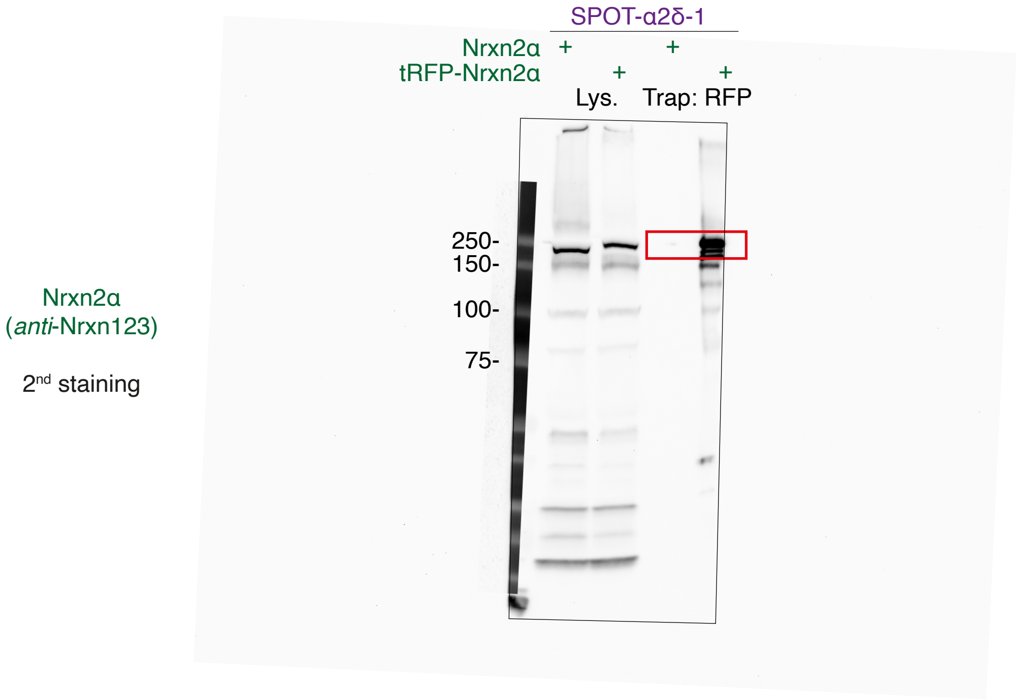

Supp. Figure S8

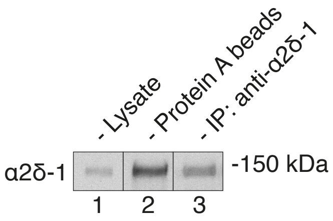

Figure S8, filename: 2022-04-12 09.58.37,5-3. scan_raw Kopie.png

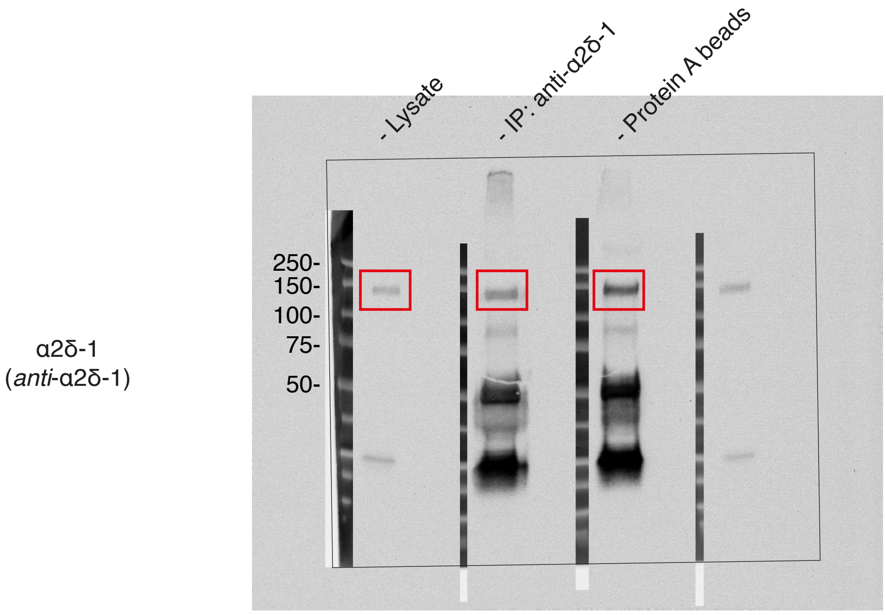

Supplement: Supplementary file 2 — Supplementary Material 2 [file 41598_2026_62077_MOESM2_ESM.pdf]
